# Supplementary material for: Autumn ichthyoplankton assemblage in the Yangtze Estuary shaped by environmental factors
Source: PeerJ. 2016 Apr 19;4:e1922. doi: 10.7717/peerj.1922 (PMC4841249; doi:10.7717/peerj.1922)
Supplement: Supplemental Information 1 [file peerj-04-1922-s001.zip › GB12763-2007/GB¿MT 12763.1-2007 ║ú╤≤╡≈▓Θ╣μ╖╢ ╡┌01▓┐╖╓ú║╫▄╘≥.pdf]

ICS 07.060
A 45

中华人民共和国国家标准

GB/T 12763.1—2007
代替 GB/T 12763.1—1991

海洋调查规范
第1部分:总则

**Specifications for oceanographic survey—
Part 1:General**

2007-08-13 发布

2008-02-01 实施

中华人民共和国国家质量监督检验检疫总局
中国国家标准化管理委员会
发布

目　次

前言 …………………………………………………………………………………… Ⅲ
1　范围 …………………………………………………………………………………… 1
2　规范性引用文件 ……………………………………………………………………… 1
3　调查基本程序 ………………………………………………………………………… 1
3.1　项目委托与合同签订阶段 ………………………………………………………… 1
3.2　调查准备阶段 ……………………………………………………………………… 1
3.3　海上作业阶段 ……………………………………………………………………… 2
3.4　样品分析阶段 ……………………………………………………………………… 2
3.5　资料处理与调查报告编写阶段 …………………………………………………… 2
3.6　调查成果的鉴定与验收阶段 ……………………………………………………… 2
4　项目委托与合同签订 ………………………………………………………………… 2
4.1　项目委托 …………………………………………………………………………… 2
4.2　合同签订 …………………………………………………………………………… 2
4.3　合同修改 …………………………………………………………………………… 2
5　调查质量控制 ………………………………………………………………………… 2
5.1　建立质量控制工作体系 …………………………………………………………… 2
5.2　实施全过程质量控制 ……………………………………………………………… 3
5.3　调查人员质量控制 ………………………………………………………………… 3
6　调查计划编制 ………………………………………………………………………… 3
6.1　编制单位 …………………………………………………………………………… 3
6.2　技术设计 …………………………………………………………………………… 3
6.3　编制原则 …………………………………………………………………………… 3
6.4　主要内容 …………………………………………………………………………… 3
6.5　计划的报批 ………………………………………………………………………… 3
6.6　调查计划的调整 …………………………………………………………………… 4
7　调查人员 ……………………………………………………………………………… 4
7.1　调查人员组织 ……………………………………………………………………… 4
7.2　调查人员条件和职责 ……………………………………………………………… 4
8　调查用计量器具要求 ………………………………………………………………… 4
8.1　海洋调查仪器设备 ………………………………………………………………… 4
8.2　标准物质 …………………………………………………………………………… 5
9　实验室、船舶、飞机、浮标和潜标 ………………………………………………… 5
9.1　实验室 ……………………………………………………………………………… 5
9.2　调查船 ……………………………………………………………………………… 5
9.3　飞机 ………………………………………………………………………………… 6
9.4　浮标和潜标 ………………………………………………………………………… 6
10　调查的导航定位 …………………………………………………………………… 7
11　海上作业 …………………………………………………………………………… 7

标准分享网 www.bzfxw.com 免费下载

11.1 时间标准…… 7
11.2 观测作业要求…… 7
11.3 原始记录…… 8
11.4 样品及观测记录的包装、存储和运输 …… 8
11.5 安全措施…… 8
11.6 航次报告编写…… 8
11.7 原始资料和样品的验收…… 8
12 室内样品分析测试和鉴定…… 9
12.1 实验室资质…… 9
12.2 样品交接与记录…… 9
12.3 样品的惟一性标识…… 9
12.4 样品的预处理和分析测试鉴定…… 9
12.5 分析测试鉴定的质量检查…… 9
12.6 分析测试鉴定结果的发出…… 9
12.7 剩余样品和标样的处置…… 9
13 资料处理…… 9
13.1 资料汇总…… 9
13.2 数据处理…… 9
13.3 建立文档和图件绘制…… 9
13.4 计算机处理资料的要求 …… 10
14 调查报告编写 …… 10
14.1 编写内容 …… 10
14.2 编写要求 …… 11
15 调查资料和成果归档 …… 11
15.1 归档范围 …… 11
15.2 归档要求 …… 12
15.3 归档时间 …… 12
15.4 档案质量要求 …… 12
16 成果鉴定和验收 …… 12
16.1 成果鉴定 …… 12
16.2 成果验收 …… 12
附录A(规范性附录) 航次报告 …… 13
附录B(规范性附录) 海洋调查人员登记表 …… 18
附录C(规范性附录) 海洋调查仪器设备检查登记表 …… 19
附录D(规范性附录) 标准物质及化学试剂检查登记表 …… 20

前言

GB/T 12763《海洋调查规范》分为11个部分：

——第1部分：总则；

——第2部分：海洋水文观测；

——第3部分：海洋气象观测；

——第4部分：海水化学要素调查；

——第5部分：海洋声、光要素调查；

——第6部分：海洋生物调查；

——第7部分：海洋调查资料交换；

——第8部分：海洋地质 地球物理调查；

——第9部分：海洋生态调查指南；

——第10部分：海底地形地貌调查；

——第11部分：海洋工程地质调查。

其中第9部分、第10部分和第11部分对应于GB/T 12763—1991是新增部分。

本部分为GB/T 12763的第1部分，代替GB/T 12763.1—1991《海洋调查规范 第1部分：总则》。

本部分与GB/T 12763.1—1991相比主要变化如下：

——删除了1991年版"术语"的内容；

——增加了"项目委托与合同签订"一章(见第4章)；

——"海洋调查质量控制"一章中增加了对质量计划的要求(见5.1.2)；

——"海洋调查计划编制"一章中明确了调查计划的编制单位和技术依据(见6.1和6.2)，增加了分包计划、人员组织和质量计划的内容(1991年版6.3；本版6.4)；

——"海洋调查人员"一章修改了对调查人员的要求：强调具有上岗资质证书、法律知识和质量意识(1991年版11.2；本版7.2)；

——修改了对海洋调查仪器设备的要求：增加了仪器检定、校准和自校(互校)的内容(1991版10.2和10.4；本版8.1)，删除了对传感器的要求(1991版10.3)；

——增加了对船上和岸上实验室的要求(见9.1)；

——对潜标和浮标的要求中增加了防雷击的要求(见9.4.2)；

——修改了对导航定位的要求和准确度规定(1991版第9章；本版第10章)；

——修改了对海上作业的要求：时间标准中明确了记时的误差(1991版7.1；本版11.1.2)，增加了对海上作业的观测要求(见11.2)，并调整和修改了原始资料和样品的验收(1991版第12章；本版见11.7)；

——增加了室内样品分析与测试的内容(见第12章)；

——调整并细化了资料处理的内容(1991版13.2；本版第13章)；

——海洋调查报告中增加了质量计划实施情况报告的要求(见14.1.5)；

——海洋调查资料和成果归档中增加了对档案的质量要求(见15.4)；

——增加了规范性附录"航次报告"(见附录A)；

——增加了规范性附录"海洋调查人员登记表"(见附录B)；

——修改了规范性附录"海洋调查仪器设备检查登记表"(1991版附录A；本版附录C)；

——增加了规范性附录"标准物质及化学试剂检查登记表"(见附录D)。

标准分享网 www.bzfxw.com 免费下载

本部分与 GB/T 12763 的其他部分配套使用。

本部分的附录 A、附录 B、附录 C、附录 D 是规范性附录。

本部分由国家海洋局提出。

本部分由国家海洋标准计量中心归口。

本部分由国家海洋标准计量中心负责起草，国家海洋局第一海洋研究所、国家海洋局第二海洋研究所参加起草。

本部分主要起草人：秦嗣仁、康寿岭、路应贤、杨哲玲、姚勇、雷富、郭小勇、柯长志、叶盛林。

本标准所代替标准的历次发布情况为：

——GB/T 12763.1—1991。

海洋调查规范
第1部分：总则

1 范围

GB/T 12763的本部分规定了海洋环境基本要素调查的基本程序、质量控制、计划编制、资源配置、调查作业、资料处理、报告编写、资料归档和成果鉴定与验收中的基本要求。

本部分适用于海洋环境基本要素调查的组织管理。

2 规范性引用文件

下列文件中的条款通过GB/T 12763的本部分的引用而成为本部分的条款。凡是注日期的引用文件，其随后所有的修改单(不包括勘误的内容)或修订版均不适用于本部分，然而，鼓励根据本部分达成协议的各方研究是否使用这些文件的最新版本。凡是不注日期的引用文件，其最新版本适用于本部分。

GB/T 12763.2 海洋调查规范 第2部分：海洋水文观测

GB/T 12763.3 海洋调查规范 第3部分：海洋气象观测

GB/T 12763.4 海洋调查规范 第4部分：海水化学要素调查

GB/T 12763.5 海洋调查规范 第5部分：海洋声、光要素调查

GB/T 12763.6 海洋调查规范 第6部分：海洋生物调查

GB/T 12763.7 海洋调查规范 第7部分：海洋调查资料交换

GB/T 12763.8 海洋调查规范 第8部分：海洋地质与地球物理调查

GB/T 12763.9 海洋调查规范 第9部分：海洋生态调查指南

GB/T 12763.10 海洋调查规范 第10部分：海底地形地貌调查

GB/T 12763.11 海洋调查规范 第11部分：海洋工程地质

GB/T 15481 检测和校准实验室能力的通用要求

GB/T 15527 船用全球定位系统(GPS)接收机通用技术条件

GB/T 17424 差分全球定位系统(DGPS)技术要求

HY/T 058 海洋调查观测监测档案业务规范

3 调查基本程序

3.1 项目委托与合同签订阶段

工作内容包括：

a) 委托项目；

b) 评审合同；

c) 签订合同。

3.2 调查准备阶段

工作内容包括：

a) 确定调查项目负责人；

b) 收集、分析调查海区与调查任务有关的文献、资料；

c) 确定首席科学家(或调查技术负责人)；

d) 进行技术设计，编写调查计划，报项目委托单位审批；

标准分享网 www.bzfxw.com 免费下载

e) 组织调查队伍,明确岗位责任;

f) 做好资源配置,申报航行计划,做好出海准备。

3.3 海上作业阶段

工作内容包括:

a) 获取现场资料和样品;

b) 编写航次报告,见附录 A 中的表 A.1～表 A.6;

c) 验收本航次原始资料和样品。

3.4 样品分析阶段

工作内容包括:

a) 验收、交接、预处理样品;

b) 分析、测试与鉴定样品;

c) 处理数据与样品处置。

3.5 资料处理与调查报告编写阶段

工作内容包括:

a) 验收原始资料;

b) 处理资料与编制资料报表;

c) 编绘成果图件;

d) 编写调查报告。

3.6 调查成果的鉴定与验收阶段

工作内容包括:

a) 调查资料和成果的归档;

b) 调查成果的鉴定和验收。

4 项目委托与合同签订

4.1 项目委托

4.1.1 项目委托应以合同委托方式或下达任务方式办理,委托书或任务书由项目委托单位提出。项目委托单位可以是各级政府相应主管部门,也可以是企、事业单位。

4.1.2 项目承担单位应是独立法人或由其授权的,并具有相应资质的海洋技术机构。该机构应按 GB/T 15481 的规定建立适宜本单位的质量管理体系并保持有效运行。承担向社会出具公正数据的调查任务的单位,应通过计量认证。

4.2 合同签订

项目承担单位应按《中华人民共和国合同法》及本标准的要求起草合同书,并组织评审,在与项目委托单位达成共识的基础上,签订合同。

4.3 合同修改

需要修改合同时,应重新进行评审,经双方协商达到一致,并保留记录。

5 调查质量控制

5.1 建立质量控制工作体系

5.1.1 项目承担单位应接受项目委托单位和技术监督机构的监督。

5.1.2 项目承担单位应将调查过程的质量控制纳入本单位的质量体系运行,并根据本单位的质量体系和调查项目要求制定质量计划。

5.1.3 项目负责人应指定质量负责人负责建立调查过程的质量管理工作体系。

5.2 实施全过程质量控制

5.2.1 项目承担单位应充分理解委托项目的质量目标。

5.2.2 对采用的已有文献、资料应就合法性、单位制、溯源性、准确度、时效性、可靠性、容量、密度及适用区域等有明确的质量要求，并进行具体质量分析、评估。

5.2.3 技术设计应进行测量准确度估算，保证调查项目诸要素实现调查技术指标。

5.2.4 在规定有效期内按规定的程序使用符合质量要求的仪器、设备、工具和材料。

5.2.5 对海上获取的样品、资料进行现场质量检查。

5.2.6 航次结束后，应对原始资料、样品进行全面质量验收。

5.2.7 对样品的分析、测试、鉴定结果和资料处理结果进行质量检查。

5.2.8 文件、资料成果归档应符合归档要求。

5.2.9 调查项目应通过验收，科技成果应通过鉴定。

5.3 调查人员质量控制

5.3.1 调查人员应符合 7.2 的要求，并经必要的岗前培训。

5.3.2 对调查人员应明确质量责任，并进行质量意识教育。

6 调查计划编制

6.1 编制单位

签订合同书或收到项目任务书后，项目承担单位应在技术设计的基础上编制调查计划。

6.2 技术设计

首席科学家负责技术设计，技术设计应按 GB/T 12763.2～12763.6、GB/T 12763.8～12763.11 中的有关规定进行，工作内容应满足项目任务书或合同的要求。

6.3 编制原则

调查计划编制应遵循下列原则：

a) 符合合同书的要求和 GB/T 12763 的规定；

b) 规定相应的资源配置；

c) 充分利用已有的具有溯源性的文献和资料；

d) 提高效益、减少损耗，充分利用资源，进行综合调查。

6.4 主要内容

调查计划的内容主要包括：

a) 任务描述及任务来源；

b) 技术设计；

c) 分包计划；

d) 人员组织；

e) 时间安排；

f) 条件保证；

g) 质量计划；

h) 安全措施；

i) 经费预算。

6.5 计划的报批

6.5.1 调查计划的报批

由项目承担单位按规定的时间将调查计划报项目委托单位。项目委托单位批复后即可实施。

6.5.2 航(飞)行计划的报批

按时向船舶(飞机)主管部门申报航(飞)行计划，待批复后实施。

标准分享网 www.bzfxw.com 免费下载

6.6 调查计划的调整

6.6.1 海洋调查计划应严格执行，海洋调查计划确需改动时，应报项目委托单位批准。

6.6.2 进行海洋调查作业时，在遵循最佳效益和确保安全的原则下，首席科学家根据实际情况可对站位、观测对象及作业程序进行适当调整、补充；同时将调整情况报项目委托单位。

7 调查人员

7.1 调查人员组织

7.1.1 由项目承担单位确定调查项目负责人和首席科学家。

7.1.2 调查项目负责人根据调查项目的实际需要，组成结构合理的调查队，确定调查人员，填写《海洋调查人员登记表》，见附录B中的表B.1，按规定渠道报海洋技术监督机构和项目委托单位核准备案。

7.1.3 首席科学家应对上船的调查人员，按专业和技术水平合理分工，明确岗位职责；必要时，首席科学家可以指定一名人员，负责本航次的质量保证工作。

7.2 调查人员条件和职责

7.2.1 调查项目负责人基本条件和职责如下：

a) 具有与调查项目相符的业绩和良好的组织领导能力；掌握本项目重点学科的基本理论、专业知识，正确解释调查结果中出现的现象；熟悉国家相关法律、法规，具有较强的质量意识；具备高级专业技术职称；

b) 全面负责本调查项目的组织领导工作与资源配置，保证调查项目按时完成和成果质量。

7.2.2 首席科学家基本条件和职责如下：

a) 应取得由合法资质机构颁发的且与调查项目相符的上岗资质证书，具备高级专业技术职称；应掌握本航次重点学科的基础理论、专业知识与主要专业操作技能，能正确处理调查作业中出现的问题；应熟悉国家相关法律、法规，具有较强的质量意识；

b) 负责本航次调查活动的技术领导，能保证完成本航次调查任务；

c) 负责质量计划的实施，保证海上调查数据、样品的完整和准确可靠，保证实现本航次质量目标。

7.2.3 在综合调查中设学科负责人，其基本条件和职责如下：

a) 应取得由合法资质机构颁发的且与调查项目相符的上岗资质证书，具备中级以上专业技术职称；应掌握本学科调查基础理论、专业知识与主要要素的操作技能，能正确处理调查作业中出现的问题；

b) 在首席科学家领导下，负责顺利完成本学科海上作业和返航后样品分析与数据处理等内业活动，负责向调查项目负责人提交该航次本学科的完整调查资料；

c) 负责实施质量计划相关条款，保证本学科样品、调查数据的完整和准确可靠，保证实现质量目标。

7.2.4 作业人员基本条件和职责如下：

a) 应取得由合法资质机构颁发的与调查项目相符的上岗资质证书，能胜任海上调查工作，坚守岗位，尽职尽责；

b) 应按GB/T 12763规定的方法和技术要求，按时完成本职调查工作，保证调查数据的准确可靠。

8 调查用计量器具要求

8.1 海洋调查仪器设备

8.1.1 技术设计时应进行测量准确度估算，保证调查用仪器设备的技术指标满足海洋调查项目的要求。

8.1.2 国产仪器设备的生产单位应取得由国家有关质量技术监督部门颁发的《制造计量器具许可证》

或型式批准证书;属研制、开发的科研样机的仪器设备应经授权的国家法定计量机构鉴定合格。

8.1.3 进口仪器设备应经国务院计量行政部门型式批准,或由我国或本国权威机构出具质量认定证书。

8.1.4 仪器设备应送授权的法定计量检定机构检定或校准;没有授权机构的,由持有单位按合法化的自校或互校方法进行自校或互校。为保证调查数据质量,仪器设备应在检定、校准证书有效期内使用。

8.1.5 无法在室内检定、校准的仪器设备,应与传统仪器进行现场比对,考察其有效性。

8.1.6 对测量中需定标的仪器,应按规定定标,并列人操作程序。

8.1.7 出海前应由使用者对出海仪器设备按上述条件逐一检查,调查项目负责人组织填写《海洋调查仪器设备检查登记表》,见附录C中的表C.1,报海洋技术监督机构和项目委托单位审核备案。

8.1.8 调查仪器设备的运输、安装、布放、操作、维护,应按其使用说明书的规定进行。

8.2 标准物质

8.2.1 调查中应使用具有《制造计量器具许可证》和定级证书的标准物质。标准物质应标明批号,且在使用有效期内使用。国内没有标准物质产品的项目,应经专门人员、以专用仪器和实验室,用具有出厂检验合格证且在有效使用期内化学试剂配置,并经互校或比对确认符合要求。

8.2.2 出海前由使用者对出海用标准物质和化学试剂按上述条件逐一检查,项目负责人组织填写《标准物质及化学试剂检查登记表》,见附录D中的表D.1,报海洋技术监督机构和项目委托单位审核备案。

9 实验室、船舶、飞机、浮标和潜标

9.1 实验室

9.1.1 无论移动的或固定的实验室(包括观测场及作业场),均应满足如下一般要求:

a) 实验室(包括观测场及作业场)应安排在方便工作、安全操作的地方;
b) 应配有满足调查要求的水、电、照明、排风、消防设施和相应实验、办公设备;
c) 实验室内的温度、湿度、空间、采光等环境参数应符合仪器设备使用与人员安全卫生的规定;
d) 测量、办公用仪器设备应摆放整齐、有序,固定牢固,便于操作;
e) 外界或内部的污染以及机械、噪声、热、光及电磁等干扰水平应对测量结果无显著影响。

9.1.2 实验室应制定周密、切实可行的规章制度,至少应满足如下管理要求:

a) 对仪器设备应实行标志管理:满足原技术指标的加贴绿色合格标志;虽因某项性能消失、某段范围超差或降级使用,但仍能满足使用要求的加贴黄色准用标志;发现故障或超检的加贴红色停用标志;
b) 样品、试剂应按规定包装、储存、分类摆放,稳固有序,标识应清楚,防止混淆、丢失、遗漏、变质及交叉污染;
c) 剧毒、贵重、易燃、易爆物品应以特定程序管理、特殊设施存放;
d) 应建立仪器设备的管理制度,应规定仪器设备交接班检查和定期通电检查、维护的要求;
e) 进出实验室(包括观测场及作业场)或交接班应认真检查水、电、热供应设施是否处于正常开关状态;
f) 应建立三废处理制度,应规定收集、处理、排放废弃物、废水、废气和过期试剂的有关要求;
g) 应建立并保持对有特殊要求(如恒温、恒湿、超净、无菌、电磁屏蔽等)实验室环境条件的测试记录;
h) 应保持实验室(包括观测场及作业场)洁净、整齐、有序。

9.2 调查船

9.2.1 调查船应满足如下一般要求:

a) 应具有适应海洋调查用的甲板及机械设备;

标准分享网 www.bzfxw.com 免费下载

b) 应有满足现场调查作业和现场样品处理、测试、分析与资料整理所需的实验室；

c) 应有调查需要的电源；

d) 应有周密、可靠、有效的航海安全、消防和救生措施及设备；

e) 可在不同航速下连续航行；

f) 应有准确可靠的测深、导航定位系统和通讯系统；

g) 远洋调查船应有较大续航力和自持力，能在广泛的洋区调查作业，并配有全球导航定位系统；

h) 海洋生物调查船应有满足需要的拖网绞车，船尾适于拖网作业；

i) 海洋声、光调查船应噪声低，具有较好的防电磁干扰能力；

j) 极地考察船应具有相应的抗风暴和破冰、抗冰能力。

9.2.2 调查船至少应满足如下管理要求：

a) 应通过船舶和有关检验机构的检查，符合适航标准和安全检查条例；

b) 船长及船员应具有相应职位的资质证书，熟悉本职业务，明确调查任务对船舶的作业要求，并能积极主动地配合调查人员完成调查任务；

c) 应保证调查人员的必要工作条件和生活条件；

d) 应按计划完成备航和安全检查、教育工作，能按时出海作业；在不影响安全的前提下，船舶的行动应尊重首席科学家的意见；

e) 应按调查任务的需要准确地操纵船舶，保证航行安全；

f) 凡属船上的设备，均需经常保持良好状态。

9.3 飞机

9.3.1 飞机应满足如下一般要求：

a) 应具有稳定慢速、定高低空飞行能力，有准确可靠的导航定位系统和相应的续航力；

b) 有安装或吊放机载传感器和施放传感器的窗口。有与驾驶舱隔离而又能进行通话联系的调查工作用舱。

9.3.2 飞机至少应满足如下管理要求：

a) 每个机组需配备经过海上飞行训练并取得相应上岗资质证书、适应三种以上气象条件飞行的空勤人员及地勤人员。每次飞行前，应进行充分的地面准备，不能带故障与隐患起飞执行任务；

b) 应选用起降条件合适的机场。每次起降，应有良好、适宜的气象条件，预先获得准许，服从调度指挥；

c) 应制定严格的安全飞行措施。飞行中应随时保持与地面指挥部门的通讯联络，不准擅自飞离批准的调查飞行区域。

9.3.3 调查人员应遵守如下管理要求：

a) 各调查单位应按规定将调查计划和登机人员报飞行主管部门，办理有关登机手续；调查用的仪器设备、工具、材料和调查人员，上机前应由主管部门认真进行安全检查；

b) 调查人员应遵守机上工作的有关规定，不准进入驾驶舱；

c) 除地面指挥部门批准的调查项目的特殊需要外，在飞机起降过程中，应关闭调查仪器设备，如有需拖曳于机体以外的传感器，应于起飞后放出，降落前收回。

9.4 浮标和潜标

9.4.1 浮标和潜标应具备如下基本性能：

a) 应有牢固的浮体和系泊系统，应有可靠的应答、释放装置；

b) 应有在规定的布放时间内可靠地自动供电和自动采集、贮存和定时传输资料的能力；

c) 在布放海区极限海况条件下应能连续正常工作；

d) 应具有在规定时间内防生物附着能力和防腐蚀能力；

e) 应具有报告其工作状况和位置的能力；

f) 浮标应具有夜间灯光显示装置和雷达波反射装置。

9.4.2 浮标和潜标至少应满足如下管理要求：

a) 布放的位置（潜标还应固定在设计深度上）应使观测资料有良好的代表性，一般应避开航道和其他危险区，尽可能避免布放在渔船作业区；

b) 布放和回收浮标，应选择适宜的海况条件，由专人指挥，按规定程序实施；

c) 应经常监测浮标位置。当发现距岸不大于 300 km 的浮标移位超过 1.0 km，或距岸大于 300 km 的浮标移位超过 2.0 km 时，应立即记录移位后的实际位置，并将浮标重新拖回并系留到原位置上。移位超过以上规定距离后，观测到的资料，不能作为原位置资料使用；

d) 正常工作时应按期对浮标各部分进行检修或更换，出现故障应立即进行维修；

e) 浮标接收站应选建在有利于遥控指令发射和资料接收的位置上，应配备可靠的资料接收和处理设备，接收设备应配备有效的防雷击装置。

10 调查的导航定位

10.1 海洋环境基本要素调查的导航定位设备一般为全球定位系统（GPS）或差分全球定位系统（DGPS），GPS 应符合 GB/T 15527 的要求，DGPS 应符合 GB/T 17424 的要求。

10.2 调查船、飞机应配备能胜任的导航定位操作人员；并备有相应的图件和技术资料。

10.3 导航定位设备应按规定定期进行校准和性能测试，标定其系统参数。

10.4 GPS 或 DGPS 的安装、操作应按其使用说明书进行。

10.5 在海上调查开始前，应由导航定位人员将设计好的调查测线和测点画在导航定位图上或输入导航定位计算机内。

10.6 航海部门人员应在航海日志中准确记录与海洋调查有关的时间、站号、站位、航向、航速、水深等信息，并及时向调查人员提供航行参数和测线、测点的编号。在利用调查船进行漂泊调查时，应在进入、离开调查站位时分别报告一次时间与船位。条件允许时，应按首席科学家要求，提供尽可能加密测定的船位和船舶漂移轨迹。

10.7 返航后应及时将完整的导航定位资料和不确定度评价结果，提供给首席科学家。

10.8 导航定位准确度应符合调查项目的要求，推荐海洋地质地球物理调查（除采样活动外）定位准确度为 10 m，其他各专业、各要素为 50 m。

11 海上作业

11.1 时间标准

11.1.1 在领海、专属经济区和大陆架进行的海洋调查，应采用北京时间；远洋调查可采用北京时间或格林威治时间，并在资料载体上标明所采用的时间标准。

11.1.2 注意每 24 h 校对一次计时器，计时绝对误差不应超过 ±5 s（或相对误差不应超过 $\pm5.9\times10^{-5}$）。

11.2 观测作业要求

11.2.1 按 GB/T 12763.2～12763.6 和 GB/T 12763.8～12763.11 中相应条款的要求进行海洋要素观测、样品采集等海上作业，并按质量计划进行质量控制。

11.2.2 按 GB/T 12763.2～12763.6 和 GB/T 12763.8～12763.11 中相应条款要求分割、处理、包装、保存样品；并应及时进行必要的预处理和现场描述，准确地记录其状态并标识，填写有关记录表、簿。

11.2.3 现场描述的项目及其内容应简单并表格化，其内容应主要包括要素名称、调查海区、调查时间、测线和站位（观测点）层次、编号及样品状态等。标识应有利于实验室对样品的检查确认。

11.2.4 需马上分析测试的样品，应立即送船上实验室，并按 12.2～12.6 条的规定进行分析测试。

标准分享网 www.bzfxw.com 免费下载

11.2.5 值班人员应遵守值班和交接班制度，坚守岗位，认真负责。交接班时应将有关情况交接清楚。

11.2.6 应以学科为单位建立值班日志，值班日志应统一、规范，有确保填写记录内容真实的保障制度以及确保记录数据准确可靠的规定。值班日志由值班人填写，交接班时由接班人核验，学科负责人应定期检查，确保内容完整可靠。

11.2.7 值班日志内容主要包括：

a) 仪器安装调试及运行情况；

b) 站位作业情况，指时间、站位、人员、观测要素、作业深度、获取数据载体编号登记、采样登记、作业概况、质量偏离记录和处理措施(同时应及时将这些信息标注到样品和资料载体的标识上)；

c) 仪器设备故障、维修、更换记录；

d) 值班人员姓名；

e) 质量计划现场执行结果；

f) 事故与处理过程；

g) 调查中遇到的特殊海洋现象及处理情况等。

11.3 原始记录

11.3.1 按规定的期限记录、保存原始观测数据，以及调查现场状况、突发事件、异常现象、作业概况等信息。原始记录应以“共　页第　页”的形式标注页码，以空白表示无观测数据，以填划横杠表示漏测、缺测数据，以终结线表示其后无记录。观测、采样、测试的执行人员以及结果校核人员应签名。

11.3.2 记录应能长期保存。原始记录可以是磁载体、记录纸的自动记录，也可以是表簿的人工记录。人工记录除海上现场用黑色铅笔外，其他一律用蓝黑、黑色墨水笔填写；原始记录的数字或符号应填写在格的左下方，要求字迹清楚，字高约为表格高度的三分之二，不得伪造、涂擦；当记录出现错误时，应在原记录中间划一横线，在其右上方填写正确的数字或符号，并应有更正人员的签名或印章。

11.3.3 应考虑原始自动记录与人工记录间的一致性。

11.3.4 某项要素无法调查或因为仪器故障等影响调查结果质量时，应在相关的记录表的记录栏中注明，并在值班日志中详细说明；某项因故提前或延迟调查时，除注明原因外，应记录实际调查时间。

11.4 样品及观测记录的包装、存储和运输

样品、资料载体及采样记录应按有关的包装技术要求整理包装，标注清楚后应由负责人按规定的保管和运输要求进行保管、存储和运输。

11.5 安全措施

应制定具体明确的人身、仪器、资料的安全保障措施，建立安全岗位责任制和必要的奖惩制度。特别应注意规定在大风大浪、夜间、雷暴和雨雪等恶劣天气下工作及遇到特殊情况(如船舶碰撞、火灾、海啸等灾害)时采取的应急安全措施。

11.6 航次报告编写

航次结束后，各学科负责人负责整理本学科航次调查原始资料，审核签字后交首席科学家汇总；首席科学家在此基础上编写航次报告。

11.7 原始资料和样品的验收

11.7.1 在航次结束后十天内，由项目承担单位组织未参加本调查项目的三名以上同行专家，根据调查计划、GB/T 12763，组织对原始资料和样品的验收。

验收内容主要包括航次报告、记录到不同媒体上的调查数据和图件资料、导航定位资料、样品及其采集现场记录和描述、质量计划执行记录、调查值班日志和出海人员、仪器设备、标准物质的登记表。

11.7.2 数量不够、已变质、被污染、结构已破坏、标识不清、无状态描述的样品应作废；由不符合要求的调查人员、以不合格的仪器设备或标准物质、违反 GB/T 12763 或操作规程获取的资料为不合格资料；记录不清、观测不完整、数据丢失严重、载体破坏严重的资料及不具备溯源性的数据应为不合格资料；离散严重或观测准确度不能满足标准或质量计划对该要素要求的数据均为不合格数据。

11.7.3 验收不合格的样品和资料，不应计人有效工作量，不再进行样品分析鉴定和资料整理、计算。

11.7.4 不合格样品或资料超过三分之一的观测量为不合格观测量；主要观测量不合格或两个以上辅助观测量不合格为不合格要素；两个以上要素不合格的学科为不合格学科；主学科不合格或两个以上辅助学科不合格为不合格航次。

11.7.5 原始资料和样品的验收结果应作文字评语，由参加验收者签字、单位盖章，作为调查成果验收和资料归档内容之一。

11.7.6 航次报告及航次调查原始资料应提交调查项目负责人。

12 室内样品分析测试和鉴定

12.1 实验室资质

承担样品室内分析与测试的实验室应符合4.1.2的要求。

12.2 样品交接与记录

样品交接时应办理正式交接手续。实验人员在接收检验样品时，应记录其状态。包括是否异常或是否与相应的检验方法中所描述的标准状态有偏离。如果对样品是否适用于检验有任何疑问，或者样品与提供的说明不符，或者对要求的检验规定得不完全，实验人员应在工作开始之前要求送样者予以说明。样品交接时应办理正式交接手续。

12.3 样品的惟一性标识

实验室应建立对送检样品的惟一识别系统，以保证在任何时候对样品的识别不发生混淆。

12.4 样品的预处理和分析测试鉴定

实验室应按GB/T 12763.2～12763.6和GB/T 12763.8～12763.11中相关条款规定的方法和技术要求在规定的时间内完成样品的预处理、分析、测试和鉴定工作。

12.5 分析测试鉴定的质量检查

应在规定的时间内对分析、测试与鉴定结果按质量计划规定的要求进行质量检查。如发现误差超出规定范围，应重新分析、测试与鉴定。

质量检查措施为由质量保证人员安排的内控样、平行双样、盲样及实验室间互校等。

12.6 分析测试鉴定结果的发出

分析（测试、鉴定）结果应以规范的格式和内容表述，应由分析（测试、鉴定）者签名，经核验人核验、实验室负责人批准后及时发往送样单位。

12.7 剩余样品和标样的处置

剩余样品按相应专业的要求和项目质量计划的规定处置，并充分尊重委托方的意见。

13 资料处理

13.1 资料汇总

项目负责人负责将调查的现场作业与室内测试资料按船、航次汇总，并组织进行数据处理。

13.2 数据处理

13.2.1 按GB/T 12763.2～12763.6和GB/T 12763.8～12763.11中相关条款规定的方法和要求处理数据，发现并剔除坏值，修正系统误差，进行针对影响量的订正，整理、计算出各测量要素观测结果、导出量及内插值，推导测量要素分布函数。数据分析、计算应有责任制度，分析（计算）者、校核者应签字。

13.2.2 数据处理及导出量计算均应按《中华人民共和国法定计量单位使用方法》正确使用法定计量单位。

13.3 建立文档和图件绘制

13.3.1 根据调查获取的资料，按GB/T 12763.2～12763.6和GB/T 12763.8～12763.11中相关条款规定的格式建立数据文档。

标准分享网 www.bzfxw.com 免费下载

13.3.2 调查资料汇编一般是调查获取资料最终提交的压缩文档，其压缩程度应按项目委托单位的要求确定。

13.3.3 根据海洋调查资料汇编，按 GB/T 12763.2～12763.6 和 GB/T 12763.8～12763.11 中相关条款的规定来编绘海洋要素时空分布变化图件和声像资料，其种类、内容、编绘方法应按合同要求和海洋调查资料状况而定。

13.3.4 调查资料汇编、图件及声像资料上的数字、线条、符号应准确、清楚、端正、规格统一、注记完整、颜色鲜明。在图件和报表规定的位置上，应有有关人员的签名。

13.3.5 绘制的图件、声像资料应由相应水平的科技人员进行检查，由不低于编制者水平的其他科技人员进行复核，并对不恰当的地方进行必要的修改并签字。

13.3.6 调查资料汇编应附数据转换(或反演)、发现并剔除坏值、系统误差修正、对影响量的订正、导出量及内插值计算、测量要素分布函数推导及相关量比较等方法的说明，附以对各要素的数据进行准确度及离散性检查的结果。

13.4 计算机处理资料的要求

13.4.1 用于数据处理工作的计算机，应具有满足工作要求的环境、配套设施、硬件配置和相应工作软件。应建立必要的规章制度，以保证其正常、安全地工作，不受无关人员、信号及自然现象的干扰。

13.4.2 计算机工作软件应是正版合法产品；委托或自行开发的工作软件则应经鉴定合格方可使用；全部工作软件应经调查项目承担单位批准。

13.4.3 由同岗人员定期地认真检查输入参数和软件系统；推导反演计算数据时，应首先由第二人对计算公式、方法、步骤和使用数据进行严格审查与核算，再用其他独立计算工具对计算结果抽查核验 5% 的数据；计算、审查和抽查核验均应有责任人签字。

13.4.4 输入计算机或录入报表上的数据，应经第二人校对，应保证误码率低于 1×10^{-4}。

13.4.5 记录调查资料的电子媒体原件应存档，用其复制品进行资料的整理。

14 调查报告编写

14.1 编写内容

14.1.1 前言

前言应至少包括如下内容：

a) 任务及其来源，重点论述应突出；
b) 调查海区的位置及地理坐标；
c) 调查船(飞机)及调查时间；
d) 调查方法；
e) 任务执行概况；
f) 项目组成员、调查队成员、质管人员及其分工。

14.1.2 调查海区

调查海区应至少包括如下内容：

a) 调查海区及周围地区的自然环境；
b) 以前对该海区的调查研究程度。

14.1.3 海上调查工作状况

海上调查工作状况应至少包括如下内容：

a) 测网、测线及测点的布设；
b) 导航定位系统及准确度评价；
c) 仪器设备的性能和运转情况；
d) 调查方法和现场资料描述。

14.1.4 **样品分析和资料整理**

样品分析和资料整理应至少包括如下内容：

a) 原始资料和样品的周转与审核；

b) 样品分析、测试、鉴定方法及概况；

c) 资料整理、处理、计算和图件编绘方法及概况；

d) 调查要素时空分布特征。

14.1.5 **质量计划实施情况报告**

质量计划实施情况报告应至少包括如下内容：

a) 资源配置的合理性、符合性和量值溯源的实现程度；

b) 调查数据处理中统计方法的说明；

c) 本单位及分包单位的质量控制措施实施情况与结论；

d) 参考资料的溯源性和合理性；

e) 样品、原始资料、资料汇编和图集的质量评价；

f) 调查结果的质量及应用价值评估；

g) 质量目标实现状况。

14.1.6 **资料分析与结论**

14.1.6.1 分析解释调查资料和图件时，应阐明调查资料时空分布特征、重要发现，并评价其海洋学意义及其与海洋管理、工程设计、开发利用的关系。

14.1.6.2 应将观测到的数据进行统计分析，并与历史资料、临近水域（站位）资料，相关学科等资料进行比较和综合分析，然后确立分布规律，发现演变规律及相关量间的经验公式，从中发现并解释异常值和异常规律。

14.1.6.3 根据14.1.6.2的统计分析结果，对调查海域的水文、气象、地质、化学、生物等环境特性进行评估，就委托方提出的调查目的给出客观、科学、公正的评估结论。

14.1.7 **存在问题和建议**

应总结调查发现的问题，并提出今后应开展工作的建议。

14.2 **编写要求**

14.2.1 应在对已有文献、资料和本次调查资料、图件进行深入分析、研究的基础上编写。

14.2.2 应按任务书或合同书、调查计划和GB/T 12763的有关规定编写。

14.2.3 应重点分析、研究本次调查获得的资料和图件，同时充分利用调查海区及周围地区内已有的资料和文献。

14.2.4 应重点论述样品的分析、测试和资料整理、计算，资料、图件的分析和解释。

14.2.5 力求内容全面、重点突出、论据充分、文字精练。

14.2.6 应有必要的附图和插图。

14.2.7 按调查计划规定的时限完成调查报告的编写。

15 调查资料和成果归档

15.1 归档范围

调查项目负责人应确保每一项调查归档文件材料的完整、准确和系统。归档范围主要包括：

a) 任务合同书（委托书、协议书）、有关的请示报告、批复、汇报及重要信函等；

b) 海洋调查计划、技术设计及其实施记录；

c) 各种载体的重要原始记录、原始资料、实验室分析测试报告、图纸；

d) 航次报告、阶段性调查成果及其验收记录；

e) 调查报告最终原稿及印刷稿；

标准分享网 www.bzfxw.com 免费下载

f) 技术鉴定材料或验证资料；

g) 成果鉴定和验收证书和文件(待鉴定、验收后归档)；

h) 经费结算报告；

i) 符合 GB/T 12763.7 规定的调查元数据。

15.2 归档要求

15.2.1 从项目立项开始，由项目负责人负责组织对海洋调查研究过程中所形成的有关文件材料，按 HY/T 058 的规定系统整理立卷。归档资料由项目负责人审核签字，经调查项目承担单位档案管理部门审查，确认符合 HY/T 058 的相应规定后验收归档。

15.2.2 归档应按 HY/T 058 规定的程序和要求办理归档手续。归档的文件、制作材料应优良，应格式统一、字迹工整、图样清晰、装订牢固、签字手续完备。

15.2.3 调查资料应按保密规定划分密级，妥善保管。调查实施过程中所形成的重要管理文件、材料、海洋调查的调查计划、原始记录、原始资料、资料汇编、图集图件、调查报告、规范性作业文件、追溯性记录等均应永久保管；其他文件材料可定为长期或短期保管。

15.2.4 归档和移交的有关文件材料应是原件。

15.2.5 以磁带、磁盘等不能长期保存的载体归档的资料，应按载体特性及时转录为光盘或(另一种载体)，并在防磁、防潮和适宜温度条件下保存。

15.2.6 电子文件材料归档应注明技术环境条件、相关软件版本、数据类型格式、操作数据、检测数据及备份要求等。

15.2.7 分包单位和受委托承担样品实验室分析、测试单位的原始记录和数据处理记录一般由该单位归档，其分包调查报告和分析、测试报告作为项目承担单位的调查原始记录归档。

15.3 归档时间

持续时间为 2 年以内的调查项目，于验收或鉴定前、后两次完成归档；持续时间为 2 年以上的调查项目，还应在每个航次结束后六个月内归档一次。

15.4 档案质量要求

海洋调查档案质量应符合 HY/T 058 的有关规定，与 5.2 条规定相应的质量保证或证明文件齐备。归档不符合要求的项目，不应进行成果验收。

16 成果鉴定和验收

16.1 成果鉴定

16.1.1 调查成果鉴定对象应包括调查报告、成果图件(或图集)和资料报表(或资料汇编)等。

16.1.2 鉴定依据是项目合同书(委托书、协议书)、GB/T 12763 和调查计划。

16.1.3 鉴定应在档案管理部门出具调查成果完成归档的证明后，及时按《科学技术成果鉴定办法》进行。鉴定通过时，应按规定填写科技成果鉴定证书；鉴定不通过时，限期补充修改，再次报请重新鉴定。

16.2 成果验收

16.2.1 验收内容主要是对调查进行情况和调查成果进行验收。

16.2.2 应依据项目合同书(委托书、协议书)、调查计划、GB/T 12763、原始资料验收结论、归档证明和成果鉴定证书进行验收。电子媒体的海洋调查资料汇编在验收时应按 GB/T 12763.7 中的有关规定进行检验。

16.2.3 应由调查项目委托单位派人组织验收，确认合格后形成由验收人签字和验收双方单位盖章的书面验收结论，并由双方归档。与验收依据有明显差距的成果不予验收，责令限期修改、提高，并重新组织验收。

16.2.4 通过验收的调查成果各独立文本，应经调查项目负责人、调查项目承担单位授权的签字人签字，正本交项目委托单位，副本留承担单位归档。其他资料的移交内容和形式根据合同书或验收协议确定。

附 录 A
（规范性附录）
航 次 报 告

A.1 航次报告由航次概况、主要调查人员岗位、、观测与取样概况、航线与站位图、海区地理特征、质量计划实施情况等部分构成。

A.2 航次概况格式见表 A.1。

A.3 主要调查人员指本航次负责各不同调查学科、要素的调查人员，其岗位表格式见表 A.2。

A.4 浮标潜标漂流浮标等观测系统布放/回收概况表格式见表 A.3。

A.5 观测与取样概况表格式见表 A.4。

A.6 填入航线与站位图，并附上测线、测点和站位的必要说明，格式如表 A.5。

A.7 海区地理和环境特征应填写本航次调查的洋区/海域名称，地理坐标，具体调查海域内对调查结果的准确度或代表性有影响的流场、水质、底质、地形、地貌，工程设施、航道、养殖区、排污口及沉船等人为设施。格式见表 A.6。

A.8 质量计划实施情况应填写质量计划规定的质量控制措施的执行情况，质量目标达标情况，及可能的调查资料质量验证情况，格式自定。

标准分享网 www.bzfxw.com 免费下载

表 A.1 航次概况

航次报告		承担单位资料室填写<br>保管部门______查阅号______			
		资料交换限制	可	否	部分
是否属出具社会公证数据					

填入获取资料的调查船(或观测平台)的名称及国际无线电呼号,并注明调查船(或平台)类型

调查船/观测平台名称______呼号______

调查船/观测平台类型______

填入本航次编设的航次号、名称或缩写。(可能的话,注明测线)

航次号/名称______

调查日期　从___年___月___日___至___年___月___日

始发港(填入国家和港口名称)______

抵达港(填入国家和港口名称)______

承担单位

名称______资质证书号______

国家______地址______邮政编码______

首席科学家

姓　名______职称______职务______

所在单位______

航次目的概述:填入本航次有关调查目的和特征的信息

______

______

______

______

______

______

______

项目委托单位______合同编号______

计划名称______计划编号______

协作机构______;______;______

表 A.2 主要调查人员岗位

姓 名	工 作 单 位	负责学科	负责要素	证书号码
注：视主要人员数量调整表中的行数。				

表 A.3 浮标、潜标、漂流浮标布放/回收概况

		站 位							
站位概况	站位号	纬 度			经 度			布放/回收日期，时间	站位标识
		(°)	(′)	N/S	(°)	(′)	E/W		
主要调查人员	姓 名	职 责		值班时间		姓 名		职 责	值班时间
仪器设备传感器使用概况	名称	型号		出厂编号		布放深度		设定值	工作状况
资料登记	资料名称	载体编号		位置		数据量		记录者	核验者
注：视各站的具体信息状况，调整该站该项信息的行数。									

标准分享网 www.bzfxw.com 免费下载

表 A.4 观测与取样概况

站位号	资料/样品概况				测量仪器/采样设备概况			调查人员	备注
	名称	数据量<br>数 量	载体编号<br>包装编号	样品有<br>效期	仪器设<br>备名称	型号	出厂<br>编号		

统计资料	光盘	软盘	磁带	自记纸	记录表	记录簿	照片	传真	胶片	其他

统计样品	盐度	溶解氧	营养盐	有机碳	叶绿素	pH	悬浮物	浮游生物	游泳生物	底栖生物	污损生物	沉积物	岩芯	其他

注：视各站的具体信息状况，调整该站该项信息的行数。

表 A.5 航线与站位图

航线与站位图：	如提供航行记录，则在方框内划"√" □<br>测线、测点和站位的必要说明：

表 A.6 海区地理和环境特征

调查海区名称：
调查海区地理和环境特征：

标准分享网 www.bzfxw.com 免费下载

附 录 B
（规范性附录）
海洋调查人员登记表

海洋调查人员登记表的格式见表B.1。

表 B.1 海洋调查人员登记表

序号	姓名	单 位	证书号码	持证项目	出海岗位	使用仪器设备	审核意见

填报单位： 填报人： 填报日期： 年 月 日

审核单位： 审核人： 审核日期： 年 月 日

附 录 C
（规范性附录）
海洋调查仪器设备检查登记表

海洋调查仪器设备检查登记表的格式见表 C.1。

表 C.1 海洋调查仪器设备检查登记表

序号	仪器名称	型号	出厂编号	检定(校准)证书编号	检定(校准)证书有效期限	检查调试情况	检查者签名	复核意见

填报单位： 填报人： 填报日期： 年 月 日

审核单位： 审核人： 审核日期： 年 月 日

标准分享网 www.bzfxw.com 免费下载

附 录 D
（规范性附录）
标准物质及化学试剂检查登记表

标准物质及化学试剂检查登记表的格式见表D.1。

表 D.1 标准物质及化学试剂检查登记表

序号	标准物质（或化学试剂）名称	生产单位	生产许可证编号	批号	有效期限	检查结果	检查者签名	复核意见

填报单位：　　填报人：　　填报日期：　年　月　日

审核单位：　　审核人：　　审核日期：　年　月　日
