# Supplementary material for: Autumn ichthyoplankton assemblage in the Yangtze Estuary shaped by environmental factors
Source: PeerJ. 2016 Apr 19;4:e1922. doi: 10.7717/peerj.1922 (PMC4841249; doi:10.7717/peerj.1922)
Supplement: Supplemental Information 1 [file peerj-04-1922-s001.zip › GB12763-2007/GB¿MT 12763.2-2007 ║ú╤≤╡≈▓Θ╣μ╖╢ ╡┌02▓┐╖╓ú║║ú╤≤╦«╬─╣█▓Γ.pdf]

ICS 07.060
A 45

中华人民共和国国家标准

GB/T 12763.2—2007
代替 GB/T 12763.2—1991

海洋调查规范
第2部分:海洋水文观测

Specifications for oceanographic survey—
Part 2:Marine hydrographic observation

2007-08-13 发布 2008-02-01 实施

中华人民共和国国家质量监督检验检疫总局
中国国家标准化管理委员会 发布

目　　次

前言 …… Ⅲ
1　范围 …… 1
2　规范性引用文件 …… 1
3　术语和定义 …… 1
4　一般规定 …… 3
4.1　技术设计 …… 3
4.2　观测要素、方式及顺序 …… 3
4.3　测站布设原则及观测间隔选取 …… 4
4.4　测站定位和观测时间标准 …… 4
4.5　水文观测仪器和设备的基本要求 …… 4
4.6　质量控制管理 …… 4
4.7　观测资料记录、整理和交换，以及调查成果的验收 …… 4
4.8　水深测量 …… 4
5　水温观测 …… 5
5.1　技术指标 …… 5
5.2　观测方法 …… 5
5.3　资料处理 …… 7
6　盐度测量 …… 7
6.1　技术指标 …… 7
6.2　观测方法 …… 7
6.3　资料处理 …… 8
7　海流观测 …… 8
7.1　技术指标 …… 8
7.2　观测方法 …… 8
7.3　资料处理 …… 11
8　海浪观测 …… 11
8.1　技术指标 …… 11
8.2　观测方法 …… 12
8.3　资料处理 …… 13
9　水位观测 …… 14
9.1　技术指标 …… 14
9.2　观测方法 …… 14
9.3　资料处理 …… 15
10　海水透明度、水色和海发光观测 …… 15
10.1　技术指标 …… 15
10.2　观测方法 …… 15
11　海冰观测 …… 16
11.1　技术指标 …… 16

11.2 观测方法 …… 17
11.3 资料处理 …… 19
附录A(规范性附录) 实验室盐度计测量海水样品盐度和计算盐度的有关公式 …… 20
附录B(规范性附录) 利用颠倒温度表观测海水温度 …… 23
附录C(规范性附录) 海洋水文观测用表 …… 26
附录D(资料性附录) 海洋水文观测记录表格式 …… 30

图1 锚碇浅水应用型潜标 …… 10
图2 锚碇深水应用型潜标 …… 10
图3 锚碇明标系统的组成 …… 10
图4 波面随时间的变化曲线 …… 14

表1 水温观测的准确度和分辨率 …… 5
表2 标准观测层次 …… 5
表3 盐度测量的准确度和分辨率 …… 7
表4 海流观测的准确度 …… 8
表5 海况等级表 …… 12
表6 波型分类表 …… 12
表7 海冰观测要素的单位和准确度 …… 16
表8 浮冰漂流速度的目测估计 …… 18
表B.1 颠倒温度表系列主要技术指标 …… 23
表C.1 波级表 …… 26
表C.2 十六个方位与度数换算表 …… 26
表C.3 海发光类型及强度等级表 …… 27
表C.4 浮冰冰型表 …… 27
表C.5 浮冰表面特征分类表 …… 28
表C.6 浮冰冰状表 …… 28
表C.7 固定冰冰型表 …… 28
表C.8 冰山等级表 …… 29
表C.9 冰山形状分类表 …… 29
表D.1 目测海浪记录表 …… 30
表D.2 海水透明度、水色和海发光观测记录表 …… 30
表D.3 浮冰观测记录表 …… 31
表D.4 固定冰观测记录表 …… 31
表D.5 冰山观测记录表 …… 32
表D.6.1 颠倒温度表测温记录表(Ⅰ) …… 32
表D.6.2 颠倒温度表测温记录表(Ⅱ) …… 33
表D.7 盐度分析记录表 …… 33
表D.8 CTD观测记录表 …… 34
表D.9 漂流浮标记录表 …… 34
表D.10 船只锚碇测流记录表 …… 35
表D.11 声学多普勒测流记录表 …… 35

前　言

GB/T 12763《海洋调查规范》分为11个部分：

——第1部分：总则；
——第2部分：海洋水文观测；
——第3部分：海洋气象观测；
——第4部分：海洋化学要素调查；
——第5部分：海洋声、光要素调查；
——第6部分：海洋生物调查；
——第7部分：海洋调查资料交换；
——第8部分：海洋地质地球物理调查；
——第9部分：海洋生态调查指南；
——第10部分：海底地形地貌调查；
——第11部分：海洋工程地质调查。

其中第9部分、第10部分和第11部分对应于GB/T 12763—1991是新增部分。

本部分为GB/T 12763的第2部分，代替GB/T 12763.2—1991《海洋调查规范　海洋水文观测》。

本部分与GB/T 12763的第1部分和GB/T 12763的第7部分配套使用。

本部分与GB/T 12763.2—1991相比主要变化如下：

——引用标准中增加了GB/T 15920—1995《海洋学术语：物理海洋学》和GB/T 14914—1994《海滨观测规范》(见第2章)；
——术语和定义全部归入第3章(见第3章)；
——在“一般规定”中，补充了“技术设计”的内容，并明确规定技术设计“应形成文件，并报主管部门审批”(1991版第一篇中的3；本版的4.1)；增加了质量控制管理的规定，以适应新建立的质量管理体系(即ISO 9000)的要求(本版的4.6)；同时，将“观测资料记录、整理和验收的一般要求”改为“观测资料记录、整理和交换，以及调查成果的验收”(1991版第一篇的8；本版的4.7)；
——删掉“深度测量”篇，水深测量仅在“一般规定”中简单提及(1991版第二篇；本版4.8)；
——对1991版的“水温观测”做了较重大的修改：
  a) 通过引导语，对水温观测准确度做了更灵活、更符合实际的规定(1991版的14.1；本版的5.1.1)；
  b) 将连续测站水温观测时次由“一般每两小时观测一次”改为“一般每小时观测一次”(1991版的14.2；本版的5.1.2)；
  c) 将“水温观测的标准层次”的内容全部改用表格表示，并将表层改为“海面下3 m以内的水层”(1991版的14.3；本版的5.1.3)；
  d) 本版摒弃了1991版有关水温观测方法和观测记录整理的叙述方式，突出了已普遍使用的现代化温盐深仪(即CTD)观测水温的方法和技术，以及资料处理方法(1991版的15和16；本版的5.2.1和5.3.1)；同时，增加了走航测温的内容(本版的5.2.2和5.3.2)；
  e) 在“利用颠倒温度表观测水温”中，增加了“仪器设备和主要技术指标”内容；操作使用方法按“使用前检查”与“安装和测量”两部分重新做了规范(1991版的附录A；本版附录B)；
——与“水温观测”相对应，“盐度测量”中重点规范了CTD仪测量盐度的方法(1991版的19.1；本版6.2.1和6.3.1)；增加了走航测量盐度的内容(本版的6.2.2和6.3.2)；同时，将1991版中

"利用实验室盐度计测量海水样品盐度"以及"实验记录的整理"的部分内容皆从正文移入附录(1991 版的 19.2、20.1.2 和 20.2;本版的附录 A);

——大幅度地改写了"海流观测"一章:

a) 将海流"测量的准确度"的内容全部改用表格表示,并通过引导语对测量的准确度提出了更高的要求(1991 版的 22.2;本版的 7.1.2);

a) 增加了海表面漂移浮标测流、锚定测流和走航测流的观测方法和观测记录整理(本版的 7.2.1,7.2.3,7.2.4 和 7.2.5);

b) 按"漂流浮标测流"、"船只锚定测流"、"锚定潜标测流"、"锚定明标测流"和"走航测流"五大系列重新规定了海流观测方法和观测记录的整理(1991 版的 23 和 24;本版的 7.2 和 7.3);

——"波浪观测"作了以下两方面的修改:

a) 对波高、周期和波向测量的准确度作了更具广泛性的规定(1991 版的 26.2;本版的 8.1.2);

b) 本版摒弃了过去按观测要素撰写观测方法,而将观测方法按"目测"和"仪器观测"进行规范。同时,扩充了仪器观测方法内容,增加了"以船只为承载工具测波和锚定测波"的观测方法(1991 年版的 27;本版的 8.2);

——水位观测从附录移入正文,并重写了水位观测方法(1991 年版附录 D;本版的第 9 章);

——对"海冰观测"作了以下几方面的修改:

a) 在"技术指标"中增加了"观测要素的单位与准确度"(本版的 11.1.2);

b) 对漂流方向和速度,以及海冰厚度的观测方法作了部分修改(1991 年版的 34.2.6,34.2.7;本版的 11.2.2.6,11.2.2.7);

c) 增加了固定冰厚度观测的内容(本版的 11.2.3.2);

——对"水文观测用表"作了以下的删补和修改:

a) 删掉了有关"深度测量"的有关用表(1991 年版附录 C 中的表 C1 和表 C2);

b) "波级表"的内容做了补充,并增加了"十六个方位与度数换算表"(1991 年附录 B 中的表 B3;本版附录 C 中的表 C.1、表 C.2);

c) "海冰用表"按新的格式做了调整(1991 年附录 B 中的表 B5～表 B8;本版附录 C 中的表 C.4～表 C.7);

d) 与修改后的水温、海流观测方法相对应,在附录 D 中增加了表 D.8、表 D.9、表 D.10 和表 D.11。

本部分的附录 A、附录 B、附录 C 为规范性附录,附录 D 为资料性附录。

本部分由国家海洋局提出。

本部分由国家海洋标准计量中心归口。

本部分由国家海洋局第一海洋研究所负责起草,国家海洋局第二海洋研究所、北海分局参加起草。

本部分主要起草人:汤毓祥、孙洪亮、胡筱敏、娇玉田、熊学军、梁楚进、王炜阳。

本部分所代替标准的历次版本发布情况为:

——GB/T 12763.2—1991。

海洋调查规范
第2部分:海洋水文观测

1 范围

GB/T 12763的本部分规定了海洋水文观测的基本要素、技术指标、观测方法和资料处理。

本部分适用于海洋环境基本要素调查中的海洋水文观测。

2 规范性引用文件

下列文件中的条款通过GB/T 12763本部分的引用而成为本部分的条款。凡是注日期的引用文件,其随后所有的修改单(不包括勘误的内容)或修订版均不适用于本部分。然而,鼓励根据本部分达成协议的各方研究是否可使用这些文件的最新版本。凡是不注日期的引用文件,其最新版本适用于本部分。

GB/T 12763.1 海洋调查规范 第1部分:总则

GB/T 12763.3 海洋调查规范 第3部分:海洋气象观测

GB/T 12763.7 海洋调查规范 第7部分:海洋调查资料交换

3 术语和定义

下列术语和定义适用于GB/T 12763的本部分。

3.1

现场水深 in site water depth

现场测得的自海面至海底的垂直距离。测量的目的主要用于确定测站的深度。

3.2

仪器沉放深度 deployed depth of instrument

自海面至水下观测仪器的垂直距离。用于确定所测得的水文要素值所在深度。

3.3

水温 water temperature

现场条件下测得的海水温度。

注:单位为℃。

3.4

盐度 salinity

海水中含盐量的一个标度。

注:绝对盐度和实用盐度的定义见GB/T 15920—1995:海洋学术语 物理海洋学。实用盐度的计算式见附录A。

3.5

海流 ocean current

海水的宏观流动,以流速和流向表征。

注1:流速单位为cm/s。

注2:流向指海水流去的方向。单位为度(°),正北为零,顺时针计量。

3.6

海浪 ocean wave

海洋中由风产生的波浪。包括风浪及其演变而成的涌浪。

注1:风浪和涌浪的定义见GB/T 15920—1995。

注2:海浪的基本要素包括波高、波向和周期等,其定义见GB/T 15920—1995。

3.7

海况　sea condition

在风力作用下的海面特征。

3.8

波型　wave pattern

海浪的外貌特征。

3.9

水位　water level

观测点处海面相对于某参照面的垂直距离。

注：计量单位为 m。

3.10

海水透明度　sea water transparency

表征海洋水体透明程度的物理量，表征光在海水中的衰减程度。

注：计量单位为 m。

3.11

水色　water colour

位于透明度值一半的深度处，白色透明度盘上所显示的海水颜色。

注：用水色计的色级号码表示。

3.12

海发光　luminescence of the sea

夜间海面上出现的生物发光现象。

3.13

海冰 sea ice

在海上所见到的由海水冻结而成的冰。广义的是指海洋上一切冰的总称。

注：浮冰、固定冰和冰山的定义见 GB/T 15920—1995。

3.14

海洋观测　sea observation

在海上观察和测量海洋环境要素的过程。

3.15

大面观测　extensive observation

在调查海区布设的若干观测点上，船到站即测即走的观测。

3.16

断面观测　sectional observation

在调查海区一水平直线上设计多个观测点，由这些观测点的垂线所构成的面称为断面。在此断面之站点上进行的海洋观测称为断面观测。

3.17

连续观测　continuosly observation

在调查海区有代表性的测点上，连续进行 25 h 以上的海洋观测。

3.18

同步观测　synchronous survey

在调查海区若干站点上，同时进行相同海洋环境要素的观测。

3.19

走航观测　running　observation

根据预先设计的航线，使用单船或多船携带走航式传感器采集观测要素数据。

3.20

CTD conductivity-temperature-depth

温盐深仪，用于测量深度以及温度和盐度垂直连续变化的自记仪器。

3.21

XBT expendable bathythermography

抛弃式温深仪，一种在船只以规定船速航行下投放，用于测量温度、深度的仪器。

3.22

XCTD expendable conductivity-temperature-depth

抛弃式温盐深仪，一种在船只以规定船速航行下投放，用于测量温度、盐度和深度的仪器。

3.23

ADCP acoustic doppler current profiler

声学多普勒海流剖面仪，以声波在流动液体中的多普勒频移来测量流速的仪器。

4 一般规定

4.1 技术设计

4.1.1 技术设计的内容

接收调查项目后，承担单位应根据任务书或合同书的要求进行技术设计，内容主要包括：

a) 调查海区范围与测站布设；

b) 观测要素与观测层次；

c) 观测方式与时次；

d) 调查船及其主要设备的要求；

e) 主要观测仪器的名称、型号及数量；

f) 人员的组织和分工；

g) 观测资料的分析方法；

h) 质量要求与质量控制要点；

i) 应提交的调查成果、完成时间及验收方式；

j) 其他。

4.1.2 技术设计的形成

技术设计由项目负责人组织编制，应形成文件，并报主管部门审批。

4.2 观测要素、方式及顺序

4.2.1 观测要素

海洋水文观测要素一般包括：水温、盐度、海流、海浪、透明度、水色、海发光和海冰等。如有需要，还要观测水位。每次调查的具体观测要素，据任务书或合同书的要求而定，并应在技术设计文件中明确规定。

4.2.2 观测方式

依据调查任务的要求与客观条件的允许程度，水文观测方式可选择下列中的一种或多种：

a) 大面观测；

b) 断面观测；

c) 连续观测；

d) 同步观测；

e) 走航观测。

4.2.3 观测顺序

水文观测一般按下列顺序进行：

a) 观测前准备和检查仪器；

b) 对于大面(或断面)观测，到站后首先测量水深；对于连续观测应在正点前测量水深；

c) 观测水温、盐度，并采水；

d) 观测海流，对于连续观测站，海流观测应尽可能在正点完成；

e) 观测海浪、透明度、水色和海发光；

f) 观测海冰。

4.3 测站布设原则及观测间隔选取

测站的布设和观测间隔的选取应符合以下原则：

a) 布设的测站在观测海区应具有代表性，使所测得的水文要素数据能够反映该要素的分布特征和变化规律。

b) 每一水文断面应不少于三个测站。同一断面上各测站的观测工作应在尽可能短的时间内完成。

c) 相邻两测站的站距，应不大于所研究海洋过程空间尺度的一半；在所研究海洋过程的时间尺度内，每一测站的观测次数应不少于两次。如条件允许，应尽量缩小时、空观测间隔。

4.4 测站定位和观测时间标准

测站的定位和观测的时间标准按 GB/T 12763.1 的有关规定执行。

4.5 水文观测仪器和设备的基本要求

水文观测仪器和设备应符合 GB/T 12763.1 的有关规定。同时，还应满足以下要求：

a) 仪器的适用水深范围和测量范围应满足观测水深和所测要素的变化范围，同时还须满足对观测要素及其计算参数的准确度及时空连续性的要求。

b) 选用的仪器应适于所采用的承载工具和观测方式。

c) 调查设备安装位置的基本要求是：工作方便，各项工作互不妨碍，防止建筑物、辐射热和船只排出污水等对观测结果的影响。

d) 每航次观测结束后，调查设备和观测仪器应认真维护保养。凡入水的仪器均须用淡水洗净晾干后保存。绞车和钢丝绳等应仔细擦拭，并进行保养。

4.6 质量控制管理

海洋水文观测质量控制管理的详细内容见 GB/T 12763.1 的有关规定。

4.7 观测资料记录、整理和交换，以及调查成果的验收

按以下有关规定实施：

a) 对原始观测资料记录和验收按 GB/T 12763.1 的有关规定实施。

b) 观测资料的记录格式和交换内容按 GB/T 12763.7 的有关规定执行。

c) 海洋调查报告的编写按 GB/T 12763.1 的有关规定实施。

d) 调查成果的鉴定和验收按 GB/T 12763.1 的有关规定实施。

4.8 水深测量

包括现场水深和仪器沉放深度的测量：

a) 水深以米(m)为单位。记录取一位小数，准确度为±2%。

b) 大面或断面测站，船到站测量一次；连续测站，每小时测量一次。

c) 现场水深测量采用回声测深仪。如条件不具备或水深较浅，可采用钢丝绳测深法。

d) 钢丝绳测深时若钢丝绳倾斜，应用偏角器量取钢丝绳倾角。倾角超过 10°时，应进行钢丝绳的倾角订正。倾角较大时，应加大铅锤重量或利用其他方法使倾角尽量控制在 30°以内。

e) 仪器沉放深度，通常由仪器本身所配压力传感器测得。但当仪器本身未装配压力传感器时，可参照钢丝绳测现场水深的方法进行测量。

5 水温观测

5.1 技术指标

5.1.1 水温观测的准确度

主要根据项目的要求和研究目的，同时兼顾观测海区和观测方法的不同以及仪器的类型，按表1确定水温观测的准确度。

表1 水温观测的准确度和分辨率

准确度等级	准确度/<br>℃	分辨率/<br>℃
1	±0.02	0.005
2	±0.05	0.01
3	±0.2	0.05

5.1.2 观测时次

大面或断面测站，船到站观测一次；连续测站，一般每小时观测一次。

5.1.3 水温观测的标准层次

标准观测层次如表2所示。

表2 标准观测层次 单位为米

水深范围	标准观测水层	底层与相邻标准层的最小距离
<50	表层，5，10，15，20，25，30，底层	2
50～100	表层，5，10，15，20，25，30，50，75，底层	5
100～200	表层，5，10，15，20，25，30，50，75，100，125，150，底层	10
>200	表层，10，20，30，50，75，100，125，150，200，250，300，400，500，600，700，800，1 000，1 200，1 500，2 000，2 500，3 000（水深大于3 000 m时，每千米加一层），底层	25

注1：表层指海面下3 m以内的水层。

注2：底层的规定如下：水深不足50 m时，底层为离底2 m的水层；水深在50 m～200 m范围内时，底层离底的距离为水深的4%；水深超过200 m时，底层离底的距离，根据水深测量误差、海浪状况、船只漂移情况和海底地形特征综合考虑，在保证仪器不触底的原则下尽量靠近海底。

注3：底层与相邻标准层的距离小于规定的最小距离时，可免测接近底层的标准层。

5.2 观测方法

5.2.1 温盐深仪（CTD）定点测温

5.2.1.1 仪器设备

CTD仪分实时显示和自容式两大类。

5.2.1.2 观测步骤和要求

CTD仪操作主要包括室内和室外操作两大部分。前者主要是控制作业进程，后者则是收放水下单元，但两者应密切配合、协调进行。具体观测步骤和要求如下：

a) 观测期间首先应按表D.8的格式记录有关信息；并在计算机中输入观测日期、文件名、站位（经度、纬度）和其他有关的工作参数。

b) 投放仪器前应确认机械连接牢固可靠，水下单元和采水器水密情况良好。待整机调试至正常工作状态后开始投放仪器。

c) 将水下单元吊放至海面以下，使传感器浸入水中感温 3 min～5 min。对于实时显示 CTD，观测前应记下探头在水面时的深度（或压强值）；对自容式 CTD，应根据取样间隔确认在水面已记录了至少三组数据后方可下降进行观测。

d) 根据现场水深和所使用的仪器型号确定探头的下放速度。一般应控制在 1.0 m/s 左右。在深海季节温跃层以下下降速度可稍快些，但以不超过 1.5 m/s 为宜。在一次观测中，仪器下放速度应保持稳定。若船只摇摆剧烈，可适当增加下放速度，以避免在观测数据中出现较多的深度（或压强）逆变。

e) 为保证测量数据的质量，取仪器下放时获取的数据为正式测量值，仪器上升时获取的数据作为水温数据处理时的参考值。

f) 获取的记录，如磁盘、记录板和存储器等，应立即读取或查看。如发现缺测数据、异常数据、记录曲线间断或不清晰时，应立即补测。如确认测温数据失真，应检查探头的测温系统，找出原因，排除故障。

g) CTD 仪测温注意事项：

1) 释放仪器应在迎风舷，避免仪器压入船底。观测位置应避开机舱排污口及其他污染源。

2) 探头出入水时应特别注意防止和船体碰撞。在浅水站作业时，还应防止仪器触底。

3) 利用 CTD 测水温时，每天至少应选择一个比较均匀的水层与颠倒温度表的测量结果比对一次。如发现 CTD 的测量结果达不到所要求的准确度，应及时检查仪器，必要时更换仪器传感器，并应将比对和现场标定的详细情况记入观测值班日志。

4) CTD 的传感器应保持清洁。每次观测完毕，须冲洗干净，不能残留盐粒和污物。探头应放置在阴凉处，切忌曝晒。

5.2.2 走航测温

5.2.2.1 仪器设备

抛弃式温深仪（XBT）、抛弃式温盐深仪（XCTD）和走航式 CTD（MVP300）等皆可按观测要求，在船只以规定船速航行下投放。

5.2.2.2 观测步骤和要求

使用 XBT、XCTD 和 MVP300 等仪器走航测温的基本步骤和要求如下：

a) XBT 和 XCTD 观测：

1) 仪器探头投放前，输入探头编号、型号、时间、站号、经纬度，并进入投放准备状态。

2) 应用手持发射枪或固定发射架（要求良好接地），将探头投入水中。带有仪器控制器的专用计算机便开始显示采集数据或绘制曲线。

3) 探头的投放，最好选在船体后部进行，以免导线与船舷磨擦。

b) 走航式 CTD（MVP300）观测：

1) 绞车系统自检、数据采集及通讯软件自检、GPS 数据检测。

2) 按观测要求，船只以规定船速航行。

3) 投放 CTD 拖鱼，并储存数据。

4) 回收 CTD 拖鱼。

5.2.3 颠倒温度表测温方法见附录 B。

5.2.4 标准层水温的观测

标准层的水温，可利用 CTD、XBT、XCTD 和走航式 CTD（MVP300）等仪器测得的标准层上、下相邻的观测值通过内插求得；也可利用颠倒温度表测得。

5.3 资料处理

5.3.1 CTD仪观测记录的整理

CTD资料的处理原则上按照仪器制造公司提供的数据处理软件或通过鉴定的软件实施。其基本规则和步骤如下：

a) 将仪器采集的原始数据转换成压力、温度及电导率数据；

b) 对资料进行编辑；

c) 对资料进行质量控制，主要包括剔除坏值、校正压强零点以及对逆压数据进行处理等；

d) 进行各传感器之间的延时滞后处理；

e) 取下放仪器时观测的数据计算温度，并按规定的标准层深度记存数据。

5.3.2 现场XBT、XCTD和走航式CTD(MVP300)资料处理

走航测温资料处理的规则如下：

a) XBT、XCTD和走航式CTD(MVP300)探头测量的原始数据，通过厂家提供的数据处理软件或通过鉴定的软件进行转换和处理。

b) XBT的资料信息也可通过发射机向有关卫星发射。

c) XCTD应通过它的校准系数计算出温度等要素。

5.3.3 颠倒温度表观测记录的整理方法见附录B。

6 盐度测量

6.1 技术指标

6.1.1 盐度测量的准确度

主要根据项目的要求和研究目的，同时兼顾观测海区和观测方法的不同以及仪器的类型，按表3确定盐度测量的准确度。

表3 盐度测量的准确度和分辨率

准确度等级	准确度	分辨率
1	±0.02	0.005
2	±0.05	0.01
3	±0.2	0.05

6.1.2 观测时次

盐度与水温同时观测。大面或断面测站，船到站观测一次；连续测站，每小时观测一次。

6.1.3 盐度测量的标准层次

盐度测量的标准层次与温度相同，见表2。

6.2 观测方法

6.2.1 温盐深仪(即CTD)定点测量盐度

基本步骤和要求如下：

a) 利用CTD测量盐度与测量温度是在同一仪器上实施，其观测步骤和要求基本相同，见5.2.1.2。

b) 利用CTD测盐度时，每天至少应选择一个比较均匀的水层，与利用实验室盐度计对海水样品的测量结果比对一次。深水区测量盐度时，每天还应采集水样，以便进行现场标定。如发现CTD的测量结果达不到所要求的准确度，应及时检查仪器，必要时更换仪器传感器，并应将比对和现场标定的详细情况记入观测值班日志。

c) CTD的电导率传感器应保持清洁。每次观测完毕，都须用蒸馏水(或去离子水)冲洗干净，不能残留盐粒或污物。

6.2.2 走航测量盐度

利用 XCTD 和走航式 CTD(MVP300)测盐度与利用这些仪器测温度的观测步骤和要求相同，见 5.2.2.2。

6.2.3 实验室盐度计测量海水样品盐度见附录 A。

6.3 资料处理

6.3.1 CTD 仪测盐度资料的处理

CTD 测得的盐度记录，可照 5.3.1 进行整理。测量的电导率值换算成盐度后，如在跃层中有明显的"异常尖锋"存在时，应将电导率或温度测量值进行时间滞后订正，然后再重新计算盐度。

6.3.2 XCTD 和走航式 CTD(MVP300)资料处理

XCTD 和走航式 CTD(MVP300)资料处理见 5.3.2。

6.3.3 实验室盐度计测量海水样品盐度资料处理见附录 A。

7 海流观测

7.1 技术指标

7.1.1 观测要素

主要观测要素为流速和流向。辅助观测要素为风速和风向，辅助要素的观测应符合 GB/T 12763.3 的有关规定。

7.1.2 测量的准确度

海流观测方式多种，有定点测流、漂流浮标和走航测流。对于定点测流，应达到表 4 中规定的准确度。

表 4 海流观测的准确度

流速/(cm/s)	水深/m	准确度	
		流速	流向
<100	≤200	±5 cm/s	±5°
	>200	±3 cm/s	
≥100	≤200	±5%	
	>200	±3%	

7.1.3 观测层次

参照表 2，根据需要选定。

7.1.4 海流观测的取样时段

流向一般为瞬时值；流速值通常使用 3 min 的平均流速。否则，应在观测记录上说明取样时段。

7.1.5 连续观测的时间长度与时次

海流连续观测的时间长度应不少于 25 h，至少每小时观测一次。预报潮流的测站，一般应不少于三次符合良好天文条件的周日连续观测。

7.2 观测方法

7.2.1 海表面漂移浮标测流

7.2.1.1 仪器设备

目前使用较为普遍的仪器设备是卫星跟踪海表面漂流浮标。

7.2.1.2 观测步骤和要求

漂流浮标按以下步骤和要求投放：

a) 布放前，应提前租用有关卫星的接收通道。

b) 投放前，应用信号感应器测试发射机工作情况。发射机工作正常，能连续工作，方可投放。

c) 漂流浮标最好在停船前或开船后 1 kn 的航速下，在船尾部的左侧或右侧投放。投放时先放漂流袋，后放漂流体。

d) 投放结束后，应及时填写漂流浮标观测记录表(见表 D.9)。

7.2.2 船只锚碇测流

7.2.2.1 仪器设备

在锚碇船上，用以测流的仪器大致可分为直读和自记两大类。目前常用的主要有直读海流计(非自记)和安德拉海流计(自记)等。

7.2.2.2 观测步骤和要求

以锚碇船为承载工具，观测海流的基本步骤和要求如下：

a) 观测期间首先应按表 D.10 的格式记录观测日期、站位(经度、纬度)等有关信息。

b) 利用直读等类型非自记海流计测海流时，待海流计沉放至预定水层后，即可进行流速和流向的测量。室内终端设备直接显示观测数据，可采用手工记录，也可采用记录仪记录。

c) 安德拉等类型自记海流计观测海流时，可根据绞车和钢丝绳的负载，以及观测任务的具体要求，串挂多台海流计同时测多层海流。测流时应记录观测开始时间和结束时间。观测结束后，取出内存记录板，使用厂家配带的交换器与计算机通讯口相连，在计算机上读取观测数据。

d) 当施放海流计的钢丝绳或电缆的倾角超过 10°时，应对仪器沉放深度进行倾角订正。

e) 在锚碇船上进行海流连续观测时，应每三小时观测一次船位。如发现船只严重走锚(超过定位准确度要求)，应移至原位，重新开始观测。

f) 周日连续观测一般不得缺测。凡中断观测两小时以上者，应重新开始观测。

7.2.3 锚碇潜标测流

7.2.3.1 潜标的组成

潜标系统的组成参见图 1 和图 2 实例，常用的海流计有 ADCP 和安德拉海流计等。

7.2.3.2 观测步骤和要求

锚碇潜标测流按以下步骤和要求实施：

a) 任务的准备

——根据研究目的和任务要求，同时参考收集到的观测海区风场、流场、水深、地形和海底底质及船只设备状况，确定锚碇系统的系留方式(见图 1 和图 2 实例)，并拟定详细的布放方案。

——出海前进行仪器的实验检查，使海流仪和声学释放器处于正常工作状态。

——按锚碇系统设计、计算，准备好全部器材。

b) 锚碇系统的投放

——船只到达锚碇投放点前，进行海流仪的采样设置，再次检查海流仪和声学释放器的工作状态，尤其是无线电发射机的状态。

——在甲板上连接各部件。

——船只到达测点后，最好抛锚并用 GPS 进行准确定位；如果水深太大，船只无法抛锚，则应随时定位，确保锚碇仪器的准确位置。同时，注意调整船向，使作业一舷迎风向。

——布放步骤：在浅水海区(水深小于 200 m)，一般应按“先锚后标”的顺序，首先放沉块，然后顺次下放声学释放器、海流计、浮力球；在深水海区，则应按“先标后锚”的顺序，首先顺次下放浮力球、海流计、声学释放器，然后释放沉块，沉块拉着锚定系统直沉海底。

——以上各步骤都应详细记录，内容包括海流仪采样设置，开始工作时间，下水时间，沉块着落海底的时间，锚碇的精确位置及有否异常情况等。

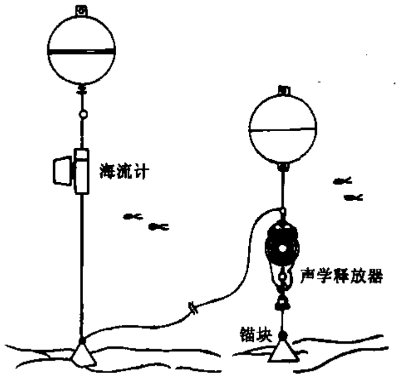

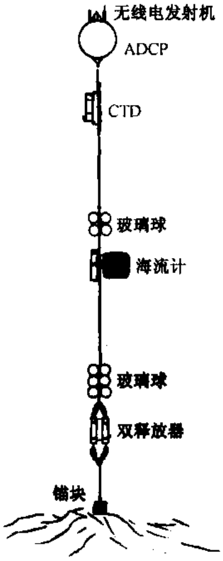

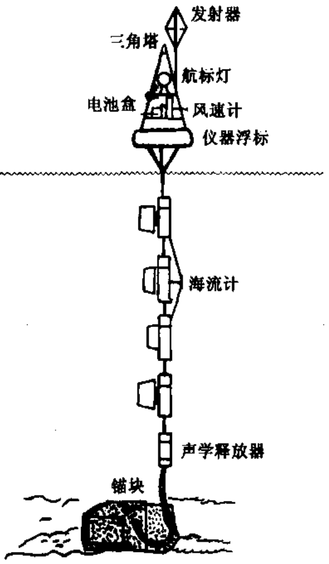

图 1　锚碇浅水应用型潜标　　**图 2　锚碇深水应用型潜标**　　**图 3　锚碇明标系统的组成**

c)　锚碇系统的回收

——回收船只应有 GPS 或其他定位设备，并应备有工作艇。

——回收应尽量在良好海况下的日间进行。

——当船只到达锚碇站后，把声学应答器放至海面下 5 m～10 m 处，发射指令信号，同时注意搜索上浮的浮标。

——浮标上浮后，用抛钩钩住系统的尼龙绳，利用船上的吊车和绞缆机收回锚碇系统；必要时，亦可放下工作艇，把缆绳系到浮标上收回锚碇系统。

7.2.4　锚碇明标测流

7.2.4.1　明标系统的组成

与潜标系统相比，主要增加了水上浮筒部分（内装有电池盒和闪光装置）（见图 3 实例）。

7.2.4.2　观测步骤和要求

a)　锚碇系统的投放

——明标投放前，应根据有关规定发布航行通告。

——明标投放方法与潜标的投放基本相同（见 7.2.3.2.a）、b））。

——明标上的闪光装置应切实水密，保证正常连续闪光。

b)　锚碇系统的回收

明标目标清晰，当船只到达锚碇站后，即可利用船只上的吊车和绞缆机收回锚碇系统。

7.2.5　走航测流

7.2.5.1　仪器设备

走航测流主要使用船载 ADCP 进行海流观测。

7.2.5.2 观测步骤和要求

使用船载 ADCP 进行海流观测应按以下步骤和要求实施：

a) 出海前应进行 ADCP、ADCP 换能器舱、罗经及 GPS 等有关设备的检查，使其皆处于正常工作状态。
b) 运行 ADCP 自检程序，记录测试程序的运行结果。
c) 检查计算机，并清理硬盘，留出足够的空间，以便存储 ADCP 观测数据。
d) 准确校准电罗经，设置 ADCP 的罗经初始角。同时校准时间，将计算机和 ADCP 的时钟与 GPS 时钟校准，校准误差应小于 1.0 s。
e) 按照技术设计书的要求，设置 ADCP 的测层间隔、数据平均方式和航次识别符等参数，建立设置文件。
f) 对新安装的 ADCP，应根据底跟踪资料，计算出 ADCP 换能器的方向修正角，并输入设置文件。
g) 启动数据采集程序，调入配置文件，检查基本参数，当一切正常后开始采集数据。采集过程中，应记录原始数据文件、平均数据文件、导航数据文件等。更改 ADCP 设置后，要及时存储新的设置文件。
h) 观测过程中，应确保值班人员在位。值班人员应随时观测 ADCP 系统的工作状态，详细填写值班日记，如发现异常，应及时处理，并将处理过程和处理结果详细记录在值班日记中。
i) 在航测中，调查船应尽可能保证匀速直线航行，并保证航速不超过 ADCP 观测的临界速度。
j) 结束 ADCP 观测后，要及时备份硬盘上的观测数据。在条件许可情况下，不使用压缩方式备份数据，以避免解压失败。
k) 结束 ADCP 观测后，要重新校准计算机和 ADCP 的时钟与 GPS 时钟，并在值班日记中详细记录时钟的差值。同时，应详细填写 ADCP 观测记录表(见表 D.11)。

7.3 资料处理

7.3.1 海表面漂移浮标测流资料处理

绘制浮标漂流轨迹时间序列图，并从原始数据中剔除明显错误的数据(一般认为，在位置资料中加速度大于 0.003 4 cm/s^2 的数据为不合理数据)；然后，获取流速和流向。

7.3.2 ADCP 测流仪的资料处理

ADCP 观测数据的处理应使用通过鉴定的软件进行，其基本规则和步骤如下：

a) 首先应对原始采集数据进行以下几方面的质量控制：
   ——剔除良好率较低的数据；
   ——剔除由于船速过快，或仪器发生故障等原因产生的坏数据；
   ——标识受干扰层，剔除来自鱼群等物体的干扰。
b) 剔除 ADCP 观测资料中的船速，计算得到真实流速。
c) 插值计算出各标准层的流速，并将处理结果按规定格式存入数据文件。
d) 绘制流的时间序列矢量图和垂直分布图。

7.3.3 其他类型海流计测流资料的整理

按以下规则和步骤实施：

a) 利用以数字显示、记录纸带或直读等记录方式的海流计测流时，应按仪器的技术性能所要求的方法和程序对所测数据进行整理，求得实际流速和真流向。
b) 利用内存记录板的自容式海流计测流时，记录板可通过厂家配带的交接器与计算机通讯口相连，在计算机上读取观测值或直接打印出流速和流向值。

8 海浪观测

8.1 技术指标

8.1.1 观测要素

主要观测要素为波高、周期、波向、波型和海况。辅助要素为风速和风向，辅助要素的观测应符合

GB/T 12763.3 的有关规定。

8.1.2 测量的单位和准确度

a) 波高测量单位为米(m),记录取一位小数。准确度规定为两级:一级为±10%,二级为±15%。

b) 周期测量单位为秒(s),准确度为±0.5 s。

c) 波向测量单位为度(°),准确度为±5°。

8.1.3 观测时次

大面或断面测站,船到站观测一次;连续测站每三小时观测一次,观测时间为北京标准时02,05,08,11,14,17,20,23时。目测只在白天进行。

8.1.4 波面记录的时间长度和采样时间间隔

自记测波仪的采样时间间隔应小于或等于0.5 s,连续记录的波数不少于100个波;记录的时间长度视平均周期的大小而定,一般取17 min~20 min。

8.2 观测方法

8.2.1 目测方法

8.2.1.1 观测点和观测海域的选择

目测海浪时,观测员应站在船只迎风面,以离船身30 m(或船长之半)以外的海面作为观测区域(同时还应环视广阔海面)来估计波浪尺寸和判断海浪外貌特征。

8.2.1.2 海况的观测

以目力观测海面征象,根据海面上波峰的形状、峰顶的破碎程度和浪花出现的多少,按表5判断海况所属等级,并填入记录表中。

表5 海况等级表

海况(级)	海 面 征 状
0	海面光滑如镜
1	波纹
2	风浪很小,波峰开始破碎,但浪花不显白色
3	风浪不大,但很触目,波峰破裂,其中有些地方形成白色浪花——白浪
4	风浪具有明显的形状,到处形成白浪
5	出现高大的波峰,浪花占了波峰上很大的面积,风开始削去波峰上的浪花
6	波峰上被风削去的浪花开始沿海浪斜面伸长成带状
7	风削去的浪花带布满了海浪斜面,有些地方到达波谷,波峰上布满了浪花层
8	稠密的浪花布满了海浪斜面,海面变成白色,只在波谷某些地方没有浪花
9	整个海面布满了稠密的浪花层,空气中充满了水滴和飞沫,能见度显著降低

8.2.1.3 波型的观测

观测时,按表6判定所属波型,并记录其符号。海面无浪,波型栏空白。

表6 波型分类表

波型	符号	海 浪 外 貌 特 征
风浪	F	受风力的直接作用,波形极不规则,波峰较尖,波峰线较短,背风面比迎风面陡,波峰上常有浪花和飞沫
涌浪	U	受惯性力作用传播,外形较规则,波峰线较长,波向明显,波陡较小
混合浪	FU	风浪和涌浪同时存在,风浪波高和涌浪波高相差不大
	F/U	风浪和涌浪同时存在,风浪波高明显大于涌浪波高
	U/F	风浪和涌浪同时存在,风浪波高明显小于涌浪波高

8.2.1.4 **波向的观测**

a) 观测波向时，观测员应站在船只较高位置，利用罗经方位仪，使其瞄准线平行于离船舷较远的波峰线，转动90°后使其对着波浪的来向，读取罗经刻度盘上的度数即为波向(用磁罗经测波向须进行磁差校正)。

b) 当海上无浪或浪向不明时，波向记C；风浪和涌浪同时存在时，波向分别观测，并填入记录表中。

8.2.1.5 **波高和周期的观测**

a) 目测波高和周期时，应先环视整个海面，注意波高的分布状况，然后目测10个显著波(在观测的波系中，较大的、发展完好的波浪)的波高及其周期，取其平均值，即为有效波高(H_s)及其对应的有效波周期。从10个波高记录中选取一个最大值作为最大波高H_{max}。将H_s、H_{max}填入记录表中。

b) 当波长小于船长时，可将甲板与吃水线间的距离作为参考标尺来测定波高；而以相邻两个显著波峰经过海面浮动的某一标志物的时间间隔，作为这个波的周期。

c) 当波长大于船长时，应在船只下沉到波谷后，估计前后两个波峰相对于船高的几分之几(或几倍)来确定波高；而以船身为标志物，相邻两个显著波峰经过此物的时间间隔，作为这个波的周期。

8.2.2 **仪器观测方法**

8.2.2.1 **以船只为承载工具观测波浪**

8.2.2.1.1 **仪器设备**

目前一般采用浮球式加速度型测波仪。

8.2.2.1.2 **观测步骤和要求**

在船上采用测波仪观测海浪的主要步骤和要求如下：

a) 当船只进入作业区后，应根据风向和海流确定船只的工作方式(漂移或抛锚)和测头的施放位置。

b) 依观测点水深和海况确定仪器记录量程，按8.1.4的要求，选定采样时间间隔，在采样的时间长度(17 min～20 min)测定不少于100个波的波高和周期，取其中100个连续波求得各特征值或记录波面模拟曲线。

c) 观测位置应避开影响海浪的障碍物，如暗礁、浅滩、岛屿和人工建筑物等。测点附近有障碍物时，应记录影响海浪的情况。

d) 在强流区测波时，不宜采用海流会导致海浪记录漂零等误差的测波仪；测点附近有强电干扰时，不宜采用遥测波浪仪。

8.2.2.2 **锚碇测波**

8.2.2.2.1 **仪器设备**

锚碇测波常使用声学测波仪和重力测波仪。

8.2.2.2.2 **观测步骤和要求**

锚碇测波的主要步骤和要求如下：

a) 应根据项目要求以及观测现场的海洋环境，选用测波仪类型，并确定浮标系留方式。

b) 锚碇系统连接前，应对仪器各项性能进行测试，确认仪器良好方可使用。

c) 锚系的投放与回收步骤，可参见7.2.3.2和7.2.4.2。

8.3 **资料处理**

8.3.1 **仪测海浪记录的整理**

按以下规则实施：

a) 仪测海浪记录以模拟曲线形式给出时，自海浪连续记录中量取相邻两上跨(或下跨)零点(图4

中的 A_1、A_2)间一个显著波峰与一个显著波谷间的铅直距离作为一个波的波高；量取相邻两个显著波峰(图 4 中的 C_2、C_3)或两个上跨零点的时间间隔作为一个波的周期。然后依有效波高和有效波周期定义，计算有效波高和有效波周期。选取所有波高中的最大值为最大波高，其所对应的周期为最大波周期。根据有效波高查波级表(见表 C.1)得波级。

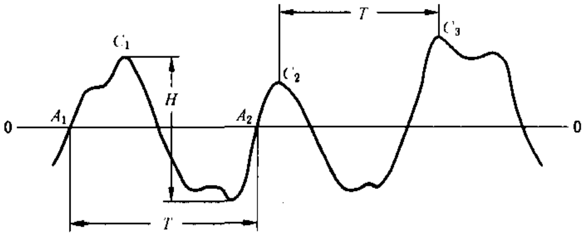

图 4　波面随时间的变化曲线

b) 海浪以存储器记录时，可利用仪器公司或有关商家提供的专用软件进行处理，并可直接打印出有效波高、有效波周期，最大波高和最大波周期。根据有效波高查波级表(见表 C.1)得波级。

8.3.2　目测海浪记录的整理

从目测的 10 个显著波的波高和周期分别取平均值，得有效波高和有效波周期。10 个波高中的最大值为最大波高，其所对应的周期为最大波周期。根据有效波高查波级表得波级。目测海浪记录表格参见表 D.1。

9　水位观测

9.1　技术指标

9.1.1　需测的量

水位观测需测的量如下：

a) 总压强，它是气压与水压的总和。由水位计的压力传感器测得，单位为 kPa。

b) 现场水温，由水位计的温度传感器测得，单位为℃。

c) 现场气压，由自记气压表测得，单位为 kPa。

9.1.2　水位测量的准确度

水位测量的准确度规定为三级：一级为±0.01 m；二级为±0.05 m；三级为±0.10 m。

9.1.3　取样时间间隔

连续观测在 30 d 以内时，取样时间间隔为 5 min；连续观测超过 30 d 时，取样时间间隔定为 10 min。

9.2　观测方法

9.2.1　仪器设备

可采用压力式和声学式等水位计进行观测。

9.2.2　观测步骤和要求

水位测量一般按以下步骤和要求实施：

a) 观测水位通常采用锚定系留方式(见图 1～图 3 实例)；

b) 测量水位锚碇系统的投放和回收步骤见 7.2.3.2 和 7.2.4.2；

c) 观测前应检查水位计的取样间隔开关是否在正确位置上，上好内存记录板，打开主机开关，记下第一次取样时间；

d) 水位观测应防止仪器下陷，确保仪器在垂直方向没有变动；

e) 观测结束收回仪器后应先用淡水冲洗，然后打开仪器。如仪器工作正常，待仪器再工作一次并记录完毕，记下结束时间，关上主开关，取出记录板，并应放入盒中妥善保存。

9.3 资料处理

水位测量资料按以下要求和方法处理：

a) 记录板可通过厂家配带的交接器与计算机通讯口相连，在计算机上读取观测值或直接打印出原始数据。

b) 根据打印的数据、记录的起止日期和时间，检查数据的总数是否正确，有无误码以及资料是否正常。

c) 经审查确认数据无误后，可用原始软盘上机，并同时输入现场气压值、海水密度值和重力加速度值进行计算，求得水位的变化值和逐时值，水位的计算公式见式(1)。

$$H = 10^3(p - p_a)\frac{1}{\rho g} \qquad \cdots\cdots(1)$$

式中：

H——水位，单位为米(m)；

p——总压强，单位为千帕斯卡(kPa)；

p_a——现场气压，单位为千帕斯卡(kPa)；

ρ——海水密度，由海水盐度与温度的关系曲线查得，或根据历史温、盐资料算得。单位为千克每立方米(kg/m^3)。

G——重力加速度，根据测站所在纬度算得。单位为米每二次方秒(m/s^2)。

10 海水透明度、水色和海发光观测

10.1 技术指标

10.1.1 海水透明度

透明度观测只在白天进行，大面或断面测站，船到站观测一次；连续测站，每两小时观测一次。计量单位为 m，观测时读数取一位小数。

10.1.2 水色

水色观测与透明度观测同时进行，准确度为±1 级。

10.1.3 海发光

10.1.3.1 观测要素

发光类型和发光强度(等级)。

10.1.3.2 观测时次

海发光观测只在夜间进行。大面或断面测站，船到站观测一次，并在两站的航行中观测一次；连续测站，在当地时间 20，23 和 02 时观测。发光类型依发光特征分三类，每类又依发光强弱分五级(见表 C.3)。

10.2 观测方法

10.2.1 海水透明度观测

按以下步骤和要求实施：

a) 海水透明度应用透明度盘进行观测。透明度盘为直径 30 cm，底部系有重锤，上部系有绳索的木质或金属质白色圆盘。绳索上有以米为单位的长度标记。

b) 透明度盘的绳索标记使用前应进行校正。标记必须清晰、完整。新绳索须事先进行缩水处理。

c) 透明度盘应保持洁白。当油漆脱落或脏污时应重新油漆。

d) 观测应在主甲板的背阳光处进行。观测时将透明度盘铅直放入水中，沉到刚好看不见的深度

后，再慢慢提升到白色圆盘隐约可见时读取绳索在水面的标记数值，即为该次观测的透明度值。有波浪时，应分别读取绳索在波峰和波谷处的数值标记，读到一位小数，重复2～3次，取其平均值作为该次观测的透明度值。观测结果记入表D.2。

e) 若倾角超过10°，则应进行深度订正。当绳索倾角过大时，盘下的铅锤应适当加重。

10.2.2 水色观测

按以下步骤和要求实施：

a) 水色依水色计目测确定。观测完透明度后，将透明度盘提升到透明度值一半的水层，根据透明度盘上方海水呈现的颜色，在水色计中找出与之相似的色级号码，即为该次观测的水色。观测时观测者的视线必须与水色计玻璃管垂直。观测结果记入表D.2。

b) 水色计应保存在阴凉干燥处，切忌日光照射，以免褪色。发现褪色现象，应立即更换。

c) 水色计在六个月内至少应与标准水色计校准一次。作为校准用的标准水色计(在同批出厂的水色计中保留一盒)平时应始终装在里红外黑的布套中，保存在阴凉处。

10.2.3 海发光观测

按以下步骤和要求实施：

a) 观测点应选在船上灯光照不到的黑暗处。当观测员从亮处到暗处观测时，待适应环境后再进行观测。

b) 观测时依表C.3所述发光特征目视判定发光类型，以符号记录。并依发光强弱程度及征象目视判定发光强度等级，按五级记录。当两种或两种以上海发光类型同时出现时，应分别记录。因月光强，无法观测时记“X”，无海发光时记“O”。观测结果记入表D.2。

c) 海面平静观测不到海发光时，可用杆子搅动海水，然后进行观测。

11 海冰观测

11.1 技术指标

11.1.1 观测要素

包括主要观测要素和辅助观测要素：

a) 浮冰观测的要素为：冰量、密集度、冰型、表面特征、冰状、漂流方向和速度、冰厚及冰区边缘线。

b) 固定冰观测的要素为：冰型、冰厚和冰界。

c) 冰山观测的要素为：位置、大小、形状及漂流方向和速度。

d) 海冰的辅助观测要素为：海面能见度、气温、风速、风向及天气现象。辅助观测项目应符合GB/T 12763.3的有关规定。

11.1.2 观测要素的单位与准确度

各观测要素测量的单位和准确度见表7。

表7 海冰观测要素的单位和准确度

观测要素	单位	准确度
海冰冰量、密集度	成	±1
漂流方向	(°)	±5
漂流速度	m/s	±0.1
冰厚	cm	±1

11.1.3 观测时次

大面或断面测站，船到站即观测；连续测站，每两小时观测一次。

11.2 观测方法

11.2.1 观测点的位置

海冰通常在调查船或飞机上进行观测。船上观测海冰的位置,应尽可能选在高处。观测对象应以二倍于船长以外的海冰为主,以避免船只对海冰观测的影响。

11.2.2 浮冰观测

11.2.2.1 冰量观测

按以下步骤和要求实施:

a) 浮冰量为浮冰覆盖整个能见海面的成数。用 0～10 和 10^-,共 12 个数字和符号表示。记录时取整数。

b) 观测时环视整个海面,估计浮冰分布面积占整个能见海域面积的成数。海面无冰时,记录栏空白;浮冰分布面积占整个能见海域面积不足半成时,冰量记"0";占半成以上,不足一成半时,冰量记"1",余类推。整个能见海面布满浮冰时,冰量记"10",有缝隙时记"10^-"。

c) 海面能见度小于或等于 1 km 时,不进行冰量观测,记录栏记横杠"—"。

11.2.2.2 密集度观测

按以下步骤和要求实施:

a) 密集度为浮冰覆盖面积与浮冰分布面积的比值。密集度观测和记录方法与冰量相同。海面无冰时,密集度栏空白;冰量为"0"时,密集度记"0"。

b) 当浮冰分布的海域内有超过其面积一成以上的完整无冰水域时,此水域不能算作浮冰分布海域。当海面上有两个或两个以上浮冰分布区域时,应分别进行观测,取平均值作为密集度。

11.2.2.3 冰型观测

按以下步骤和要求实施:

a) 冰型是根据海冰的生成原因和发展过程而划分的海冰类型。观测时环视整个能见海面,根据表 C.4 判断其所属类型,用符号记录。

b) 当海面上同时存在多种冰型时,按量多少依次记录;量相同时,按厚度大小的顺序记录。每次观测最多记五种。

c) 当海冰距离观测点很远,无法判定冰型时,冰型栏记横杠"—"。

11.2.2.4 冰表面特征观测

按以下步骤和要求实施:

a) 冰表面特征是指浮冰在动力或热力作用下所呈现的外貌。观测时环视整个能见海面,按表 D.3判断其所属种类,用符号记录。

b) 当同时存在两种或两种以上冰表面特征时,按其数量多少依次记录;量相同时,按表 C.5 所列顺序记录。每次观测最多记三种。

11.2.2.5 冰状观测

a) 冰状是浮冰冰块最大水平尺度的表征。观测时环视整个能见海面,按表 C.6 判定其所属冰状,以符号记录。

b) 当几种冰状同时存在时,按其数量多少依次记录。数量相同时,按表 C.6 所列顺序记录。每次观测最多记三种。

11.2.2.6 漂流方向和速度的观测

按以下步骤和要求实施:

a) 漂流方向指浮冰漂流的去向,以度(°)表示;漂流速度为单位时间内浮冰移动的距离,以 m/s 为单位,取一位小数。

b) 漂流方向和速度应在锚定船只上利用雷达或罗经和测距仪进行观测。观测时,首先在雷达荧光屏或海面上两倍于船长距离以外选择具有明显特征的浮冰块,测定其方向和至船的距离(起

点位置)，同时启动秒表记时。当所测冰块移动距离超过原离船距离的二分之一或其方向改变20°时，读取时间间隔，同时测定其方向和距离(终点位置)。然后，根据起点位置和终点位置的方向和距离，用矢量法计算或用计算圆盘求得浮冰的漂流方向和移动距离。再用移动距离除以间隔时间，便得漂流速度。

c) 无仪器时，可根据浮冰块的移动特征，按表8估测漂流速度(v)，以等级记录。

表8 浮冰漂流速度的目测估计

冰块移动特征	相当速度/(m/s)	速度等级
很慢	$v<0.3$	1
明显	$0.3\leqslant v<0.5$	2
快	$0.5\leqslant v<1.0$	3
很快	$v\geqslant 1.0$	4

d) 海面无浮冰或仅有初生冰时，流向、流速栏空白；漂流速度小于0.05 m/s时，流向记“C”，流速记“O”；海面有浮冰，但无法观测漂流速度和方向时，应在备注栏说明。

11.2.2.7 冰厚观测

冰厚为从冰面至冰底的垂直距离，单位为厘米(cm)，记录时只取整数。

观测时可用绞车或网具捞取冰块(最好取三个以上)，分别测量冰块厚度，最后取其平均值作为冰厚观测值。或选择有代表性的冰块，用冰钻钻孔，用冰尺测量其厚度。

11.2.2.8 冰区边缘线观测

冰区边缘线指海冰分布区域的廓线，也即冰水分界线。当冰区与开阔水域存在明显分界线时进行此项观测。观测时环视整个能见海域，在冰水分界线上选定几个特征点(一般不少于三个，远离冰区的少量冰块不能选作特征点)，用雷达或罗经和测距仪测出各点相对测站的方向和距离。将各特征点标注在调查研究海区空白图上，用圆滑曲线连接各特征点，即为冰区边缘线。观测不到冰区边缘线时，应在备注栏说明。

11.2.2.9 浮冰观测结果的记录

浮冰各观测项目的观测结果，记入浮冰观测记录表。记录表格式见表D.3。

11.2.3 固定冰观测

11.2.3.1 冰型观测

按以下步骤和要求实施：

a) 固定冰冰型是依冰的生成和形态等划分的固定冰类型。观测时环视整个能见海面，按表C.7判定其所属类型，用符号记录。

b) 当海面上同时存在多种冰型时，按表C.7的顺序记录。

c) 当海冰距离观测点很远，无法判定冰型时，冰型栏记横杠“—”。

11.2.3.2 冰厚观测

冰厚观测通常用冰钻和冰尺进行。

测点选好后，用冰钻钻孔。钻孔过程中，冰钻应保持垂直状况，直至钻透为止，然后将冰尺插入冰孔测量其厚度。

11.2.3.3 冰界观测

固定冰冰界为固定冰和浮冰或固定冰和无冰水域的分界。观测方法与浮冰冰区边缘线的观测方法相同。

11.2.3.4 固定冰观测结果的记录

固定冰各观测项目的观测结果记入固定冰观测记录表。记录表格式见表D.4。

11.2.4 **冰山观测**

11.2.4.1 **冰山位置观测**

用雷达或GPS观测确定出冰山实际位置。

11.2.4.2 **冰山大小的观测**

根据冰山露出水面部分的高度和水平尺度，将其分为四级（见表C.8）。观测时以高度为主，按表C.8确定其等级，并以符号记录。

11.2.4.3 **冰山形状观测**

冰山形状分平顶（桌状）、圆顶、尖顶和斜顶四种。观测时按表C.9目视判定，以符号记录。

11.2.4.4 **冰山漂流方向和速度观测**

观测与记录方法与浮冰相同。

11.2.4.5 **冰山观测结果的记录**

冰山观测项目的观测结果，记入冰山观测记录表。记录表格式见表D.5。

11.3 **资料处理**

资料处理按以下要求进行：

a) 根据海冰观测记录，在观测海区空白图上绘制冰情图。冰情图的内容包括：冰区边缘线、冰区内各测点的观测结果及航线附近冰山的分布和漂流情况。

b) 如视野范围内全部被海冰覆盖，不绘冰区边缘线，只绘最大视程线。

附 录 A
（规范性附录）
实验室盐度计测量海水样品盐度和计算盐度的有关公式

A.1 利用实验室盐度计测量海水样品盐度

操作使用方法如下：

a) 利用电极式或感应式实验室盐度计测定水样盐度时，须先行定标，求得温度 T(℃)时标准海水电导比的定标值，然后将定标调节在 R_T 读数各档上。重复调节定位校准各档，使得两次读数在最后一位相差在 3 以内定标结束。测量海水样品时，定位校准各旋钮不再变动。

b) 测量海水样品时，先将海水样品注入电导池中，待启动搅拌器搅拌 1 min～2 min 后测量海水样品的温度。调节 R_T 各档旋钮，使表头指针趋于零，读取 R_T 值。若发现读数可疑，必须重测，直至认为数据合理为止。将测量的海水样品温度及 R_T 值记入盐度分析记录表中。记录表格式见附录 D 的表 D.7。

c) 连续观测时，每天至少应用标准海水定标一次。定标和测量时，不得在未搅拌的情形下进行，且电导池内不允许存在气泡和其他漂浮物。

d) 被测海水样品的温度与标准海水的温度相近时方可进行测量。感应式盐度计要求两者相差在 2℃以内；电极式盐度计要求在搅拌器启动 1 min～2 min，待两者温度基本趋于相等。

e) 工作结束后，电导池应用蒸馏水清洗干净。电极式盐度计应在电导池内注满蒸馏水，以保护电极。

A.2 实用盐度的确定

实用盐度由 K_{15} 通过式(A.1)确定。

$$S = a_0 + a_1 K_{15}^{1/2} + a_2 K_{15} + a_3 K_{15}^{3/2} + a_4 K_{15}^{2} + a_5 K_{15}^{5/2} \quad \cdots\cdots\cdots\cdots\cdots\cdots\cdots(\mathrm{A.1})$$

式中：

K_{15} 为温度在 15℃时，一个标准大气压下海水样品的电导率，与相同温度和压强下质量比为 $32.435\,6\times10^{-3}$ 的氯化钾溶液的电导率的比值；

$a_0 = 0.008\,0$；

$a_1 = -0.169\,2$；

$a_2 = 25.385\,1$；

$a_3 = 14.094\,1$；

$a_4 = -7.026\,1$；

$a_5 = 2.708\,1$。

当 K_{15} 精确地等于 1 时，海水样品的实用盐度恰好等于 35，即 $\sum a_i = 35.000\,0$。式(A.1)的适用盐度范围为：$2 \leqslant S \leqslant 42$。

A.3 电导率换算为盐度

根据测得的电导率、温度和压强数据，经过处理后，可借助以下实用盐度计算公式(A.2)，将电导率换算为盐度。

$$S = a_0 + a_1 R_T^{1/2} + a_2 R_T + a_3 R_T^{3/2} + a_4 R_T^{2} + a_5 R_T^{5/2} + \frac{T-15}{1+K(T-15)}[b_0 + b_1 R_T^{1/2} + b_2 R_T + b_3 R_T^{3/2} + b_4 R_T^{2} + b_5 R_T^{5/2}] \quad \cdots\cdots\cdots(\mathrm{A.2})$$

式中：

R_T——被测海水样品与实用盐度为 35 的标准海水样品，在相同温度和一个标准大气压下电导率的比值；

T——被测海水样品的温度，单位为摄氏度(℃)。

$b_0=0.000\ 5$；

$b_1=-0.005\ 6$；

$b_2=-0.006\ 6$；

$b_3=-0.037\ 5$；

$b_4=0.063\ 6$；

$b_5=-0.014\ 4$；

$K=0.016\ 2$。

a_0, a_1, a_2, a_3, a_4 和 a_5 的值同式(A.1)。

电导比 R_T，可根据现场测得的电导率、温度和压强值，通过式(A.3)计算。

$$R_T = \frac{R}{R_p \cdot r_T} \qquad \cdots\cdots(A.3)$$

式中：

R——现场测得的电导率与 $S=35, T=15℃, p=0$ 时标准海水电导率的比值；

R_p——现场测得的电导率与同一样品在相同温度和 $p=0$ 条件下电导率的比值；

r_T——实用盐度为 35 的参考海水在温度为 T(℃)时与其在 15℃时电导率的比值。

R_p 可通过式(A.4)计算。

$$R_p = 1 + \frac{(A_1 + A_2 p + A_3 p^2)p}{1 + B_1 T + B_2 T^2 + (B_3 + B_4 T)R} \qquad \cdots\cdots(A.4)$$

式中：

p——现场测得的压强，单位为千帕斯卡(kPa)；

T——现场测得的海水温度，单位为摄氏度(℃)；

R——现场测得的电导率与 $S=35, T=15℃, p=0$ 的标准海水电导率的比值；

$A_1=2.070\times10^{-6}$；

$A_2=-6.370\times10^{\ 12}$；

$A_3=3.989\times10^{-18}$；

$B_1=3.426\times10^{-2}$；

$B_2=4.464\times10^{-4}$；

$B_3=4.215\times10^{-1}$；

$B_4=-3.107\times10^{-3}$。

r_T 的计算公式见式(A.5)。

$$r_T = C_0 + C_1 T + C_2 T^2 + C_3 T^3 + C_4 T^4 \qquad \cdots\cdots(A.5)$$

式中：

T——现场测得的海水温度，单位为摄氏度(℃)；

$C_0=0.676\ 609\ 7$；

$C_1=2.005\ 64\times10^{-2}$；

$C_2=1.104\ 259\times10^{-4}$；

$C_3=-6.969\ 8\times10^{-7}$；

$C_4=1.003\ 1\times10^{-9}$。

上述式(A.2)、式(A.3)和式(A.5)，在温度为 $-2℃\sim35℃$，压强为 0 kPa～10^5 kPa，实用盐度为

2～42 范围内均有效。

A.4 利用实验室盐度计测量海水样品盐度记录的整理

利用实验室盐度计测量海水样品的盐度时，可根据测得的电导比 R_T 值和海水样品的温度 T 值，通过(A.2)式计算盐度 S 值；也可利用测得的电导比和海水样品的温度查国际海洋学常用表得 S_0（未修正值）和 ΔS 值，然后将两者相加便得盐度值，$S=S_0+\Delta S$。

附 录 B
（规范性附录）
利用颠倒温度表观测海水温度

B.1 仪器设备和主要技术指标

B.1.1 仪器设备

标准层水温通常用颠倒温度表和颠倒采水器配合进行观测。颠倒温度表分为闭端颠倒温度表和开端颠倒温度表。闭端颠倒温度表用以测量海水温度；开端颠倒温度表与闭端颠倒温度表配合使用，测量颠倒处的深度。

B.1.2 主要技术指标

颠倒温度表系列主要技术指标见表 B.1。

表 B.1 颠倒温度表系列主要技术指标

型 式	型 号	最大使用深度/m	主温度表/℃		副温度表/℃	
			分度值	示值范围	分度值	示值范围
闭端颠倒温度表	SWC_1-1	2 500	0.1	−1～+32	0.5	−20～+50
	SWC_1-2	6 000	0.05	−2～+15		
	SWC_1-4	1 000	0.1	“0”，+15～+40		
开端颠倒温度表	SWC_2-1	250	0.1	−2～+32	0.5	−20～+50
	SWC_2-2	6 000	0.2	−2～+60		
	SWC_2-3	6 000	0.1	“0”，+30～+60		

B.2 操作使用方法

B.2.1 使用前的检查

使用前应进行以下各项的检查：

a) 检查颠倒温度表的检定证书，内容应完整，在有效期内。

b) 检查所用颠倒温度表是否合格。

c) 经检查确认合格的颠倒温度表，在同一采水器上放两支闭端颠倒温度表的情况下，要做配对工作。挑选 V_0 值接近、外形尺寸相近的两支配对使用。

B.2.2 安装和测量

使用颠倒温度表测量海水温度按以下步骤和要求实施：

a) 将选好的颠倒温度表安装到采水器上，观测 100 m 以浅（含 100 m）各标准层的水温时，使用双管式温度表套管，安装两支 V_0 值相近的闭端颠倒温度表；观测 100 m 以深各标准层的水温时，使用三管式温度表套管，安装两支 V_0 值相近的闭端颠倒温度表和一支开端颠倒温度表。

b) 将装好温度表的采水器从表层至深层依次安放在采水器架上。根据测站水深确定观测层次，将各层采水器编号、颠倒温度表器号和 V_0 值记入表 D.6.1（水深小于或等于 100 m）或表 D.6.2（水深大于 100 m）。

c) 观测时，将绳端系有重锤的绞车钢丝绳移至舷外，将底层采水器挂在重锤以上 1 m 处的钢丝绳上。然后根据各观测水层之间的间距下放钢丝绳，并将采水器依次挂在钢丝绳上。若存在温

跃层时，在跃层内应适当增加测层。

d) 当水深在 100 m 以浅时，在悬挂表层采水器之前，应先测量钢丝绳倾角。倾角大于 10°时，应求得倾角订正值。若订正值大于 5 m，应每隔 5 m 增挂一个采水器，直到底层采水器离预定的底层在 5 m 以内再挂表层采水器，最后将其下放到表层水中。

e) 颠倒温度表的感温时间自最上层（表层）采水器沉入水中后开始计时，感温 7 min～10 min 后，测量钢丝绳倾角，投下“使锤”（连续观测时正点打锤），记下钢丝绳倾角和打锤时间。并用手轻触钢丝绳，凭感觉到的振动次数（每个采水器振动两次）来判断最下层采水器是否已经颠倒。待各层采水器全部颠倒后，依次提取采水器，并将其放回采水器架原来的位置上，立即读取各层温度表的主、副温值，记入颠倒温度表测温记录表的第一次读数栏内。

f) 如需取水样，待取完水样后，第二次读取温度表的主、副温值，并记入观测记录表的第二次读数栏内。第二次读数应换人复核，若同一支温度表的主温读数相差超过 0.02℃时，应重新复核，以确认读数无误。

g) 如某预定水层的采水层未颠倒或某层水温读数有疑，应立即补测；如某水层的测量值经计算整理后，两支温度表之间的水温差值多次出现超过 0.06℃，应考虑更换其中可疑的温度表。

h) 颠倒温度表不宜长期倒置，每次观测结束后必须正置采水器。

i) 如因某种原因，不能一次完成全部标准层的水温观测时，可分两次进行。但两次观测的时间间隔应尽量缩短。

B.3 资料处理

资料处理按以下步骤和要求实施：

a) 利用颠倒温度表测标准层水温时，温度表读数须作器差订正。订正时先根据主、副温度表的第二次读数，从温度表检定书中分别查得相应的订正值，再计算闭端颠倒温度表的 t（副温＋副温器差）和 T（主温＋主温器差）及开端颠倒温度表的 t'（副温＋副温器差）和 T'（主温＋主温器差）。

b) 颠倒温度表读数经器差订正后，还应作还原订正。

1) 闭端颠倒温度表还原订正值 K 的计算公式(B.1)为：

$$K=\frac{(T-t)(T+V_0)}{n_1}\left[1+\frac{T+V_0}{n_1}\right] \qquad \cdots\cdots\cdots\cdots\cdots\cdots(\text{B.1})$$

式中：

T——经器差订正后的主温表读数，单位为摄氏度（℃）；

t——经器差订正后的副温表读数，单位为摄氏度（℃）；

V_0——闭端颠倒温度表的主温表自接受泡至刻度 0℃处的水银容积（以摄氏度表示），单位为摄氏度（℃）；

$1/n_1$——水银与温度表玻璃的相对体膨胀系数，通常 $n_1=6\ 300$ 或 $6\ 100$。

闭端颠倒温度表的主温表读数经器差和还原订正后，即为实测水温 $T_W=T+K$。

2) 开端颠倒温度表还原订正值 k 的计算公式(B.2)为：

$$k=\frac{(T_W-t')(T'+V_0')}{n_2}\left[1+\frac{(T_W-t')}{2n_2}\right] \qquad \cdots\cdots\cdots\cdots\cdots\cdots(\text{B.2})$$

式中：

T_W——闭端颠倒温度表经器差和还原订正后的主温表读数，单位为摄氏度（℃）；

t'——开端颠倒温度表的副温表经器差订正后的读数，单位为摄氏度（℃）；

V_0'——开端颠倒温度表的主温表自接受泡至刻度 0℃处的水银容积，单位为摄氏度（℃）；

T'——开端颠倒温度表的主温表经器差订正后的读数，单位为摄氏度（℃）；

$1/n_2$——水银与开端颠倒温度表玻璃的相对体膨胀系数，通常 $n_2=6\ 300$ 或 6 100。

开端颠倒温度表经器差和还原订正后的主温表读数为 $T_u=T+K$。

c) 确定观测水温时，若某观测层两支颠倒温度表实测水温的差小于 0.06℃时，取两支温度表实测水温的平均值作为该层的水温；当两支颠倒温度表实测水温的差值大于 0.06℃时，可根据相邻两层的水温或前后两次观测的水温（连续观测时）的比较，取两者中合理的一个温度值，并加括号。若无法判断时，可将两个水温值都记入记录表的 T_W 档内。

d) 确定温度表测温的实际深度时，对于 100 m 以浅的水层（含 100 m），当钢丝绳倾角在 10°以内时，放出绳长即可作为温度表测温的实际深度；钢丝绳倾角超过 10°时，应作钢丝绳的倾角订正，求得温度表测温的实际深度。对于 100 m 以深的水层，可根据开、闭端颠倒温度表的 T_u 和 T_W 值，求得各温度表的计算深度，即为温度表测温的实际深度。温度表的计算深度公式(B.3)为：

$$D=\frac{10^6(T_u-T_W)}{\rho_m gQ} \quad \cdots\cdots(B.3)$$

式中：

T_u——开端颠倒温度表经器差和还原订正后的主温表读数，单位为摄氏度(℃)；

Q——颠倒温度表的压缩系数（由温度表检定证给出），单位为摄氏度每千帕斯卡(℃/kPa)；

ρ_m——从海面至开端颠倒温度表所在深度之整个水柱的平均密度，单位为千克每立方米(kg/m³)；

g——重力加速度，单位为米每二次方秒(m/s²)。

附 录 C
（规范性附录）
海洋水文观测用表

C.1 波级观测用表

波级观测用表见表C.1和表C.2。

表C.1 波级表

波级	波高/m		名称
	H_s为有效波高	$H_{1/10}$为十分之一大波波高	
0	0	0	无浪
1	$H_s<0.1$	$H_{1/10}<0.1$	微浪
2	$0.1\leqslant H_s<0.5$	$0.1\leqslant H_{1/10}<0.5$	小浪
3	$0.5\leqslant H_s<1.25$	$0.5\leqslant H_{1/10}<1.5$	轻浪
4	$1.25\leqslant H_s<2.5$	$1.5\leqslant H_{1/10}<3.0$	中浪
5	$2.5\leqslant H_s<4.0$	$3.0\leqslant H_{1/10}<5.0$	大浪
6	$4.0\leqslant H_s<6.0$	$5.0\leqslant H_{1/10}<7.5$	巨浪
7	$6.0\leqslant H_s<9.0$	$7.5\leqslant H_{1/10}<11.5$	狂浪
8	$9.0\leqslant H_s<14.0$	$11.5\leqslant H_{1/10}<18.0$	狂涛
9	$14.0\leqslant H_s$	$18.0\leqslant H_{1/10}$	怒涛

表C.2 十六个方位与度数换算表

方位	度数	方位	度数
N	348.9°～11.3°	S	168.9°～191.3°
NNE	11.4°～33.8°	SSE	191.4°～213.8°
NE	33.9°～56.3°	SW	213.9°～236.3°
ENE	56.4°～78.8°	WSW	236.4°～258.8°
E	78.9°～101.3°	W	258.9°～281.3°
ESE	101.4°～123.8°	WNW	281.4°～303.8°
SE	123.9°～146.3°	NW	303.9°～326.3°
SSE	146.4°～168.8°	NNW	326.4°～348.8°

C.2 海发光观测用表

海发光观测用表见表C.3。

表 C.3 海发光类型及强度等级表

发光类型	发光特征	发光强度等级	强度描述
火花型(H)	发光形态与萤火虫相似，当海面受机械扰动或生物受某些化学物质刺激时，此类发光显著，通常情况下发光微弱。它主要由 0.02 mm～5 mm 的发光浮游生物引起，是常见的海发光类型	0	无发光现象
		1	在机械作用下发光勉强可见
		2	在水面或风浪的波峰处发光明晰可见
		3	在风浪和涌浪波面上发光著目可见。漆黑夜晚可借此见到水面物体轮廓
		4	发光特别明亮，波纹上也能见到发光
弥漫性(M)	海面呈现一片弥漫的光辉，它主要由发光细菌引起，只要有大量细菌存在，任何情况下都会发光	0	无发光现象
		1	发光可见
		2	发光明晰可见
		3	发光著目可见
		4	强烈发光
闪光性(S)	发光常呈阵性，在机械作用或某些物质刺激下，发光较醒目。它由大型发光动物产生，这种发光动物通常孤立地出现。当其成群出现时，这种发光更显著	0	无发光现象
		1	在视野内有几个发光体
		2	在视野内有十几个发光体
		3	在视野内有几十个发光体
		4	视野内有大量的发光体

C.3 海冰观测用表

海冰观测用表见表 C.4～表 C.9。

表 C.4 浮冰冰型表

浮冰冰型	符号	特征
初生冰<br>(new ice)	N	海水直接冻结或雪降至低温海面未被融化而成。多呈针状、薄层状、油脂状或海绵状。它比较松散，且只有当它聚集漂浮在海面上时才具有一定的形状。有初生冰存在时，海面反光微弱，无光泽，遇风不起波纹
冰皮<br>(ice rind)	R	由初生冰冻结或在平静海面上直接冻结而成的冰壳层。表面光滑、湿润而有光泽，厚度 5 cm 左右，能随风起伏，易被风浪折碎
尼罗冰<br>(nilas)	Ni	厚度小于 10 cm 的有弹性的薄冰壳层。表面无光泽，在波浪和外力作用下易于弯曲和破碎，并能产生"指状"重叠现象
莲叶冰<br>(pancake ice)	P	直径 30 cm～300 cm、厚度 10 cm 以内的圆形冰块。由于彼此互相碰撞而具有隆起的边缘。它可由初生冰冻结而成，也可由冰皮或尼罗冰破碎而成
灰冰<br>(grey)	G	厚度为 10 cm～15 cm 的冰盖层，由尼罗冰发展而成。表面平坦湿润，多呈灰色，比尼罗冰的弹性小，易被涌浪折断，受到挤压时多发生重叠
灰白冰<br>(grey-white ice)	Gw	厚度为 15 cm～30 cm 的冰层，由灰冰发展而成。表面比较粗糙，呈灰白色，受到挤压时大多形成冰脊
白冰<br>(white ice)	W	厚度为 30 cm～70 cm 的冰层，由灰白冰发展而成。表面粗糙，多呈白色

表 C.5 浮冰表面特征分类表

冰面种类	符号	特　征
平整冰<br>(level ice)	L	未受变形作用影响的海冰。冰面平整或冰块边缘仅有少量冰瘤及其他挤压冻结的痕迹
重叠冰<br>(rafted ice)	Ra	在动力作用下,一层冰叠到另一层冰上形成,有时甚至三、四层冰相互重叠而成,但其重叠面的倾斜角不大,冰面仍较平坦
冰脊<br>(ridge)	Ri	碎冰在挤压作用下形成的一排具有一定长度的山脊状的堆积冰
冰丘<br>(hummock ice)	H	在动力作用下,冰块杂乱无章地堆积在一起所形成的山丘状的堆积冰
覆雪冰<br>(snow-covered)	S	表面有积雪的冰

表 C.6 浮冰冰状表

冰状类别	符　号	水平尺度/<br>m
巨冰盘<br>(giant floe)	Gf	$L \geqslant 2\ 000$
大冰盘<br>(big floe)	Bf	$500 \leqslant L < 2\ 000$
中冰盘<br>(medium floe)	Mf	$100 \leqslant L < 500$
小冰盘<br>(small floe)	Sf	$20 \leqslant L < 100$
冰块<br>(ice cake)	Ic	$2 \leqslant L < 20$
碎冰<br>(brash ice)	Bi	$L < 2$

表 C.7 固定冰冰型表

固定冰冰型	符号	特　征
冰川舌<br>(glacier tongue)	Gf	陆地冰川向一边的舌状伸展。在南极,冰川可以向海延伸数十公里以上
冰架<br>(ice shelf)	Ls	与海岸相连的、高出海面 2 m～50 m 或更高的漂浮或搁浅的冰原。其表面平滑或略起伏,向海一边比较陡峭
沿岸冰<br>(coastal ice)	Ci	沿着海岸、浅滩或冰架形成,并与其牢固地冻结在一起的海冰。沿岸冰可以随海面的升降作垂直运动
冰脚<br>(ice foot)	If	固着在海岸上的狭窄沿岸冰带,是沿岸冰流走后的残留部分,它不随潮汐变化而升降
搁浅冰<br>(stranded ice)	Si	退潮时留在潮间带或在浅水中搁浅的海冰

表 C.8 冰山等级表

等级	名 称	符 号	高度/<br>m	水平尺度/<br>m
1	小冰山	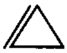	5～15	15～60
2	中冰山	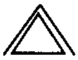	16～45	61～122
3	大冰山	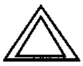	46～75	123～213
4	巨冰山	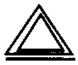	>75	>213

表 C.9 冰山形状分类表

冰山形状	符 号
平顶(桌状)冰山	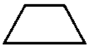
圆顶冰山	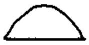
尖顶冰山	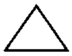
斜顶冰山	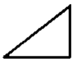

附 录 D
（资料性附录）
海洋水文观测记录表格式

海洋水文观测记录表格式见表 D.1～表 D.11。

表 D.1 目测海浪记录表

调查海区	断面号	观测日期
调查船	航次号	年 月 日至 年 月 日

序号	站号	站位						观测时间		水深 m	风向 (°)	风速 m/s	海况（级）	波型	波向 (°)	
		纬度 N/S			经度 E/W										风浪	涌浪
		(°)	(′)	(″)	(°)	(′)	(″)	时	分							

序号	站号	波要素	波序数										有效波高 m	有效波周期 s	最大波高 m	最大波周期 s	波级
			1	2	3	4	5	6	7	8	9	10					
		波高															
		周期															
		波高															
		周期															
备注																	

观测者　　计算者　　校对者

表 D.2 海水透明度、水色和海发光观测记录表

调查海区	断面号	观测日期
调查船	航次号	年 月 日至 年 月 日

序号	站号	站位						观测时间		水深 m	透明度 m	水色（级）	发光类型	发光等级（级）	海况（级）	有无星月或降水
		纬度 N/S			经度 E/W											
		(°)	(′)	(″)	(°)	(′)	(″)	时	分							
备注																

观测者　　计算者　　校对者

表 D.3 浮冰观测记录表

调查海区	断面号	观测日期
调查船	航次号	年 月 日至 年 月 日

站号	站位						观测时间		海面能见度 km	气温 ℃	风速 m/s	风向 (°)	天气现象	冰量 1/10	密集度 1/10	冰型	表面特征	冰状	冰厚 cm
	纬度 N/S			经度 E/W			时	分											
	(°)	(′)	(″)	(°)	(′)	(″)													

漂流速度和方向								冰区边缘线特征点							
起点		终点		移动距离 m	时间间隔 s	速度 m/s	方向 (°)	1		2		3		4	
方向 (°)	距离 m	方向 (°)	距离 m					方向 (°)	距离 m	方向 (°)	距离 m	方向 (°)	距离 m	方向 (°)	距离 m

备注	

观测者 计算者 校对者

表 D.4 固定冰观测记录表

调查海区	断面号	观测日期
调查船	航次号	年 月 日至 年 月 日

站号	站位						观测时间		海面能见度 km	气温 ℃	风速 m/s	风向 (°)	天气现象	冰型	冰厚 cm	冰界特征点							
	纬度 N/S			经度 E/W												1		2		3		4	
	(°)	(′)	(″)	(°)	(′)	(″)	时	分								方向 (°)	距离 m	方向 (°)	距离 m	方向 (°)	距离 m	方向 (°)	距离 m

备注	

观测者 计算者 校对者

表 D.5 冰山观测记录表

<table>
<tr><td colspan="24">调查海区 断面号 观测日期
调查船 航次号 年 月 日至 年 月 日</td></tr>
<tr><td colspan="6">船 位</td><td colspan="2" rowspan="2">观测时间</td><td colspan="6">冰山位置</td><td rowspan="3">冰山高度
m</td><td rowspan="3">冰山形状</td><td colspan="8">漂流方向和速度</td></tr>
<tr><td colspan="3">纬度
N/S</td><td colspan="3">经度
E/W</td><td rowspan="2">方向
(°)</td><td rowspan="2">距离
m</td><td colspan="2">纬度</td><td colspan="2">经度</td><td colspan="2">起点</td><td colspan="2">终点</td><td rowspan="2">移动距离
m</td><td rowspan="2">时间间隔
s</td><td rowspan="2">方向
(°)</td><td rowspan="2">速度
m/s</td></tr>
<tr><td>(°)</td><td>(′)</td><td>(″)</td><td>(°)</td><td>(′)</td><td>(″)</td><td>时</td><td>分</td><td>(°)</td><td>(′)</td><td>(°)</td><td>(′)</td><td>方向
(°)</td><td>距离
m</td><td>方向
(°)</td><td>距离
m</td></tr>
<tr><td></td><td></td><td></td><td></td><td></td><td></td><td></td><td></td><td></td><td></td><td></td><td></td><td></td><td></td><td></td><td></td><td></td><td></td><td></td><td></td><td></td><td></td><td></td><td></td></tr>
<tr><td></td><td></td><td></td><td></td><td></td><td></td><td></td><td></td><td></td><td></td><td></td><td></td><td></td><td></td><td></td><td></td><td></td><td></td><td></td><td></td><td></td><td></td><td></td><td></td></tr>
<tr><td></td><td></td><td></td><td></td><td></td><td></td><td></td><td></td><td></td><td></td><td></td><td></td><td></td><td></td><td></td><td></td><td></td><td></td><td></td><td></td><td></td><td></td><td></td><td></td></tr>
<tr><td colspan="3">备注</td><td colspan="21"></td></tr>
</table>

观测者 计算者 校对者

表 D.6.1 颠倒温度表测温记录表(Ⅰ)

<table>
<tr><td colspan="18">调查海区 调查船 站号 站位：纬度
调查机构 航次号 准确度等级 经度
观测日期 年 月 日 颠倒时间</td></tr>
<tr><td rowspan="2">采水器号</td><td rowspan="2">温度表号</td><td rowspan="2">V₀
℃</td><td colspan="2">读数Ⅰ</td><td colspan="10">读 数 Ⅱ</td><td rowspan="2">水温
T_W
℃</td><td rowspan="2">深度订正值
m</td><td rowspan="2">实际深度
m</td></tr>
<tr><td>副温
℃</td><td>主温
℃</td><td>副温
℃</td><td>器差
℃</td><td>t
℃</td><td>主温
℃</td><td>器差
℃</td><td>T
℃</td><td>T+V₀
℃</td><td>T−t
℃</td><td>K
℃</td><td>T+K
℃</td></tr>
<tr><td></td><td></td><td></td><td></td><td></td><td></td><td></td><td></td><td></td><td></td><td></td><td></td><td></td><td></td><td></td><td></td><td></td><td></td></tr>
<tr><td></td><td></td><td></td><td></td><td></td><td></td><td></td><td></td><td></td><td></td><td></td><td></td><td></td><td></td><td></td><td></td><td></td><td></td></tr>
<tr><td></td><td></td><td></td><td></td><td></td><td></td><td></td><td></td><td></td><td></td><td></td><td></td><td></td><td></td><td></td><td></td><td></td><td></td></tr>
<tr><td></td><td></td><td></td><td></td><td></td><td></td><td></td><td></td><td></td><td></td><td></td><td></td><td></td><td></td><td></td><td></td><td></td><td></td></tr>
<tr><td></td><td></td><td></td><td></td><td></td><td></td><td></td><td></td><td></td><td></td><td></td><td></td><td></td><td></td><td></td><td></td><td></td><td></td></tr>
<tr><td></td><td></td><td></td><td></td><td></td><td></td><td></td><td></td><td></td><td></td><td></td><td></td><td></td><td></td><td></td><td></td><td></td><td></td></tr>
<tr><td colspan="3">备 注</td><td colspan="15"></td></tr>
</table>

观测者 计算者 校对者

表 D.6.2 颠倒温度表测温记录表(Ⅱ)

调查海区　　调查船　　站　号　　站位：纬度
调查机构　　航次号　　准确度等级　　站位：经度
观测日期　　年　月　日　　颠倒时间

采水器号	温度表号	V_0 (V_0') ℃	读数Ⅰ 副温 ℃	读数Ⅰ 主温 ℃	读数Ⅱ 副温 ℃	读数Ⅱ 器差 ℃	读数Ⅱ t (t') ℃	读数Ⅱ 主温 ℃	读数Ⅱ 器差 ℃	读数Ⅱ T (T') ℃	读数Ⅱ $T+V_0$ ($T'+V_0'$) ℃	读数Ⅱ $T-t$ (T_W-t') ℃	读数Ⅱ K (k) ℃	读数Ⅱ $T+K$ ($T'+K$) ℃	T_W (T_u) ℃	$\frac{T_u-T_W}{\rho_m}$ Q	计算深度 m
备　注																	

观测者　　计算者　　校对者

表 D.7 盐度分析记录表

调查海区　　调查船　　站　号　　站位：纬度(N/S)　°　′　″
调查机构　　航次号　　准确度等级　　站位：经度(E/W)　°　′　″
采水日期　　年　月　日　　分析日期　　年　月　日

实测水层	采水时间 时	采水时间 分	瓶号	水浴温度 ℃	电导比 (R_T)	盐度未修正值 (S_0)	盐度修正值 (ΔS)	实用盐度 ($S=S_0+\Delta S$)	定　标	备注
									时间<br>$S=$　　$\times 10^{-3}$<br>$R_{15}=$<br>水温 $T=$　　℃<br>定标值	

观测者　　计算者　　校对者

表 D.8 CTD 观测记录表

调查船__________ 海区__________ 航次号__________

水深__________ 仪器型号__________ 探头号__________

观测日期		现场工作情况
站　号		
纬　度		
经　度		
取样间隔		
入水时间		
出水时间		
下放速度		
电池电压		
海　况		

CTD 采水记录								
采水器号	层次	压力<br>MPa	温度<br>℃	电导率	盐度	采水瓶号	盐度计值	备　注

观测者　　　　计算者　　　　校对者

表 D.9 漂流浮标记录表

海区__________ 调查船__________

航次号__________ 水　深__________ 漂流浮标器号____________

开机时间	日　期		现场情况记录
	北京时间		
	GMT		
投放时间	日　期		
	北京时间		
	GMT		
投放位置	经　度		
	纬　度		
海面温度			
海　况			
风　速			
风　向			

观测者　　　　计算者　　　　校对者

表 D.10 船只锚碇测流记录表

调查船＿＿＿＿ 海区＿＿＿＿ 磁偏差＿＿＿＿ 仪器型号＿＿＿＿
站　号＿＿＿＿ 经度＿＿＿＿ 纬　度＿＿＿＿ 水　深＿＿＿＿

<table>
<tr><td>观测日期</td><td colspan="2"></td><td>仪器工作情况</td></tr>
<tr><td rowspan="2">投放位置</td><td>经　度</td><td></td><td rowspan="8"></td></tr>
<tr><td>纬　度</td><td></td></tr>
<tr><td>直读/自记</td><td colspan="2"></td></tr>
<tr><td>入水时间</td><td colspan="2"></td></tr>
<tr><td>出水时间</td><td colspan="2"></td></tr>
<tr><td>海　况</td><td colspan="2"></td></tr>
<tr><td>风　速</td><td colspan="2"></td></tr>
<tr><td>风　向</td><td colspan="2"></td></tr>
</table>

观测者　　计算者　　校对者

表 D.11 声学多普勒测流记录表

调查船＿＿＿＿ 海区＿＿＿＿ 磁偏差＿＿＿＿ 仪器型号＿＿＿＿
站　号＿＿＿＿ 经度＿＿＿＿ 纬　度＿＿＿＿ 水　深＿＿＿＿

<table>
<tr><td>文件名</td><td></td><td>仪器工作情况</td></tr>
<tr><td>层数</td><td></td><td rowspan="13"></td></tr>
<tr><td>层厚 m</td><td></td></tr>
<tr><td>平均间隔</td><td></td></tr>
<tr><td>输入盐度</td><td></td></tr>
<tr><td>输入温度</td><td></td></tr>
<tr><td></td><td></td></tr>
<tr><td>开始 Ping 日期</td><td></td></tr>
<tr><td>开始 Ping 时间</td><td></td></tr>
<tr><td>直读/自记</td><td></td></tr>
<tr><td>入水时间</td><td></td></tr>
<tr><td>出水时间</td><td></td></tr>
<tr><td></td><td></td></tr>
<tr><td></td><td></td></tr>
</table>

观测者　　计算者　　校对者
