# Supplementary material for: Autumn ichthyoplankton assemblage in the Yangtze Estuary shaped by environmental factors
Source: PeerJ. 2016 Apr 19;4:e1922. doi: 10.7717/peerj.1922 (PMC4841249; doi:10.7717/peerj.1922)
Supplement: Supplemental Information 1 [file peerj-04-1922-s001.zip › GB12763-2007/GB¿MT 12763.4-2007 ║ú╤≤╡≈▓Θ╣μ╖╢ ╡┌04▓┐╖╓ú║║ú╦«╗»╤o╥¬╦╪╡≈▓Θ .pdf]

ICS 07.060
A 45

中华人民共和国国家标准

GB/T 12763.4—2007
代替 GB/T 12763.4—1991

海洋调查规范
第4部分:海水化学要素调查

Specifications for oceanographic survey—
Part 4:Survey of chemical parameters in sea water

2007-08-13 发布　　　　2008-02-01 实施

中华人民共和国国家质量监督检验检疫总局
中国国家标准化管理委员会　发布

目　　次

前言 …… V
1　范围 …… 1
2　规范性引用文件 …… 1
3　术语和定义 …… 1
4　一般规定 …… 3
4.1　调查方案设计的原则要求 …… 3
4.2　调查计划及其组织实施 …… 4
4.3　质量保证与分析质量控制 …… 5
4.4　样品采集与贮存 …… 6
4.5　资料处理的一般要求 …… 7
4.6　调查报告编写 …… 7
4.7　资料归档 …… 7
5　溶解氧测定(碘量滴定法) …… 7
5.1　技术指标 …… 7
5.2　方法原理 …… 8
5.3　试剂及其配制 …… 8
5.4　仪器与设备 …… 8
5.5　水样装取、预处理和贮存 …… 8
5.6　测定步骤 …… 8
5.7　计算 …… 9
5.8　仲裁方法 …… 10
6　pH 测定(pH 计法) …… 10
6.1　技术指标 …… 10
6.2　方法原理 …… 10
6.3　试剂及其配制 …… 10
6.4　仪器与设备 …… 11
6.5　水样装取、预处理和贮存 …… 11
6.6　测定步骤 …… 11
6.7　计算 …… 11
7　总碱度测定(pH 法) …… 12
7.1　技术指标 …… 12
7.2　方法原理 …… 12
7.3　试剂及其配制 …… 12
7.4　仪器与设备 …… 12
7.5　水样装取、预处理与贮存 …… 13
7.6　测定步骤 …… 13
7.7　计算 …… 13

8 活性硅酸盐测定(硅钼蓝法) …… 13
8.1 技术指标 …… 13
8.2 方法原理 …… 13
8.3 试剂及其配制 …… 13
8.4 仪器与设备 …… 14
8.5 水样装取、预处理和贮存 …… 14
8.6 测定步骤 …… 14
8.7 计算 …… 15
8.8 仲裁方法 …… 15
9 活性磷酸盐测定(抗坏血酸还原磷钼蓝法) …… 16
9.1 技术指标 …… 16
9.2 方法原理 …… 16
9.3 试剂及其配制 …… 16
9.4 仪器与设备 …… 16
9.5 水样装取、预处理和贮存 …… 17
9.6 测定步骤 …… 17
9.7 计算 …… 17
10 亚硝酸盐测定(重氮-偶氮法) …… 17
10.1 技术指标 …… 17
10.2 方法原理 …… 18
10.3 试剂及其配制 …… 18
10.4 仪器与设备 …… 18
10.5 水样装取、预处理和贮存 …… 18
10.6 测定步骤 …… 18
10.7 计算 …… 19
11 硝酸盐测定(锌镉还原法) …… 19
11.1 技术指标 …… 19
11.2 方法原理 …… 19
11.3 试剂及其配制 …… 19
11.4 仪器与设备 …… 20
11.5 水样装取、预处理和贮存 …… 20
11.6 测定步骤 …… 20
11.7 计算 …… 21
11.8 仲裁方法 …… 21
12 铵盐测定(次溴酸钠氧化法) …… 21
12.1 技术指标 …… 21
12.2 方法原理 …… 21
12.3 试剂及其配制 …… 21
12.4 仪器与设备 …… 22
12.5 水样装取、预处理和贮存 …… 22
12.6 测定步骤 …… 22
12.7 计算 …… 23
12.8 仲裁方法 …… 23

13 氯化物测定(银量滴定法) …… 23
13.1 技术指标 …… 23
13.2 方法原理 …… 23
13.3 试剂及其配制 …… 23
13.4 仪器与设备 …… 24
13.5 水样装取、预处理和贮存 …… 24
13.6 测定步骤 …… 24
13.7 计算 …… 24
14 总磷测定(过硫酸钾氧化法) …… 25
14.1 技术指标 …… 25
14.2 方法原理 …… 25
14.3 试剂及其配制 …… 25
14.4 仪器与设备 …… 25
14.5 水样装取、预处理和贮存 …… 25
14.6 测定步骤 …… 26
14.7 计算 …… 26
15 总氮测定(过硫酸钾氧化法) …… 26
15.1 技术指标 …… 26
15.2 方法原理 …… 27
15.3 试剂及其配制 …… 27
15.4 仪器与设备 …… 27
15.5 水样装取、预处理和贮存 …… 28
15.6 测定步骤 …… 28
15.7 计算 …… 28
附录 A (规范性附录) 活性硅酸盐测定(硅钼黄法) …… 30
A.1 技术指标 …… 30
A.2 方法原理 …… 30
A.3 试剂及其配制 …… 30
A.4 仪器与设备 …… 30
A.5 水样装取、预处理和贮存 …… 30
A.6 测定步骤 …… 30
A.7 计算 …… 31
附录 B (规范性附录) 硝酸盐测定(镉铜柱还原法) …… 32
B.1 技术指标 …… 32
B.2 方法原理 …… 32
B.3 试剂及其配制 …… 32
B.4 仪器与设备 …… 33
B.5 水样装取、预处理和贮存 …… 33
B.6 测定步骤 …… 33
B.7 计算 …… 34
附录 C (规范性附录) 铵盐测定(靛酚蓝法) …… 35
C.1 技术指标 …… 35
C.2 方法原理 …… 35

C.3 试剂及其配制 …… 35
C.4 仪器与设备 …… 36
C.5 水样装取、预处理和贮存 …… 36
C.6 测定步骤 …… 36
C.7 计算 …… 36
附录 D（规范性附录） 溶解氧测定（分光光度法） …… 38
D.1 技术指标 …… 38
D.2 方法原理 …… 38
D.3 试剂及其配制 …… 38
D.4 仪器与设备 …… 38
D.5 水样装取、预处理和贮存 …… 39
D.6 测定步骤 …… 39
D.7 计算 …… 40
D.8 求校准 K 值的方法 …… 40
附录 E（资料性附录） 记录表格式 …… 41
附录 F（资料性附录） 测定结果计算用表 …… 58

表 E.1 水样登记表 …… 41
表 E.2 溶解氧测定（碘量滴定法）记录表 …… 42
表 E.3 pH 测定记录表 …… 43
表 E.4 总碱度测定记录表 …… 44
表 E.5 （　　）标准曲线数据记录表 …… 45
表 E.6 活性硅酸盐测定记录表 …… 46
表 E.7 活性磷酸盐测定记录表 …… 47
表 E.8 亚硝酸盐测定记录表 …… 48
表 E.9 硝酸盐测定记录表 …… 49
表 E.10 铵盐测定（次溴酸钠氧化法）记录表 …… 50
表 E.11 氯化物测定记录表 …… 51
表 E.12 铵盐测定（靛酚蓝法）记录表 …… 52
表 E.13 溶解氧测定（分光光度法）记录表 …… 53
表 E.14 溶解氧测定（分光光度法）K 值记录表 …… 54
表 E.15 总磷测定记录表 …… 55
表 E.16 总氮测定记录表 …… 56
表 E.17 海水化学观测数据报表 …… 57
表 F.1 空气中氧在不同温度和盐度海水中的饱和浓度 …… 58
表 F.2 标准缓冲溶液的 pH 值 …… 62
表 F.3 pH 测定的温度校正值 $\alpha(t_m - t_w)$ 表 …… 62
表 F.4 pH 测定的压力校正系数 β 表 …… 63
表 F.5 pH—α_{H^+} 换算表 …… 63
表 F.6 海水氢离子活度系数 f_{H^+} 随盐度和 pH 变化 …… 63
表 F.7 元素的相对原子质量表(1997) …… 64
表 F.8 20℃时容积为 1.000 0 dm^3 玻璃容器中的蒸馏水在不同温度时的质量(m_{20}) …… 65

前言

GB/T 12763《海洋调查规范》分为11个部分：

——第1部分：总则；

——第2部分：海洋水文观测；

——第3部分：海洋气象观测；

——第4部分：海洋化学要素调查；

——第5部分：海洋声、光要素调查；

——第6部分：海洋生物调查；

——第7部分：海洋调查资料交换；

——第8部分：海洋地质地球物理调查；

——第9部分：海洋生态调查指南；

——第10部分：海底地形地貌调查；

——第11部分：海洋工程地质调查。

其中第9部分、第10部分和第11部分对应于GB/T 12763—1991是新增部分。

本部分为GB/T 12763的第4部分。

本部分代替GB/T 12763.4—1991《海洋调查规范　第4部分：海水化学要素观测》。

本部分与GB/T 12763.4—1991相比主要变化如下：

——按照GB/T 1.1—2000《标准化工作导则　第1部分：标准的结构和编写规则》规定，修改了标准的编排格式，主要有：将“篇”改为“章”；术语和定义独立成条；将各要素“测定方法”删去，内容经补充后，划分为“技术指标”、“方法原理”、“试剂及其配制”、“仪器与设备”、“水样装取、预处理和贮存”、“测定步骤”和“计算”等7个条目；

——将原“技术设计”修改为“调查方案设计的原则要求”(1991年版的3，本版的4.1)；

——将原“出海前的准备”修改为“调查计划及其组织实施”(1991年版的6，本版的4.2)；

——将原“测定过程的质量控制”修改为“质量保证与分析质量控制”(1991年版的5，本版的4.3)；

——将原“氨的测定”改称为“铵盐测定”(1991年版的第九篇，本版的12)；

——将原“氯度测定(银量滴定法)”改为“氯化物测定(银量滴定法)”(1991年版的第十篇，本版的13)；

——重新设计了“记录表格式”(见附录E)；

——增加了“总磷测定(过硫酸钾氧化法)”和“总氮测定(过硫酸钾氧化法)”等两种分析方法(本版的14、本版的15)；

——增加了“溶解氧测定(分光光度法)” 作为规范性附录编入(见附录D)；

——增加了“溶解氧测定(分光光度法)记录表”、“总磷测定记录表”、“溶解氧测定(分光光度法)K值记录表”、“总氮测定记录表”和“海水化学要素数据报表”等五种记录表格式作为资料性附录(见表E.13、表E.14、表E.15、表E.16和表E.17)。

本部分的附录A、附录B、附录C和附录D为规范性附录，附录E和附录F为资料性附录。

本部分由国家海洋局提出。

本部分由国家海洋标准计量中心归口。

本部分起草单位：国家海洋局第三海洋研究所负责起草，国家海洋局第二海洋研究所、国家海洋局海洋环境监测中心、国家海洋局南海分局、国家海洋局东海分局等参加起草。

本部分主要起草人：暨卫东、许昆灿、吴省三、邹汉阳、林建云、廖文卓、张元标。

本部分所代替标准的历次版本发布情况为：

——GB/T 12763.4—1991。

海洋调查规范
第4部分:海水化学要素调查

1 范围

GB/T 12763 的本部分规定了海水化学要素调查的方案设计、调查计划的组织实施、样品采集与贮存、测定方法、分析质量保证和数据处理。

本部分适用于海洋调查的海水化学要素调查。

2 规范性引用文件

下列文件中的条款通过 GB/T 12763 的本部分的引用而成为本部分的条款。凡是注日期的引用文件，其随后所有的修改单(不包括勘误的内容)或修订版均不适用于本部分，然而，鼓励根据本部分达成协议的各方研究是否可使用这些文件的最新版本。凡是不注明日期的引用文件，其最新版本适用于本部分。

GB/T 8170 数值修约规则

GB/T 12763.1 海洋调查规范 第1部分:总则

GB/T 12763.2 海洋调查规范 第2部分:海洋水文观测

GB/T 12763.7 海洋调查规范 第7部分:海洋调查资料交换

3 术语和定义

下列术语和定义适用于 GB/T 12763 的本部分。

3.1

溶解氧 dissolved oxygen

DO

溶解在海水中的氧气。

注1：单位为微摩尔每立方分米(μmol/dm^3)氧原子。

注2：改写 GB/T 15925，定义 1.2.20。

3.2

溶解氧饱和浓度 saturation concentration of dissolved oxygen

在任何给定的水温和盐度条件下，氧在海水中溶解至饱和时的特定浓度。

3.3

溶解氧饱和度 saturability of dissolved oxygen

测得的溶解氧浓度与水样现场水温、盐度条件下的溶解氧饱和浓度之百分比。

注：改写 GB/T 15925，定义 1.2.22。

3.4

pH

海水中氢离子活度的负对数，即 $pH = -\lg[\alpha_{H^+}]$。

3.5

总碱度 alkalinity

A

中和单位体积海水中弱酸阴离子所需氢离子的量。

注1：单位为毫摩尔每立方分米(mmol/dm^3)。

注2：改写 GB/T 15925，定义 1.2.9。

3.6

活性硅酸盐　reactive silicate

SiO_3^{2-}-Si

能被硅质生物摄取的溶解态正硅酸盐和它的二聚物。

注 1：单位为微摩尔每立方分米（μmol/dm³）硅原子。

注 2：改写 GB/T 15925，定义 1.2.28。

3.7

活性磷酸盐　reactive phosphate

PO_4^{3-}-P

能被浮游植物摄取的正磷酸盐。

注 1：单位为微摩尔每立方分米（μmol/dm³）磷原子。

注 2：改写 GB/T 15925，定义 1.2.29。

3.8

亚硝酸盐　nitrite

NO_2^--N

能被浮游植物摄取的亚硝酸盐。

注：单位为微摩尔每立方分米（μmol/dm³）氮原子。

3.9

硝酸盐　nitrate

NO_3^--N

能被浮游植物摄取的硝酸盐。

注：单位为微摩尔每立方分米（μmol/dm³）氮原子。

3.10

铵　ammonium

NH_4^+-N

能被浮游植物摄取的铵盐。

注：单位为微摩尔每立方分米（μmol/dm³）氮原子。

3.11

氯化物　chloride

Cl^-

溶解于海水中的无机氯化物。

注：单位为克每立方分米（g/dm³）氯离子。

3.12

总磷　total phosphorus

TP-P

海水中溶解态和颗粒态的有机磷和无机磷化合物的总和。

注：单位为微摩尔每立方分米（μmol/dm³）磷原子。

3.13

总氮　total nitrogen

TN-N

海水中溶解态和颗粒态的有机氮和无机氮化合物的总和。

注：单位为微摩尔每立方分米（μmol/dm³）氮原子。

4 一般规定

4.1 调查方案设计的原则要求

4.1.1 设计原则

调查工作开始之前，应进行技术方案设计。

根据任务书或合同书要求，遵循有限目标，经济效能，技术先进性与现实条件可能性相结合，并能与历史上调查计划相衔接的原则。

4.1.2 前期准备工作

收集与分析调查海区已有的水文气象、地质地貌、生物和海水化学资料。

4.1.3 目标与任务

4.1.3.1 确定的调查目标与任务应是明确的、具体的和可以衡量的。

4.1.3.2 应说明实现目标的途径与关键技术。

4.1.4 采样设计

4.1.4.1 站位布设

站位布设应考虑以下因素：调查目的、调查海区的地理位置、地形、水动力条件、物质来源、人力物力资源和采样的可能条件。调查站位一般可采用网格式布站，并选定若干横向和纵向断面布站。沿岸与近海区也可采用沿流系轴向和穿越流系、水团方向布站。穿越流系、水团断面，应与陆岸垂直，或是近似发散型的。在水文或水化学条件变化剧烈的区域，应适当加密站位。每一调查区，应选取若干个有代表性站位作为定点观测站。在保证获取所需信息的前提下，尽量减少站位数。

4.1.4.2 调查层次

4.1.4.2.1 河口、港湾、近海和洋区调查，一般可分别按表1设置采样层次。表层、底层位置和钢丝绳倾角订正按GB/T 12763.2的规定执行。

表1 采样层次

单位为米

水深范围	层　　次
≤50	表层、5、10、20、30、底层
＞50	表层、10、20、30、50、75、100、150、200、300、400、500、600、800、1 000、1 200、1 500、2 000、2 500、3 000、……以下<br>每1 000 m加一层、底层。

4.1.4.2.2 断面观察站应采集全部层次水样；非断面观察站，则可根据需要只采集水面至某一深度水样。

4.1.4.2.3 对水文、水化学等条件剧烈变化的水层，必要时，可加密采样层次。

4.1.4.3 调查时间与次数

调查时间和次数应根据水环境条件和特定调查目的确定：

a) 对水体相对稳定的洋区，一年中应在环境特征典型的季节调查一次。

b) 对受气象、流系季节影响显著的近海、边缘海，在一年中，至少应在环境特征显著差异的冬、夏两个季节，各调查一次；在人力、物力许可时，也可在春、秋两季，各增加一次调查。

c) 对沿岸、河口等受气候、水文和物质来源影响的海区，一般情况应每季度调查一次，且采样时间应充分考虑潮汐影响。若欲获取更详实的资料，则应每月调查一次。

d) 当需要进行周日观测时，一般每2 h观测一次，一周日共13次。至少应每3 h观测一次，一周日共九次。

4.1.5 调查项目与分析方法

4.1.5.1 根据海洋调查的具体需要确定调查项目。常规调查要素一般包括pH、溶解氧及其饱和度、

总碱度、活性硅酸盐、活性磷酸盐、硝酸盐、亚硝酸盐、铵盐。

4.1.5.2 海水化学调查项目可按两类选定，一类为基本调查要素，即所有采集样品必须测定的要素；另一类为辅助要素，即仅测定某些航次、站位或层次样品的要素。

4.1.5.3 营养盐测定可采用营养盐自动分析方法，溶解氧测定可采用溶解氧探头测定法。

4.1.6 成果

根据调查目的提出预期调查成果。调查成果最基本的内容应包括数据汇总报表和调查报告，较深入的海洋调查还应包括图集和研究报告。

4.1.7 风险分析

对较大规模的海水化学要素调查，应进行风险分析，指出可能遇到的困难，和拟采用的应对措施。

4.2 调查计划及其组织实施

4.2.1 调查计划

调查计划包括的主要内容：

a) 目的与任务；
b) 调查项目与分析方法；
c) 站点布设(附站位图及经纬度)，采样计划(包括各站位采样项目、层次和各项目的样品数)；
d) 航次时间安排、航次顺序和补给地点；
e) 调查人员组织、分工与协调；
f) 质量计划；
g) 计划执行期限与进度安排；
h) 经费预算；
i) 预期成果及其提供形式；
j) 风险预测与防范措施。

4.2.2 调查计划报批

由任务承担单位将调查计划呈报任务委托方。经委托方批复核准并下达任务书。调查计划执行过程中，若遇特殊情况须改变计划时，应及时呈报委托方批准。

4.2.3 组建调查队

按 GB/T 12763.1 的有关规定组建调查队，并填写《海洋调查人员登记表》(见 GB/T 12763.1 的附录 B)，报任务委托方核准备案。

4.2.4 航前准备

4.2.4.1 按照调查计划列出海上作业所需器材、物资的清单(包括船上分析的仪器、采样设备、样品瓶、水样预处理和贮存器皿、相关试剂与标准溶液及分析记录表等相关物资名称与数量)。

4.2.4.2 按规范要求清洗采样设备、样品瓶和器皿。

4.2.4.3 对各调查项目的样品瓶进行编号；溶解氧水样品瓶还应附上相应瓶号的容积数据表。

4.2.4.4 按船上分析项目需要，制备纯水、配制试剂溶液和标准溶液，并按分析样品数量备足。

4.2.4.5 准备采样记录表、船上分析的标准曲线记录表和测定记录表。

4.2.4.6 安装船上采水设备并调试；安装、固定、调试和校准船用分析仪；安装、固定其他调查物品。

4.2.4.7 将备航的器材、试剂及其他物资分类清点装箱，并附上装箱单。

4.2.4.8 出海前，应对备航工作进行一次全面检查。

4.2.5 海上作业

4.2.5.1 海上作业由项目负责人或首席科学家指挥，并与调查船船长协调船舶航行。

4.2.5.2 船舶到站前，应停止排污、清洗甲板，关闭洗手间，直至该站采样作业结束。

4.2.5.3 按调查计划和分样程序采集水样，严防水样沾污。按记录书写要求，填写采样记录。

4.2.5.4 站位采样结束时，应进行一次检查，确认采样无遗漏时方可启航。

4.2.5.5 在规定时间内，完成水样过滤和现场测定项目的测定工作，并按要求填写分析记录表。

4.2.5.6 航行结束时，应将运回陆地实验室水样贮存装箱；清洗、整理调查设备与仪器并装箱，附上装箱单，并提交航次调查报告。

4.3 质量保证与分析质量控制

4.3.1 建立质量监督管理体系

根据调查项目的具体情况，建立质量监督管理体系，确定负责人，明确其权限与职责。

4.3.2 确定质量保证的目标

根据调查计划的目的，确定每一要素分析结果的准确度要求，以此作为该要素的质量目标。

4.3.3 明确质量保证的任务和内容

4.3.3.1 质量保证的任务是根据各要素的质量目标和误差来源，采取相适应的质量控制措施，将各要素调查数据的误差减少到所需水平。

4.3.3.2 质量保证的内容主要包括：仪器设备检定和调查技术人员的业务培训；现场与陆地实验室的科学管理；采样与分析全过程（包括从取样至分析结果计算）的质量控制与质量评价；数据、资料和成果的质量控制。

4.3.4 采样与样品预处理的质量控制

4.3.4.1 采样时，应严禁船舶排污；采样位置应远离船舶排污口。

4.3.4.2 恶劣天气（如热带风暴等）可能危及作业人员安全时，应停止采样。

4.3.4.3 严格按规定程序和操作要求进行采样、分样和样品预处理（见4.4）。

4.3.4.4 分样与样品预处理的工作台应远离洗手间。

4.3.4.5 水样过滤膜不能重复使用。

4.3.4.6 应按样品规定的贮存条件贮存样品。

4.3.5 化学试剂

4.3.5.1 用于海洋调查的化学试剂，应符合GB/T 12763.1的有关规定。

4.3.5.2 化学试剂应按规定条件配制成溶液，并应在规定条件下保存、在规定期限内使用。

4.3.5.3 自配的标准溶液，应用具有保证值的国家标准溶液校准合格后，方可使用。

4.3.5.4 试剂空白值应低至与分析方法检出限同一水平，若明显超过此量值，应检查原因，并对产生高试剂空白值的主要试剂再作纯化处理，或选用新批号或其他厂家生产的试剂。若试剂空白值确实难于降低的，则加入的试剂量应准确。在分析过程中，应平行测定分析空白，以监视其空白值变化。

4.3.6 器皿

4.3.6.1 用于海洋调查的器皿，应符合GB/T 12763.1的有关规定。

4.3.6.2 按照各要素分析对所使用器皿材质的要求，合理选择分析过程所需各种器皿的材质。

4.3.6.3 器皿洗涤应采用恰当的洗涤方法。洗涤剂不应含有被测成分。

4.3.7 实验室环境

船上或陆地的实验室均应符合GB/T 12763.1的有关规定。

4.3.8 仪器

用于海洋调查的分析仪器，包括天平与砝码、容量仪器、pH计和分光光度计，均应符合GB/T 12763.1的有关规定。

4.3.9 原始记录与样品采集记录

4.3.9.1 原始调查数据的记录与保存应按GB/T 12763.1的有关规定执行，原始记录表的格式规定参见附录E。

4.3.9.2 样品采集记录按GB/T 12763.1的有关规定执行。样品采集记录表格式参见表E.1。

4.3.10 实验室内质量评价

4.3.10.1 初次参加海洋调查的分析人员，应对其应用的分析方法进行验证，熟练掌握分析测定步骤。

验证内容包括工作曲线的线性范围、斜率，方法的精密度、准确度以及分析空白值的稳定性。其验证结果应与规范要求一致。

4.3.10.2 每一航次或每隔一定时间，应采用人工配制水样或天然水样，进行平行测定，以控制偶然误差。

4.3.10.3 每一航次或每隔一定时间，应采用标准物质配制水样检查测定结果是否存在系统误差。若确实存在系统误差，应寻找原因并加以改进。

4.3.10.4 对多个实验室合作的海洋调查，在调查计划执行前，应进行实验室间分析比测，判断实验室间分析结果的一致性。

4.4 样品采集与贮存

4.4.1 采水器材质的要求

根据各调查要素分析所需水样量和对采水器材质的要求，选择合适容积和材质的采水器，并洗净。

4.4.2 分装水样

水样采上船甲板后，先填好水样登记表(参见表 E.1)，并核对瓶号。然后，立即按以下分样顺序分装水样：溶解氧、pH、总碱度与氯化物、五项营养盐、总磷与总氮。

4.4.3 样品分装与贮存

4.4.3.1 溶解氧

a) 碘量滴定法：

水样瓶容积约为 120 cm^3(事先测定容积准确至 0.1 cm^3)的棕色磨口硬质玻璃瓶，瓶塞应为斜平底。

装取方法与贮存：将乳胶管的一端接上玻璃管，另一端套在采水器的出水口，放出少量水样洗涤水样瓶二次。然后，将玻璃管插到水样瓶底部，慢慢注入水样，并使玻璃管口始终处于水面下，待水样装满并溢出水样瓶体积的 1/2 时，将玻璃管慢慢抽出，瓶内不可有气泡。每一水样装取 2 瓶。立即用自动加液器(管尖在紧靠液面下)依次注入 1.0 cm^3 氯化锰溶液(见 5.3.1)和 1.0 cm^3 碱性碘化钾溶液(见 5.3.2)，应注意此加液管外壁不可沾有碘试剂。加液后立刻塞紧瓶盖并用手压住瓶塞和瓶底，将水样瓶缓慢地上下翻转 20 次。将水样瓶浸泡于水中，有效保存时间为 24 h(对于受有机物污染严重的水样，则应立即滴定)。

b) 分光光度法：

将乳胶管的一端接上玻璃管，另一端套在采水器的出口，放出少量水样荡洗水样瓶(水样瓶为 60 cm^3棕色磨口玻璃瓶)二次。将玻璃管插到水样瓶底部，慢慢注入水样，待水样装满并溢出约为瓶子体积的 1/2 时，将玻璃管慢慢抽出，立即用自动加液器(注入口埋入液面下)依次注入 0.50 cm^3 氯化锰溶液(见 D.3.1)和 0.5 cm^3 碱性碘化钠/叠氮化钠溶液(见 D.3.2)。塞紧瓶塞，将瓶子缓慢上下颠倒 20 次，将水样瓶浸泡于水中，有效保存时间为 24 h。

4.4.3.2 pH

a) 水样瓶为容积约 50 cm^3 具有双层盖的广口聚乙烯瓶。
b) 装取方法与贮存：用少量水样洗涤样品瓶二次，慢慢地将瓶子注满水样，立即旋紧瓶盖，存于阴暗处，放置时间不得超过 2 h。对于不能在 2 h 内测定的水样，应加入 1 滴氯化汞溶液(见 6.3.4)固定，旋紧瓶盖，混合均匀。有效保存时间为 24 h。

4.4.3.3 总碱度与氯化物

a) 水样瓶为容积约 250 cm^3 具塞、平底硬质玻璃瓶，或 200 cm^3 具有螺旋盖的广口聚乙烯瓶。使用前应用体积分数 1% 的盐酸浸泡 7 d，然后用蒸馏水彻底洗净，晾干。
b) 装取方法与贮存：用少量水样洗涤样品瓶二次，然后，装取水样约 100 cm^3(若欲测定氯化物，则应装取 200 cm^3 水样)，立即盖紧瓶塞。有效保存时间为 3 d。

4.4.3.4 **五项营养盐**

a) 硅酸盐、磷酸盐、硝酸盐、亚硝酸盐和铵盐水样合并装于同一个水样瓶中(铵的靛酚蓝测定法，样品单独分装于 200 cm^3 的具有两层盖的高密度聚乙烯瓶中，无须过滤处理)。

b) 水样瓶为容积约 500 cm^3 具有双层盖的高密度聚乙烯瓶。初次使用前，应用体积分数为 1% 的盐酸浸泡 7 d，然后洗涤干净，备用。

c) 滤膜：海水过滤滤膜为孔径 0.45 μm 的混合纤维素酯微孔滤膜。使用前应用体积分数为 1% 的盐酸浸泡 12 h，然后用蒸馏水洗至中性，浸泡于蒸馏水中，备用。每批滤膜经处理后，应对各要素作膜空白试验，确认滤膜符合要求后，空白值应低于各要素的检测下限方可使用。若任一要素的膜空白超过其检测下限时，应更换新批号滤膜。

d) 装取方法与贮存：用少量水样荡洗水样瓶二次，然后，装取约 500 cm^3 水样。立即用处理过的滤膜过滤于另一个 500 cm^3 水样瓶中。若需保存，应加入占水样体积千分之二的三氯甲烷(警告—剧毒，小心操作!)，盖好瓶塞，剧烈振摇 1 min，放在冰箱或冰桶内于 4℃～6℃低温保存，有效保存时间为 24 h。未经三氯甲烷固定和冷藏的水样，应在采样后 2 h 内测定。

4.4.3.5 **总磷、总氮**

取 500 cm^3 海水水样于聚乙烯瓶中，加入 1.0 cm^3 体积分数为 50%的硫酸溶液，混匀，旋紧瓶盖贮存，有效保存时间为一个月。

50%硫酸溶液的配制：在水浴冷却和不断搅拌下，将 250 cm^3 浓硫酸(H_2SO_4，ρ= 1.84 g/cm^3)缓慢加入 250 cm^3 蒸馏水中配制。

4.5 资料处理的一般要求

4.5.1 数据报表

按船、航次汇总数据，填写数据报表(参见表 E.17)。

4.5.2 数据处理

数据处理应符合 GB/T 12763.1 的有关规定。数据处理的计算用表参见附录 F。

4.5.3 有效数字

4.5.3.1 数据的有效数字位数应真实反映其可达到的精度，既不可增加位数，也不要减少位数。

4.5.3.2 一组数据运算结果的有效数字位数应严格按照有效数字运算规则确定。

4.5.3.3 数值修约按 GB/T 8170 的规定执行。

4.5.4 数据检验

4.5.4.1 数据统计之前，首先应检查调查数据是否存在计算、记录或誊写的失误，若属这类失误数据则应改正或直接剔除。

4.5.4.2 数据的检验、异常值判别与处理按 GB/T 12763.7 的有关规定执行。

4.5.5 资料格式

数据文档、图件和声像资料格式应符合 GB/T 12763.7 的有关规定。

4.6 调查报告编写

调查资料获取后，应按合同任务书要求和 GB/T 12763.1 的有关规定编写调查报告。

4.7 资料归档

资料归档按 GB/T 12763.1 和 GB/T 12763.7 的有关规定执行。

5 溶解氧测定(碘量滴定法)

5.1 技术指标

测定范围：5.3 $\mu mol/dm^3$～1.0×10^3 $\mu mol/dm^3$。

检测下限：5.3 $\mu mol/dm^3$。

精密度：含量低于 160 $\mu mol/dm^3$ 时，标准偏差为±2.8 $\mu mol/dm^3$；含量大于或等于 550 $\mu mol/dm^3$

时，标准偏差为±4.0 μmol/dm³。

5.2 方法原理

当水样加入氯化锰和碱性碘化钾试剂后，生成的氢氧化锰被水中溶解氧氧化生成 $MnO(OH)_2$ 褐色沉淀。加硫酸酸化后，沉淀溶解。用硫代硫酸钠标准溶液滴定析出的碘，换算溶解氧含量。

5.3 试剂及其配制

除另有说明外，所用试剂均为分析纯，水为蒸馏水或等效纯水。

5.3.1 氯化锰溶液：$c(MnCl_2)$ = 2.4 mol/dm³

称取 480 g 氯化锰（$MnCl_2 \cdot 4H_2O$）溶于水中，并稀释至 1 000 cm³。

5.3.2 碱性碘化钾溶液：$c(NaOH)$ = 6.4 mol/dm³，$c(KI)$ = 1.8 mol/dm³

称取 256 g 氢氧化钠（NaOH）溶解于 300 cm³ 水中，另称取 300 g 碘化钾（KI）溶解于 300 cm³ 水中，然后将上述二溶液混合，并稀释至 1 000 cm³。

5.3.3 硫酸溶液：体积分数为 25%

在搅拌和水浴的冷却下，将 1 体积的浓硫酸（H_2SO_4，ρ = 1.84 g/cm³）缓慢地加入于 3 体积的水中。

5.3.4 硫代硫酸钠溶液：$c(Na_2S_2O_3)$ = 1×10^4 μmol/dm³

称取 25.0 g 硫代硫酸钠（$Na_2S_2O_3 \cdot 5H_2O$），用少量水溶解后，稀释至 1 000 cm³，加入 1.0 g 无水碳酸钠（Na_2CO_3），混匀。贮于棕色试剂瓶中，此溶液浓度为 0.1 mol/dm³。放置 15 d 后，用刚煮沸冷却的水稀释成 0.01 mol/dm³ 的溶液。保存于棕色瓶中，使用前标定。

5.3.5 碘酸钾标准溶液：$c(1/6\ KIO_3)$ = 1.000×10^4 μmol/dm³

称取 3.567 g 碘酸钾（KIO_3，优级纯，预先在 120℃烘 2 h，置于硅胶干燥器中冷却至室温），溶于刚煮沸并冷却至室温水中，转移入 1 000 cm³ 量瓶中，稀释至标线，混匀，贮于棕色试剂瓶中。在 5℃～6℃低温保存，有效期三个月。使用时移取 10.00 cm³，用水稀释至 100 cm³，此溶液浓度为 1.000×10^4 μmol/dm³。

5.3.6 淀粉-丙三醇（甘油）指示剂

称取 3.0 g 可溶性淀粉[$(C_6H_{10}O_5)_n$]，加入 100 cm³ 丙三醇[$C_3H_5(OH)_3$]，搅拌并加热至 190℃至淀粉完全溶解。此溶液在常温下可保存一年，出现浑浊不影响指示剂的功效。

5.4 仪器与设备

5.4.1 水样瓶：容积约 120 cm³ 棕色磨口玻璃瓶，瓶塞为锥形或斜平底形，磨口要严密，每个水样瓶应按下述程序测定容积准确至 0.1 cm³：将水样瓶装满蒸馏水，塞上瓶塞、擦干，称重。减去干燥的空瓶重量，除以该水温时蒸馏水的密度（参见表 F.8），测得水样瓶容积。将瓶号及相应的水样瓶容积测量结果记录，备查。

5.4.2 溶解氧滴定管：25 cm³，分刻度值为 0.05 cm³。

5.4.3 电磁搅拌器：转速可调至 150 r/min～400 r/min。

5.4.4 磁转子（玻璃或聚四氟乙烯包裹）：直径约 3 mm～5 mm，长 25 mm。

5.4.5 定量加液器：1.0 cm³，5.0 cm³。

5.4.6 移液吸管：15.00 cm³。

5.5 水样装取、预处理和贮存

水样装取、预处理和贮存的操作和要求见 4.4.3.1。

5.6 测定步骤

5.6.1 硫代硫酸钠溶液的标定

用移液吸管吸取 15.00 cm³ 碘酸钾标准溶液（见 5.3.5），沿壁注入 250 cm³ 碘量瓶中，用少量水冲洗瓶内壁，加入 0.6 g 碘化钾，混匀。再加入 1.0 cm³ 硫酸溶液（见 5.3.3），再混匀，盖好瓶塞，在暗处放置 2 min。取下瓶塞，沿壁加入 110 cm³ 水，放入磁转子，置于电磁搅拌器上，立即开始搅拌并用硫代硫酸钠溶液（见 5.3.4）进行滴定，待试液呈淡黄色时加入三滴至四滴淀粉指示剂（见 5.3.6），继续滴至溶液蓝色刚消失。

重复标定至两次滴定管读数相差不超过 0.03 cm^3 为止。将滴定管读数记入溶解氧测定记录表(参见表 E.2)中,每隔 24 h 标定一次。

5.6.2 水样测定

水样固定(见 4.4.3.1)后,待沉淀物沉降聚集至瓶的下部,便可进行滴定。

将水样瓶上层清液倒出一部分于 250 cm^3 锥形烧瓶中,立即向沉淀中加入 1.0 cm^3 硫酸溶液(见 5.3.3),塞紧瓶塞,振荡水样瓶至沉淀全部溶解。

将水样瓶内溶液沿壁倾倒入上述锥形烧瓶中,将其置于电磁搅拌器上,立即搅拌,并滴定,待试液呈淡黄色时,加入三滴至四滴淀粉指示剂(见 5.3.6),继续滴定至呈淡蓝色。

用锥形烧瓶中的少量试液荡洗原水样瓶,再将其倒回锥形烧瓶中,继续滴定至无色。待 20 s 后,如试液不呈淡蓝色,即为终点。将滴定所消耗的硫代硫酸钠溶液体积记录于溶解氧测定记录表(参见表 E.2)中。

5.6.3 试剂空白试验

取 100 cm^3 海水,加入 1.0 cm^3 硫酸溶液(见 5.3.3),1.0 cm^3 碱性碘化钾溶液(见 5.3.2),混匀,加入 1.0 cm^3 氯化锰溶液(见 5.3.1),混合均匀,放置 10 min,加入三滴至四滴淀粉指示剂(见 5.3.6),混匀。此时,若溶液呈现淡蓝色,继续用硫代硫酸钠溶液(见 5.3.4)滴定。如硫代硫酸钠用量超出 0.1 cm^3,则应核查碘化钾和氯化锰试剂的可靠性并重新配制试剂。如果硫代硫酸钠用量小于或等于 0.1 cm^3,或加入淀粉指示剂后溶液不呈现淡蓝色,且加入一滴碘酸钾溶液(见 5.3.5)后,溶液立即呈现蓝色,则试剂空白可以忽略不计。

每批新配制试剂应进行一次空白试验。

5.7 计算

5.7.1 海水中溶解氧浓度计算见式(1)。

$$c(\mathrm{O}) = \frac{c \times V}{(V_1 - V_2) \times 2} \qquad \cdots\cdots(1)$$

式中:

$c(\mathrm{O})$——海水中溶解氧浓度,单位为微摩尔每立方分米($\mu mol/dm^3$);

V——滴定样品时消耗的硫代硫酸钠溶液体积,单位为立方厘米(cm^3);

c——硫代硫酸钠溶液标定浓度,单位为微摩尔每立方分米($\mu mol/dm^3$);

$V_1 - V_2$——实际水样的体积,单位为立方厘米(cm^3)。其中,V_1 为水样瓶的容积,单位为立方厘米(cm^3),V_2 为固定水样的固定剂体积,单位为立方厘米(cm^3)。

5.7.2 氧饱和度 $r(\mathrm{O})$ 的计算公式为式(2)。

$$r(\mathrm{O}) = \frac{c(\mathrm{O})}{c(\mathrm{O}')} \times 100\% \qquad \cdots\cdots(2)$$

式中:

$c(\mathrm{O})$——测得水样的氧浓度,单位为微摩尔每立方分米($\mu mol/dm^3$);

$c(\mathrm{O}')$——在现场水温、盐度下,氧在海水中的饱和浓度,单位为微摩尔每立方分米($\mu mol/dm^3$)(由表 F.1 查得或由 5.7.3 的公式(3)求得)。

5.7.3 氧在不同水温、盐度的海水中的饱和浓度计算公式为式(3)。

$$\ln c(\mathrm{O}') = A_1 + A_2(100/T) + A_3\ln(T/100) + A_4(T/100) + S[B_1 + B_2(T/100) + B_3(T/100)^2] + 0.4912 \qquad \cdots\cdots(3)$$

式中:

$c(\mathrm{O}')$——氧在海水中的饱和浓度,单位为微摩尔每立方分米($\mu mol/dm^3$);

T——现场的海水热力学温度,单位为开(K);

S——现场的海水盐度;

A、B——常数，其量值分别为：

$A_1=-173.4292$；

$A_2=249.6339$；

$A_3=143.3483$；

$A_4=-21.8492$；

$B_1=-0.033096$；

$B_2=0.014259$；

$B_3=-0.001700$。

5.8 仲裁方法

溶解氧测定（碘量滴定法）为仲裁方法。除本方法外，另有分光光度法（见附录 D）。

6 pH 测定（pH 计法）

6.1 技术指标

准确度：±0.02 pH。

精密度：±0.01 pH。

6.2 方法原理

海水的 pH 值是根据测定玻璃-甘汞电极对的电动势而得。将海水水样的 pH 与标准溶液的 pH 和该电池电动势的关系定义为：

$$pH_x=pH_s+(E_s-E_x)/(2.3026\,RT/F)$$

当玻璃-甘汞电极对插入标准缓冲溶液时：

令：

$$A=pH_s+\frac{E_s}{2.3026\,RT/F} \qquad \cdots\cdots(4)$$

当玻璃-甘汞电极对插入水样时，则：

$$pH_x=A-\frac{E_x}{2.3026\,RT/F} \qquad \cdots\cdots(5)$$

在同一温度下，分别测定同一电极对在标准缓冲溶液和水样中的电动势，则水样的 pH 值为：

$$pH_x=pH_s+\frac{E_s-E_x}{2.3026\,RT/F} \qquad \cdots\cdots(6)$$

式中：

pH_x——水样的 pH 值；

pH_s——标准缓冲溶液的 pH 值；

E_x——玻璃-甘汞电极对插入水样中的电动势，单位为毫伏（mV）；

E_s——玻璃-甘汞电极对插入标准缓冲溶液中的电动势，单位为毫伏（mV）；

R——气体常数；

F——法拉第常数；

T——热力学温度，单位为开（K）。

6.3 试剂及其配制

除另有说明外，所用试剂均为分析纯，水为蒸馏水或等效纯水。

6.3.1 磷酸二氢钾（KH_2PO_4）

置于 115℃±5℃烘箱中烘 2 h，于干燥器中冷却至室温。

6.3.2 磷酸氢二钠（Na_2HPO_4）

置于 115℃±5℃烘箱中烘 2 h，于干燥器中冷却至室温。

6.3.3　十水四硼酸钠($Na_2B_4O_7 \cdot 10H_2O$)

置于盛有蔗糖饱和溶液的干燥器中 48 h,并继续存于此干燥器中备用。

6.3.4　pH 标准缓冲溶液

6.3.4.1　0.025 mol/dm^3 磷酸二氢钾和 0.025 mol/dm^3 磷酸氢二钠混合标准缓冲溶液(25℃时,pH_s= 6.864)

称取 3.39 g 磷酸二氢钾(见 6.3.1.1)和 3.55 g 磷酸氢二钠(见 6.3.1.2)溶于水中并稀释至 1 000 cm^3,加 1.0 cm^3 三氯甲烷,混匀,保存于聚乙烯瓶中。使用期三个月。使用标准缓冲溶液时,应采用测定溶液温度下的标准 pH 值(参见表 F.2)。

6.3.4.2　四硼酸钠标准缓冲溶液:c ($Na_2B_4O_7 \cdot 10H_2O$)= 0.010 mol/dm^3(25℃时,pH_s= 9.182)

称取 3.80 g 十水四硼酸钠(见 6.3.1.3)溶于刚煮沸冷却的蒸馏水,并稀释至 1 000 cm^3,加 1.0 cm^3 三氯甲烷,混匀,保存于聚乙烯瓶中,瓶口用石蜡熔封。可稳定三个月。开瓶后,使用期不得超过一星期。使用标准缓冲溶液时,应采用测定溶液温度下的标准 pH 值(参见表 F.2)。

6.3.5　饱和氯化钾溶液

称取 40 g 氯化钾(KCl),加 100 cm^3 水,充分搅拌后,将该溶液连同未溶解氯化钾全部转移入试剂瓶中(此溶液应与固体氯化钾共存)。

6.3.6　氯化汞溶液:ρ=25 g/dm^3

称取 2.5 g 氯化汞($HgCl_2$)溶于水并稀释至 100 cm^3,混匀,盛于棕色试剂瓶中。

警告——氯化汞剧毒,小心操作!

6.4　仪器与设备

pH 计:精度为 0.01pH 单位。

6.5　水样装取、预处理和贮存

水样装取、预处理和贮存的操作和要求见 4.4.3.2。

6.6　测定步骤

6.6.1　pH 计校准

在室温下用混合磷酸盐标准缓冲溶液(见 6.3.2.1)和四硼酸钠标准缓冲溶液(见 6.3.2.2)校准 pH 计。将 pH 计上温度补偿器刻度调至与溶液温度一致(若 pH 计有自动温度补偿此步骤省略)。按 pH 计说明书操作步骤分别用上述二种标准缓冲溶液的液温对应的标准 pH 值反复对 pH 计进行校准,至电极电位平衡稳定。每次更换标准缓冲溶液时,应用蒸馏水冲洗电极,然后用滤纸吸干。

6.6.2　水样测定

pH 计校准后将电极对提起,移开标准缓冲溶液,用蒸馏水淋洗电极,然后用滤纸将水吸干。将电极对浸入待测水样中,使电极电位充分平衡,待仪器读数稳定后,记下水样温度和 pH 值读数,填入 pH 测定记录表(参见表 E.3)中。

6.7　计算

将测得的 pH 值按式(7)进行温度和压力校正,求得现场 pH 值。

$$pH_w = pH_m + \alpha(t_m - t_w) - \beta d \qquad (7)$$

式中:

pH_w,pH_m——分别为现场和测定时的 pH 值;

t_w,t_m——分别为现场和测定时的水温,单位为摄氏度(℃);

d——水样深度,单位为米(m);

α,β——分别为温度和压力校正系数,$\alpha(t_m - t_w)$和 βd 分别由表 F.3 和表 F.4 查得。

如果水样深度在 500 m 以内,不必作压力校正,公式(7)可简化为式(8)。

$$pH_w = pH_m + \alpha(t_m - t_w) \qquad (8)$$

按 pH 测定记录表(参见表 E.3)的要求,将数据逐项计算并填写。

7 总碱度测定(pH 法)

7.1 技术指标

准确度:总碱度为 1.5 mmol/dm³ 时,相对误差为±3.5%;总碱度为 2.2 mmol/dm³ 时,相对误差为±2.5%。

精密度:相对标准偏差为±1.5%。

7.2 方法原理

向水样中加入过量已知浓度盐酸溶液以中和水样中的碱,然后用 pH 计测定此混合溶液的 pH 值,由测得值计算混合溶液中剩余的酸量,再从加入的酸总量中减去剩余的酸量即得到水样中碱的量。根据公式(10)计算水样总碱度。

7.3 试剂及其配制

除另有说明外,所用试剂均为分析纯,水为蒸馏水或等效纯水。

7.3.1 邻苯二甲酸氢钾标准缓冲溶液:$c\ (KHC_8H_4O_4) = 0.050$ mol/dm³(25℃时,pH=4.003)

称取 10.21 g 邻苯二甲酸氢钾($KHC_8H_4O_4$,预先在 115℃±5℃下烘干 2 h,于干燥器中冷却)用少量水溶解后,稀释至 1 000 cm³,加 1.0 cm³ 三氯甲烷,混匀,保存于聚乙烯瓶中,可稳定三个月。

7.3.2 盐酸溶液:$c\ (HCl) = 0.006$ mol/dm³

7.3.2.1 量取 8.4 cm³ 盐酸(HCl,$\rho = 1.18$ g/cm³,优级纯)于 1 000 cm³ 量瓶中,用煮沸 15 min 冷至室温的水稀释至标线,混匀。

7.3.2.2 量取 60 cm³ 上述盐酸溶液,用水稀释至 1 000 cm³。即得浓度为 0.006 mol/dm³ 盐酸溶液。

7.3.3 碳酸钠标准溶液:$c\ (1/2\ Na_2CO_3) = 0.010\ 0$ mol/dm³

称取 0.530 0 g 无水碳酸钠(Na_2CO_3,优级纯,预先在 220℃恒温 2 h,置干燥器中冷却至室温),用少量水溶解后,稀释至 1 000 cm³。

7.3.4 甲基红-次甲基蓝混合指示剂

甲基红乙醇溶液:称取 0.032 g 甲基红($C_{15}H_{15}N_3O_2$)溶于 80 cm³ 95%乙醇中。

次甲基蓝乙醇溶液:称取 0.01 g 次甲基蓝($C_{16}H_{18}ClN_3S \cdot 3H_2O$)溶于 100 cm³ 95%乙醇中。

氢氧化钠溶液($\rho = 40.0$ g/dm³):称取 4.0 g 氢氧化钠(NaOH)溶于 100 cm³ 水中。

混合指示剂:将上述已配好的 80 cm³ 甲基红乙醇溶液,加入 6.0 cm³ 次甲基蓝乙醇溶液,混合均匀后,加入 1.2 cm³ 氢氧化钠溶液,溶液呈暗色,贮于棕色瓶中。

7.3.5 盐酸溶液的标定

移取 15.00 cm³ 碳酸钠标准溶液(见 7.3.3)于锥形烧瓶中,加 6 滴甲基红-次甲基蓝混合指示剂(见 7.3.4),用盐酸溶液(见 7.3.2)滴定。当溶液由橙黄色转变为稳定浅紫红色时,即为终点。按式(9)计算盐酸溶液标定浓度。

$$c(HCl) = \frac{c(1/2Na_2CO_3) \times V_{Na_2CO_3}}{V_{HCl}} \quad \cdots\cdots(9)$$

式中:

$c(HCl)$——盐酸溶液标定浓度,单位为摩尔每立方分米(mol/dm³);

$c(1/2Na_2CO_3)$——碳酸钠标准溶液浓度,单位为摩尔每立方分米(mol/dm³);

V_{HCl}——盐酸溶液体积,单位为立方厘米(cm³);

$V_{Na_2CO_3}$——碳酸钠标准溶液体积,单位为立方厘米(cm³)。

7.4 仪器与设备

7.4.1 pH 计:精度为 0.01 pH 单位。

7.4.2 具有内塞的聚乙烯广口瓶:50 cm³。

7.4.3 具塞滴定管。

7.5 水样装取、预处理与贮存

水样装取、预处理与贮存的操作和要求见4.4.3.3。

7.6 测定步骤

pH计定位：用邻苯二甲酸氢钾标准缓冲溶液(见7.3.1)进行定位。

移取25.00 cm^3 水样于50 cm^3 具塞聚乙烯广口瓶中(取双份平行样测定)，加入10.00 cm^3 经标定的盐酸溶液(见7.3.2)，加盖旋紧，充分摇匀。

测定酸化水样的pH值，其测定值应在3.40～3.90范围内。如pH大于3.90时，应取出电极对，另外加入1.00 cm^3 盐酸溶液(见7.3.2)，重新测定pH值；如pH小于3.40，则应另加入5.0 cm^3 水样，重新测定pH值。将加入的盐酸溶液或水样的体积记录于总碱度测定记录表(参见表E.4)中。

7.7 计算

按总碱度测定记录表的要求将数据逐项填写并按式(10)计算总碱度。

$$A=\frac{V_{HCl}\times c(HCl)}{V_W}\times 1\,000-\frac{\alpha_{H^+}\times(V_W+V_{HCl})}{V_W\times f_{H^+}}\times 1\,000 \qquad \cdots\cdots(10)$$

式中：

A——水样总碱度，单位为毫摩尔每立方分米($mmol/dm^3$)；

$c(HCl)$——盐酸溶液标定浓度，单位为摩尔每立方分米(mol/dm^3)；

V_W——水样体积，单位为立方厘米(cm^3)；

V_{CHl}——盐酸溶液体积，单位为立方厘米(cm^3)；

α_{H^+}——与测定溶液pH对应的氢离子活度(由表F.5查得)；

f_{H^+}——与测定溶液pH和实际盐度对应的氢离子活度系数(由表F.6查得)。

8 活性硅酸盐测定(硅钼蓝法)

8.1 技术指标

测定范围：0.10 $\mu mol/dm^3$～25.0 $\mu mol/dm^3$。

检测下限：0.10 $\mu mol/dm^3$。

准确度：浓度为4.5 $\mu mol/dm^3$ 时，相对误差为±5.0%。

精密度：浓度为4.5 $\mu mol/dm^3$ 时，相对标准偏差为±4.0%。

8.2 方法原理

水样中的活性硅酸盐在弱酸性条件下与钼酸铵生成黄色的硅钼黄络合物后，用对甲替氨基酚硫酸盐(米吐尔)-亚硫酸钠将硅钼黄络合物还原为硅钼蓝络合物，于812 nm波长处进行分光光度测定。

8.3 试剂及其配制

除非另有说明，本法中所用试剂均为分析纯；水为无硅去离子水或等效纯水。试剂及纯水应贮于聚乙烯瓶中。

8.3.1 无硅去离子水

自来水经二个串联的阴、阳离子交换树脂纯水器纯化的水。

8.3.2 酸性钼酸铵溶液：ρ=8.0 g/dm^3

称取8.0 g钼酸铵[$(NH_4)_6Mo_7O_{24}\cdot 4H_2O$]，溶于600 cm^3 水中，加24.0 cm^3 盐酸(HCl，ρ=1.18 g/cm^3)，稀释至1 000 cm^3，置于聚乙烯瓶中，避光存放。若容器壁出现大量沉积物，应弃之不用。

8.3.3 草酸溶液：ρ= 100 g/dm^3

称取10 g草酸($H_2C_2O_4\cdot 2H_2O$)，溶于水，稀释至100 cm^3，贮于聚乙烯瓶中。

8.3.4 硫酸溶液：体积分数为25%

在搅拌和水浴冷却下，将100 cm^3 硫酸(H_2SO_4，ρ= 1.84 g/cm^3)缓慢地加入300 cm^3 水中，冷却后贮于聚乙烯瓶中。

8.3.5 对甲替氨基酚硫酸盐-亚硫酸钠溶液

称取 5.0 g 对甲替氨基酚硫酸盐(米吐尔)[$(CH_3NHC_6H_4OH)_2 \cdot H_2SO_4$],溶于 240 cm³ 水,加 3.0 g无水亚硫酸钠(Na_2SO_3),溶解后稀释至 250 cm³,过滤,存于棕色瓶中,盖紧。此溶液不稳定,易变质,最长可存放一个月。

8.3.6 混合还原剂

将 100 cm³ 对甲替氨基酚硫酸盐-亚硫酸钠溶液(见 8.3.5)和 60 cm³ 草酸溶液(见 8.3.3)混合,加 120 cm³ 硫酸溶液(见 8.3.4),混匀,冷却后稀释至 300 cm³,贮于聚乙烯瓶中。此溶液应使用前配制。

8.3.7 人工海水

盐度为 28 的人工海水:称取 25.0 g 氯化钠(NaCl,优级纯)和 8.0 g 硫酸镁($MgSO_4 \cdot 7H_2O$,优级纯),溶于无硅去离子水中,稀释至 1 000 cm³,贮于聚乙烯瓶中。

盐度为 35 的人工海水:称取 31.0 g 氯化钠(NaCl,优级纯)和 10.0 g 硫酸镁($MgSO_4 \cdot 7H_2O$,优级纯),溶于无硅去离子水中,稀释至 1 000 cm³,贮于聚乙烯瓶中。其他盐度的人工海水可按上述比例配制。

8.3.8 硅酸盐标准溶液

8.3.8.1 硅酸盐标准贮备溶液:$c(SiO_3^{2-}\text{-Si}) = 25.0\ \mu mol/cm^3$

将氟硅酸钠(Na_2SiF_6,优级纯)在 105℃下烘干 1 h,取出置于干燥器中冷却至室温。称取 4.702 g 于聚乙烯瓶中,加入约 300 cm³ 无硅水,搅拌至完全溶解,全量转移至 1 000 cm³ 容量瓶中,加水至标线。立刻移入聚乙烯瓶中贮存,加 1 cm³ 三氯甲烷,有效保存期一年。

8.3.8.2 硅酸盐标准使用溶液:$c(SiO_3^{2-}\text{-Si}) = 0.500\ \mu mol/cm^3$

在 100 cm³ 容量瓶中加入 10 cm³ 相应的人工海水,然后移入 2.00 cm³ 硅酸盐标准贮备液(见 8.3.8.1),用水稀释至标线,混匀。使用前配制,不得存放。

8.4 仪器与设备

8.4.1 分光光度计。

8.4.2 容量瓶:100 cm³。

8.4.3 反应瓶:50 cm³。

8.5 水样装取、预处理和贮存

水样装取、预处理和贮存的操作和要求见 4.4.3.4。

8.6 测定步骤

8.6.1 标准工作曲线绘制(0 mol/dm³～25.0 μmol/dm³)

8.6.1.1 取六个 100 cm³ 容量瓶(见 8.4.2),分别移入硅酸盐标准使用溶液(见 8.3.8.2)0 cm³,1.00 cm³,2.00 cm³,3.00 cm³,4.00 cm³,5.00 cm³,用与水样盐度接近的人工海水(盐度 28 或 35)稀释至标线,混匀,即得硅酸盐浓度依次为 0 μmol/dm³,5.0 μmol/dm³,10.0 μmol/dm³,15.0 μmol/dm³,20.0 μmol/dm³,25.0 μmol/dm³ 的标准系列。

8.6.1.2 在两组各六个 50 cm³ 反应瓶中,各加入 10.0 cm³ 酸性钼酸铵溶液(见 8.3.2)。分别依次移入 25.0 cm³ 上述硅标准系列,立即混匀,放置 10 min(但不得超过 30 min)后,各加入 15 cm³ 混合还原剂(见 8.3.6),混匀。

8.6.1.3 30 min 至 40 min 后,在分光光度计上,用 2 cm 比色池,以无硅离子水为参照液,于 812 nm 波长处测定吸光值 A_s。

8.6.1.4 将测定数据记录于标准曲线数据表(参见表 E.5)中。以扣除空白吸光值后的吸光值 A_n 为纵坐标,相应的硅酸盐-硅浓度 C_s 为横坐标绘制标准工作曲线,用线性回归法求得标准工作曲线的截距 a 和斜率 b。

8.6.2 水样测定

加入 10.0 cm³ 酸性钼酸铵溶液(见 8.3.2)于 50 cm³ 反应瓶中,然后移入 25.0 cm³ 水样(每份水样

取双样测定)，立即混匀，以下按 8.6.1.2～8.6.1.3 测定水样吸光值 A_w。

将测定数据记录于活性硅酸盐测定记录表(参见表 E.6)中。

8.6.3 批量样品质量检验

测定每一批样品时，至少必需测定一个标准样品和一个试剂空白样品。根据其吸光值 A_s 和 A_b，按公式(11)求得 C_g，再按公式(12)计算误差 S_b，本方法误差 S_b 不可大于±5.0%。

$$C_g = \frac{(A_s - A_b) - a}{b} \quad \cdots\cdots(11)$$

$$S_b = \frac{C_s - C_g}{C_s} \times 100\% \quad \cdots\cdots(12)$$

式中：

C_g——标准样测得的浓度，单位为微摩尔每立方分米(μmol/dm^3)；

A_s——标准样吸光值；

A_b——试剂空白吸光值；

S_b——检验误差；

C_s——标准样浓度，单位为微摩尔每立方分米(μmol/dm^3)；

a——标准工作曲线截距；

b——标准工作曲线斜率。

8.7 计算

计算数据记录在活性硅酸盐测定记录表(参见表 E.6)中。

按式(13)计算水样中活性硅酸盐-硅的浓度。

$$c(SiO_3^{2-}\text{-}Si) = \frac{(\overline{A}_w - A_b) - a}{b} \quad \cdots\cdots(13)$$

式中：

$c(SiO_3^{2-}\text{-}Si)$——水样中活性硅酸盐-硅的浓度，单位为微摩尔每立方分米(μmol/dm^3)；

$\overline{A}_w$——水样测得的平均吸光值；

A_b——空白吸光值；

a——标准工作曲线截距；

b——标准工作曲线斜率。

对于盐度变化很大的河口水活性硅酸盐的高精确度测定，需用蒸馏水代替人工海水绘制标准工作曲线，并按式(14)计算水样中活性硅酸盐浓度。

$$c(SiO_3^{2-}\text{-}Si) = \frac{(\overline{A}_w - A_b)(1 + 0.004\ S) - a}{b} \quad \cdots\cdots(14)$$

式中：

$c(SiO_3^{2-}\text{-}Si)$——水样中活性硅酸盐-硅的浓度，单位为微摩尔每立方分米(μmol/dm^3)；

$\overline{A}_w$——水样的平均吸光值；

A_b——空白吸光值；

S——水样盐度；

a——标准工作曲线截距；

b——标准工作曲线斜率。

8.8 仲裁方法

活性硅酸盐测定(硅钼蓝法)为仲裁方法。除本方法外，另有硅钼黄法(见附录 A)。

9 活性磷酸盐测定(抗坏血酸还原磷钼蓝法)

9.1 技术指标

测定范围:0.02 μmol/dm^3～4.80 μmol/dm^3。

检测下限:0.02 μmol/dm^3。

准确度:浓度为 0.20 μmol/dm^3 时,相对误差为±10%;浓度为 2.0 μmol/dm^3 时,相对误差为±3.5%。

精密度:浓度为 0.20 μmol/dm^3 时,相对标准偏差为±10%;浓度为 2.0 μmol/dm^3 时,相对标准偏差为±3.0%。

9.2 方法原理

在酸性介质中,活性磷酸盐与钼酸铵反应生成磷钼黄络合物,在酒石酸氧锑钾存在下,磷钼黄络合物被抗坏血酸还原为磷钼蓝络合物,于 882 nm 波长处进行分光光度测定。

9.3 试剂及其配制

除另有说明外,所用试剂均为分析纯,水为蒸馏水或等效纯水。

9.3.1 三氯甲烷(**警告——试剂剧毒,小心操作!**)

9.3.2 硫酸溶液:体积分数为 17%

在水浴冷却和不断搅拌下,将 60 cm^3 硫酸(H_2SO_4,ρ= 1.84 g/cm^3)缓慢加入 300 cm^3 水中,贮存于玻璃瓶中。

9.3.3 钼酸铵溶液:ρ= 30.0 g/dm^3

称取 15.0 g 钼酸铵[$(NH_4)_6Mo_7O_{24}\cdot 4H_2O$]溶于水中并稀释至 500 cm^3,贮于聚乙烯瓶中,避光保存。

9.3.4 抗坏血酸溶液:ρ= 54.0 g/dm^3

称取 5.40 g 抗坏血酸($C_6H_8O_6$)溶于水中并稀释至 100 cm^3。此液贮于聚乙烯瓶中,避免阳光直射。有效期为一星期。在 5℃～6℃下低温保存,可稳定一个月。

9.3.5 酒石酸氧锑钾溶液:ρ= 1.4 g/dm^3

称取 1.4 g 酒石酸氧锑钾($KSbO\cdot C_4H_4O_6\cdot 1/2H_2O$)溶于水中并稀释至 1 000 cm^3,贮于聚乙烯瓶中,有效期为六个月。

9.3.6 硫酸-钼酸铵-酒石酸氧锑钾混合溶液

依次量取 100 cm^3 硫酸溶液(见 9.3.2),40 cm^3 钼酸铵溶液(见 9.3.3),20 cm^3 酒石酸氧锑钾溶液(见 9.3.5),混合均匀。临用时配制。

9.3.7 磷酸盐标准溶液

9.3.7.1 磷酸盐标准贮备溶液:$c(PO_4^{3-}\text{-}P)$ = 8.00 μmol/cm^3

称取 1.088 g 磷酸二氢钾(KH_2PO_4,优级纯,在 110℃～115℃烘干 2 h,置于干燥器中冷却至室温),用少量水溶解后,全量转移至 1 000 cm^3 容量瓶中,用水稀释至标线,加 1.0 cm^3 三氯甲烷,混匀。暗处存放,有效期六个月。

9.3.7.2 磷酸盐标准使用溶液:$c(PO_4^{3-}\text{-}P)$ = 0.080 μmol/cm^3

移取 1.00 cm^3 磷酸盐标准贮备溶液(见 9.3.7.1)于 100 cm^3 容量瓶中,用水稀释至标线。加 3 滴三氯甲烷,混匀,贮存于棕色玻璃瓶中,有效期为 24 h。

9.4 仪器与设备

9.4.1 分光光度计。

9.4.2 可调定量加液器:1.0 cm^3,5.0 cm^3。

9.4.3 容量瓶:100 cm^3。

9.4.4 反应瓶:50 cm^3。

9.5 水样装取、预处理和贮存

水样装取、预处理和贮存的操作和要求见4.4.3.4。

9.6 测定步骤

9.6.1 标准工作曲线绘制(0 μmol/dm^3～4.80 μmol/dm^3)

9.6.1.1 取六个100 cm^3 容量瓶(见9.4.3),分别移入磷酸盐标准使用溶液(见9.3.7.2)0 cm^3, 0.50 cm^3,1.00 cm^3,2.00 cm^3,4.00 cm^3,6.00 cm^3,用水稀释至标线,混匀,即得磷酸盐-磷的浓度依次为0 μmol/dm^3,0.40 μmol/dm^3,0.80 μmol/dm^3,1.60 μmol/dm^3,3.20 μmol/dm^3,4.80 μmol/dm^3 的标准溶液系列。

9.6.1.2 取两组各六个50 cm^3 反应瓶(见9.4.4),分别依次移入25.0 cm^3 上述标准系列溶液(见9.6.1.1),各加入2.0 cm^3 硫酸-钼酸铵-酒石酸氧锑钾混合溶液(见9.3.6)和0.5 cm^3 抗坏血酸溶液(见9.3.4),混匀。

9.6.1.3 显色10 min后,在分光光度计上,用10 cm比色池,以蒸馏水作参照液,于882 nm波长处测量吸光值 A_s,其中空白吸光值为 A_b。

9.6.1.4 将测得的数据记录于标准工作曲线记录表(参见表E.5)中。以扣除空白吸光值后的吸光值 A_n 为纵坐标,相应的磷酸盐-磷浓度 C_s 为横坐标,绘制标准工作曲线。以用线性回归法求得工作曲线截距 a 和斜率 b。

9.6.2 水样测定

量取25.0 cm^3 水样于50 cm^3 反应瓶中(每份水样取双样测定),以下按9.6.1.2～9.6.1.3测定水样吸光值 A_w。

将测得的数据记录于活性磷酸盐测定记录表(参见表E.7)中。

9.6.3 批量样品质量检验

检验方法同8.6.3。本方法误差 S_b 不可大于±5.0%。

9.7 计算

计算数据记录在活性磷酸盐测定记录表(参见表E.7)中。

按下式计算水样的磷酸盐-磷的浓度:

$$c(PO_4^{3-}\text{-P}) = \frac{(\overline{A}_w - A_b) - a}{b} \qquad \cdots\cdots (15)$$

式中:

$c(PO_4^{3-}\text{-P})$——水样中活性磷酸盐-磷的浓度,单位为微摩尔每立方分米(μmol/dm^3);

$\overline{A}_w$——水样的平均吸光值;

A_b——空白吸光值;

a——标准工作曲线截距;

b——标准工作曲线斜率。

10 亚硝酸盐测定(重氮-偶氮法)

10.1 技术指标

测定范围:0.02 μmol/dm^3～4.00 μmol/dm^3。

检测下限:0.02 μmol/dm^3。

准确度:浓度为0.5 μmol/dm^3 时,相对误差为±5.0%;浓度为1.00 μmol/dm^3 时,相对误差为±3.0%。

精密度:浓度为0.3 μmol/dm^3 时,相对标准偏差为±5.0%;浓度为1.00 μmol/dm^3 时,相对标准偏差为±2.0%。

10.2 方法原理

在酸性(pH=2)条件下,水样中的亚硝酸盐与对氨基苯磺酰胺进行重氮化反应,反应产物与1-萘替乙二胺二盐酸盐作用,生成深红色偶氮染料,于543 nm波长处进行分光光度测定。

10.3 试剂及其配制

除另有说明外,所用试剂均为分析纯,水为蒸馏水或等效纯水。

10.3.1 盐酸溶液:体积分数为14%

量取100 mL盐酸(HCl,ρ= 1.18 g/cm^3)与600 cm^3 水混匀。

10.3.2 对氨基苯磺酰胺溶液:ρ= 10 g/dm^3

称取5.0 g对氨基苯磺酰胺($NH_2SO_2C_6H_4NH_2$)溶于350 cm^3 盐酸溶液(见10.3.1)中,用水稀释至500 cm^3,混匀。贮于棕色玻璃瓶中,有效期二个月。

10.3.3 1-萘替乙二胺二盐酸盐溶液:ρ= 1.0 g/dm^3

称取0.5 g 1-萘替乙二胺二盐酸盐($C_{10}H_7NHCH_2CH_2NH_2 \cdot 2HCl$),用少量水溶解后,稀释至500 cm^3,混匀。贮于棕色玻璃瓶中,低温保存。(如出现棕色时应重配)

警告——试剂具有毒性,小心操作!

10.3.4 亚硝酸盐标准贮备溶液:$c(NO_2^- \text{-N})$ = 5.00 μmol/cm^3

称取0.345 0 g亚硝酸钠($NaNO_2$,优级纯,预先在110℃下烘干1 h,置于干燥器中冷却至室温),用少量水溶解后,全量转移至1 000 cm^3 容量瓶中,用水稀释至标线,加1.0 cm^3 三氯甲烷,混匀。避光低温保存,有效期二个月。

10.3.5 亚硝酸盐标准使用溶液:$c(NO_2^- \text{-N})$ = 0.050 μmol/cm^3

吸取1.00 cm^3 亚硝酸盐标准贮备溶液(见10.3.4)于100 cm^3 容量瓶中,用水稀释至标线,混匀。使用前配制,可稳定4 h。

10.4 仪器与设备

10.4.1 分光光度计。

10.4.2 容量瓶:100 cm^3。

10.4.3 反应瓶:50 cm^3。

10.5 水样装取、预处理和贮存

水样装取、预处理和贮存的操作和要求见4.4.3.4。

10.6 测定步骤

10.6.1 标准工作曲线绘制(0 μmol/dm^3~4.00 μmol/dm^3)

10.6.1.1 在两组各六个100 cm^3 容量瓶中依次分别加入亚硝酸盐标准使用溶液(见10.3.5)0 cm^3,0.50 cm^3,1.00 cm^3,2.00 cm^3,4.00 cm^3,8.00 cm^3,用水稀释至标线,混匀。此标准溶液系列的亚硝酸盐-氮的浓度依次为0 μmol/dm^3,0.25 μmol/dm^3,0.50 μmol/dm^3,1.00 μmol/dm^3,2.00 μmol/dm^3,4.00 μmol/dm^3。

10.6.1.2 分别量取25.0 cm^3 上述系列标准溶液,依次放入两组各六个50 cm^3 反应瓶中。各加入0.5 cm^{3}对氨基苯磺酰胺溶液(见10.3.2),混匀。放置5 min,然后,加入0.5 cm^3 1-萘替乙二胺二盐酸盐溶液(见10.3.3),混匀。放置15 min。

10.6.1.3 在分光光度计上,用5 cm比色池,以蒸馏水为参比,于543 nm波长处测量吸光值A_s,其中,空白吸光值为A_b。吸光值测定应在4 h内完成。测定数据记录于标准曲线数据记录表(参见表E.5)中。

10.6.1.4 以扣除空白吸光值A_b后的吸光值A_n为纵坐标,相应的亚硝酸盐-氮浓度C_s为横坐标,绘制标准工作曲线。用线性回归方法求得标准曲线的截距a和斜率b。

10.6.2 水样测定

量取25.0 cm^3 水样于50 cm^3 反应瓶中(取双样)。以下按10.6.1.2~10.6.1.3测定水样吸光

值 A_w。

将测定数据记录于亚硝酸盐测定记录表(参见表 E.8)中。

10.6.3 批量样品质量检验

检验方法同 8.6.3。本方法误差 S_b 不可大于±5.0%。

10.7 计算

按式(16)计算求得水样中亚硝酸盐-氮的浓度：

$$c(NO_2^- \text{-N}) = \frac{(\overline{A}_w - A_b) - a}{b} \qquad \cdots\cdots(16)$$

式中：

$c(NO_2^- \text{-N})$——水样中亚硝酸盐-氮的浓度，单位为微摩尔每立方分米($\mu mol/dm^3$)；

$\overline{A}_w$——水样的平均吸光值；

A_b——空白吸光值；

a——标准工作曲线截距；

b——标准工作曲线斜率。

11 硝酸盐测定(锌镉还原法)

11.1 技术指标

测定范围：0.05 $\mu mol/dm^3$～16.0 $\mu mol/dm^3$。

检测下限：0.05 $\mu mol/dm^3$。

准确度：浓度为 2.0 $\mu mol/dm^3$ 时，相对误差为±7.0%；浓度为 10.0 $\mu mol/dm^3$ 时，相对误差为±4.0%。

精密度：浓度为 5.0 $\mu mol/dm^3$ 时，相对标准偏差为±4.0%；浓度为 10.0 $\mu mol/dm^3$ 时，相对标准偏差为±3.0 %。

11.2 方法原理

用镀镉的锌片将水样中的硝酸盐定量地还原为亚硝酸盐，水样中的总亚硝酸盐再用重氮-偶氮法测定，然后对原有的亚硝酸盐进行校正，计算硝酸盐含量。

11.3 试剂及其配制

除另有说明外，本法中所用试剂均为分析纯，水为蒸馏水或等效纯水。

11.3.1 三氯甲烷($CHCl_3$)

11.3.2 锌卷

将锌片(纯度 99.99%，厚度 0.1 mm)，裁成 5.0 cm×3.0 cm 小片，卷成内径约 1.5 cm 的锌卷。

11.3.2.1 锌片表面应光洁明亮，无边角毛刺、残缺，无腐蚀斑点。

11.3.2.2 锌片剪裁前应用纱布仔细擦净表面。

11.3.3 人工海水：盐度为 35

称取 31.0 g 氯化钠(NaCl，优级纯)、10.0 g 硫酸镁($MgSO_4 \cdot 7H_2O$，优级纯)和 0.5 g 碳酸氢钠($NaHCO_3$，优级纯)溶于水中，稀释至 1 dm^3。

11.3.4 无氮海水

取低氮海水过滤后放置陈化半年，用孔径 0.45 μm 微孔滤膜过滤即得。

11.3.5 氯化镉溶液：ρ= 20.0 g/dm^3

称取 20.0 g 氯化镉($CdCl_2 \cdot 5/2H_2O$)溶于水中，并用水稀释至 1 000 cm^3，混匀。

警告——试剂剧毒，小心操作！

11.3.6 对氨基苯磺酰胺溶液：ρ=10 g/dm^3

对氨基苯磺酰胺溶液的配制方法同 10.3.2。

11.3.7 1-萘替乙二胺二盐酸盐溶液：$\rho = 1.0\ g/dm^3$

1-萘替乙二胺二盐酸盐溶液的配制方法同10.3.3。

11.3.8 硝酸盐标准贮备溶液：$c(NO_3^- -N) = 10.0\ \mu mol/cm^3$

称取1.011 g硝酸钾（KNO_3，优级纯，预先在110℃烘1 h，置于干燥器中冷却至室温）用少量水溶解后，全量转移至1 000 cm^3容量瓶中，用水稀释至标线，加1.0 cm^3三氯甲烷，混匀。有效期为半年。

11.3.9 硝酸盐标准使用溶液：$c(NO_3^- -N) = 0.100\ \mu mol/cm^3$

移取1.00 cm^3硝酸盐标准贮备溶液（见11.3.8）于100 cm^3容量瓶中，用水稀释至标线，混匀。使用前配制。

11.4 仪器与设备

11.4.1 分光光度计。

11.4.2 往返式电动振荡器：频率150 r/min～250 r/min。

11.4.3 具塞广口玻璃瓶：30 cm^3。

11.4.4 定量加液器：1.0 cm^3。

11.4.5 秒表。

11.5 水样装取、预处理和贮存

水样装取、预处理和贮存的操作和要求见4.4.3.4。

11.6 测定步骤

11.6.1 标准工作曲线的绘制（0 $\mu mol/dm^3$～16.0 $\mu mol/dm^3$）

11.6.1.1 在两组各六个25 cm^3容量瓶中，分别依次移入硝酸盐标准使用溶液（见11.3.9）0 cm^3，0.50 cm^3，1.00 cm^3，1.50 cm^3，2.50 cm^3，4.00 cm^3，用盐度为35的人工海水稀释至标线，混匀。此标准溶液系列硝酸盐-氮浓度依次为0 $\mu mol/dm^3$，2.00 $\mu mol/dm^3$，4.00 $\mu mol/dm^3$，6.00 $\mu mol/dm^3$，10.0 $\mu mol/dm^3$，16.0 $\mu mol/dm^3$。

11.6.1.2 将上述标准溶液系列（见11.6.1.1）分别全量转移到一组干燥的30 cm^3具塞广口瓶中，向每个瓶中放入一个锌卷（见11.3.2），加入0.50 cm^3氯化镉溶液（见11.3.5），迅速放在振荡器上振荡10 min。振荡后迅速将瓶中的锌卷取出。

11.6.1.3 加入0.50 cm^3对氨基苯磺酰胺溶液（见11.3.6），混匀，放置5 min，再加入0.50 cm^3 1-萘替乙二胺二盐酸盐溶液（见11.3.7），混匀，放置15 min。颜色可稳定4 h。

11.6.1.4 颜色稳定后，在分光光度计上，用2 cm比色池，以水为参比，于543 nm波长处测定吸光值A_s，其中空白吸光值为A_b。测定结果记录于标准工作曲线记录表（参见附录E.5）中。

11.6.1.5 以扣除空白吸光值A_b后的吸光值A_n为纵坐标，硝酸盐-氮的浓度C_s为横坐标绘制标准工作曲线，并用线性回归法求出标准工作曲线截距a和斜率b。

11.6.2 水样测定

量取25.0 cm^3水样（双样）于30 cm^3干燥的具塞广口瓶中，以下按11.6.1.2～11.6.1.4步骤测定水样的吸光值A_w，并记录于硝酸盐测定记录表（参见表E.9）中。

如果水样盐度低于25，测定时每份水样应加入0.5 g优级纯氯化钠。

将海水样品中原有亚硝酸盐在"亚硝酸盐测定"测得的净平均吸光值$\overline{A}_{NO_2^- -N}$（已扣除试剂空白），和"硝酸盐测定"与"亚硝酸盐测定"的比色池长度的比值X，记录于硝酸盐测定记录表（参见表E.9）中。

11.6.3 批量样品质量检验

检验方法同8.6.3。本方法误差S_b不可大于±8.0%。

11.6.4 锌镉还原率测定

11.6.4.1 各量取25.0 cm^3人工海水和含10.0 $\mu mol/dm^3$硝酸盐人工海水分别放于30 cm^3具塞广口瓶中，按11.6.1.2～11.6.1.4步骤测定其吸光值A_{b1}和$A_{NO_3^- -N}$。

11.6.4.2 各量取25.0 cm^3人工海水和含10.0 $\mu mol/dm^3$亚硝酸盐人工海水分别放于30 cm^3具塞广

口瓶中，按11.6.1.2～11.6.1.4步骤测定其吸光值 A_{b2} 和 $A_{NO_2^--N}$。

11.6.4.3 还原率计算

按下式计算镀镉锌卷的还原率 R：

$$R = \frac{A_{NO_3^--N} - A_{b1}}{A_{NO_2^--N} - A_{b2}} \times 100\% \quad \cdots\cdots (17)$$

每批样品测定用的锌卷还原率应大于75%，且其还原率偏差应小于5%。

11.7 计算

水样中硝酸盐-氮浓度按下式计算：

$$c(NO_3^--N) = \frac{(\overline{A}_w - A_b) - X \cdot \overline{A}_{NO_2^--N} - a}{b} \quad \cdots\cdots (18)$$

式中：

$c(NO_3^--N)$——水样中硝酸盐-氮的浓度，单位为微摩尔每立方分米（$\mu mol/dm^3$）；

$\overline{A}_w$——水样测得的平均吸光值；

A_b——空白吸光值；

$\overline{A}_{NO_2^--N}$——该水样在“亚硝酸盐测定”时测得的平均吸光值（已扣除试剂空白）；

X——“硝酸盐测定”和“亚硝酸盐测定”所用比色池的长度比，按本规范条件为0.4；

a——标准工作曲线截距；

b——标准工作曲线斜率。

11.8 仲裁方法

硝酸盐测定（锌镉还原法）为仲裁方法。除本方法外，另有铜镉柱还原法（见附录B）。

12 铵盐测定（次溴酸钠氧化法）

12.1 技术指标

测定范围：0.03 $\mu mol/dm^3$～8.00 $\mu mol/dm^3$。

检测下限：0.03 $\mu mol/dm^3$。

准确度：浓度为1.00 $\mu mol/dm^3$ 时，相对误差为±7.0%；浓度为7.0 $\mu mol/dm^3$ 时，相对误差为±4.0%。

精密度：浓度为1.00 $\mu mol/dm^3$ 时，相对标准偏差为±7.0%；浓度为7.0 $\mu mol/dm^3$ 时，相对标准偏差为±3.0%。

12.2 方法原理

在碱性条件下，次溴酸钠将海水中的铵定量氧化为亚硝酸盐，用重氮-偶氮法测定生成亚硝酸盐和水样中原有的亚硝酸盐，然后，对水样中原有的亚硝酸盐进行校正，计算铵氮的浓度。

12.3 试剂及其配制

12.3.1 无铵水

无铵水可采用下述两种制备方法中的任何一种制得。

a) 方法1：将蒸馏水通过一个长30 cm，直径为1 cm～2 cm的阳离子交换树脂柱（使用前转为氢型树脂），收集于玻璃瓶中，塞紧瓶盖。

b) 方法2：将1.0 dm^3 蒸馏水放入蒸馏水器中，加入15 cm^3 0.5 mol/dm^3 氢氧化钠溶液和2.0 g过硫酸钾（$K_2S_2O_8$）。先敞口煮沸10 min，然后接好冷凝器，收集馏出液于聚乙烯瓶中，直至蒸馏器中的水剩下150 cm^3 左右，所收集的蒸馏水即为无铵蒸馏水，盖紧瓶塞，待用。

12.3.2 氢氧化钠溶液：ρ= 400 g/dm^3

称取400 g氢氧化钠（NaOH，优级纯），溶于2 000 cm^3 无铵蒸馏水中，蒸煮浓缩至1 000 cm^3，冷去后，贮于聚乙烯瓶中。盖紧瓶塞。

12.3.3 盐酸溶液:体积分数为50%

量取500 cm^3 盐酸(HCl,ρ=1.18 g/cm^3,应保证浓度符合要求)和500 cm^3 水混合,贮于试剂瓶中。

12.3.4 溴酸钾-溴化钾溶液

称取2.8 g溴酸钾($KBrO_3$)和20.0 g溴化钾(KBr)溶于1 000 cm^3 无铵水中,低温保存,此溶液有效期一年。

12.3.5 次溴酸钠氧化剂溶液

吸取1.0 cm^3 溴酸钾-溴化钾溶液(见12.3.4)于棕色试剂瓶中,加入49 cm^3 水,加入3.0 cm^3 盐酸溶液(见12.3.3),迅速盖上瓶盖,混匀,置于暗处5 min,加入50 cm^3 氢氧化钠溶液(见12.3.2),混匀。使用前配制,此溶液在35℃以下可稳定8 h。

12.3.6 对氨基苯磺酰胺溶液:ρ=2.0 g/dm^3

称取1.0 g对氨基苯磺酰胺($NH_2SO_2C_6H_4NH_2$)溶于500 cm^3 盐酸溶液(见12.3.3)中,贮于棕色试剂瓶中。

12.3.7 1-萘替乙二胺二盐酸盐溶液:ρ=1.0 g/dm^3

配制方法同10.3.3。

12.3.8 铵标准贮备溶液:$c(NH_4^+\text{-}N)$ = 10.0 μmol/cm^3

称取0.534 9 g氯化铵(NH_4Cl,预先在100℃烘1 h,置于干燥器中冷却至室温),用少量水溶解后,全量转移至1 000 cm^3 容量瓶中,用水稀释至标线,加1.0 cm^3 三氯甲烷,混匀。低温冷藏,有效期六个月。

12.3.9 铵标准使用溶液:$c(NH_4^+\text{-}N)$ = 0.050 μmol/cm^3

移取1.00 cm^3 铵标准贮备溶液(见12.3.8)于200 cm^3 容量瓶中,用水稀释至标线,混匀。有效期1 d。

12.3.10 说明

除另有说明外,本法所用试剂均为优级纯,水为无铵蒸馏水,或新制备的去离子水。

12.4 仪器与设备

12.4.1 分光光度计。

12.4.2 玻璃蒸馏器或阳离子交换纯水器。

12.5 水样装取、预处理和贮存

水样装取、预处理和贮存的操作和要求见4.4.3.4。

12.6 测定步骤

12.6.1 标准工作曲线的绘制(0 μmol/dm^3~8.00 μmol/dm^3)

12.6.1.1 在两组各六个50 cm^3 容量瓶中分别移入铵标准使用溶液(见12.3.9)0 cm^3,0.50 cm^3,1.00 cm^3,2.50 cm^3,5.00 cm^3,8.00 cm^3,用无铵水稀释至标线,混匀。此标准溶液系列铵氮浓度依次为0 μmol/dm^3,0.50 μmol/dm^3,1.00 μmol/dm^3,2.50 μmol/dm^3,5.00 μmol/dm^3,8.00 μmol/dm^3。

12.6.1.2 将标准溶液系列分别移取25.0 cm^3 到50 cm^3 反应瓶中,加入2.5 cm^3 次溴酸钠氧化剂溶液(见12.3.5),混匀,放置30 min。

12.6.1.3 加入2.5 cm^3 对氨基苯磺酰胺溶液(见12.3.6),混匀,放置5 min。然后加入0.5 cm^3 1-萘替乙二胺二盐酸盐溶液(见12.3.7),充分混匀,放置15 min。颜色可稳定4 h。

12.6.1.4 颜色稳定后,在分光光度计上,用5 cm比色池,以无铵蒸馏水为参比,于543 nm波长处测定吸光值 A_s,其中,空白吸光值为 A_b。记录于标准工作曲线数据记录表(参见表E.5)中。

12.6.1.5 以扣除空白吸光值 A_b 后的吸光值 A_n 为纵坐标,铵氮浓度 C_s 为横坐标绘制标准工作曲线,并用线性回归法求出标准工作曲线的截距 a 和斜率 b。

12.6.2 水样测定

量取25.0 cm^3 水样(双样)于50 cm^3 反应瓶中,按照12.6.1.2~12.6.1.4步骤测定水样的吸光值

A_w，记录于铵盐测定记录表(参见表 E.10)中。

将该水样在“亚硝酸盐测定”时，亚硝酸盐扣除试剂空白后的吸光值$\overline{A}_{NO_2^- \text{-N}}$，填入铵盐测定记录表(参见表 E.10)中。

12.6.3 批量样品质量检验

检验方法同 8.6.3。本方法误差 S_b 不可大于±8.0%。

12.7 计算

水样中铵氮浓度按式(19)计算。

$$c(NH_4^+\text{-N}) = \frac{(\overline{A}_w - A_b) - k \cdot \overline{A}_{NO_2^- \text{-N}} - a}{b} \quad \cdots\cdots (19)$$

式中：

$c(NH_4^+\text{-N})$——水样中铵氮的浓度，单位为微摩尔每立方分米(μmol/dm³)；

$\overline{A}_w$——水样测得的平均吸光值；

A_b——空白吸光值；

$\overline{A}_{NO_2^- \text{-N}}$——水样在“亚硝酸盐测定”时，扣除试剂空白后的平均吸光值；

a——铵标准工作曲线截距；

b——铵标准工作曲线斜率；

k——测定亚硝酸盐和测定铵的试液体积(水样体积与试剂体积之和)比值，按本规范条件为 0.85。

12.8 仲裁方法

铵盐测定(次溴酸钠氧化法)为仲裁方法。除本方法外，另有靛酚蓝法(见附录 C)。

13 氯化物测定(银量滴定法)

13.1 技术指标

测定范围：0.2 g/dm³～20.0 g/dm³。

检测下限：0.2 g/dm³。

准确度：浓度为 2.0 g/dm³ 时，相对误差为±1.0%；浓度为 18.0 g/dm³ 时，相对误差为±0.15%。

精密度：浓度为 18.0 g/dm³ 时，相对标准偏差为±0.10%。

13.2 方法原理

海水中的氯离子在中性或弱碱性条件下，用硝酸银溶液滴定形成氯化银沉淀，以荧光黄钠盐为指示剂判断滴定终点。当溶液由黄绿色刚转变为浅玫瑰红色时，即为滴定终点。用相同方法滴定氯化钠标准溶液，从而计算海水样品氯离子浓度。

13.3 试剂及其配制

除另有说明外，所用试剂均为分析纯，水为蒸馏水或等效纯水。

13.3.1 氢氧化钠溶液：ρ= 4.0 g/dm³

称取 4.0 g 氢氧化钠($NaOH$)溶于水中，并用水稀释至 1 000 cm³。

13.3.2 硝酸溶液：$c(HNO_3)$= 0.1 mol/dm³

移取 1.0 cm³ 硝酸(HNO_3，ρ= 1.42 g/cm³)，用水稀释至 140 cm³。

13.3.3 硝酸银溶液：ρ= 60 g/dm³

称取 60.0 g 硝酸银($AgNO_3$)溶于水中，并用水稀释至 1 000 cm³。贮于棕色瓶中，置于暗处备用，使用前标定。如果硝酸银不纯或溶液久置后产生沉淀，可以把上层清液倾出或滤去沉淀，标定后仍可使用。

13.3.4　氯化钠标准溶液：$\rho_{NaCl-Cl}=19.86\ g/dm^3$

称取 32.74 g 氯化钠（NaCl，优级纯，预先在 450℃～500℃灼烧 1 h，在干燥器中冷却至室温），溶解于适量水中，全量转移至 1 000 cm^3 容量瓶中，用水稀释至标线，混匀。贮于具塞玻璃瓶中，盖紧。

13.3.5　荧光黄钠盐溶液：$\rho=1.0\ g/dm^3$

称取 0.1 g 荧光黄（$C_{20}H_{22}O_5$）溶于 10 cm^3 氢氧化钠溶液（见 13.3.1）中，用 pH 试纸指示，以硝酸溶液（见 13.3.2）中和至中性，用水稀释至 100 cm^3，贮于棕色试剂瓶中。

13.3.6　淀粉溶液

称取可溶性淀粉 2.5 g，用少量水调成糊状，加入 250 cm^3 沸水中，再煮至沸，冷却，贮于试剂瓶中。

13.3.7　荧光黄钠盐指示剂

量取 12.5 cm^3 荧光黄钠盐溶液（见 13.3.5）加于 250 cm^3 淀粉溶液（见 13.3.6），再加入 0.25 g 苯甲酸钠（C_6H_5COONa），混合均匀，贮于棕色试剂瓶中。此溶液可稳定一个月，如有絮状物析出应弃去重配。

13.4　仪器与设备

13.4.1　海水吸量管：10 cm^3。

13.4.2　滴定管：25 cm^3 溶解氧滴定管。

13.4.3　电磁搅拌器及包裹聚乙烯或玻璃的磁搅拌转子。

13.5　水样装取、预处理和贮存

水样装取、预处理和贮存的操作和要求见 4.4.3.3。

13.6　测定步骤

13.6.1　硝酸银溶液的标定

13.6.1.1　用氯化钠标准溶液（见 13.3.4）洗涤海水吸量管二次，然后，在三个 100 cm^3 烧杯中，分别移入三份 10.00 cm^3 氯化钠标准溶液（见 13.3.4），各加入 1.5 cm^3 荧光黄钠盐指示剂（见 13.3.7），放入磁转子。

13.6.1.2　用硝酸银溶液（见 13.3.3）洗涤滴定管二次，然后注入硝酸银溶液至满标。

13.6.1.3　在电磁搅拌下进行滴定。当溶液变为玫瑰红色时，即为终点。读取滴定管体积读数 V_s（读准至 0.01 cm^3），将滴定管体积读数 V_s 填入氯化物测定记录表（参见表 E.11）。

注：每批样品测定前，均应进行硝酸银溶液标定。如长时间连续测定，一般每隔 4 h 标定一次。若室温较稳定，可每隔 8 h 标定一次。

13.6.2　水样测定

样品应放置至其温度接近室温时测定。用少量水样洗涤海水吸量管二次，然后吸取 10.00 cm^3 水样于 100 cm^3 烧杯中，加入 1.5 cm^3 荧光黄钠盐指示剂（见 13.3.7），放入磁转子，按 13.6.1.3 进行滴定。每个水样应取双样平行测定。将滴定管体积读数 V_w 记录于氯化物测定记录表（参见表 E.11）中。

13.7　计算

水样中氯化物的氯离子浓度 ρ_{Cl} 按式（20）计算。

$$\rho_{Cl}=\frac{\rho_{NaCl-Cl}\times V_1\times\overline{V}_w}{V_2\times\overline{V}_s}\qquad\cdots\cdots(20)$$

式中：

ρ_{Cl}——水样的氯离子浓度，单位为克每立方分米（g/dm^3）；

$\rho_{NaCl-Cl}$——氯化钠标准溶液中氯离子的浓度，单位为克每立方分米（g/dm^3）；

V_1——标定硝酸银溶液时，使用的氯化钠标准溶液体积，单位为立方厘米（cm^3）；

$\overline{V}_w$——水样滴定时，消耗的硝酸银溶液体积平均值，单位为立方厘米（cm^3）；

V_2——水样体积，单位为立方厘米（cm^3）；

$\overline{V}_s$——标定硝酸银标准溶液时，消耗的硝酸银溶液体积的平均值，单位为立方厘米（cm^3）。

14 总磷测定(过硫酸钾氧化法)

14.1 技术指标

测定范围:0.09 $\mu mol/dm^3$～6.4 $\mu mol/dm^3$。

检测下限:0.09 $\mu mol/dm^3$。

准确度:以甘油磷酸钠($C_3H_7NaO_6P \cdot 5\ 1/2H_2O$)为标准加入物,其方法回收率为 98%～100%;以六偏磷酸钠[$(NaPO_3)_6$]为标准加入物,其方法回收率为 93%～98%。

精密度:总磷浓度为 1 $\mu mol/dm^3$～6.4 $\mu mol/dm^3$ 时,相对标准偏差为±5%。

14.2 方法原理

海水样品在酸性和 110℃～120℃条件下,用过硫酸钾氧化,有机磷化合物被转化为无机磷酸盐,无机聚合态磷水解为正磷酸盐。消化过程产生的游离氯,以抗坏血酸还原。消化后水样中的正磷酸盐与钼酸铵形成磷钼黄。在酒石酸氧锑钾存在下,磷钼黄被抗坏血酸还原为磷钼蓝,于 882 nm 波长处进行分光光度测定。

14.3 试剂及其配制

除另有说明外,所用试剂均为分析纯,实验用水为二次蒸馏水或等效纯水。

14.3.1 硫酸溶液:体积分数为 17%

硫酸溶液的配制方法同 9.3.2。

14.3.2 过硫酸钾溶液:ρ= 50 g/dm^3

称取 5.0 g 过硫酸钾($K_2S_2O_8$)溶于水中,并用水稀释至 100 cm^3,混匀。此溶液室温避光保存可稳定 10 d;4℃～6℃避光保存可稳定 30 d。

过硫酸钾的试剂空白若达不到要求时,可用多次重结晶方法提纯。

14.3.3 钼酸铵溶液:ρ= 30.0 g/dm^3

钼酸铵溶液的配制方法同 9.3.3。

14.3.4 抗坏血酸溶液:ρ= 54.0 g/dm^3

抗坏血酸溶液的配制方法同 9.3.4。

14.3.5 酒石酸氧锑钾溶液:ρ= 1.4 g/dm^3

酒石酸氧锑钾溶液的配制方法同 9.3.5。

14.3.6 硫酸-钼酸铵-酒石酸氧锑钾混合溶液

硫酸-钼酸铵-酒石酸氧锑钾混合溶液的配制方法同 9.3.6。

14.3.7 磷酸盐标准溶液

14.3.7.1 磷酸盐标准贮备溶液:$c(PO_4^{3-}\text{-P})$ = 8.00 $\mu mol/cm^3$

磷酸盐标准贮备溶液的配制方法同 9.3.7.1。

14.3.7.2 磷酸盐标准使用溶液:$c(PO_4^{3-}\text{-P})$ = 0.080 $\mu mol/cm^3$

磷酸盐标准使用溶液的配制方法同 9.3.7.2。

14.4 仪器与设备

14.4.1 仪器与设备包括:

a) 医用手提式蒸气灭菌器或家用压力锅,压力可达到 1.1 kPa～1.4 kPa,温度可达 120℃～124℃。

b) 分光光度计。

c) 消煮瓶:60 cm^3～100 cm^3,带螺旋盖的聚四氟乙烯瓶或聚丙烯瓶。

14.4.2 实验所用的器皿均应用体积分数为 10% 盐酸溶液浸泡 24 h 后,再用水冲洗干净。

14.5 水样装取、预处理和贮存

水样装取、预处理和贮存的操作和要求见 4.4.3.5。

14.6 测定步骤

14.6.1 标准工作曲线绘制(0 μmol/dm³～6.4 μmol/dm³)

14.6.1.1 在六个100 cm³容量瓶中,分别移入磷酸盐标准使用溶液(见14.3.7.2)0 cm³,0.5 cm³,1.00 cm³,2.00 cm³,4.00 cm³,8.00 cm³,用水稀释至标线,混匀。此标准溶液系列的浓度依次为0 μmol/dm³,0.40 μmol/dm³,0.80 μmol/dm³,1.6 μmol/dm³,3.2 μmol/dm³,6.4 μmol/dm³。

14.6.1.2 在两组各六个消煮瓶(见14.4.3)中,分别依次移入25.0 cm³上述标准溶液系列,各加入2.5 cm³过硫酸钾溶液(见14.3.2),混匀,旋紧瓶盖。

14.6.1.3 把上述消煮瓶置于不锈钢丝筐中,放入高压蒸汽消煮器(见14.4.1)中加热消煮,待压力升至1.1 kPa(温度为120℃)时,控制压力在1.1 kPa～1.4 kPa(温度120℃～124℃)保持30 min。然后,停止加热,自然冷却至压力为"0"时,方可打开锅盖,取出消煮瓶。

14.6.1.4 消煮后的水样冷却至室温后,加入0.50 cm³抗坏血酸溶液(见14.3.4),摇匀,加入2.0 cm³硫酸-钼酸铵-酒石酸氧锑钾混合溶液(见14.3.6)和0.50 cm³抗坏血酸溶液(见14.3.4),混匀,显色10 min后,在分光光度计上,用5 cm比色池,以水作参比,于882 nm波长处测定溶液的吸光值A_s,其中,空白吸光值为A_b。记录于标准工作曲线数据记录表(参见表E.5)中。

14.6.1.5 以扣除空白吸光值A_b后的吸光值A_n为纵坐标,标准溶液系列的浓度C_s为横坐标,绘制标准工作曲线,并用线性回归法求出标准工作曲线的截距a和斜率b。

14.6.2 水样测定

14.6.2.1 量取25.0 cm³海水水样(双样)于消煮瓶(见14.4.3)中,加入2.5 cm³过硫酸钾溶液(见14.3.2),混匀,旋紧瓶盖。

14.6.2.2 以下按14.6.1.3～14.6.1.4步骤测定水样吸光值A_w,记录于总磷测定记录表(参见表E.15)中。

14.6.3 批量样品质量检验

检验方法同8.6.3。本方法校正误差S_b不可大于±5.0%。

14.6.4 浑浊度测定

如果水样的浊度对吸光值有影响,应进行浊度校正:取25.0 cm³水样按14.6.1.2～14.6.1.3步骤,于相同的波长(882 nm)处测定水样浊度的吸光值A_t。

14.7 计算

水样中总磷浓度可按式(21)计算。

$$c(\text{TP-P}) = \frac{(\overline{A}_w - A_t - A_b) - a}{b} \quad \cdots\cdots(21)$$

式中:

c(TP-P)——水样中总磷的浓度,单位为微摩尔每立方分米(μmol/dm³);

$\overline{A}_w$——水样中总磷的平均吸光值;

A_t——水样中浊度的吸光值,如果无需浊度校正时,该项为0;

A_b——空白吸光值;

a——标准工作曲线截距;

b——标准工作曲线斜率。

15 总氮测定(过硫酸钾氧化法)

15.1 技术指标

测定范围:3.78 μmol/dm³～32.0 μmol/dm³。

检测下限:3.78 μmol/dm³。

准确度:以标准有机氮物(日本化学会制)、甘氨酸为有机氮标准加入物进行回收实验,其方法回收

率为 94%～101%。

精密度：浓度为 20 μmol/dm^3 时，相对标准偏差为±5%。

15.2 **方法原理**

海水样品在碱性和 110℃～120℃条件下，用过硫酸钾氧化，有机氮化合物被转化为硝酸氮。同时，水中的亚硝酸氮、铵态氮也定量地被氧化为硝酸氮。硝酸氮经还原为亚硝酸盐后与对氨基苯磺酰胺进行重氮化反应，反应产物再与 1-萘替乙二胺二盐酸盐作用，生成深红色偶氮染料，于 543 nm 波长处进行分光光度测定。

15.3 **试剂及其配制**

除另有说明外，所用试剂均为分析纯，实验用水为二次蒸馏水或等效纯水。

15.3.1 二次蒸馏水

在 1 000 cm^3 蒸馏水中加入 0.5 g 过硫酸钾($K_2S_2O_8$)和 20 cm^3 氢氧化钠溶液(见 15.3.2)，于全玻璃蒸馏器中重蒸馏，敞口煮沸 3 min 后接冷凝管，弃前 50 cm^3 馏出水，然后将馏出水收集于具塞玻璃瓶中，蒸馏至母液剩下约 150 cm^3 时，停止蒸馏，弃去母液。

15.3.2 氢氧化钠溶液：c (NaOH)＝ 1.0 mol/dm^3

称取 20.0 g 氢氧化钠(NaOH)于 1 000 cm^3 烧杯中，加入 500 cm^3 水(见 15.3.1)，煮沸 5 min，冷却后用水补充至 500 cm^3，贮于聚乙烯瓶中。

所使用的氢氧化钠应经总氮试剂空白值检验合格方可使用。

15.3.3 过硫酸钾溶液：ρ＝ 50.0 g/dm^3

称取 5.0 g 过硫酸钾($K_2S_2O_8$)溶解于 50 cm^3 氢氧化钠溶液(见 15.3.2)中，用水(见 15.3.1)稀释至 100 cm^3，存放于聚乙烯瓶中。此溶液于室温避光保存可稳定 7 d；在 4℃～6℃避光保存可稳定 30 d。

所使用过硫酸钾应进行试剂空白值检验，总氮空白值若达不到要求时，可用多次重结晶方法提纯。

15.3.4 盐酸溶液：c (HCl)＝ 1.5 mol/dm^3

量取 12.5 cm^3 浓盐酸(HCl，ρ＝ 1.19 g/cm^3)加入到 87.5 cm^3 水中，混匀。

15.3.5 氯化钠溶液：ρ＝ 35.0 g/dm^3

称取 35.0 g 氯化钠(NaCl)溶于水中，并用水稀释至 1 000 cm^3，混匀，贮于试剂瓶中。

15.3.6 四硼酸钠溶液：ρ＝ 38.1 g/dm^3

称取 19.05 g 四硼酸钠($Na_2B_4O_7 \cdot 10H_2O$)溶于水中，并用水稀释至 500 cm^3，混匀，贮于试剂瓶中。

15.3.7 锌卷

锌卷的配制方法同 11.3.2。

15.3.8 氯化镉溶液：ρ＝ 20.0 g/dm^3

氯化镉溶液的配制方法同 11.3.5。

15.3.9 对氨基苯磺酰胺溶液：ρ＝ 10 g/dm^3

对氨基苯磺酰胺溶液的配制方法同 10.3.2。

15.3.10 1-萘替乙二胺二盐酸盐溶液：ρ＝ 1.0 g/dm^3

1-萘替乙二胺二盐酸盐溶液的配制方法同 10.3.3。

15.3.11 硝酸盐标准贮备溶液：c (NO_3-N)＝ 10.0 μmol/cm^3

硝酸盐标准贮备溶液的配制方法同 11.3.8。

15.3.12 硝酸盐标准使用溶液：c (NO_3-N)＝ 0.100 μmol/cm^3

硝酸盐标准使用溶液的配制方法同 11.3.9。

15.4 **仪器与设备**

15.4.1 医用手提式蒸气灭菌器或家用压力锅

压力可达到 1.1 kPa～1.4 kPa，温度可达 120℃～124℃。

15.4.2 分光光度计

15.4.3 消煮瓶：60 cm^3～100 cm^3

带螺旋盖的聚四氟乙烯瓶或聚丙烯瓶。

注：实验所用的器皿均用体积分数为10%盐酸溶液浸泡24 h后，再用水冲洗干净。

15.5 水样装取、预处理和贮存

水样装取、预处理和贮存的操作和要求见4.4.3.5。

15.6 测定步骤

15.6.1 标准工作曲线的绘制(0 μmol/dm^3～32.0 μmol/dm^3)

15.6.1.1 在两组各六个25 cm^3 容量瓶中，分别移入0 cm^3，1.00 cm^3，2.00 cm^3，4.00 cm^3，6.00 cm^3，8.00 cm^3 硝酸盐标准使用溶液(见15.3.12)，用氯化钠溶液(见15.3.5)稀释至标线，混匀。此标准溶液系列的硝酸盐氮浓度依次为0 μmol/dm^3，4.00 μmol/dm^3，8.00 μmol/dm^3，16.00 μmol/dm^3，24.0 μmol/dm^3，32.0 μmol/dm^3。

15.6.1.2 将上述标准溶液系列分别全量转移到消煮瓶中，每个容量瓶用10 cm^3 氯化钠溶液(见15.3.5)分二次洗涤，洗涤液一并转入对应的消煮瓶中。

15.6.1.3 各加入4.0 cm^3 过硫酸钾溶液(见15.3.3)旋紧瓶盖。

15.6.1.4 把装上水样的消煮瓶置于不锈钢筐中，放入高压蒸汽消煮器(见15.4.1)中加热消煮，待压力升至1.1 kPa(温度120℃)时，控制压力在1.1 kPa～1.4 kPa(温度120℃～124℃)并保持30 min。然后，放置使之自然冷却，待压力降至“0”后方可打开锅盖，取出样品。

15.6.1.5 样品冷却后，加入0.5 cm^3 盐酸溶液(见15.3.4)，振摇使沉淀物溶解。

15.6.1.6 水样转移到100 cm^3 容量瓶中，用氯化钠溶液(见15.3.5)洗涤消煮瓶3次，洗涤液一并转入容量瓶中，加入2.0 cm^3 四硼酸钠溶液(见15.3.6)，用氯化钠溶液(见15.3.5)稀释至标线，混匀。

15.6.1.7 量取25.0 cm^3 经消煮定容后的样品，于50 cm^3 具塞广口瓶中，放入一个锌卷(见15.3.7)，加入0.50 cm^3 氯化镉溶液(见15.3.8)，迅速放在振荡器上，振荡10 min，振荡后迅速取出瓶中的锌卷。

15.6.1.8 加入0.50 cm^3 对氨基苯磺酰胺溶液(见15.3.9)，混匀，放置5 min，再加入0.50 cm^3 1-萘替乙二胺二盐酸盐溶液(见15.3.10)，混匀，放置15 min。颜色可稳定8 h。

15.6.1.9 颜色稳定后，在分光光度计上，用2 cm比色池，以水作为参比，于543 nm波长处，测定溶液的吸光值 A_s，其中，空白吸光值为 A_b。记录于标准工作曲线数据记录表(参见表E.5)中。

15.6.1.10 以扣除空白吸光值 A_b 后的吸光值 A_n 为纵坐标，标准溶液系列的硝酸盐氮浓度 C_s 为横坐标，绘制标准工作曲线，并用线性回归法求出标准工作曲线的截距 a 和斜率 b。

15.6.2 水样测定

15.6.2.1 量取25.0 cm^3 水样于消煮瓶中，加入10 cm^3 氯化钠溶液(见15.3.5)，再加4.0 cm^3 过硫酸钾溶液(见15.3.3)，旋紧瓶盖。以下按照15.6.1.4～15.6.1.6步骤进行消煮、调节酸度和定容。

15.6.2.2 量取25.0 cm^3 经消煮定容(见15.6.2.1)后的样品于50 cm^3 具塞广口玻璃中，以下按照15.6.1.7～15.6.1.9步骤测定水样的吸光值 A_w，记录于总氮测定记录表(参见表E.16)中。

15.6.3 批量样品质量检验

检验方法同8.6.3。本方法误差 S_b 不可大于±10.0%。

15.6.4 浑浊度测定

如果水样较为浑浊时，有必要进行浊度影响校正：量取25.0 cm^3 经消煮定容后的水样(见15.6.2.1)，加0.50 cm^3 对氨基苯磺酰胺溶液(见15.3.9)和1.50 cm^3 水，混匀后，用相同的比色池于543 nm波长处测定浊度吸光值 A_t。

15.7 计算

水样中总氮浓度可按式(22)计算。

$$c(\text{TN-N}) = \frac{(A_w - A_t - A_b) - a}{b} \quad \cdots\cdots(22)$$

式中：

c(TN-N)——水样中总氮的浓度，单位为微摩尔每立方分米（μmol/dm^3）；

A_w——水样中总氮的吸光值；

A_t——水样中浊度的吸光值，如果无需浊度校正时，该项为0；

A_b——空白吸光值；

a——标准工作曲线截距；

b——标准工作曲线斜率。

附　录　A
（规范性附录）
活性硅酸盐测定（硅钼黄法）

A.1　技术指标

测定范围：0.45 $\mu mol/dm^3$～160 $\mu mol/dm^3$。

检测下限：0.45 $\mu mol/dm^3$。

准确度：浓度为 20 $\mu mol/dm^3$ 时，相对误差为±5.0%；浓度为 100 $\mu mol/dm^3$ 时，相对误差为±3.0%。

精密度：浓度为 20 $\mu mol/dm^3$ 时，相对标准偏差为±6.0%；浓度为 100 $\mu mol/dm^3$ 时，相对标准偏差为±4.0%。

A.2　方法原理

在弱酸性条件下，水样中的活性硅酸盐与钼酸铵反应生成黄色硅钼酸盐络合物，于 380 nm 波长处进行分光光度测定。

A.3　试剂及其配制

除另有说明外，所用试剂均为分析纯，所用的水均为无硅蒸馏水（见 8.3.1）。

A.3.1　钼酸铵溶液：ρ= 100 g/dm^3

称取 20 g 钼酸铵[$(NH_4)_6Mo_7O_{24}\cdot 4H_2O$]，溶于水中，并用水稀释至 200 cm^3，混匀。（如混浊应过滤），贮于聚乙烯瓶中。

A.3.2　硫酸溶液：体积分数为 20%

在搅拌和水浴冷却下，将 50 cm^3 硫酸（H_2SO_4，ρ= 1.84 g/cm^3），缓慢加入到 200 cm^3 水中。

A.3.3　钼酸铵-硫酸显色试剂

将 100 cm^3 硫酸溶液（见 A.3.2）和 200 cm^3 钼酸铵溶液（见 A.3.1）混匀，贮于聚乙烯瓶中，有效期 7 d。

A.3.4　硅酸盐标准溶液

A.3.4.1　硅酸盐标准贮备溶液：$c(SiO_3^{2-}\text{-}Si)$ = 25.0 $\mu mol/cm^3$

硅酸盐标准贮备溶液的配制方法同 8.3.8.1。

A.3.4.2　硅酸盐标准使用溶液：$c(SiO_3^{2-}\text{-}Si)$ = 0.500 $\mu mol/cm^3$

硅酸盐标准使用溶液的配制方法同 8.3.8.2。

A.4　仪器与设备

A.4.1　分光光度计。

A.4.2　容量瓶：25 cm^3。

A.5　水样装取、预处理和贮存

水样装取、预处理和贮存的操作和要求见 4.4.3.4。

A.6　测定步骤

A.6.1　标准工作曲线绘制（0 $\mu mol/dm^3$～160 $\mu mol/dm^3$）

A.6.1.1 在两组各六个 25 cm^3 容量瓶中分别移入浓度为 0.500 $\mu mol/dm^3$ 的硅酸盐标准使用溶液(见 A.3.4.2)0 cm^3,1.00 cm^3,2.00 cm^3,4.00 cm^3,6.00 cm^3,8.00 cm^3,用无硅去离子水(见 8.3.1)稀释至标线,混匀。此溶液系列的硅酸盐-硅浓度依次为 0 $\mu mol/dm^3$,20.0 $\mu mol/dm^3$,40.0 $\mu mol/dm^3$,80.0 $\mu mol/dm^3$,120.0 $\mu mol/dm^3$,160.0 $\mu mol/dm^3$。

A.6.1.2 对上述溶液系列,全量移入 50 cm^3 反应瓶中,各加入 1.5 cm^3 钼酸铵-硫酸显色试剂(见 A.3.3),混匀。在 5℃～10℃时,显色 20 min～30 min;在 10℃～20℃时,显色 15 min;在 20℃以上时,显色 10 min。

A.6.1.3 颜色稳定后(可稳定 45 min),在分光光度计上用 2 cm 比色池,以无硅蒸馏水为参比,于 380 nm波长处测定溶液的吸光值 A_s,其中,空白吸光值为 A_b。将测定数据记录于标准工作曲线数据记录表(参见表 E.5)中。

A.6.1.4 以扣除空白吸光值 A_b 后的吸光值 A_n 为纵坐标,相应的硅酸盐-硅浓度 C_s 为横坐标绘制标准工作曲线,并用线性回归法求出硅酸盐标准工作曲线的截距 a 和斜率 b。

A.6.2 水样测定

量取 25.0 cm^3 水样(每份水样取双样)于 50 cm^3 的反应瓶中,按 A.6.1.2～A.6.1.3 步骤测定水样吸光值 A_w,将测定数据记录于活性硅酸盐测定记录表(参见表 E.6)中。

A.6.3 批量样品质量检验

检验方法同 8.6.3。本方法误差 S_b 不可大于±5.0%。

A.7 计算

不同盐度水样的硅酸盐硅的浓度,分别按式(A.1)和式(A.2)计算:

当盐度(S)＞ 7 时:

$$c(SiO_3^{2-}\text{-}Si) = \frac{(\overline{A}_w - A_b)(1.05 + 0.001\ S) - a}{b} \quad \cdots\cdots(A.1)$$

当盐度(S)≤ 7 时:

$$c(SiO_3^{2-}\text{-}Si) = \frac{(\overline{A}_w - A_b)(1.0 + 0.008\ S) - a}{b} \quad \cdots\cdots(A.2)$$

式中:

$c(SiO_3^{2-}\text{-}Si)$——水样中活性硅酸盐-硅的浓度,单位为微摩尔每立方分米($\mu mol/dm^3$);

$\overline{A}_w$——水样的平均吸光值;

S——盐度;

A_b——空白吸光值;

a——标准工作曲线截距;

b——标准工作曲线斜率。

附 录 B
（规范性附录）
硝酸盐测定（镉铜柱还原法）

B.1 技术指标

测定范围：0.04 μmol/dm³～14.0 μmol/dm³。

检测下限：0.04 μmol/dm³。

准确度：浓度为 6.00 μmol/dm³ 时，相对误差为±4.0%；浓度为 10.0 μmol/dm³ 时，相对误差为±2.0%。

精密度：浓度为 4.00 μmol/dm³ 时，相对标准偏差为±3.0%；浓度为 9.00 μmol/dm³ 时，相对标准偏差为±2.0%。

B.2 方法原理

水样通过填有镀铜镉屑（或镉粒）还原柱时，水中的硝酸盐几乎定量地被还原为亚硝酸盐，然后用重氮-偶氮法测定总亚硝酸盐吸光值，扣除水样中原有亚硝酸盐的吸光值后，计算硝酸盐含量。

B.3 试剂及其配制

除另有说明外，所用试剂均为分析纯，水为二次去离子水或等效纯水。

B.3.1 镉粒：Cd，纯度为 99.99%，粒度：过 40 目（0.25 mm）～60 目（0.45 mm）。

注：剧毒，小心操作！

B.3.2 人工海水：盐度为 35

人工海水的配制方法同 8.3.7.2。

B.3.3 无氮海水

无氮海水的配制方法同 11.3.4。

B.3.4 对氨基苯磺酰胺溶液：ρ= 10 g/dm³

对氨基苯磺酰胺溶液的配制方法同 10.3.2。

B.3.5 1-萘替乙二胺二盐酸盐溶液：ρ= 1.0 g/dm³

1-萘替乙二胺二盐酸盐溶液的配制方法同 10.3.3。

B.3.6 氯化铵溶液 A：ρ= 250 g/dm³

称取 250 g 氯化铵（NH_4Cl）溶于水中，加入 25 cm³ 浓氨水（NH_4OH），并稀释至 1 000 cm³。

B.3.7 氯化铵溶液 B：ρ= 6.25 g/dm³

量取 25 cm³ 氯化铵溶液 A（B3.6），用水稀释至 1 000 cm³。

B.3.8 硫酸铜溶液：ρ= 20.0 g/dm³

称取 20.0 g 硫酸铜（$CuSO_4 \cdot 5H_2O$）溶于水中并稀释至 1 000 cm³。

B.3.9 碳酸钠溶液：ρ= 40.0 g/dm³

称取 40.0 g 碳酸钠（Na_2CO_3）溶于水中并稀释至 1 000 cm³。

B.3.10 再生液

称取 38 g 乙二胺四乙酸二钠（$C_{10}N_{14}N_2O_8Na_2 \cdot 2H_2O$）和 12.5 g 硫酸铜溶于水中并稀释至 1 000 cm³，然后用碳酸钠溶液（见 B.3.9）调节 pH 为 7。

B.3.11 硝酸盐标准贮备溶液：$c(NO_3^- \text{-N})$ = 10.0 μmol/cm³

硝酸盐标准贮备溶液的配制方法同 11.3.8。

B.3.12 硝酸盐标准使用溶液：$c(NO_3^- \text{-N}) = 0.400\ \mu mol/cm^3$

移取 4.00 cm^3 硝酸盐标准贮备溶液(见 B.3.11)于 100 cm^3 容量瓶中，用水稀释至标线，混匀。临用时配制。

B.3.13 还原柱淋洗液：$c(NO_3^- \text{-N}) = 100\ \mu mol/dm^3$

移取 10.0 cm^3 硝酸盐标准贮备溶液(见 B.3.11)于 1 000 cm^3 容量瓶中，用水稀释至标线，混匀。

B.4 仪器与设备

B.4.1 分光光度计。

B.4.2 反应瓶：50 cm^3。

B.4.3 玻璃样品瓶：100 cm^3。

B.4.4 还原装置

还原装置的主要部分是 U 型玻璃管，全长约为 40 cm，内径 4 mm～6 mm，两端用弯曲的毛细管(内径约 2 mm 聚乙烯管)分别与 100 cm^3 样品瓶，50 cm^3 锥形瓶连接起来。

B.4.5 还原柱的制备

取约 70 g 镉粒(见 B.3.1)于 150 cm^3 锥形瓶中，用 50 cm^3 浓度为 2.0 mol/dm^3 的盐酸洗涤二次。用水洗涤 2 遍。加入 150 cm^3 硫酸铜溶液(见 B.3.8)，用力摇动约 3 min，然后用水彻底洗去含胶体态铜和分散的细铜粒，即为镀铜镉粒。

先将 U 型管注满水，再将镀铜镉粒缓慢倒入 U 型管中(装柱时镉粒不得接触空气)，装柱时轻轻敲打柱子，U 型管的两端应留下一些空间填充玻璃纤维。

用氯化铵溶液 B(见 B.3.7)，以 8 cm^3/min ～12 cm^3/min 流量淋洗 10 min，然后注入氯化铵溶液 B(见 B.3.7)，放置 24 h～48 h。

使用前，取 3.0 dm^3 无氨海水或人工海水，加入 15.0 cm^3 硝酸盐标准贮备溶液(见 B.3.11)，加 15 cm^3 氯化铵溶液 A(见 B.3.6)，混匀后流过还原柱，以稳定其还原率，然后注满氯化铵溶液 B(见 B.3.7)保存。

B.5 水样装取、预处理和贮存

水样装取、预处理和贮存的操作和要求见 4.4.3.4。

B.6 测定步骤

B.6.1 标准工作曲线的绘制(0 $\mu mol/dm^3$～14.0 $\mu mol/dm^3$)

B.6.1.1 取 100 cm^3 还原柱淋洗液(见 B.3.13)，加 2.0 cm^3 氯化铵溶液 A(见 B.3.6)混匀，以 8 cm^3/min～12 cm^3/min 流量通过还原柱，然后以 100 cm^3 氯化铵溶液 B(见 B.3.7)洗柱。

B.6.1.2 在六个 100 cm^3 容量瓶中，分别加入 0 cm^3，0.50 cm^3，1.00 cm^3，1.50 cm^3，2.50 cm^3，3.50 cm^3 硝酸盐标准使用溶液(见 B.3.12)，用盐度约为 35 的人工海水(见 B.3.2)稀释至标线，混匀。此标准溶液系列硝酸盐-氮浓度依次为 0 $\mu mol/dm^3$，2.00 $\mu mol/dm^3$，4.00 $\mu mol/dm^3$，6.00 $\mu mol/dm^3$，10.0 $\mu mol/dm^3$，14.0 $\mu mol/dm^3$。

B.6.1.3 将上述溶液分别全量倒入 100 cm^3 样品瓶中，各加入 2.0 cm^3 氯化铵溶液 A(见 B.3.6)，混匀。

B.6.1.4 以 8 cm^3/min ～12 cm^3/min 流量通过还原柱，弃去最初流出液约 25 cm^3，然后各收集两份 25.0 cm^3 分别置于 50 cm^3 反应瓶中。

B.6.1.5 向收集的溶液中加入 0.5 cm^3 对氨基苯磺酰胺溶液(见 B.3.4)，混匀，5 min 后，加入 0.5 cm^3 1-萘替乙二胺二盐酸盐溶液(见 B.3.5)，混匀，放置 15 min，颜色可稳定 4 h。

B.6.1.6 颜色稳定后，用 2 cm 比色池，以人工海水为参比，在 543 nm 波长处，测定标准溶液系列的吸

光值 A_s，其中，空白吸光值为 A_b。记录于标准工作曲线记录表(参见表 E.5)中。

B.6.1.7 以扣除空白吸光值 A_b 后的吸光值 A_n 为纵坐标，标准溶液系列硝酸盐-氮的浓度 C_s 为横坐标，绘制标准工作曲线，并用线性回归法求出标准工作曲线的截距 a 和斜率 b。

B.6.2 水样测定

用 100 cm³ 氯化铵溶液 B(见 B.3.7)洗涤还原柱。

取 100 cm³ 水样于 100 cm³ 样品瓶中，加 2.0 cm³ 氯化铵溶液 A(见 B.3.6)，混匀，按 B.6.1.4～B.6.1.6步骤测定水样吸光值 A_w，记录于硝酸盐测定记录表(参见表 E.9)中。

B.6.3 批量样品质量检验

检验方法同 8.6.3。本方法误差 S_b 不可大于±5.0%。

B.6.4 还原柱还原率的测定

B.6.4.1 各量取 100 cm³ 人工海水和 100 cm³ 含 10.0 μmol/dm³ 的硝酸盐人工海水分别放于 100 cm³ 样品瓶中，各加入 2.0 cm³ 氯化铵溶液 A(见 B.3.6)，混匀，然后按 B.6.13～B.6.15 的步骤分别测定其吸光值 A_{b1}，A_{NO_3}。

B.6.4.2 各量取 50.0 cm³ 人工海水和 50.0 cm³ 含 10.0 μmol/dm³ 的亚硝酸盐人工海水分别放于 50 cm³反应瓶中，加入 0.5 cm³ 氯化铵溶液 A(见 B.3.6)，混匀，加入 0.5 cm³ 对氨基苯磺酰胺溶液(见 B.3.4)，混匀，5 min 后，加入 0.5 cm³ 1-萘替乙二胺二盐酸盐溶液(见 B.3.5)，混匀，放置 15 min 后，分别测定其吸光值 A_{b2}，A_{NO_2}。

B.6.4.3 硝酸盐还原率 R 的计算

计算方法同 11.6.4.3 的公式(17)。当 $R<$ 95%时，还原柱应再生或重新装柱。

B.6.5 还原柱的活化、保养及再生方法

B.6.5.1 若还原柱数天不用，则按 B.6.1.1 重新淋洗活化。

B.6.5.2 若还原柱数小时不用，需用 50 cm³ 还原柱淋洗液(见 B.3.13)，加 1.0 cm³ 氯化铵溶液 A(见 B.3.6)淋洗活化，再以 100 cm³ 氯化铵溶液 B(见 B.3.7)淋洗。

B.6.5.3 实验结束，还原柱均需注满氯化铵溶液 B(见 B.3.7)或密封保存。

B.6.5.4 若还原柱的还原率低于 95%时，用 200 cm³ 再生液(见 B.3.10)流过还原柱，然后分别用水和氯化铵溶液 B(见 B.3.7)淋洗。最后按 B.6.1.1 的操作步骤活化。

B.7 计算

水样中硝酸盐-氮浓度按下式计算：

$$c(NO_3^- \text{-N}) = \frac{(\overline{A}_w - A_b) - X \cdot \overline{A}_{NO_2^- \text{-N}} - a}{b} \qquad \cdots\cdots\cdots\cdots(B.1)$$

式中：

$c(NO_3^-\text{-N})$——水样中硝酸盐-氮浓度，单位为微摩尔每立方分米(μmol/dm³)；

$\overline{A}_w$——水样的平均吸光值；

A_b——空白吸光值；

$\overline{A}_{NO_2^-\text{-N}}$——该水样在“亚硝酸盐测定”时，扣除空白后亚硝酸盐的平均吸光值；

X——“硝酸盐测定”和“亚硝酸盐测定”时，所用比色池的长度比，按本规范条件为 0.4；

a——标准工作曲线截距；

b——标准工作曲线斜率。

附 录 C
（规范性附录）
铵盐测定（靛酚蓝法）

C.1 技术指标

测定范围：0.05 μmol/dm³～8.00 μmol/dm³。

检测下限：0.05 μmol/dm³。

准确度：浓度为 0.70 μmol/dm³ 时，相对误差为±5.0%；浓度为 3.20 μmol/dm³ 时，相对误差为±3.0%。

精密度：浓度为 0.70 μmol/dm³ 时，相对标准偏差为±4.0%；浓度为 3.20 μmol/dm³ 时，相对标准偏差为±2.5%。

C.2 方法原理

铵在弱碱介质中先与次氯酸钠反应生成氯胺，后者在适量催化剂亚硝基铁氰化钠（硝普钠）和过量次氯酸钠存在下与苯酚反应形成靛酚蓝，于 630 nm 波长处进行分光光度测定。海水中碱土金属离子在弱酸性溶液中生成氢氧化物并与溶液中颗粒物质和腐殖质一起沉淀于反应瓶底部。用上部显色清液进行测定，可消除浊度对测定的干扰。

C.3 试剂及其配制

除另有说明外，所用试剂均为优级纯，所用水为无铵蒸馏水或新制备的去离子水。

C.3.1 无铵水

无铵水的配制方法同 12.3.1。

C.3.2 无铵海水

取浮游植物刚刚大量繁殖后的表层海水或外海的表层无铵海水，置于具塞的容器中密封储藏备用。

C.3.3 苯酚-亚硝基铁氰化钠混合试剂

称取 38 g 苯酚（C_6H_5OH）和 0.4 g 亚硝基铁氰化钠[$Na_2Fe(CN)_5NO \cdot 2H_2O$]，在热水浴中用少量水溶解后，再用水稀释至 1 000 cm³。贮于棕色玻璃瓶中。低温保存，若溶液呈淡绿色，应重配（一般稳定约一个月）。

C.3.4 次氯酸钠碱性溶液

取 1 000 cm³ 0.5 mol/dm³ 氢氧化钠溶液加入 31 cm³ 含 5%有效氯的次氯酸钠溶液，混匀，贮于聚乙烯瓶中，低温保存，有效期二星期。若次氯酸钠溶液所含有效氯低于 5%时，应按配得的溶液中应含 0.15%有效氯来计算次氯酸钠的用量。

有效氯的测定方法：取 0.5 g 碘化钾溶于 50 cm³ 硫酸溶液[H_2SO_4，$c(H_2SO_4)$ = 0.5 mol/dm³]中。加入 0.10 cm³ 次氯酸钠溶液，用已准确标定过的硫代硫酸钠溶液[$Na_2S_2O_3$，$c(Na_2S_2O_3)$ = 0.01 mol/dm³]按溶解氧滴定方法测定游离碘。消耗 1.00 cm³ 0.01 mol/dm³ 硫代硫酸钠溶液相当 0.354 mg有效氯。

C.3.5 铵标准贮备溶液：$c(NH_4^+\text{-N})$ = 10.0 μmol/cm³

铵标准贮备溶液的配制方法同 12.3.8。

C.3.6 铵标准使用溶液：$c(NH_4^+\text{-N})$ = 0.050 μmol/cm³

铵标准使用溶液的配制方法同 12.3.9。

C.4 仪器与设备

C.4.1 分光光度计。

C.4.2 螺口玻璃瓶：50 cm^3。

C.4.3 容量瓶：50 cm^3。

C.5 水样装取、预处理和贮存

水样装取、预处理和贮存的操作和要求见 4.4.3.4。

C.6 测定步骤

C.6.1 标准工作曲线的绘制：0 $\mu mol/dm^3$～8.00 $\mu mol/dm^3$

C.6.1.1 在 6 个 50 cm^3 容量瓶中，分别移入铵标准使用溶液(见 12.3.9)0 cm^3，0.50 cm^3，1.00 cm^3，2.50 cm^3，5.00 cm^3，8.00 cm^3，用无铵蒸馏水(或天然无氨海水)稀释至标线，混匀。此标准溶液系列铵-氮浓度依次为 0 $\mu mol/dm^3$，0.50 $\mu mol/dm^3$，1.00 $\mu mol/dm^3$，2.50 $\mu mol/dm^3$，5.00 $\mu mol/dm^3$，8.00 $\mu mol/dm^3$。

C.6.1.2 将上述标准溶液系列分别全量转移入六个 50 cm^3 螺口玻璃瓶中后，加入 1.5 cm^3 苯酚-亚硝基铁氰化钠混合试剂(见 C.3.3)，立即混匀，再加入 1.5 cm^3 次氯酸钠碱性溶液(见 C.3.4)，旋紧瓶盖，混匀，放置 6 h(室温 15℃以上放置 6 h；10℃以下放置 10 h。如室温太低，可在温水浴中升温，以缩短显色时间)。颜色可稳定 4 d。

C.6.1.3 将上部清液小心移入 5 cm 比色池内，以无铵蒸馏水为参比，于 630 nm 波长处测定其吸光值 A_s，其中，空白吸光值为 A_b。记录于标准工作曲线记录表(参见表 E.5)中。

C.6.1.4 以扣除空白吸光值 A_b 后的吸光值 A_n 为纵坐标，标准溶液系列铵-氮浓度 C_s 为横坐标绘制标准工作曲线，并用线性回归法求出该工作曲线的截距 a 和斜率 b。

C.6.2 水样测定

准确移取 50 cm^3 水样置于螺口玻璃瓶中。按 C.6.1.2～C.6.1.3 步骤测定水样吸光值 A_w，记录于铵盐测定记录表(参见表 E.12)中。

C.6.3 批量样品质量检验

检验方法同 8.6.3。本方法误差 S_b 不可大于±5.0%。

C.7 计算

水样中的铵氮浓度按下式计算：

$$c(NH_4^+\text{-}N)=\frac{(\overline{A}_w-A_b)-a}{b} \qquad \cdots\cdots\cdots\cdots (C.1)$$

式中：

$c(NH_4^+\text{-}N)$——水样中铵-氮浓度，单位为微摩尔每立方分米($\mu mol/dm^3$)；

$\overline{A}_w$——水样的平均吸光值；

A_b——空白吸光值；

a——标准工作曲线截距；

b——标准工作曲线斜率。

注：若以与水样盐度相近的无铵海水制备铵氮标准系列溶液，则水样测定的吸光值应进行盐度校正；若以无铵蒸馏水配制标准系列溶液，则应将 $\overline{A}_w$ 乘于盐度校正系数 F_s。

F_s 的校正值见表 C.1。

表 C.1 F_s 的校正值

S	0-8	11	14	17	20	23	27	30	33	36
F_s	1.00	1.01	1.02	1.03	1.04	1.05	1.06	1.07	1.08	1.09

附　录　D
（规范性附录）
溶解氧测定（分光光度法）

D.1　技术指标

测定范围：1.0 μmol/dm³～600 μmol/dm³。

检测下限：1.0 μmol/dm³。

精密度：浓度为 340 μmol/dm³ 时，相对标准偏差为±0.5%，浓度为 540 μmol/dm³ 时，相对标准偏差为±0.5%。

D.2　方法原理

水样中加入适量氯化锰和碘化钾/叠氮化钠溶液后，生成的氢氧化锰被水中的溶解氧氧化为高价氢氧化锰沉淀。叠氮化钠使水中亚硝酸盐分解而消除干扰。加酸酸化后，沉淀溶解，同时释出与溶解氧等当量的游离碘。然后，试液经虹吸流入具流动式比色池的分光光度计，在 456 nm 波长下进行分光测定。

D.3　试剂及其配制

D.3.1　氯化锰溶液：$c(MnCl_2)$= 3.0 mol/dm³

称取 600 g 氯化锰（$MnCl_2\cdot 4H_2O$）溶于水中，并用水稀释至 1 000 cm³。

D.3.2　碱性碘化钠/叠氮化钠溶液：

称取 600 g 碘化钠（NaI），在不断搅拌下，逐次加入到 700 cm³ 水中，待完全溶解后，置于水浴冷却并不断搅拌下，将 320 g 氢氧化钠（NaOH）慢慢加入到碘化钠溶液中，直到完全溶解。另外称取 10 g 叠氮化钠（NaN_3）溶于 40 cm³ 水中。将上述两溶液混合，并用水稀释至 1 000 cm³。

D.3.3　硫酸溶液：体积分数为 29%

在水浴冷却和不断搅拌下，将 100 cm³ 浓硫酸（H_2SO_4，ρ= 1.84 g/cm³）缓慢地加入到 250 cm³ 水中。

D.3.4　硫代硫酸钠溶液：$c(Na_2S_2O_3)$= 0.2 mol/dm³

称取 24.82 g 硫代硫酸钠（$Na_2S_2O_3\cdot 5H_2O$）溶于 500 cm³ 水中，贮于棕色试剂瓶。

D.3.5　碘酸钾标准溶液：$c(KIO_3)$= 5.00×10^3 μmol/dm³

称取 1.070 g 碘酸钾（KIO_3，优级纯，预先在 120℃烘 2 h，置于干燥器中冷却至室温）溶于少量水中，转移至 1 000 cm³ 容量瓶中，用水稀释至标线，混匀，贮于棕色试剂瓶。

D.4　仪器与设备

D.4.1　分光光度计。

D.4.2　带三通阀的流动式比色池：1 cm，进样系统，如图 D.1 所示。

D.4.3　水样瓶：60 cm³ 棕色磨口玻璃瓶。

D.4.4　电磁搅拌器：可调速。

D.4.5　可调定量加液器：1 cm³，5 cm³。

D.4.6　容量瓶：1 000 cm³。

D.4.7　移液管：1 cm³，5 cm³。

D.5 水样装取、预处理和贮存

水样装取、预处理和贮存的操作和要求见4.4.3.5。

D.6 测定步骤

D.6.1 分光光度测定装置调试

装有吸样器的分光光度计其光室外侧加挂一个吸样三通阀，三端用ϕ2 mm聚四氟乙烯管分别与高位槽(装蒸馏水)、流动式比色池和水样瓶连接(见图D.1所示)。将波长调至456 nm，转动三通阀与蒸馏水桶联通，使蒸馏水充满管路(流量约30 cm³/min～40 cm³/min)，再转动三通阀与盛有染色蒸馏水的水样瓶联通，染色水样被虹吸流经比色池(吸样流量控制在20 cm³/min～30 cm³/min)，吸光值升高，待染色水样充满比色池，吸光值稳定后，再转动三通阀与蒸馏水桶联通，使蒸馏水冲洗比色池，至吸光值降至趋于零，并稳定，即可进行仪器调零。

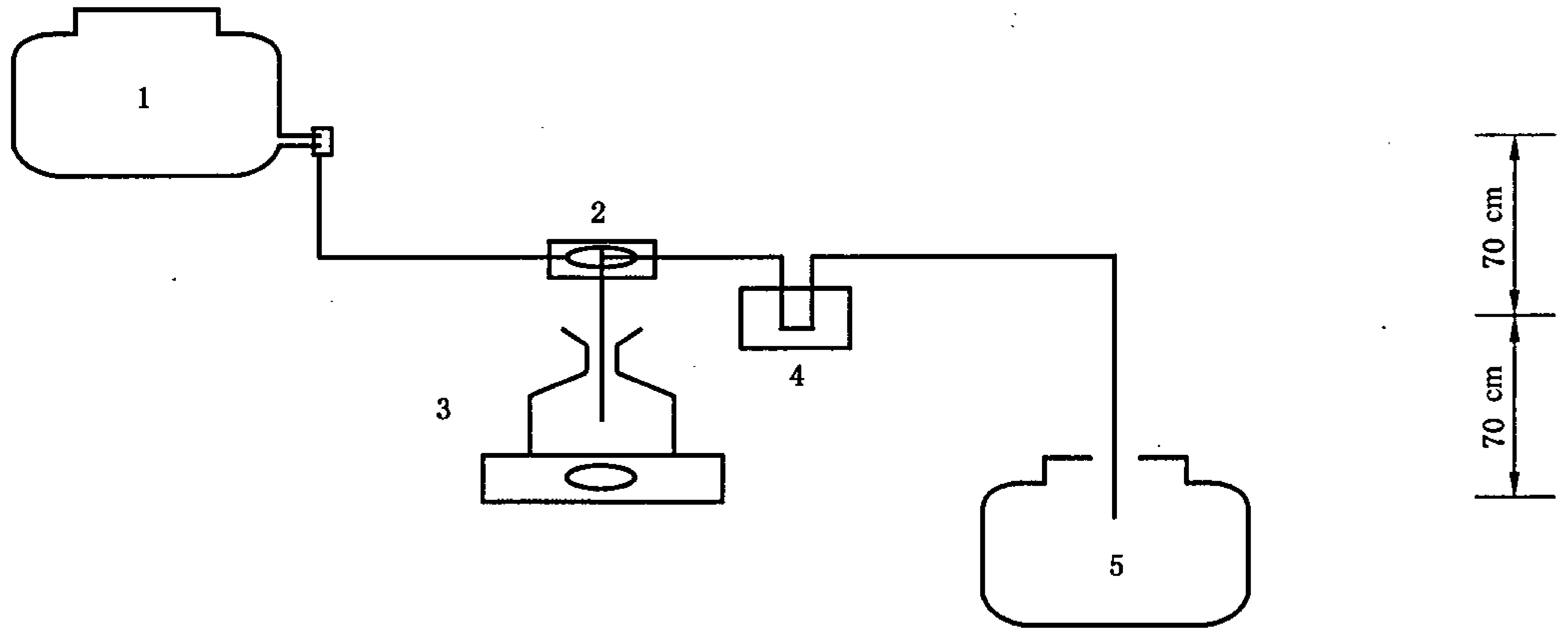

1——蒸馏水桶；
2——三通阀；
3——水样瓶与搅拌器；
4——流动比色池；
5——废液桶。

图D.1 测定装置示意图

D.6.2 水样测定

水样固氧(见4.4.3.1 b)后，待沉淀降至瓶的下部便可进行测定。

D.6.2.1 小心打开水样瓶塞，投入一颗搅拌子。

D.6.2.2 加入0.5 cm³硫酸溶液(见D.3.3)，将水样瓶置于电磁搅拌器上，缓缓搅拌使沉淀完全溶解。

D.6.2.3 转动三通阀与水样瓶联通，以20 cm³/min～30 cm³/min的流量吸入比色池，在波长456 nm下进行分光测定。20秒～30秒后吸光值稳定时，记录吸光值A_w(参见表E.13)。

D.6.3 浊度校正

在按D.6.2.3测得水样的吸光值后，转动三通阀与蒸馏水桶联通，继续搅拌水样，并加入0.5 cm³硫代硫酸钠溶液(见D.3.4)，使碘分子颜色消失，再转动三通阀与水样瓶联通，将水样吸入比色池进行光度测定，稳定后记录水样浊度吸光值A_t(参见表E.13)。

D.6.4 试剂空白测定

由试剂反加法的分光测定求得试剂空白值：

水样瓶注满水，依次加入0.5 cm³硫酸溶液(见D.3.3)，0.5 cm³碱性碘化钠/叠氮化钠溶液(见D.3.2)，混匀后，加入一颗搅拌子，置于电磁搅拌器上，加入0.5 cm³氯化锰溶液(见D.3.1)，搅拌均匀后，吸入比色池进行光度测定。吸光值稳定后记录试剂空白吸光值A_b(参见表E.13)。

D.7 计算

溶解氧吸光值按式(D.1)校正:

$$A_c = A_w - A_t - A_b \quad \cdots\cdots(\text{D.1})$$

式中:

A_c——校正后的水样溶解氧吸光值;

A_w——水样测得的吸光值;

A_t——水样浊度吸光值;

A_b——试剂空白吸光值。

溶解氧浓度按式(D.2)计算:

$$c(\text{O}) = A_c \times \frac{V_b + V_3}{K \times b \times (V_b - V_1 - V_2)} - [\text{O}]_T \quad \cdots\cdots(\text{D.2})$$

式中:

$c(\text{O})$——水样溶解氧浓度,单位为微摩尔每立方分米(μmol/dm^3);

A_c——校正后的水样吸光值;

V_b——水样瓶体积,单位为立方厘米(cm^3);

V_1——加入氯化锰溶液体积,单位为立方厘米(cm^3);

V_2——加入碱性碘化钠/叠氮化钠溶液体积,单位为立方厘米(cm^3);

V_3——加入硫酸溶液体积,单位为立方厘米(cm^3);

b——比色池长度,单位为厘米(cm),本法中 b=1 cm;

K——校准系数(见 D.8);

$[\text{O}]_T$——试剂含氧量的扣除值,单位为微摩尔每立方分米(μmol/dm^3,本法中的经验值为 1.0 μmol/dm^3)。

D.8 求校准 K 值的方法

标准系数 K 值,采用碘酸根添加法进行校正,其操作步骤如下:

将 12 个水样瓶(每瓶均标有体积 V_b)分为四组,每组 3 个。按 4.4.3.16 的取样方法用饱和蒸馏水装满水样瓶,分别加入 0.5 cm^3 硫酸溶液(见 D.3.3)、0.5 cm^3 碱性碘化钾/叠氮化钠溶液(见 D.3.2),盖上瓶盖,颠倒数次混合,打开瓶盖,各放入一颗搅拌子,置于电磁搅拌器上缓慢搅拌,均匀后加入 0.5 cm^{3}氯化锰溶液(见 D.3.1),再搅拌均匀。每组水样瓶分别加入 0.00 cm^3,1.00 cm^3,2.00 cm^3,3.00 cm^{3}碘酸钾标准溶液(见 D.3.5),混合后吸入比色池测定添加吸光值 A_s,其中,零浓度为空白吸光值 A_b。将数据记录于溶解氧测定(分光光度法)K 值记录表(参见表 E.14)中。

K 值可按式(D.3)计算:

$$K = \frac{A_s \times (V_b + V_1 + V_a)/(V_b + V_1) - A_b}{3 \times b \times c(\text{KIO}_3) \times V_a/(V_b + V_1)} \quad \cdots\cdots(\text{D.3})$$

式中:

K——校准系数;

A_s——添加碘酸钾(KIO_3)后的吸光值;

A_b——空白吸光值;

$c(\text{KIO}_3)$——碘酸钾浓度,单位为微摩尔每立方分米(μmol/dm^3);

V_b——水样瓶体积,单位为立方厘米(cm^3);

V_1——加入氯化锰溶液体积,单位为立方厘米(cm^3);

V_a——加入碘酸钾标准溶液体积,单位为立方厘米(cm^3);

b——比色池长度,单位为厘米(cm,本法中 b=1 cm)。

求出的平均 K 值记录于溶解氧测定(分光光度法)记录表(参见表 E.13)中。

附　录　E
（资料性附录）
记录表格式

表 E.1～表 E.17 中给出了采集样品的登记表、测定记录表和数据报表的格式。

表 E.1　水样登记表

编号＿＿＿＿＿＿＿＿　　　　　　　　　　第＿＿页，共＿＿页
调查项目名称＿＿＿＿＿＿＿＿＿＿＿＿　　　　　代码＿＿＿＿＿＿
航次＿＿＿＿＿＿＿＿　调查海区＿＿＿＿＿＿＿＿　海况说明＿＿＿＿＿＿＿＿
调查船＿＿＿＿＿＿＿＿＿＿　采样日期＿＿＿年＿＿月＿＿日　至＿＿＿年＿＿月＿＿日

序号	站号	经度	纬度	采样时间	站位水深 m	采样深度 m	水温	水样瓶号						
								溶解氧		pH	营养盐			
1														
2														
3														
4														
5														
6														
7														
8														
9														
10														
11														
12														
13														
14														
15														
16														
17														
18														
19														
20														
21														
22														

记录者＿＿＿＿＿　校对者＿＿＿＿＿

表 E.2 溶解氧测定(碘量滴定法)记录表

水样登记表编号____至____,采样日期________年____月____日至________年____月____日　　　编号____________

水样接收人________,接收日期______年____月____日,分析日期________年____月____日　　　共____页,第____页

序号	站号	采样时间<br>时、分	采样深度<br>m	样品 1				样品 2				c (O)<br>平均值<br>μmol/dm^3	水温<br>℃	盐度	饱和浓度<br>μmol/dm^3	饱和度<br>%
				瓶号	容积<br>cm^3	消耗 $Na_2S_2O_3$<br>体积,cm^3	c (O)<br>μmol/dm^3	瓶号	容积<br>cm^3	消耗 $Na_2S_2O_3$<br>体积,cm^3	c (O)<br>μmol/dm^3					
1																
2																
3																
4																
5																
6																
7																
8																
9																
10																
11																
12																

硫代硫酸钠溶液标定

$$c(Na_2S_2O_3)=\frac{V_1}{V_2}\times c(KIO_3)$$

KIO_3 标准溶液

$c(KIO_3)=1.000\times10^4$ μmol/dm^3

体积(V_1)=__________(cm^3)

标定消耗 $Na_2S_2O_3$ 体积(V_2)

(1)__________(cm^3)

(2)__________(cm^3)

平均__________(cm^3)

硫代硫酸钠浓度 $c(Na_2S_2O_3)$

(1)__________(μmol/dm^3)

(2)__________(μmol/dm^3)

平均__________(μmol/dm^3)

备注:标定日期__________

有效使用期________

分析者__________　　校对者__________

表 E.3　pH 测定记录表

水样登记表编号________________至________________　　编号________________
采样日期________年____月____日至________年____月____日　　共______页，第______页
水样接收人____________　接收日期________年____月____日　　分析日期________年____月____日

序号	站号	采样时间 时、分	采样深度 m	瓶号	现场水温 ℃	测定时水温 ℃	pH_m			$t_m - t_w$ ℃	$\alpha(t_m - t_w)$	β	β_d	pH_w
							1	2	平均					
1														
2														
3														
4														
5														
6														
7														
8														
9														
10														
11														
12														
13														
14														
15														
16														
17														
18														
19														
20														

pH 计校准	时间________室温 t_m ________℃<br>pHs：(1)________　(2)________<br>序号________至________	注：pH_m 一栏记录双样测定 pH 值及其平均值。

分析者__________　校对者__________

表 E.4　总碱度测定记录表

水样登记表编号＿＿＿＿＿＿＿＿至＿＿＿＿＿＿＿＿　　编号＿＿＿＿＿＿＿＿

采样日期＿＿＿＿年＿＿月＿＿日至＿＿＿＿年＿＿月＿＿日　　共＿＿＿页，第＿＿＿页

水样接收人＿＿＿＿＿＿　接收日期＿＿＿＿年＿＿月＿＿日　　分析日期＿＿＿＿年＿＿月＿＿日

序号	站号	采样时间<br>时、分	水样<br>深度<br>m	瓶号	pH 值			补加的酸或<br>海水体积<br>cm^3		α_{H^+}	S	f_{H^+}	A<br>$mmol/dm^3$
					1	2	平均	酸	海水				
1													
2													
3													
4													
5													
6													
7													
8													
9													
10													
11													
12													
13													
14													
15													
16													
17													
18													

说明：pH_m 一栏记录双样测定值及其平均值。

盐酸标准溶液：$c(HCl)=$

分析者＿＿＿＿＿＿　校对者＿＿＿＿＿＿

表 E.5 （　　）标准曲线数据记录表

标准曲线绘制日期________年____月____日　　　　编号________________

序　　号	1		2		3		4		5		6	
添加标准使用溶液体积　cm^3												
标准系列浓度　c_s，$\mu mol/dm^3$												
吸光值　A_s												
平均吸光值　$\overline{A}_s$												
$A_n=\overline{A}_s-A_b$												

备注：

标准使用溶液浓度：________________

仪器型号：________________

测定波长：________________ nm

比色皿：________________ cm

A_n 为扣除空白吸光值 A_b 后，各标准溶液吸光值，即

$A_n=\overline{A}_s-A_b$；

标准曲线回归方程：

1）截距 $a=$

2）斜率 $b=$

相关系数：

附标准曲线图

绘制者__________　校对者__________

表 E.6 活性硅酸盐测定记录表

水样登记表编号________至________ 编号________
采样日期____年__月__日至____年__月__日 共___页,第___页
水样接收人______ 接收日期____年__月__日 分析日期____年__月__日

序号	站号	采样时间<br>时、分	采样<br>深度<br>m	瓶号	吸光值			盐度	$c(SiO_3^{2-}-Si)$<br>μmol/dm³	备注
					A_w		$\overline{A}_w$			
					(1)	(2)				
1										1) 标准曲线数据记录表<br>编号：<br>标准曲线斜率<br>b：<br>标准曲线截距<br>a：<br>2) 试剂空白检验<br>A_b：<br>3) 标准曲线校正<br>标准样浓度：<br>C_s：<br>标准样吸光值<br>A_s：<br>4) A_w 及 $\overline{A}_w$ 分别为双样吸光值及其平均值。
2										
3										
4										
5										
6										
7										
8										
9										
10										
11										
12										
13										
14										
15										
16										
17										
18										
19										
20										
21										

分析者________ 校对者________

表 E.7 活性磷酸盐测定记录表

水样登记表编号________________至________________ 编号________________

采样日期________年____月____日至________年____月____日 共______页，第______页

水样接收人____________ 接收日期________年____月____日 分析日期________年____月____日

序号	站号	采样时间<br>时、分	采样<br>深度<br>m	瓶号	吸光值			$c(PO_4^{3-}\text{-}P)$<br>$\mu mol/dm^3$	备注
					A_w		$\overline{A}_w$		
					(1)	(2)			
1									1) 标准曲线数据记录表<br>编号：
2									
3									标准曲线斜率<br>b：
4									
5									标准曲线截距<br>a：
6									
7									2) 试剂空白检验<br>A_b：
8									
9									3) 标准曲线校正<br>标准样浓度：<br>C_s：
10									
11									标准样吸光值<br>A_s：
12									
13									4) A_w 及 $\overline{A}_w$ 分别为双样测定吸光值及其平均值。
14									
15									
16									
17									
18									
19									
20									
21									

分析者__________ 校对者__________

表 E.8　亚硝酸盐测定记录表

水样登记表编号________________至________________　　　　编号________________

采样日期________年____月____日至________年____月____日　　　　共______页，第______页

水样接收人____________　接收日期________年____月____日　　　　分析日期________年____月____日

序号	站号	采样时间<br>时、分	水样<br>深度<br>m	瓶号	吸光值			$c(NO_2^- -N)$<br>μmol/dm³	备注
					A_w		$\overline{A}_w$		
					(1)	(2)			
1									1) 标准曲线数据记录表
2									编号：
3									标准曲线斜率
4									b：
5									标准曲线截距
6									a：
7									2) 试剂空白检验<br>A_b：
8									
9									3) 标准曲线校正<br>标准样浓度：
10									C_s：
11									标准样吸光值
12									A_s：
13									4) A_w 及 $\overline{A}_w$ 分别为双样
14									测定吸光值及其平均值。
15									
16									
17									
18									
19									
20									
21									

分析者__________　校对者__________

表 E.9　硝酸盐测定记录表

水样登记表编号________至________　　编号________
采样日期____年____月____日至____年____月____日　　共____页，第____页
水样接收人________　接收日期____年____月____日　　分析日期____年____月____日

序号	站号	采样时间 时、分	水样深度 m	瓶号	吸光值			$\overline{A}_{NO_2^- -N}$	$X \cdot \overline{A}_{NO_2^- -N}$	$c(NO_3^- -N)$ μmol/dm³	备注
					A_w		$\overline{A}_w$				
					(1)	(2)					
1											1）标准曲线数据记录表<br>编号：____<br>标准曲线斜率<br>b：<br>标准曲线截距<br>a：<br>2）硝酸盐和亚硝酸盐比色池长度比<br>X ____<br>3）试剂空白检验<br>A_b：<br>4）标准曲线校正<br>标准样浓度：<br>C_s：<br>标准样吸光值<br>A_s：<br>5）A_w 及 $\overline{A}_w$ 分别为双样测定吸光值及其平均值。
2											
3											
4											
5											
6											
7											
8											
9											
10											
11											
12											
13											
14											
15											
16											
17											
18											
19											
20											

分析者________　校对者________

表 E.10 铵盐测定(次溴酸钠氧化法)记录表

水样登记表编号________________至________________　　　　　　编号________________

采样日期________年____月____日至________年____月____日　　　　　　共______页，第______页

水样接收人____________　接收日期________年____月____日　　　　　　分析日期________年____月____日

序号	站号	采样时间<br>时、分	水样<br>深度<br>m	瓶号	吸光值<br>A_w<br>(1)	 <br>(2)	 $\bar{A}_w$	$\bar{A}_{NO_2^--N}$	$c(NH_4^+-N)$<br>$\mu mol/dm^3$	备注
1										1) 标准曲线数据记录表编号：<br><br>标准曲线斜率 b：<br><br>标准曲线截距 a：<br><br>2) 试剂空白检验 A_b：<br><br>3) 标准曲线校正<br>标准样浓度：<br>C_s：<br><br>标准样吸光值<br>A_s：<br><br>4) A_w 及 $\bar{A}_w$ 分别为双样测定吸光值及其平均值。
2										
3										
4										
5										
6										
7										
8										
9										
10										
11										
12										
13										
14										
15										
16										
17										
18										
19										
20										
21										

分析者__________　校对者__________

表 E.11 氯化物测定记录表

水样登记表编号________至________ 编号________

采样日期____年__月__日至____年__月__日 共___页，第___页

水样接收人______ 接收日期____年__月__日 分析日期____年__月__日

序号	站号	采样时间<br>时、分	水样<br>深度<br>m	瓶号	测定样<br>体积<br>cm^3	消耗氯化银体积<br>cm^3			ρ_{Cl}<br>g/dm^3	备 注
						V_w		$\overline{V}_w$		
						(1)	(2)			
1										硝酸溶液标定：<br>氯化钠标准溶液氯离子浓度<br>ρ=________ g/dm^3；<br><br>氯化钠标准溶液体积<br>V_1=________ cm^3<br><br>消耗硝酸银体积 V_S<br>(1)=________ cm^3<br>(2)=________ cm^3<br>(3)=________ cm^3<br>$\overline{V}_s$=________ cm^3<br><br>注：V_w 与 $\overline{V}_w$ 分别为双样测定消耗硝酸银体积及其平均值
2										
3										
4										
5										
6										
7										
8										
9										
10										
11										
12										
13										
14										
15										
16										
17										
18										
19										
20										
21										
22										

分析者________ 校对者________

表 E.12　铵盐测定(靛酚蓝法)记录表

<table>
<tr><td colspan="10">水样登记表编号________至________　　　　　　编号________
采样日期____年___月___日至____年___月___日　　　　　　共___页,第___页
水样接收人______　接收日期____年___月___日　　　　分析日期____年___月___日</td></tr>
<tr><td rowspan="3">序号</td><td rowspan="3">站号</td><td rowspan="3">采样时间
时、分</td><td rowspan="3">水样
深度
m</td><td rowspan="3">瓶号</td><td colspan="3">吸 光 值</td><td rowspan="3">$c(NH_4^+\text{-}N)$
$\mu mol/dm^3$</td><td rowspan="3">备　注</td></tr>
<tr><td colspan="2">A_w</td><td rowspan="2">$\overline{A}_w$</td></tr>
<tr><td>(1)</td><td>(2)</td></tr>
<tr><td>1</td><td></td><td></td><td></td><td></td><td></td><td></td><td></td><td></td><td rowspan="23">1) 标准曲线数据记录表编号:

标准曲线斜率
b:

标准曲线截距
a:

2) 试剂空白检验
A_b:

3) 标准曲线校正
标准样浓度
C_s:

标准样吸光值
A_s:

4) A_w 及 $\overline{A}_w$ 分别为双样测定吸光值及其平均值。</td></tr>
<tr><td>2</td><td></td><td></td><td></td><td></td><td></td><td></td><td></td><td></td></tr>
<tr><td>3</td><td></td><td></td><td></td><td></td><td></td><td></td><td></td><td></td></tr>
<tr><td>4</td><td></td><td></td><td></td><td></td><td></td><td></td><td></td><td></td></tr>
<tr><td>5</td><td></td><td></td><td></td><td></td><td></td><td></td><td></td><td></td></tr>
<tr><td>6</td><td></td><td></td><td></td><td></td><td></td><td></td><td></td><td></td></tr>
<tr><td>7</td><td></td><td></td><td></td><td></td><td></td><td></td><td></td><td></td></tr>
<tr><td>8</td><td></td><td></td><td></td><td></td><td></td><td></td><td></td><td></td></tr>
<tr><td>9</td><td></td><td></td><td></td><td></td><td></td><td></td><td></td><td></td></tr>
<tr><td>10</td><td></td><td></td><td></td><td></td><td></td><td></td><td></td><td></td></tr>
<tr><td>11</td><td></td><td></td><td></td><td></td><td></td><td></td><td></td><td></td></tr>
<tr><td>12</td><td></td><td></td><td></td><td></td><td></td><td></td><td></td><td></td></tr>
<tr><td>13</td><td></td><td></td><td></td><td></td><td></td><td></td><td></td><td></td></tr>
<tr><td>14</td><td></td><td></td><td></td><td></td><td></td><td></td><td></td><td></td></tr>
<tr><td>15</td><td></td><td></td><td></td><td></td><td></td><td></td><td></td><td></td></tr>
<tr><td>16</td><td></td><td></td><td></td><td></td><td></td><td></td><td></td><td></td></tr>
<tr><td>17</td><td></td><td></td><td></td><td></td><td></td><td></td><td></td><td></td></tr>
<tr><td>18</td><td></td><td></td><td></td><td></td><td></td><td></td><td></td><td></td></tr>
<tr><td>19</td><td></td><td></td><td></td><td></td><td></td><td></td><td></td><td></td></tr>
<tr><td>20</td><td></td><td></td><td></td><td></td><td></td><td></td><td></td><td></td></tr>
<tr><td>21</td><td></td><td></td><td></td><td></td><td></td><td></td><td></td><td></td></tr>
<tr><td>22</td><td></td><td></td><td></td><td></td><td></td><td></td><td></td><td></td></tr>
<tr><td>23</td><td></td><td></td><td></td><td></td><td></td><td></td><td></td><td></td></tr>
</table>

分析者________　校对者________

表 E.13 溶解氧测定（分光光度法）记录表

水样登记表编号__________至__________ 编号__________
采样日期_____年___月___日至_____年___月___日 共____页，第____页
水样接收人_______ 接收日期_____年___月___日 分析日期_____年___月___日

序号	站号	采样时间<br>时、分	水样<br>深度<br>m	瓶号	容积<br>cm^3	总吸<br>光值<br>A_w	试剂<br>空白<br>吸光值<br>A_b	浊度<br>吸光值<br>A_t	校正后<br>吸光值<br>A_c	溶解氧<br>c(O)<br>$\mu mol/dm^3$	备　注
1											*K* 值记录表<br>编号______<br>*K*=
2											
3											
4											
5											
6											
7											
8											
9											
10											
11											
12											
13											
14											
15											
16											
17											
18											
19											
20											

分析者_______ 校对者_______

表 E.14 溶解氧测定(分光光度法)K 值记录表

K 值测定日期________年____月____日									编号__________________			
	1			2			3			4		
水样瓶体积 V_b<br>cm^3												
添加 $MnCl_2$ 体积 V_1<br>cm^3												
添加 KIO_3 体积 V_a<br>cm^3	—											
V_b+V_1<br>cm^3	—											
$V_b+V_1+V_a$<br>cm^3	—											
吸光值 A_b				—	—	—	—	—	—	—	—	—
吸光值 A_s												
K 值	—											
平均 K 值	—											
总平均 K 值	—											
备　注	标准碘酸钾浓度 $c\,(KIO_3)=$__________________											

测定者__________　校对者__________

表 E.15 总磷测定记录表

水样登记表编号________________至________________ 编号________________

采样日期________年____月____日至________年____月____日 共______页，第______页

水样接收人____________ 接收日期________年____月____日 分析日期________年____月____日

序号	站号	采样时间<br>时、分	水样<br>深度<br>m	瓶号	吸光值			浊度<br>A_t	总磷浓度<br>c(TP-P)<br>μmol/dm³	备注
					A_w		$\overline{A}_w$			
					(1)	(2)				
1										1）标准曲线数据记录表编号：<br>标准曲线斜率<br>b：<br>标准曲线截距<br>a：<br>2）试剂空白检验<br>A_b：<br>3）标准曲线校正<br>标准样浓度<br>C_s：<br>标准样吸光值<br>A_s：<br>4）A_w 及 $\overline{A}_w$ 分别为双样测定吸光值及其平均值。
2										
3										
4										
5										
6										
7										
8										
9										
10										
11										
12										
13										
14										
15										
16										
17										
18										
19										
20										

分析者__________ 校对者__________

表 E.16 总氮测定记录表

水样登记表编号__________至__________ 编号__________

采样日期_____年___月___日至_____年___月___日 共____页，第____页

水样接收人_______ 接收日期_____年___月___日 分析日期_____年___月___日

序号	站号	采样时间<br>时、分	水样<br>深度<br>m	瓶号	吸光值			浊度<br>A_t	总氮浓度<br>c(TN-N)<br>μmol/dm^3	备注
					A_w		$\overline{A}_w$			
					(1)	(2)				
1										1) 标准曲线数据记录表编号：<br>标准曲线斜率 b：<br>标准曲线截距 a：<br>2) 试剂空白检验 A_b：<br>3) 标准曲线校正<br>标准样浓度：<br>C_s：<br>标准样吸光值<br>A_s：<br>4) A_w 及 $\overline{A}_w$ 分别为双样测定吸光值及其平均值。
2										
3										
4										
5										
6										
7										
8										
9										
10										
11										
12										
13										
14										
15										
16										
17										
18										
19										
20										
21										

分析者________ 校对者________

表 E.17　海水化学观测数据报表

调查项目名称＿＿＿＿＿＿　代码＿＿＿＿＿＿　编码＿＿＿＿＿＿

调查海区＿＿＿＿＿＿　航次＿＿＿＿，采样日期＿＿年＿＿月＿＿日至＿＿年＿＿月＿＿日　共＿＿页，第＿＿页

数据来源 1)：DO ＿＿，pH ＿＿，A ＿＿，SiO_3^{2-}-Si ＿＿，PO_4^{3-}-P ＿＿，NO_2^--N ＿＿ NO_3^--N ＿＿，NH_4^+-N ＿＿，Cl ＿＿，TP ＿＿，TN ＿＿

序号	站位号	水深 m	采样深度 m	DO μmol/dm³	pH	A mmol/dm³	SiO_3^{2-}-Si μmol/dm³	PO_4^{3-}-P μmol/dm³	NO_2^--N μmol/dm³	NO_3^--N μmol/dm³	NH_4^+-N μmol/dm³	Cl g/dm³	TP μmol/dm³	TN μmol/dm³	备　注
1															1) 填写测定记录表编号
2															
3															
4															
5															
6															
7															
8															
9															
10															
11															
12															

抄录者＿＿＿＿　校对者＿＿＿＿

附 录 F
（资料性附录）
测定结果计算用表

表 F.1～表 F.8 给出了海水溶解氧、pH 值测定中有关参数和换算用表，元素的相对原子质量表，容积校正表。

表 F.1 空气中氧在不同温度和盐度海水中的饱和浓度 单位为微摩尔每立方分米

t/℃	S									
	0.0	1.0	2.0	3.0	4.0	5.0	6.0	7.0	8.0	9.0
0.0	912	906	899	893	887	881	875	869	863	857
1.0	887	881	875	869	863	857	851	845	840	834
2.0	863	857	851	845	840	834	828	823	817	812
3.0	840	834	828	823	817	812	807	801	796	791
4.0	818	812	807	801	796	791	786	780	775	770
5.0	797	791	786	781	776	771	766	761	756	751
6.0	776	771	766	761	756	752	747	742	737	732
7.0	757	752	747	743	738	733	728	724	719	714
8.0	739	734	729	725	720	715	711	706	702	697
9.0	721	717	712	707	703	698	694	690	685	681
10.0	704	700	695	691	686	682	678	674	669	665
11.0	688	684	679	675	671	667	662	658	654	650
12.0	672	668	664	660	656	652	647	643	639	635
13.0	657	653	649	645	641	637	633	629	625	621
14.0	643	639	635	631	627	623	619	616	612	608
15.0	629	625	621	617	614	610	606	602	599	595
16.0	616	612	608	604	601	597	593	590	586	583
17.0	603	599	595	592	588	585	581	578	574	571
18.0	590	587	583	580	576	573	569	566	563	559
19.0	578	575	571	568	565	561	558	555	551	548
20.0	567	563	560	557	554	550	547	544	541	537
21.0	556	552	549	546	543	540	536	533	530	527
22.0	545	542	539	535	532	529	526	523	520	517
23.0	534	531	528	525	522	519	516	513	510	507
24.0	524	521	518	516	513	510	507	504	501	498
25.0	515	512	509	506	503	500	497	495	492	489
26.0	505	503	500	497	494	491	489	486	483	480
27.0	496	494	491	488	485	483	480	477	475	472
28.0	488	485	482	479	477	474	471	469	466	464
29.0	479	476	474	471	469	466	463	461	458	456
30.0	471	468	466	463	461	458	455	453	451	448
31.0	463	460	458	455	453	450	448	445	443	441
32.0	455	453	450	448	445	443	440	438	436	433
33.0	447	445	443	440	438	436	433	431	429	426
34.0	440	438	435	433	431	429	426	424	422	419
35.0	433	431	428	426	424	422	419	417	415	413

表 F.1（续） 单位为微摩尔每立方分米

t/℃	S									
	10.0	11.0	12.0	13.0	14.0	15.0	16.0	17.0	18.0	19.0
0.0	852	846	840	834	829	823	817	812	806	801
1.0	828	823	817	812	806	801	795	790	785	779
2.0	806	801	796	790	785	780	774	769	764	759
3.0	785	780	775	770	765	759	754	749	744	739
4.0	765	760	755	750	745	740	735	730	726	721
5.0	746	741	736	731	726	722	717	712	708	703
6.0	727	723	718	713	709	704	699	695	690	686
7.0	710	705	700	696	691	687	683	678	674	669
8.0	693	688	684	679	675	671	666	662	658	654
9.0	676	672	668	664	659	655	651	647	643	639
10.0	661	657	652	648	644	640	636	632	628	624
11.0	646	642	638	634	630	626	622	618	614	610
12.0	631	628	624	620	616	612	608	604	601	597
13.0	618	614	610	606	602	599	595	591	588	584
14.0	604	601	597	593	590	586	582	579	575	572
15.0	592	588	584	581	577	574	570	567	563	560
16.0	579	576	572	569	565	562	558	555	552	548
17.0	567	564	561	557	554	550	547	544	541	537
18.0	556	553	549	546	543	539	536	533	530	527
19.0	545	542	538	535	532	529	526	523	520	516
20.0	534	531	528	525	522	519	516	513	510	507
21.0	524	521	518	515	512	500	506	503	500	497
22.0	514	511	508	505	502	499	496	494	491	488
23.0	504	502	499	496	493	490	487	484	482	479
24.0	495	492	490	487	484	481	478	476	473	470
25.0	486	484	481	478	475	473	470	467	465	462
26.0	478	475	472	470	467	464	462	459	456	454
27.0	469	467	464	461	459	456	454	451	449	446
28.0	461	459	456	453	451	448	446	443	441	439
29.0	453	451	448	446	443	441	438	436	434	431
30.0	446	443	441	438	436	434	431	429	426	424
31.0	438	436	433	431	429	426	424	422	419	417
32.0	431	429	426	424	422	419	417	415	413	411
33.0	424	422	419	417	415	413	411	408	406	404
34.0	417	415	413	411	408	406	404	402	400	398
35.0	411	408	406	404	402	400	398	396	394	392

表 F.1（续）　　单位为微摩尔每立方分米

t/℃	S									
	20.0	21.0	22.0	23.0	24.0	25.0	26.0	27.0	28.0	29.0
0.0	795	790	785	779	774	769	763	758	753	748
1.0	774	769	764	759	753	748	743	738	733	728
2.0	754	749	744	739	734	729	724	719	714	710
3.0	735	730	725	720	715	710	706	701	696	692
4.0	716	711	707	702	697	693	688	683	679	674
5.0	698	694	689	685	680	676	671	667	662	658
6.0	681	677	672	668	664	659	655	651	647	642
7.0	665	661	656	652	648	644	640	635	631	627
8.0	649	645	641	637	633	629	625	621	617	613
9.0	635	630	626	622	618	615	611	607	603	599
10.0	620	616	612	608	605	601	597	593	589	586
11.0	606	603	599	595	591	588	584	580	577	573
12.0	593	589	586	582	578	575	571	568	564	561
13.0	580	577	573	570	566	563	559	556	552	549
14.0	568	565	561	558	554	551	548	544	541	537
15.0	556	553	550	546	543	540	536	533	530	527
16.0	545	542	538	535	532	529	526	522	519	516
17.0	534	531	528	525	521	518	515	512	509	506
18.0	524	520	517	514	511	508	505	502	499	496
19.0	513	510	507	504	501	498	495	492	490	487
20.0	504	501	498	495	492	489	486	483	480	478
21.0	494	491	488	486	483	480	477	474	472	469
22.0	485	482	479	477	474	471	468	466	463	460
23.0	476	473	471	468	465	463	460	457	455	452
24.0	468	465	462	460	457	454	452	449	447	444
25.0	459	457	454	452	449	446	444	441	439	436
26.0	451	449	446	444	441	439	436	434	431	429
27.0	444	441	439	436	434	431	429	427	424	422
28.0	436	434	431	429	426	424	422	419	417	415
29.0	429	426	424	422	419	417	415	413	410	408
30.0	422	419	417	415	413	410	408	406	404	401
31.0	415	413	410	408	406	404	402	399	397	395
32.0	408	406	404	402	400	397	395	393	391	389
33.0	402	400	398	395	393	391	389	387	385	383
34.0	396	393	391	389	387	385	383	381	379	377
35.0	389	387	385	383	381	379	377	375	373	371

表 F.1（续）

单位为微摩尔每立方分米

t/℃	S									
	30.0	31.0	32.0	33.0	34.0	35.0	36.0	37.0	38.0	39.0
0.0	743	738	733	728	723	718	713	708	703	699
1.0	723	718	714	709	704	699	695	690	685	681
2.0	705	700	695	691	686	681	677	672	668	663
3.0	687	682	678	673	669	664	660	656	651	647
4.0	670	666	661	657	652	648	644	640	635	631
5.0	654	649	645	641	637	633	628	624	620	616
6.0	638	634	630	626	622	618	614	610	606	602
7.0	623	619	615	611	607	603	599	596	592	588
8.0	609	605	601	597	593	590	586	582	578	575
9.0	595	591	588	584	580	576	573	569	565	562
10.0	582	578	575	571	567	564	560	557	553	550
11.0	569	566	562	559	555	552	548	545	541	538
12.0	557	554	550	547	543	540	537	533	530	527
13.0	545	542	539	535	532	529	525	522	519	516
14.0	534	531	528	524	521	518	515	512	508	505
15.0	523	520	517	514	511	508	504	501	498	495
16.0	513	510	507	504	501	498	495	492	489	486
17.0	503	500	497	494	491	488	485	482	479	476
18.0	493	490	487	484	481	479	476	473	470	467
19.0	484	481	478	475	472	470	467	464	461	459
20.0	475	472	469	466	464	461	458	456	453	450
21.0	466	463	461	458	455	453	450	447	445	442
22.0	458	455	452	450	447	444	442	439	437	434
23.0	449	447	444	442	439	437	434	432	429	427
24.0	442	439	437	434	432	429	427	424	422	419
25.0	434	431	429	427	424	422	419	417	415	412
26.0	427	424	422	419	417	415	412	410	408	405
27.0	419	417	415	412	410	408	406	403	401	399
28.0	412	410	408	406	403	401	399	397	394	392
29.0	406	404	401	399	397	395	393	390	388	386
30.0	399	397	395	393	391	388	386	384	382	380
31.0	393	391	389	387	384	382	380	378	376	374
32.0	387	385	383	381	378	376	374	372	370	368
33.0	381	379	377	375	373	371	369	367	365	363
34.0	375	373	371	369	367	365	363	361	359	357
35.0	369	367	365	364	362	360	358	356	354	352

注：本表由 5.7.3 的公式(3)求得。

表 F.2　标准缓冲溶液的 pH 值

水温/℃	邻苯二甲酸氢钾<br>0.050 0 mol/dm^3	混合磷酸盐(1∶1)<br>KH_2PO_4,0.025 0 mol/dm^3<br>Na_2HPO_4,0.025 0 mol/dm^3	十水四硼酸钠<br>0.010 0 mol/dm^3
0	4.006	6.981	9.458
5	3.999	6.949	9.391
10	3.999	6.921	9.330
15	3.996	6.898	9.276
20	3.998	6.879	9.220
25	4.003	6.864	9.182
30	4.010	6.852	9.142
35	4.019	6.844	9.105
40	4.029	6.838	9.072
45	4.042	6.834	9.042

表 F.3　pH 测定的温度校正值 $\alpha(t_m - t_w)$ 表

$(t_m - t_w)$ ℃	pH											
	7.5	7.6	7.7	7.8	7.9	8.0	8.1	8.2	8.3	8.4	8.5	8.6
1	0.01	0.01	0.01	0.01	0.01	0.01	0.01	0.01	0.01	0.01	0.01	0.01
2	0.02	0.02	0.02	0.02	0.02	0.02	0.02	0.02	0.02	0.02	0.02	0.02
3	0.03	0.03	0.03	0.03	0.03	0.03	0.03	0.03	0.03	0.03	0.03	0.04
4	0.03	0.03	0.04	0.04	0.04	0.04	0.04	0.04	0.04	0.05	0.05	0.05
5	0.04	0.04	0.04	0.05	0.05	0.05	0.05	0.05	0.06	0.06	0.06	0.06
6	0.05	0.05	0.05	0.06	0.06	0.06	0.06	0.06	0.07	0.07	0.07	0.07
7	0.06	0.06	0.06	0.07	0.07	0.07	0.07	0.07	0.08	0.08	0.08	0.08
8	0.07	0.07	0.07	0.07	0.08	0.08	0.08	0.08	0.09	0.09	0.09	0.10
9	0.07	0.08	0.08	0.08	0.09	0.09	0.09	0.10	0.10	0.10	0.10	0.11
10	0.08	0.09	0.09	0.09	0.10	0.10	0.10	0.11	0.11	0.11	0.12	0.12
11	0.09	0.09	0.10	0.10	0.11	0.11	0.11	0.12	0.12	0.12	0.13	0.13
12	0.10	0.10	0.11	0.11	0.12	0.12	0.12	0.13	0.13	0.14	0.14	0.14
13	0.11	0.11	0.12	0.12	0.12	0.13	0.13	0.14	0.14	0.15	0.15	0.16
14	0.12	0.12	0.13	0.13	0.13	0.14	0.14	0.15	0.15	0.16	0.16	0.17
15	0.13	0.13	0.14	0.14	0.14	0.15	0.15	0.16	0.16	0.17	0.17	0.18
16	0.13	0.14	0.14	0.15	0.15	0.16	0.16	0.17	0.18	0.18	0.19	0.19
17	0.14	0.15	0.15	0.16	0.16	0.17	0.18	0.18	0.19	0.19	0.20	0.20
18	0.14	0.15	0.16	0.17	0.17	0.18	0.19	0.19	0.20	0.20	0.21	0.22
19	0.15	0.16	0.17	0.18	0.18	0.19	0.20	0.20	0.21	0.21	0.22	0.23
20	0.16	0.17	0.18	0.19	0.19	0.20	0.21	0.21	0.22	0.23	0.23	0.24
21	0.17	0.18	0.19	0.20	0.20	0.21	0.22	0.22	0.23	0.24	0.24	0.25
22	0.18	0.19	0.20	0.20	0.21	0.22	0.23	0.23	0.24	0.25	0.26	0.26
23	0.19	0.20	0.21	0.21	0.22	0.23	0.24	0.24	0.25	0.26	0.27	0.28
24	0.20	0.21	0.22	0.22	0.23	0.24	0.25	0.25	0.26	0.27	0.28	0.29
25	0.21	0.22	0.22	0.23	0.24	0.25	0.26	0.26	0.28	0.28	0.29	0.30

表 F.4 pH 测定的压力校正系数 β 表

pH_m	7.5	7.6	7.7	7.8	7.9	8.0	8.1	8.2	8.3	8.4
$\beta\times10^6$	35	31	28	25	23	22	21	20	20	20

表 F.5 pH—a_{H^+} 换算表

V	N	V	N	V	N	V	N
0.00	1.000	0.25	0.562	0.50	0.316	0.75	0.178
0.01	0.977	0.26	0.549	0.51	0.309	0.76	0.174
0.02	0.955	0.27	0.537	0.52	0.302	0.77	0.170
0.03	0.933	0.28	0.525	0.53	0.295	0.78	0.166
0.04	0.912	0.29	0.513	0.54	0.288	0.79	0.162
0.05	0.891	0.30	0.501	0.55	0.282	0.80	0.158
0.06	0.871	0.31	0.490	0.56	0.275	0.81	0.155
0.07	0.851	0.32	0.479	0.57	0.269	0.82	0.151
0.08	0.832	0.33	0.468	0.58	0.263	0.83	0.148
0.09	0.813	0.34	0.457	0.59	0.257	0.84	0.144
0.10	0.794	0.35	0.447	0.60	0.251	0.85	0.141
0.11	0.776	0.36	0.437	0.61	0.245	0.86	0.138
0.12	0.759	0.37	0.427	0.62	0.240	0.87	0.135
0.13	0.741	0.38	0.417	0.63	0.234	0.88	0.132
0.14	0.725	0.39	0.407	0.64	0.229	0.89	0.129
0.15	0.709	0.40	0.398	0.65	0.224	0.90	0.126
0.16	0.692	0.41	0.389	0.66	0.219	0.91	0.123
0.17	0.676	0.42	0.380	0.67	0.214	0.92	0.120
0.18	0.661	0.43	0.372	0.68	0.209	0.93	0.117
0.19	0.645	0.44	0.363	0.69	0.204	0.94	0.115
0.20	0.631	0.45	0.355	0.70	0.200	0.95	0.112
0.21	0.617	0.46	0.347	0.71	0.195	0.96	0.110
0.22	0.603	0.47	0.339	0.72	0.191	0.97	0.107
0.23	0.589	0.48	0.331	0.73	0.186	0.98	0.105
0.24	0.575	0.49	0.324	0.74	0.182	0.99	0.102

注：Q 为 pH 的整数部分。表中 V 为 pH 值的小数部分，由 V 值查表得相应的 N 值，代入 $a_{H^+}=N\times10^Q$，即得氢离子活度。

表 F.6 海水氢离子活度系数 f_{H^+} 随盐度和 pH 变化

pH	S						
	3.5	7	11	14.8	18	21～33	36
2.8～2.9	0.865	0.800	0.785	0.775	0.770	0.768	0.773
3.0～3.9	0.845	0.782	0.770	0.760	0.755	0.753	0.758
4.0	0.890	0.822	0.810	0.800	0.795	0.793	0.798

表 F.7 元素的相对原子质量表(1997)

1	氢	H	1.000 79	36	氪	Kr	83.80	71	镥	Lu	174.97
2	氦	He	4.002 6	37	铷	Rb	85.468	72	铪	Hf	178.49
3	锂	Li	6.941	38	锶	Sr	87.62	73	钽	Ta	180.95
4	铍	Be	9.012 2	39	钇	Y	88.906	74	钨	W	183.84
5	硼	B	10.811	40	锆	Zr	91.224	75	铼	Re	186.21
6	碳	C	12.011	41	铌	Nb	92.906	76	锇	Os	190.23
7	氮	N	14.007	42	钼	Mo	95.94	77	铱	Ir	192.22
8	氧	O	15.999	43	锝	Tc	97.907	78	铂	Pt	195.06
9	氟	F	18.998	44	钌	Ru	101.07	79	金	Au	196.97
10	氖	Ne	20.179	45	铑	Rh	102.91	80	汞	Hg	200.59
11	钠	Na	22.990	46	钯	Pd	106.42	81	铊	Tl	204.38
12	镁	Mg	24.305	47	银	Ag	107.87	82	铅	Pb	207.2
13	铝	Al	26.982	48	镉	Cd	112.41	83	铋	Bi	208.98
14	硅	Si	28.086	49	铟	In	114.82	84	钋	Po	208.98
15	磷	P	30.974	50	锡	Sn	118.71	85	砹	At	209.99
16	硫	S	32.066	51	锑	Sb	121.76	86	氡	Rn	222.02
17	氯	Cl	35.453	52	碲	Te	127.60	87	钫	Fr	223.02
18	氩	Ar	39.948	53	碘	I	126.90	88	镭	Ra	226.03
19	钾	K	39.098	54	氙	Xe	131.29	89	锕	Ac	227.03
20	钙	Ca	40.078	55	铯	Cs	132.91	90	钍	Th	232.04
21	钪	Sc	44.956	56	钡	Ba	137.33	91	镤	Pa	231.04
22	钛	Ti	47.867	57	镧	La	138.91	92	铀	U	238.03
23	钒	V	50.942	58	铈	Ce	140.12	93	镎	Np	237.05
24	铬	Cr	51.996	59	镨	Pr	140.91	94	钚	Pu	244.06
25	锰	Mn	54.938	60	钕	Nd	144.24	95	镅	Am	243.06
26	铁	Fe	55.847	61	钷	Pm	145.91	96	锔	Cm	247.07
27	钴	Co	58.933	62	钐	Sm	150.36	97	锫	Bk	247.07
28	镍	Ni	58.693	63	铕	Eu	151.96	98	锎	Cf	251.08
29	铜	Cu	63.546	64	钆	Gd	157.25	99	锿	Es	252.08
30	锌	Zn	65.39	65	铽	Tb	158.93	100	镄	Fm	257.10
31	镓	Ga	69.723	66	镝	Dy	162.50	101	钔	Md	258.10
32	锗	Ge	72.59	67	钬	Ho	164.93	102	锘	No	259.10
33	砷	As	74.922	68	铒	Er	167.26	103	铹	Lr	260.11
34	硒	Se	78.96	69	铥	Tm	168.93				
35	溴	Br	79.904	70	镱	Yb	173.04				

注：Pm、Po、At、Rn、Fr、Ra、Ac、Np、Pu、Am、Cm、Bk、Cf、Es、Fm、Md、No、Lr 为半衰期最长同位素的相对原子质量。

表 F.8　20℃时容积为 1.000 0 dm³ 玻璃容器中的蒸馏水在不同温度时的质量(m_{20})

单位为克每立方分米

t/℃	m_{20}	t/℃	m_{20}	t/℃	m_{20}	t/℃	m_{20}	t/℃	m_{20}	t/℃	m_{20}
0	998.30	15.2	997.92	19.2	997.30	23.2	996.54	27.2	995.60	31.2	994.52
1	998.40	15.4	997.89	19.4	997.28	23.4	996.50	27.4	995.55	31.4	994.47
2	998.46	15.6	997.87	19.6	997.24	23.6	996.45	27.6	995.50	31.6	994.41
3	998.51	15.8	997.84	19.8	997.21	23.8	996.41	27.8	995.45	31.8	994.35
4	998.54	16.0	997.81	20.0	997.17	24.0	996.36	28.0	995.40	32.0	994.29
5	998.56	16.2	997.78	20.2	997.14	24.2	996.32	28.2	995.35	32.2	994.23
6	998.56	16.4	997.76	20.4	997.10	24.4	996.27	28.4	995.29	32.4	994.17
7	998.55	16.6	997.73	20.6	997.06	24.6	996.23	28.6	995.24	32.6	994.11
8	998.52	16.8	997.70	20.8	997.02	24.8	996.18	28.8	995.19	32.8	994.05
9	998.48	17.0	997.67	21.0	996.99	25.0	996.14	29.0	995.14	33.0	993.99
10	998.42	17.2	997.64	21.2	996.95	25.2	996.09	29.2	995.08	33.2	993.93
11	998.35	17.4	997.61	21.4	996.91	25.4	996.04	29.4	995.03	33.4	993.87
12	998.27	17.6	997.58	21.6	996.87	25.6	996.00	29.6	994.97	33.6	993.81
13	998.17	17.8	997.55	21.8	996.83	25.8	995.95	29.8	994.92	33.8	993.75
14	998.06	18.0	997.51	22.0	996.79	26.0	995.90	30.0	994.86	34.0	993.68
14.2	998.04	18.2	997.48	22.2	996.75	26.2	995.85	30.2	994.81	34.2	993.62
14.4	998.02	18.4	997.45	22.4	996.71	26.4	995.80	30.4	994.75	34.4	993.56
14.6	997.99	18.6	997.42	22.6	996.66	26.6	995.75	30.6	994.69	34.6	993.50
14.8	997.97	18.8	997.38	22.8	996.62	26.8	995.70	30.8	994.64	34.8	993.43
15.0	997.94	19.0	997.35	23.0	996.58	27.0	995.65	31.0	994.58	35.0	993.37

中 华 人 民 共 和 国
国 家 标 准
海洋调查规范
第 4 部分：海水化学要素调查
GB/T 12763.4—2007
*
中国标准出版社出版发行
北京复兴门外三里河北街 16 号
邮政编码：100045
网址 www.spc.net.cn
电话：68523946　68517548
中国标准出版社秦皇岛印刷厂印刷
各地新华书店经销
*
开本 880×1230　1/16　印张 4.75　字数 144 千字
2007 年 11 月第一版　2007 年 11 月第一次印刷
*

GB/T 12763.4-2007

如有印装差错　由本社发行中心调换
版权专有　侵权必究
举报电话：(010)68533533
