# Supplementary material for: Autumn ichthyoplankton assemblage in the Yangtze Estuary shaped by environmental factors
Source: PeerJ. 2016 Apr 19;4:e1922. doi: 10.7717/peerj.1922 (PMC4841249; doi:10.7717/peerj.1922)
Supplement: Supplemental Information 1 [file peerj-04-1922-s001.zip › GB12763-2007/GB¿MT 12763.9-2007 ║ú╤≤╡≈▓Θ╣μ╖╢ ╡┌09▓┐╖╓ú║║ú╤≤╔·╠1⁄4╡≈▓Θ╓╕─╧.pdf]

ICS 01.140.20; 07.060
A 45

中华人民共和国国家标准

GB/T 12763.9—2007

海洋调查规范
第 9 部分：海洋生态调查指南

Specifications for oceanographic survey
Part 9: Guidelines for marine ecological survey

2007—08—13 发布　　2008—02—01 实施

中华人民共和国
国家质量监督检验检疫总局　发布

目　次

前　言 III
引　言 IV
1 范围 1
2 规范性引用文件 1
3 术语和定义 1
4 一般规定 3
4.1 技术设计和调查计划编制 3
4.2 调查内容 4
4.3 海上调查作业一般规定 5
4.4 质量控制管理 5
4.5 资料整理、交换以及成果验收 5
5 海洋生物要素调查 6
5.1 海洋生物群落结构要素调查 6
5.2 海洋生态系统功能要素调查 7
6 海洋环境要素调查 7
6.1 海洋水文要素调查 7
6.2 海洋气象要素调查 7
6.3 海洋光学要素调查 7
6.4 海水化学要素调查 8
6.5 海洋底质要素调查 10
7 人类活动要素调查 11
7.1 海水养殖生产要素调查 11
7.2 海洋捕捞生产要素调查 11
7.3 入海污染要素调查 11
7.4 海上油田生产要素调查 11
7.5 其它人类活动要素调查 11
8 海洋生物群落结构分析与评价 11
8.1 单元法分析 11
8.2 多变量分析 14
9 海洋生态系统功能评价 16
9.1 初级生产功能评价 16
9.2 新生产功能评价 16
9.3 细菌生产功能评价 16
10 海洋生态压力评价 16

10.1 富营养化压力评价 16
10.2 污染压力评价 17
10.3 养殖压力评价 18
10.4 捕捞压力评价 18
11 编写海洋生态调查报告 19
11.1 海洋生态调查报告内容 19
11.2 编写要求、完成时间和调查成果验收 20
附录A记录表格式（资料性附录） 21
参考文献 27
表1 海水富营养化评价标准 16
表A.1 调查海区温跃层特征值记录表 22
表A.2 调查海区盐跃层特征值记录表 23
表A.3 调查海区悬浮颗粒物和颗粒有机物测定记录表 24
表A.4 调查海区不同粒级的颗粒有机碳测定记录表 25
表A.5 调查海区不同粒级的颗粒氮测定记录表 26

前　言

GB/T 12763《海洋调查规范》分为九个部分：

——第1部分：总则；

——第2部分：海洋水文观测；

——第3部分：海洋气象观测；

——第4部分：海洋化学要素调查；

——第5部分：海洋声、光要素调查；

——第6部分：海洋生物调查；

——第7部分：海洋调查资料交换；

——第8部分：海洋地质地球物理调查；

——第9部分：海洋生态调查指南。

其中，第9部分：海洋生态调查指南对应于GB/T 12763－1991是新增部分。

本部分与GB/T 12763的第1部分至第7部分、GB/T 17378的第4部分、第5部分和第7部分配套使用。

本部分的附录A为资料性附录。

本部分由国家海洋局提出。

本部分由国家海洋标准计量中心归口。

本部分由国家海洋局第一海洋研究所负责起草，国家海洋局第三海洋研究所参加起草。

本部分主要起草人：陈尚、李瑞香、朱明远、王宗灵、张朝辉、吕瑞华、丁德文、唐森铭。

引 言

海洋生态调查与研究涉及海洋科学的主要领域，1990 年以来发展迅猛。我国现有的《海洋调查规范》和《海洋监测规范》主要针对海洋自然要素的调查与监测，不能满足海洋生态调查的要求。海洋生态调查有别于传统的海洋生物调查，有着自己独特的要求，侧重生态过程和生态系统评价，考虑到高强度人类活动对海洋生态系统的影响，这些都超出了现有的海洋生物调查规范的框架体系。而且，其它国家目前尚未发布类似的海洋生态调查指南，没有现成的指南可以借鉴。因此，需要制定一个指导性的海洋生态调查技术文件，建立海洋生态调查的框架体系，以指导我国海洋生态调查研究，推动我国乃至世界海洋生态科学的快速发展。

编制海洋生态调查指南是我国实施海洋综合管理的需要。海洋综合管理的的本质就是基于海洋生态系统的管理，包括海洋生态系统的调查/监测、生态系统评估、管理计划制定、管理措施落实、管理成效评估等方面。我国海洋生态系统具有独特的生态特征，面临着严峻的生态环境问题，需要加强调查、评估、管理和保护。因此，编制了我国第一部海洋生态调查指南，以满足海洋管理、海洋生态保护和海洋生物资源管护的需要，并且积极推动我国海洋生态系统长期定位监测工作的发展。

我国实施海洋生态调查的主要目的就是了解海洋生态系统基本的群落结构和生态功能及其重要影响因素，并掌握生态系统的健康状况，包括对生态系统现状的了解和对未来变化趋势的把握。海洋生态调查涉及要素和内容很多，鉴于目前的技术水平、方法和经费限制，应选择那些最能反映我国海洋生态系统特征的要素和评价内容。该指南主要针对一般性的海洋生态调查研究工作，提供指导性的框架体系和方法，主要采用简单易行、成熟的方法。另外，考虑到指南要具有前瞻性和引导性，因此也采用了较成熟的先进方法，在使用过程中改进，今后修订时完善。

海洋调查规范
第 9 部分：海洋生态调查指南

1 范围

GB/T 12763 的本部分规定了海洋生态调查的内容、方法、技术要求和资料处理。

本部分适用于中华人民共和国管辖的近海、海湾、河口海洋生态调查，大洋生态调查可参照使用。

2 规范性引用文件

下列文件中的条款通过 GB/T 12763 的本部分的引用而成为本部分的条款。凡是注日期的引用文件，其随后所有的修改单（不包括勘误的内容）或修订版均不适用于本部分。然而，鼓励根据本部分达成协议的各方，研究是否可使用这些文件的最新版本。凡是不注日期的引用文件，其最新版本适用于本部分。

GB/T 12763.1 海洋调查规范 第 1 部分：总则

GB/T 12763.2 海洋调查规范 第 2 部分：海洋水文观测

GB/T 12763.3 海洋调查规范 第 3 部分：海洋气象观测

GB/T 12763.4 海洋调查规范 第 4 部分：海水化学要素调查

GB/T 12763.5 海洋调查规范 第 5 部分：海洋声、光要素调查

GB/T 12763.6 海洋调查规范 第 6 部分：海洋生物调查

GB/T 12763.7 海洋调查规范 第 7 部分：海洋调查资料交换

GB/T 12763.8 海洋调查规范 第 8 部分：海洋地质地球物理调查

GB/T 17378.4 海洋监测规范 第 4 部分：水质检测与分析

GB/T 17378.5 海洋监测规范 第 5 部分：沉积物分析

GB/T 17378.7 海洋监测规范 第 7 部分：近海污染生态调查和生物监测

3 术语和定义

GB/T 15919-1995 和 GB/T 12763 确立的相关术语和定义以及下列术语和定义适用于 GB/T 12763 的本部分。

3.1

海洋生态系统 marine ecosystem

一定海域内生物群落与周围环境相互作用构成的自然系统，具有相对稳定功能并能自我调控的生态单元。

[GB/T 15919-1995，定义 2.94]

3.2

海洋生物群落结构 marine biotic community structure

海洋生物群落的物种组成、空间格局和时间动态等特征。

3.3

海洋生态系统功能 marine ecosystem function

海洋生态系统中的物质循环、能量流动、信息传递及其调控作用。

3.4

海洋生态系统健康 marine ecosystem health

海洋生态系统随着时间的进程有活力并且能维持其组织结构及自主性，在外界胁迫下容易恢复。

3.5

优势种 dominant species

具有控制群落和反映群落特征、数量上所占比例较多的种群。

[GB/T 15919-1995，定义 2.88]

3.6

指示种 indicated species

海洋生物群落在一定海域一定状态出现的标志性的物种。

3.7

关键种 keystone species

食物网中处于关键环节起到控制作用的物种。

3.8

物种多样性 species diversity

生物群落中物种的丰富度及其个体数量分布。

3.9

群落均匀度 community evenness

生物群落中各物种间数量分布的均匀程度。

3.10

群落演变 temporal change of community

生物群落的结构随时间而发生的变化。

3.11

群落空间格局 spatial pattern of community

沿一定的环境梯度（如纬度梯度、水深、温度梯度、盐度梯度、营养盐梯度、底质类型等）海洋生物群落结构发生相应改变而形成的分布型。

3.12

生态压力 ecological stress

来自陆地、海洋、大气的自然干扰和人类活动对海洋生态系统产生的胁迫。

3.13

富营养化 eutrophication

海水中营养盐的自然或人为增加及其引起的生态效应。

3.14

污染压力 pollution stress

入海污染物质对海洋生态系统结构和功能的胁迫。

3.15

养殖压力 aquaculture stress

通过养殖生产输出物质对海洋生态系统物质循环的胁迫。

3.16

捕捞压力 fishing stress

通过捕捞生产输出物质对海洋生态系统物质循环的胁迫。

4 一般规定

4.1 技术设计和调查计划编制

4.1.1 技术设计

接受调查项目后，承担单位应根据任务书或合同书的要求，在调查工作开始前，进行详细的技术设计，内容应包括：

a) 调查目的和任务；

b) 调查海区、采样层次，参照 GB/T 12763.2、GB/T 12763.4、GB/T 12763.6 的规定执行；

c) 站位布设原则：

1）研究对象空间分布变化大的区域，多布站位；空间差异小的区域，少布站位。

2）人类活动强度大的海区多布站位，近岸海区多布站位，内湾多布站位，环境复杂的海区多布设站位等。

3）海湾和近岸调查站位间隔不低于每 10 分 1 个站位，河口和排污口应适当加密设站，远海调查站位间隔不低于每 1 度 1 个站位。具体站位间隔应根据调查的目标和对象确定。

4）沿调查要素变化梯度（如盐度、温度、深度、营养盐、污染物、海流流向、潮区等）布设站位。

5）考虑经费保障和时间。

6）其它特殊要求。

d) 调查时间和频率：

1）调查时间应考虑环境对生物的长期效应，应保证资料的连续性。调查时间和频次应根据具体的调查对象作适当调整，调查频次的时间间隔原则上应小于调查对象的生活（变化）周期。

2）昼夜连续观测推荐每 3h 采样一次，一昼夜共九次。在正规半日潮的海区，应考虑潮周期，采样时间应包括高潮时和低潮时，采用现场自动记录仪可加密观测。

3）大面和断面调查建议 1~3 个月调查一次，各月调查的时间间隔应尽量相等。海湾、河口、港湾调查应在相同的潮期进行，适当增加调查频次。

4）季度调查宜安排在 2 月、5 月、8 月和 11 月，如有特殊需要应根据不同海区调整调查月份。

5）突发事故，如赤潮灾害、溢油、污染物排放等，应增加调查频次。

6）海洋工程及海岸工程的环境影响调查，根据管理需求安排调查频率和时间。

e) 调查要素、方法、技术要求；

f) 测定方法与质量控制；

g) 上船的人员、仪器设备、试剂和表格；

h) 室内的分析人员、仪器设备、试剂和表格；

i) 对调查船及其主要设备的要求；

j) 调查资料质量要求与资料的整理和验收；

k) 应提交的调查成果及完成时间；

l) 其它特殊要求。

4.1.2 调查计划编制

在技术设计的基础上应编制调查计划，按 GB/T 12763.1 的规定执行。

4.2 调查内容

4.2.1 海洋生态要素调查

4.2.1.1 海洋生物要素调查

海洋生物要素调查包括：

a）海洋生物群落结构要素调查；

b）海洋生态系统功能要素调查。

4.2.1.2 海洋环境要素调查

海洋环境要素调查包括：

a) 海洋水文要素调查；

b) 海洋气象要素调查；

c) 海洋光学要素调查；

d) 海水化学要素调查；

e) 海洋底质要素调查。

4.2.1.3 人类活动要素调查

人类活动要素调查包括：

a) 海水养殖生产要素调查；

b) 海洋捕捞生产要素调查；

c) 入海污染要素调查；

d) 海上油田生产要素调查；

e) 其它人类活动要素调查。

4.2.2 海洋生态评价

4.2.2.1 评价对象

评价对象包括：

a）微生物；

b）浮游植物；

c）浮游动物；

d）游泳动物；

e）底栖生物；

f）潮间带生物；

g）污损生物。

4.2.2.2 评价内容

评价内容包括：

a) 海洋生物群落结构评价；

b) 海洋生态系统功能评价；

c) 海洋生态压力评价。

4.3 海上调查作业一般规定

有关海上调查作业的一般规定按 GB/T 12763.1 的规定执行。

4.4 质量控制管理

质量控制包括质量控制体系的建立、调查全过程的质量控制以及对调查分析人员的要求等。有关质量控制管理的详细内容按 GB/T 12763.1 的规定执行。

4.5 资料整理、交换以及成果验收

4.5.1 数据记录、整理、交换和验收

数据的记录、整理、交换和验收按 GB/T 12763.1 和 GB/T 12763.7 的规定执行。

4.5.2 航次报告编写

每个航次结束后，应及时按合同或任务书和 GB/T 12763.1 的规定编写航次报告。

4.5.3 精密度

精密度要求如下：

a）浮游植物、浮游动物和小型底栖生物样品计数的精密度，以标准误差表示；

b）群落结构差异比较需进行显著性统计检验。若取样前已存在某种零假设，则可根据几种试验设计类型做出检验。建议 3 个重复样时，成对比较的显著水平最小不超过 10%，4 个重复样时为 3%，5 个重复样时为 1%，若要在 5%的水平获得显著差异，一般至少需要 4 个重复。

4.5.4 资料归档

资料归档按 GB/T 12763.1 的规定执行。

5. 海洋生物要素调查

5.1 海洋生物群落结构要素调查

5.1.1 微生物、叶绿素a、游泳动物、底栖生物、潮间带生物和污损生物调查

微生物、叶绿素 a、游泳动物、底栖生物、潮间带生物和污损生物调查均按 GB/T 12763.6 的规定执行。

5.1.2 浮游植物调查

5.1.2.1 网采样品和采水样品的采集、处理

网采样品和采水样品的采集与处理按 GB/T 12763.6 的规定执行。

5.1.2.2 采水样品的鉴定计数

采水样品显微鉴定计数时分三个粒级：小于 20μm、20μm～200μm、大于 200μm。粒级按细胞最大长度计算，对于那些多个细胞聚集形成的群体，则按群体的最大长度分级。对于小于 20μm 的浮游植物鉴定到种或属会有一定难度，如没有倒置显微镜和荧光显微镜，细胞的测量和计数都有困难，可根据调查任务的要求酌情处理。

5.1.2.3 绘制分布图

分别绘制总浮游植物和各粒级浮游植物细胞密度的分布图和粒级结构图，各粒级浮游植物细胞密度的等值线取值标准参照 GB/T 12763.6 执行，也可视具体情况酌情增减。

5.1.3 浮游动物调查

5.1.3.1 网采浮游动物

网采浮游动物调查按 GB/T 12763.6 的规定执行。

5.1.3.2 水采浮游动物

5.1.3.2.1 技术要求

采样技术要求如下：

a）采样层次：按 GB/T 12763.6 的规定执行；

b）分级：20μm～200μm、200μm～500μm、大于 500μm；

c）采水量：30dm^3～70dm^3，依不同海区情况而定；

d）连续观测采样频次：每 3h 采样一次，一昼夜共九次。

5.1.3.2.2 样品处理

样品处理步骤如下：

a）过滤：取 20dm^3～60dm^3 水样，依次经 500μm、200μm、20μm 筛绢过滤，分别冲洗到小瓶中，各规格筛绢也可自行设计成直径大小不同的的小网，网口直径一般为 15cm、20cm、25cm 均可，网衣长度分别为 15cm、20cm、30cm，滤过样品的固定同 GB/T 12763.6 规定的网采样品；

b）样品编号：按 GB/T 12763.6 浮游动物的规定，但编号末尾应加 020、200 和 500，分别表示经 20μm、200μm、500μm 筛绢过滤。

5.1.3.2.3 鉴定计数

水采浮游动物的鉴定计数按 GB/T 12763.6 浮游动物的规定执行。

5.1.3.2.4 数据处理

水采浮游动物的数据处理按GB/T 12763.6中浮游动物的数据处理方法执行。但应计算各粒级浮游动物的种类、个体数量和生物量（粒级小的浮游动物如原生动物，可酌情考虑不称量），并绘制浮游动物总数和各粒级的分布图和粒级结构图，各粒级的个体数量和生物量的等值线取值标准参照GB/T 12763.6浮游动物的规定执行，也可视具体情况酌情增减。

5.2 海洋生态系统功能要素调查

海洋生态系统功能要素目前着重调查初级生产力、新生产力和细菌生产力，具体调查内容按GB/T 12763.6的规定执行。

6 海洋环境要素调查

6.1 海洋水文要素调查

6.1.2 深度、水温、盐度、水位和海流

深度、水温、盐度、水位和海流调查按GB/T 12763.2的规定执行。

6.1.3 温跃层和盐跃层

调查方法同水温和盐度。特别处理如下：提取CTD仪器输出的每米水层的水温和盐度，记录格式参见表A.1和A.2，绘制水温、盐度的垂直分布图，确定温跃层和盐跃层的上界深度、下界深度、厚度和强度。

温跃层和盐跃层判断标准按GB/T 12763.7的规定执行。

6.1.4 海面状况

记录调查期间每日和采样时刻的海洋状况，包括：海水混浊状况、波浪大小、漂浮物种类等。

6.1.5 入海河流径流量和输沙量

对调查海区影响比较大的河流，应收集海上调查期间的入海径流量和输沙量。

6.2 海洋气象要素调查

6.2.1 日照时数

从调查海区附近的气象台站收集调查期间逐日（月）的日照时数。

6.2.2 气温、风速和风向

气温、风速、风向的调查按GB/T 12763.3的规定执行。

6.2.3 天气状况

记录调查期间每日和采样时刻的天气状况，如阴、晴、雨、雾等。

6.3 海洋光学要素调查

6.3.1 海面照度、水下向下辐照度和真光层深度

海面照度、水下向下辐照度调查按GB/T 12763.5的规定执行。

真光层深度计算如下：提取表层和每米水层的向下辐照度数据，作垂直分布图，确定向下辐照度为表层的100%、50%、30%、10%、5%和1%的深度。

真光层判断标准：取向下辐照度为表层1%的深度作为真光层的下界深度；若真光层大于水深，取水深作为真光层的深度。

6.3.2 透明度

透明度调查按GB/T 17378.4的规定执行。

6.4 海水化学要素调查

6.4.1 总氮、硝酸盐、亚硝酸盐、铵盐、总磷、活性磷酸盐、活性硅酸盐、溶解氧和pH

总氮、硝酸盐、亚硝酸盐、铵盐、总磷、活性磷酸盐、活性硅酸盐、溶解氧和pH调查按GB/T 12763.4的规定执行。

6.4.2 化学耗氧量

化学耗氧量调查按GB/T 17378.4的规定执行。

6.4.3 重金属、有机污染物和油类

重金属（总汞、铜、铅、镉、总铬、砷）、有机污染物（硫化物、氰化物、有机氯农药、挥发酚类）和油类调查按GB/T12763.4和 GB/T17378.4的规定执行。所测定的要素可根据调查任务和海区的具体情况酌情增减。

6.4.4 悬浮颗粒物（SPM）和颗粒有机物（POM）

6.4.4.1 技术要求

6.4.4.1.1 采样层次

采样层次按GB/T 12763.4规定的标准层次采样。

6.4.4.1.2 采水量

近海水采水量一般 1000cm^3～2000 cm^3，远海水一般 2000 cm^3～3000cm^3，可视具体情况酌情增减。

6.4.4.1.3 精密度

重复样品的相对误差为±10%。

6.4.4.2 样品处理与测定

6.4.4.2.1 滤膜的预处理

滤膜的预处理如下：

a）将∅47mmGF/F 玻璃纤维滤膜先在 450°C 下灼烧 5h，待冷却至室温后，置于干燥器保存；

b）称重：将灼烧过的玻璃纤维滤膜编号，用 0.01mg 的电子天平称其膜重。

如果只测定 SPM，可用 0.45μm 醋酸纤维滤膜代替，详细方法按 GB/T 12763.4 的规定执行。

6.4.4.2.2 过滤

过滤步骤如下：

a）过滤设备：包括滤器、抽滤瓶和真空泵；

b）过滤: 取水样，近海水 1000cm^3～2000cm^3、远海水 2000cm^3～3000cm^3，经 GF/F 玻璃纤维滤膜真空过滤（过滤压力不超过 50kPa）。

6.4.4.2.3 样品处理、称重与计算

样品的处理、称重与计算如下：

a）滤过的玻璃纤维滤膜按顺序号平放在搪瓷盘中，在 60℃干燥箱中烘干 24h，待恢复至室温后称重，将称得的重量减去膜重，得出悬浮颗粒物的重量；

b）将称过的膜一一对应放入小玻璃培养皿中，记好顺序编号。如果一个培养皿中放几张膜，膜与膜之间必须用无尘的铝箔纸隔开。然后在 450℃马福炉中灼烧 5h，待恢复至室温后取出，放入干燥器中。称重时逐一从干燥器中拿出称重。避免在空气中暴露时间过长增大测量误差。称得的重量为颗粒无机物的重量，再被悬浮颗粒物的重量减，得数为颗粒有机物的重量。

6.4.4.3 数据处理

数据记录参见表 A.3，计算 SPM 和 POM 含量，单位为 mg·dm^{-3}，绘制平面分布图和垂直分布图。

6.4.4.4 等值线取值标准

SPM 取值标准为 1.0，2.5，5.0，10.0，15.0，20.0，25.0，50.0。POM 取值标准为 0.1，0.5，1.0，2.0，3.0，4.0，5.0，10.0，15.0，20.0。

以上取值标准，可视具体情况酌情增减。

6.4.5 颗粒有机碳（POC）和颗粒氮（PN）

6.4.5.1 技术要求

6.4.5.1.1 采样层次

颗粒有机碳（POC）和颗粒氮（PN）的采样层次按 GB/T 12763.4 规定的标准层次采样。

6.4.5.1.2 采水量

近海水 1000cm^3～2000 cm^3，远海水 2000 cm^3～5000cm^3，可视具体情况酌情增减。

6.4.5.1.3 检测下限

颗粒有机碳 20μg·dm^{-3}，颗粒氮 5μg·dm^{-3}。

6.4.5.1.4 分级

分四级：小于 2μm、2μm～20μm、20μm～200μm、大于 200μm。

6.4.5.1.5 精密度

重复样品的相对误差为 ±10%。

6.4.5.2 样品处理与测定

样品处理与测定按以下步骤进行：

a) 滤膜和网具：∅25mm 玻璃纤维滤膜（预先在 450°C 下灼烧 5h 待用）、2μm 核孔滤膜、20μm 筛绢和 200μm 筛绢；

b) 过滤设备：包括滤器、支架、抽滤瓶和真空泵；

c) 过滤：取四份完全相同水样，每份水样体积：近海水 200 cm^3～500 cm^3、远海水 1000 cm^3～3000cm^3，分别标记为 A、B、C、D。水样 A 直接经玻璃纤维滤膜过滤；水样 B 依次经 200μm

筛绢、玻璃纤维滤膜过滤；水样 C 依次经 20μm 筛绢、玻璃纤维滤膜过滤；水样 D 依次经 2μm 核孔滤膜过滤、玻璃纤维滤膜过滤。过滤时抽气负压应小于 50kPa；

d) 样品预处理: 滤过的玻璃纤维滤膜和未过滤的滤膜（作为对照）放入培养皿中，置于密闭容器中，用浓盐酸蒸气熏蒸 30min。熏蒸过的样品置于－18℃冷冻暂存，并尽快置于干燥箱中在 60℃温度下干燥 24h，取出置于干燥器保存；

e) 样品测定: 采用元素分析仪测定，氧化温度 750℃～760℃。

6.4.5.3 数据处理

数据处理如下：

a）颗粒有机碳：

A、B、C、D 四份样品的颗粒有机碳分别表示为：POC_A、POC_B、POC_C、POC_D. 则：

总的有机碳：$POC_{GF/F} = POC_A$；

小于2μm粒级的有机碳：$POC_{GF/F-2} = POC_D$；

2μm～20μm粒级的有机碳：$POC_{2-20} = POC_C - POC_D$；

20μm～200μm粒级的有机碳：$POC_{20-200} = POC_B - POC_C$；

大于200μm粒级的有机碳：$POC_{200} = POC_A - POC_B$。

数据记录参见表A.4，计算各粒级的POC含量，单位为$\mu g \cdot dm^{-3}$，绘制平面分布图和粒级结构图。

b）颗粒氮：

A、B、C、D 四份样品的颗粒氮分别表示为：PN_A、PN_B、PN_C、PN_D. 则：

总的颗粒氮：$PN_{GF/F} = PN_A$；

小于 2μm 粒级的颗粒氮：$PN_{GF/F-2} = PN_D$；

2μm～20μm 粒级的颗粒氮：$PN_{2-20} = PN_C - PN_D$；

20μm～200μm 粒级的颗粒氮：$PN_{20-200} = PN_B - PN_C$；

大于 200μm 粒级的颗粒氮：$PN_{200} = PN_A - PN_B$。

数据记录参见表A.5，计算各粒级的PN含量，单位为$\mu g \cdot dm^{-3}$，绘制平面分布图和粒级结构图。

6.4.5.4 等值线取值标准

各粒级颗粒有机碳取值范围为25、50、75、100、250、500、1000、大于1000；颗粒氮取值范围为5、10、25、50、75、100、250、500、大于500。

以上取值标准，可视具体情况酌情增减。

6.5 海洋底质要素调查

6.5.1 底质类型、粒度、有机碳、总氮、总磷、pH 和 Eh

底质类型、粒度、有机碳、总氮、总磷、pH 和 Eh 的调查均按 GB/T 12763.8 的规定执行。

6.5.2 底质污染物： 硫化物、有机氯、油类、重金属（总汞、铜、铅、镉、总铬、砷、硒）

硫化物、有机氯、油类、重金属（总汞、铜、铅、镉、总铬、砷、硒）的调查按 GB/T 17378.5 的规定执行。

7 人类活动要素调查

7.1 海水养殖生产要素调查

调查海区如果存在一定规模的养殖活动，应调查养殖海区坐标、面积，养殖的种类、密度、数量、方式；收集养殖海区多年的养殖数据，包括养殖时间、种类、密度、数量、单位产量、总产量、养殖从业人口等，并制作养殖空间分布图。具体养殖数据根据不同海区的养殖情况相应增减。

7.2 海洋捕捞生产要素调查

存在捕捞生产活动的海区，应现场调查和调访捕捞作业情况，进行渔获物拍照和统计，并收集该海区多年的捕捞生产数据，包括捕捞生产海区坐标、面积，捕捞的种类、方式、时间、产量，渔船数量（马力），网具规格，捕捞从业人口等，并制作捕捞生产空间分布图。具体捕捞生产数据根据不同海区的情况相应增减。

7.3 入海污染要素调查

存在排海污染（陆源、海上排污等）的调查海区，应调查和收集多年的排污数据，包括排污口、污染源分布，主要污染物种类、成分、浓度、入海数量、排污方式等，并制作排污口和污染源的空间分布图。具体情况根据不同海区的污染源的情况相应增减。

7.4 海上油田生产要素调查

存在油田生产的调查海区，应收集多年的油田生产和污染数据，包括石油平台位置、坐标、数量、产量、输油方式、污水排放量、油水比、溢油事故发生时间、溢油量、污染面积、持续时间，受污染生物种类和数量，使用消油剂种类和使用量等，并制作石油污染源分布图。具体情况根据不同海区的污染源的情况相应增减。

7.5 其它人类活动要素调查

若调查海区存在建港、填海、挖沙、疏浚、倾废、围垦、运动（游泳、帆船、滑水等）、旅游、航运、管线铺设等情况，而且对主要调查对象可能有较大影响时，应调查这些人类活动的情况，调查要素主要包括位置、数量、规模、建设和营运情况，对周围海域自然环境的影响程度，排放污染物的种类、数量、时间等，对海洋生物的影响程度等方面。具体内容根据调查目标确定。

8 海洋生物群落结构分析与评价

8.1 单元法分析

8.1.1 生物量评价

8.1.1.1 评价对象

评价对象包括微生物、浮游植物群落、浮游动物群落、游泳动物群落、底栖生物群落、潮间带生物群落和污损生物群落。

8.1.1.2 评价方法和结果表达

分析各类群的个体数量（微生物指菌落数量，浮游植物指细胞数量，底栖生物、潮间带生物和污损生物指栖息密度）和生物量，绘制空间分布图，评价其变化趋势。

8.1.2 优势种评价

8.1.2.1 评价对象

评价对象包括浮游植物群落、浮游动物群落、游泳动物群落、底栖生物群落、潮间带生物群落和污损生物群落。

8.1.2.2 评价方法

采用优势度评价。某一个站位的优势度，用百分比表示。优势度的计算公式如下：

$$D_i = \frac{n_i}{N} \cdot 100\% \qquad \cdots\cdots(1)$$

式中：

D_i — 第i种的百分比优势度；

n_i —该站位第i 种的数量；

N —该站位群落中所有种的数量，单位可用个体数、密度、重量等表示。

8.1.2.3 结果表达

分析群落优势种丰度及其优势度，绘制空间分布图，评价其变化趋势。

8.1.3 指示种评价

8.1.3.1 评价对象

评价对象包括浮游植物群落、浮游动物群落、游泳动物群落、底栖生物群落、潮间带生物群落和污损生物群落。

8.1.3.2 评价方法和结果表达

分析不同环境压力（如有机污染、重金属污染、油污染等）下生物群落出现的指示性物种，计算其生物量，绘制空间分布图，评价环境和群落的变化趋势。

8.1.4 关键种评价

8.1.4.1 评价对象

评价对象为海洋食物网，包括浮游食物网、高营养阶层食物网、底栖碎屑食物网等。

8.1.4.2 评价方法和结果表达

分析食物网各营养阶层的关键物种，计算其生物量，绘制空间分布图，评价其变化趋势。

8.1.5 物种多样性评价

8.1.5.1 评价对象

评价对象包括浮游植物群落、浮游动物群落、底栖生物群落、潮间带生物群落。

8.1.5.2 评价方法

采用物种多样性指数评价。物种多样性指数一般采用 Shannon 信息指数计算，计算公式如下：

$$H' = -\sum_{i=1}^{s} P_i \log_2 P_i \qquad \cdots\cdots（2）$$

式中：

H' ——种类多样性指数；

P_i ——群落第i种的数量或重量占样品总数量之比值；

S ——群落中的物种数。

数量可以采用个体数、密度表示；重量可用湿重或干重表示。

8.1.5.3 结果表达

计算生物群落的物种多样性，制作空间分布图，评价其变化趋势。多样性指数的等值线取值标准为 0.5，1.0，1.5，2.0，2.5，3.0，3.5，4.0，4.5，5.0，6.0，7.0，8.0。

以上取值标准，可视具体情况酌情增减。

8.1.6 群落均匀度评价

8.1.6.1 评价对象

评价对象包括浮游植物群落、浮游动物群落、底栖生物群落、潮间带生物群落。

8.1.6.2 评价方法

采用均匀度指数评价。采用 Pielou 均匀度指数，计算公式如下：

$$J' = \frac{H'}{\log_2 S} \qquad \cdots\cdots（3）$$

式中：

J'——均匀度指数；

H' ——群落实测的物种多样性指数；

S——群落中的物种数。

8.1.6.3 结果表达

计算不同生物群落的均匀度，制作空间分布图，评价其变化趋势。均匀度指数等值线取值标准为0.2，0.4，0.6，0.8，1.0。

以上取值标准，可视具体情况酌情增减。

8.1.7 群落演变评价

8.1.7.1 评价对象

评价对象浮游植物群落、浮游动物群落、底栖生物群落、潮间带生物群落。

8.1.7.2 评价方法

群落演变评价采用演变速率指标,群落演变速率指标采用β多样性指数评价。β多样性指数测度群落间的相似性大小。

演变速率（E）的计算方法如下：

$$E=1-\frac{S_{IMi}}{S_{IMO}} \quad \cdots\cdots(4)$$

式中：

S_{IMO}、S_{IMi}—初始群落和时间尺度上第i群落的相似性指数。

相似性指数计算公式如下：

$$S_{IM}=2\frac{N_{coi}}{S_o+S_i} \quad \cdots\cdots(5)$$

式中：

$N_{coi}=\sum_{j=1}^{n}\min(N_{coo}, N_{coi})$，表示初始群落和时间尺度上第i时刻群落共有种的个体数较小者之和，

n —第i时刻和初始群落共有的物种数；

S_0、S_i —初始群落和第i群落的物种数。

演变速率（E）介于0~1之间。E＝0，两个群落结构完全相同，没有发生演变；E＝1，两个群落结构完全不同，没有共同种，发生完全演变。通常情况下,$0<E<1$，两个群落的结构发生部分改变。

8.1.7.3 结果表达

计算不同生物群落的演变速率，沿时间系列绘制演变图，评价其演变趋势。

8.2 多变量分析

8.2.1 评价对象

评价对象主要适应于无运动能力或运动能力较弱的浮游植物、浮游动物和区域性较强的底栖生物和潮间带生物群落。

8.2.2 分析方法

等级聚类（Cluster）、非度量多维标度（MDS）、主分量分析（PCA）等多变量分析是包括一系列以等级相似性为基础的非参数技术方法，用于分析生物群落的空间格局和确定其主要支配因素。

8.2.2.1 等级聚类（Cluster）

等级聚类的目的是确定生物群落样品的自然分组，使得组内样品彼此间较组间样品更为相似，分析结果以树枝图的形式表示,该图给出了样品间彼此的相似性水平。

8.2.2.2 非度量多维标度（MDS）

非度量多维标度就是在一个低维标序空间中建立一个样品的“地图”或构型图，使样品间欧氏距离的等级顺序与其相似性或非相似性的等级顺序保持一致，比较准确地反映复杂的生物群落样品之间的关系。非度量多维标度与等级聚类结合使用可以有效地揭示群落变化的连续梯度。

8.2.2.3 主分量分析（PCA）

主分量分析的功能是把多维空间中的点向低维空间作有效投影以使点的排列遭受最小可能的畸变，得到较少的主要分量，并尽可能多地反映原来变量的信息，并找出生物群落变化的主要支配因素。

8.2.3 分析步骤

分析包括如下步骤：

a）原始生物资料矩阵和环境资料矩阵的建立；

b）样品间（非）相似性测定和（非）相似性矩阵的建立；第 j 与第 k 个样品间的 Bray-Curtis 相似性 S_{jk} 由下式计算：

$$S_{jk} = 100 \times \left\{ 1 - \frac{\sum_{i=1}^{p} \left| y_{ij} - y_{ik} \right|}{\sum_{i=1}^{p} \left(y_{ij} + y_{ik} \right)} \right\} \quad \cdots\cdots（6）$$

式中：

y_{ij}——原始矩阵第i行和第j列的输入值。表示第j个样品中第i种的丰度（或生物量）（i=1,2,…P; j=1,2,……n）,y_{ik}由此类推。

c）计算原始环境矩阵中每对样品间环境组成非相似性，产生一个三角形非相似性矩阵。第 j 与第 k 个样品间的欧氏距离非相似性（d_{jk}）计算公式为：

$$d_{jk} = \sqrt{\sum_{i=1}^{p} \left(y_{ij} - y_{ik} \right)^2} \quad \cdots\cdots（7）$$

式中符号同公式（6）。

d）通过样品的聚类和标序表达群落结构格局。

e）统计检验。

8.2.4 群落结构差异的统计检验

等级聚类、非度量多维标度和主分量分析的结果应进行群落结构差异显著性检验。可采用以下几种检验：

a）方差分析（ANOVA）；

b）样品相似性矩阵的 ANOSIM 检验；

c）BIOENV/BVSTEP 分析；

d）RELATE 检验。

多变量分析的内容视研究目的和调查任务承担单位的技术条件确定是否进行或是部分进行。

8.2.5 数据处理

上述多变量分析的数据处理可以自行编写程序，也可采用现成软件。

8.2.6 结果表达

绘制多变量分析有关图表：如等级聚类图、MDS 图、主分量贡献图、ABC 曲线、K－优势度曲线等。

9 海洋生态系统功能评价

9.1 初级生产功能评价

海洋生态系统中初级生产功能主要由浮游植物承担，初级生产提供了生态系统运转的大部分的能量来源。初级生产功能采用初级生产力评价，单位：$mg \cdot m^{-3} \cdot d^{-1}$ 或 $g \cdot m^{-2} \cdot d^{-1}$（均以碳计）。

绘制初级生产功能的空间分布图，评价其变化趋势。

9.2 新生产功能评价

新生产指由浮游植物利用新进入真光层的营养盐完成的有机物生产。新生产功能采用新生产力评价，单位：$mg \cdot m^{-3} \cdot d^{-1}$ 或 $g \cdot m^{-2} \cdot d^{-1}$（均以碳计）。

绘制新生产功能的空间分布图，评价其变化趋势。

9.3 细菌生产功能评价

海洋生态系统中细菌生产功能主要由异样细菌承担，细菌生产提供了生态系统运转的补充能量来源。细菌生产功能采用细菌生产力评价，单位：$mg \cdot m^{-3} \cdot d^{-1}$ 或 $g \cdot m^{-2} \cdot d^{-1}$（均以碳计）。

绘制细菌生产功能的空间分布图，评价其变化趋势。

10 海洋生态压力评价

10.1 富营养化压力评价

富营养化压力评价采用海水营养指数。营养指数的计算主要有两种方法。

第一种方法考虑化学耗氧量、总氮、总磷和叶绿素 a。计算公式如下：

$$N_I = C_{COD}/S_{COD} + C_{TN}/S_{TN} + C_{TP}/S_{TP} + C_{Chla}/S_{Chla} \quad \cdots\cdots (8)$$

式中：

N_I —营养指数；

C_{COD}、C_{TN}、C_{TP}、C_{Chla} —分别为水体中化学耗氧量、总氮、总磷、叶绿素 a 的实测浓度；

S_{COD}、S_{TN}、S_{TP}、S_{Chla} —分别为水体中化学耗氧量、总氮、总磷、叶绿素 a 的评价标准，见表 1。

当营养指数大于 4 时，认为海水达到富营养化。

表 1 海水富营养化评价标准

S_{COD}	S_{TN}	S_{TP}	S_{Chla}
$3.0 mg \cdot dm^{-3}$	$0.6 mg \cdot dm^{-3}$	$0.03 mg \cdot dm^{-3}$	$10 \mu g \cdot dm^{-3}$

此方法为仲裁方法。

第二种方法考虑化学耗氧量、溶解无机氮、溶解无机磷。计算公式如下：

$$N_I = (C_{COD} \times C_{DIN} \times C_{DIP})/4500 \quad \cdots\cdots (9)$$

式中：

N_I —营养指数；

C_{COD}、C_{DIN}、C_{DIP} —分别为水体中化学耗氧量（mg·dm^{-3}）、溶解无机氮（μg·dm^{-3}）、溶解无机磷（μg·dm^{-3}）的实测浓度。

4500－为 COD、DIN 和 DIP 的三类海水水质标准值的乘积。

当营养指数 N_I >1，认为水体富营养化。

10.2 污染压力评价

10.2.1 氮污染压力评价

采用氮污染压力指数评价。

某月（年）的氮污染压力指数等于该月（年）的入海氮通量除以该月（年）水体中总氮平均含量。这里，入海氮通量指进入调查海区的氮的总量，包括无机态氮和有机态氮。计算公式如下：

$$P_N = F_{LUXN} / C_N \quad \cdots\cdots (10)$$

式中：

P_N —氮污染压力指数，单位：m^3/月（年）；

F_{LUXN} —入海氮通量，单位：kg/月（年）；

C_N —水体中总氮含量，单位：kg·m^{-3}。

根据以上计算，确定高污染压力海区，分析氮污染压力的变化趋势。

10.2.2 磷污染压力评价

采用磷污染压力指数评价。

某月（年）的磷污染压力指数等于该月（年）的入海磷通量除以该月（年）水体中总磷平均含量。这里，入海磷通量指进入调查海区的磷的总量，包括无机磷和有机磷。计算公式如下：

$$P_P = F_{LUXP} / C_P \quad \cdots\cdots (11)$$

式中：

P_P —磷污染压力指数，单位： m^3/月（年）；

F_{LUXP} —入海磷通量，单位： kg/月（年）；

C_P —水体中总磷含量，单位：kg·m^{-3}。

根据以上计算，确定高污染压力海区，分析污染压力的变化趋势。

10.2.3 油污染压力评价

采用油污染压力指数评价法。

某月（年）的油污染压力指数等于该月（年）的入海油通量除以该月（年）水体中油的平均含量。计算公式如下：

$$P_O = F_{LUXO} / C_O \quad \cdots\cdots (12)$$

式中：

P_O —油污染压力指数，单位：m^3/月（年）；

F_{LUXO} —入海油通量，单位：kg/月（年）；

C_O —水体中油含量，单位：kg·m^{-3}。

根据以上计算，确定高污染压力海区，分析污染压力的变化趋势。

10.2.4 有机污染压力评价

采用COD污染压力指数评价。

某月（年）的COD污染压力指数等于该月（年）的入海COD通量除以该月（年）水体中COD的平均含量。计算公式如下：

$$P_{COD}=F_{LUXCOD}/C_{COD} \quad \cdots\cdots(13)$$

式中：

P_{COD} —COD污染压力指数，单位：m^3/月（年）；

F_{LUXCOD} —入海COD通量，单位：kg/月（年）；

C_{COD} —水体中COD含量，单位：$kg \cdot m^{-3}$。

根据以上计算，确定COD高污染压力海区，分析污染压力的变化趋势。

10.3 养殖压力评价

采用养殖压力指数法评价。

对于滤食性贝类和浮游生物食性鱼类，其养殖压力指数等于单位时间内养殖收获净输出的有机碳（氮）通量除以该调查区同时期水体中颗粒有机碳（氮）的平均含量。单位时间为月或年。计算公式如下：

$$P_{PA}=P_A/C_{POC-PON} \quad \cdots\cdots(14)$$

式中：

P_{PA} —养殖压力指数，单位：m^3/月（年）；

P_A —养殖收获净输出的有机碳或有机氮通量，单位： kg/月（年）；

$C_{POC-PON}$ —水体中颗粒有机碳或有机氮含量，单位：$kg \cdot m^{-3}$。

这里，养殖收获净输出的碳（氮）通量等于养殖收获物的有机碳（氮）通量减去苗种和饵料的有机碳（氮）通量。

$$P_A=P_{RODA}\times f_A-Q_A\times f_A-F_{OODA}\times f_{FOODA} \quad \cdots\cdots(15)$$

式中：

P_{RODA} —养殖产量，单位： kg/月（年）；

Q_A —养殖苗种投放量，单位： kg/月（年）；

F_{OODA} —饵料投喂量，单位： kg/月（年）；

f_A —养殖生物的有机碳（氮）含量系数；

f_{FOODA} —投喂饵料的有机碳（氮）含量系数。

根据以上计算，确定高养殖压力的海区，分析养殖压力的变化趋势。

10.4 捕捞压力评价

捕捞压力分为两类。在高营养阶层，捕捞直接减少渔业生物的现存量，称为Ⅰ类捕捞压力。在低营养阶层，捕捞加速浮游生态系统中颗粒有机物质的输出，称为Ⅱ类捕捞压力。捕捞压力评价应分别进行。

10.4.1 Ⅰ类捕捞压力评价

采用Ⅰ类捕捞压力指数法评价。某月（年）的捕捞压力指数等于该月（年）渔获量除以该月（年）的渔业资源现存量。计算公式如下：

$$P_{PF}=P_F / S_F \quad \cdots\cdots(16)$$

$$P_F=P_{RODF}\times f_F \quad \cdots\cdots(17)$$

式中：

P_{PF} —捕捞压力指数，单位：km^2/月（年）；

P_F —通过渔获物输出的有机碳（氮）通量，单位：kg/月（年）；

S_F —调查海区的渔业资源现存量（以有机碳或有机氮计），单位：$kg\cdot km^{-2}$；

P_{RODF} —渔获量，以湿重计算，单位：kg/月（年）；

f_F —渔获物中有机碳（氮）含量系数。

若研究的渔获量和资源量指同一种类，或者虽然是不同种类但具有相同的有机碳（氮）含量，它们的单位可以用质量表示，不必转换为碳（氮）计算。

渔业资源现存量计算按 GB/T 12763.6 的规定执行。

根据以上计算，确定高捕捞压力的海区，分析捕捞压力的变化趋势。

10.4.2 Ⅱ类捕捞压力评价

采用Ⅱ类捕捞压力指数法评价。某月（年）的捕捞压力指数等于该月（年）渔获物的有机碳（氮）通量除以该月（年）海水中颗粒有机碳（氮）平均含量。计算公式如下：

$$P_{PF}=P_F / C_{POC-PON} \quad \cdots\cdots(18)$$

$$P_F=P_{RODF}\times f_F \quad \cdots\cdots(19)$$

式中：

P_{PF} —捕捞压力指数，单位：m^3/月（年）；

P_F —通过渔获物输出的有机碳（氮）通量，单位：kg/月（年）；

$C_{POC-PON}$ —水体中颗粒有机碳（氮）含量，单位：$kg\cdot m^{-3}$；

P_{RODF} —渔获量，单位：kg/月（年）；

f_F —渔获物中有机碳（氮）含量系数。

根据以上计算，确定高捕捞压力的海区，分析捕捞压力的变化趋势。

11 编写海洋生态调查报告

11.1 海洋生态调查报告内容

编写内容包括：

a）前言；

b）调查海区的自然环境和社会经济特征；

c）野外调查工作状况；

d）样品采集分析和数据处理方法；

e）质量计划实施情况报告；

f）调查资料汇编；

g）图集；

h）海洋生态调查分析；

海洋生态调查分析主要内容包括：

1）调查海区生物特征；

2）调查海区环境特征；

3）调查海区人类活动特征；

4）调查海区海洋生物群落结构评价；

5）调查海区海洋生态系统功能评价；

6）调查海区生态压力评价；

7）调查海区海洋生态系统总体评价；

8）改善生态环境健康的对策建议。

a）～ g）项内容按 GB/T 12763.1 的规定编写。

11.2 编写要求、完成时间和调查成果验收

编写要求、完成时间和调查成果验收按 GB/T 12763.1 的规定执行。

附录 A
（资料性附录）
记录表格式

A.1 调查海区温跃层特征值记录表，见表 A.1。

A.2 调查海区盐跃层特征值记录表，见表 A.2。

A.3 调查海区悬浮颗粒物（SPM）和颗粒有机物（POM）测定记录表，见表 A.3。

A.4 调查海区不同粒级的颗粒有机碳（POC）记录表，见表 A.4。

A.5 调查海区不同粒级的颗粒氮（PN）记录表，见表 A.5。

表 A.1 调查海区温跃层特征值记录表

海区： 调查船： 测定日期 年 月 日

站位	经度	纬度	水深 m	温跃层上界深度 m	温跃层下界深度 m	温跃层厚度 m	温跃层强度 ℃·m^{-1}

测定者 计算者 校对者

表 A.2 调查海区盐跃层特征值记录表

海区：　　　　　　调查船：　　　　　　　　　测定日期：　　　　　年　月　日

站位	经度	纬度	水深<br>m	盐跃层上界深度 m	盐跃层下界深度 m	盐跃层厚度<br>m	盐跃层强度<br>m^{-1}

测定者　　　　　　计算者　　　　　　校对者

表 A.3 调查海区悬浮颗粒物和颗粒有机物测定记录表

海区__________ 调查船 _______ 站位________ 经度_____________ 纬度_____________ 航次_________

水深 _________ 采样日期 ______年____月____日 测定日期 ______年____月____日 第_____页 共_____页

序号	滤膜编号	水层<br>m	过滤水体<br>dm^{-3}	滤膜重量<br>mg	60℃烘干后<br>重量 mg	450℃灼烧后<br>重量 mg	SPM<br>$mg\cdot dm^{-3}$	POM<br>$mg\cdot dm^{-3}$	备注

测定者 计算者 校对者

表 A.4 调查海区不同粒级的颗粒有机碳测定记录表

海区__________ 调查船 _______ 站位________ 经度_____________ 纬度_____________ 航次_________

水深 _________ 采样日期 ______年____月____日 测定日期 _______年____月____日 第_____页 共_____页

序号	滤膜编号	水层<br>m	过滤水体<br>dm^{-3}	POC_A<br>$\mu g \cdot dm^{-3}$	POC_B<br>$\mu g \cdot dm^{-3}$	POC_C<br>$\mu g \cdot dm^{-3}$	POC_D<br>$\mu g \cdot dm^{-3}$	$POC_{GF/F}$<br>$\mu g \cdot dm^{-3}$	$POC_{GF/F-2}$<br>$\mu g \cdot dm^{-3}$	POC_{2-20}<br>$\mu g \cdot dm^{-3}$	POC_{200}<br>$\mu g \cdot dm^{-3}$

测定者 计算者 校对者

25

表 A.5 调查海区不同粒级的颗粒氮测定记录表

海区__________ 调查船 ________ 站位________ 经度_____________ 纬度_____________ 航次_________

水深 _________ 采样日期 ______年____月____日 测定日期 ______年____月____日 第_____页 共_____页

序号	滤膜编号	水层<br>m	过滤水体<br>dm^{-3}	PN_A<br>$\mu g \cdot dm^{-3}$	PN_B<br>$\mu g \cdot dm^{-3}$	PN_C<br>$\mu g \cdot dm^{-3}$	PN_D<br>$\mu g \cdot dm^{-3}$	$PN_{GF/F}$<br>$\mu g \cdot dm^{-3}$	$PN_{GF/F-2}$<br>$\mu g \cdot dm^{-3}$	PN_{2-20}<br>$\mu g \cdot dm^{-3}$	PN_{200}<br>$\mu g \cdot dm^{-3}$

测定者　　　　计算者　　　　校对者

参考文献

[1] 国家海洋局.海洋生态环境监测技术规程.2002.

[2] 马克平.生物群落多样性的测度方法.见钱迎倩,马克平主编.生物多样性研究的原理和方法.北京：科学出版社.1994.

[3] 任海等编.恢复生态学导论.北京:科学出版社.2002.

[4] 沈国英等编.海洋生态学.北京:科学出版社.2002.

[5] 大森倍.池田勉著,罗会明等译，黄振祥校.浮游动物生态的研究.北京:科学出版社.1987.

[6] J.S.格雷著,阎铁等译,张志南校.海洋沉积物生态学－底栖生物群落结构与功能导论.北京:海洋出版社1987.

[7] 杨鹤鸣等.胶州湾海水颗粒有机碳和颗粒氮测定方法的改进.见董金海等主编.胶州湾生态学研究.北京:科学出版社. 1994.

[8] 邹娥梅等.黄东海温跃层的分布特征及其季节变化.黄渤海海洋.2001,19(3):8-18.

[9] 邹景忠等.渤海湾富营养化和赤潮问题的初步探讨.海洋环境科学.1983,2(2):42-55.

[10] 任海, 邬建国, 彭少麟. 生态系统健康的评估.热带地理.2000, 20(4):310-316.

[11]郭玉洁等. GB/T 15919-1995 海洋学术语 海洋生物学.中国标准出版社.1996.

[12] UNESCO-IOC ed. Training course report on environmental effects on benthic communities. No.19. 1992.

[13] Harris P ed. Phytoplankton ecology: structure, function and fluctuation. Chapman & Hall. 1986.

[14] Jumars P A ed. Concepts in biological oceanography. Oxford University Press. 1993.

[15] Lalli C M and Parsons T R eds. Biological oceanography: an introduction. Pergamon Press. 1993.

[16] Rapport D J ed. Ecosystem health. Oxford : Blackwell Science Inc. 1998.

[17] Maeda M ed. Microbial processes in aquaculture. London: Biocreate Press. 1999.
