# Supplementary material for: Autumn ichthyoplankton assemblage in the Yangtze Estuary shaped by environmental factors
Source: PeerJ. 2016 Apr 19;4:e1922. doi: 10.7717/peerj.1922 (PMC4841249; doi:10.7717/peerj.1922)
Supplement: Supplemental Information 1 [file GB¿MT 12763.6-2007 ║ú╤≤╡≈▓Θ╣μ╖╢ ╡┌06▓┐╖╓ú║║ú╤≤╔·╬∩╡≈▓Θ.pdf › GB12763-2007/GB¿MT 12763.6-2007 ║ú╤≤╡≈▓Θ╣μ╖╢ ╡┌06▓┐╖╓ú║║ú╤≤╔·╬∩╡≈▓Θ.pdf]

ICS 07.060
A 45

中华人民共和国国家标准

GB/T 12763.6—2007
代替 GB/T 12763.6—1991

海洋调查规范
第6部分:海洋生物调查

Specifications for oceanographic survey—
Part 6:Marine biological survey

2007-08-13 发布　　　　2008-02-01 实施

中华人民共和国国家质量监督检验检疫总局
中国国家标准化管理委员会　发布

目　　次

前言 …… Ⅶ
1　范围 …… 1
2　规范性引用文件 …… 1
3　术语和定义 …… 1
4　一般规定 …… 3
4.1　技术设计 …… 3
4.2　调查要求 …… 3
4.3　调查和分析仪器设备 …… 4
4.4　采样 …… 4
4.5　样品分析 …… 5
4.6　资料整理及报告编写 …… 5
5　叶绿素、初级生产力和新生产力的测定 …… 6
5.1　技术要求和测定要素 …… 6
5.2　海水浮游植物色素测定 …… 6
5.3　海洋初级生产力(^{14}C示踪法)测定 …… 10
5.4　叶绿素a和初级生产力的粒度分级测定 …… 11
5.5　海洋新生产力(^{15}N示踪法)测定 …… 12
5.6　资料整理 …… 14
6　微生物调查 …… 18
6.1　技术要求和调查要素 …… 18
6.2　采样 …… 19
6.3　样品分析 …… 19
6.4　资料整理 …… 29
7　微微型、微型和小型浮游生物调查 …… 30
7.1　技术要求和调查要素 …… 30
7.2　采样 …… 30
7.3　样品分析 …… 32
7.4　资料整理 …… 33
8　大、中型浮游生物调查 …… 34
8.1　技术要求和调查要素 …… 34
8.2　采样 …… 35
8.3　样品分析 …… 36
8.4　资料整理 …… 37
9　鱼类浮游生物调查 …… 38
9.1　技术要求和调查要素 …… 38
9.2　采样 …… 38
9.3　样品分析 …… 40

9.4 资料整理 …… 40
10 大型底栖生物调查 …… 41
10.1 技术要求和调查要素 …… 41
10.2 采样 …… 41
10.3 样品分析 …… 43
10.4 资料整理 …… 44
11 小型底栖生物调查 …… 44
11.1 技术要求和调查要素 …… 44
11.2 采样 …… 45
11.3 样品分析 …… 46
11.4 资料整理 …… 48
12 潮间带生物调查 …… 48
12.1 技术要求和调查要素 …… 48
12.2 采样 …… 49
12.3 样品分析 …… 51
12.4 资料整理 …… 51
13 污损生物调查 …… 52
13.1 技术要求和调查要素 …… 52
13.2 采样 …… 52
13.3 样品分析 …… 54
13.4 资料整理 …… 55
14 游泳动物调查 …… 56
14.1 技术要求和调查要素 …… 56
14.2 采样 …… 57
14.3 样品分析 …… 58
14.4 资料整理 …… 61
附录 A（规范性附录） 微生物最可能数(MPN)计数及其检索表 …… 63
附录 B（规范性附录） 浮游生物样品编号、生物量测定、计数和鱼类浮游生物网具 …… 66
附录 C（规范性附录） 大型底栖生物固定液配制、样品编号和海底照相与录像 …… 71
附录 D（规范性附录） 小型底栖生物样品分离、计算和几种仪器设备图 …… 73
附录 E（规范性附录） 潮间带生物调查中潮位测量法和几种设备图 …… 79
附录 F（规范性附录） 游泳动物性腺成熟度、摄食强度、含脂量及拖网网型 …… 83
附录 G（资料性附录） 渔业资源声学调查与评估(选做) …… 89
附录 H（资料性附录） 海洋生物调查采样和分析记录表格式 …… 98
参考文献 …… 158

图 B.1 双鼓网图 …… 69
图 B.2 北太平洋网图 …… 70
图 D.1 小型底栖生物分样器结构府面观和侧面观示意图 …… 76
图 D.2 砂质样品封闭淘洗装置示意图 …… 76
图 D.3 海冰法分选小型生物示意图 …… 77

图 D.4 不同体型桡足类的转换系数 …… 77
图 D.5 携带配套的水下呼吸器(SCUBA)等装置的潜水员示意图 …… 78
图 E.1 潮汐水位曲线图解法曲线 …… 80
图 E.2 直接测量法曲线 …… 80
图 E.3a) 覆盖面积计数框 …… 80
图 E.3b) 岩石定量采样框 …… 80
图 E.4 滩涂定量采样框 …… 81
图 E.5 漩涡分选装置 …… 81
图 E.6 过筛器 …… 82
图 F.1 双船底层有翼单囊 A 型拖网网图 …… 86
图 F.2 双船底层有翼单囊 B 型拖网网图 …… 87
图 F.3 单船有翼单囊拖网网图 …… 88
图 G.1 调查专用底层拖网网图 …… 95
图 G.2 调查专用变水层拖网网图 …… 96
图 G.3 声学仪器校正标准目标的悬挂示意图 …… 97

表 1 采水层次 …… 3
表 2 HPLC 梯度洗脱程序 …… 10
表 3 微微型、微型和小型浮游生物垂直分段采样水层 …… 30
表 4 微型和小型浮游生物网具的规格及适用对象 …… 31
表 5 大中型浮游生物垂直分段采样水层 …… 34
表 6 大中型浮游生物网具的规格及适用对象 …… 35
表 7 鱼类浮游生物网具的规格及适用对象 …… 39
表 8 试板种类、规格及数量 …… 53
表 A.1 最可能数(MPN)计数法(15 管)菌数检索表 …… 63
表 B.1 双鼓网构造与规格 …… 69
表 B.2 北太平洋网构造与规格 …… 70
表 D.1 小型底栖生物的换算系数表 …… 74
表 F.1 双船底层有翼单囊 B 型拖网网具第 7 段～第 24 段编结规格 …… 87
表 G.1 鱼类目标强度与体长关系参考值 …… 94
表 H.1 叶绿素采样记录表 …… 98
表 H.2 叶绿素(萃取荧光法)测定记录表 …… 99
表 H.3 叶绿素(分光光度法)测定记录表 …… 100
表 H.4 叶绿素(高效液相色谱法)测定记录表 …… 101
表 H.5 初级生产力采样、过滤、测定记录表 …… 102
表 H.6 新生产力采样、过滤、测定记录表 …… 103
表 H.7 微生物现场采样记录表 …… 104
表 H.8 海洋水体病毒、细菌数量直接计数记录表 …… 105
表 H.9 细菌菌体大小测定记录表 …… 106
表 H.10 水样、泥样微生物培养计数和菌株分离记录表 …… 107
表 H.11 水样细菌生产力记录表 …… 108

表 H.12 水样细菌异养活性测定记录表 …… 109
表 H.13 样品细菌最可能数(MPN)记录表 …… 110
表 H.14 浮游生物海上采样记录表 …… 111
表 H.15 浮游生物垂直分层拖网采样记录表 …… 112
表 H.16 浮游生物样品登记表 …… 113
表 H.17 微微型光合浮游生物细胞数量记录表 …… 114
表 H.18 微型和小型浮游生物标本个数计数记录表 …… 115
表 H.19 采水浮游生物标本个数计数记录表 …… 116
表 H.20 浮游植物(孢囊)细胞记录表 …… 117
表 H.21 浮游动物体积分数测定记录表 …… 118
表 H.22 浮游动物湿重生物量测定记录表 …… 119
表 H.23 浮游动物干重生物量测定记录表 …… 120
表 H.24 浮游动物个体计数记录表 …… 121
表 H.25 夜光藻个体计数记录表 …… 122
表 H.26 鱼类浮游生物海上采集记录表 …… 123
表 H.27 鱼类浮游生物标本登记表 …… 124
表 H.28 鱼类浮游生物计数记录表 …… 125
表 H.29 鱼类浮游生物数量统计表 …… 126
表 H.30 大型底栖生物海上采样记录表 …… 127
表 H.31 大型底栖生物定量分析记录表 …… 128
表 H.32 大型底栖生物定性分析记录表 …… 129
表 H.33 大型底栖生物定量分析种类分布记录表 …… 130
表 H.34 大型底栖生物定性分析种类分布记录表 …… 131
表 H.35 小型底栖生物海上采样记录表 …… 132
表 H.36 小型底栖生物定量分析记录表 1 …… 133
表 H.37 小型底栖生物定量分析记录表 2 …… 134
表 H.38 小型底栖生物定性分析记录表 …… 135
表 H.39 潮间带生物野外采集记录表 …… 136
表 H.40 潮间带生物定量分析记录表 …… 137
表 H.41 潮间带生物定性分析记录表 …… 138
表 H.42 潮间带生物种类分布表 …… 139
表 H.43 潮间带生物主要种类垂直分布表 …… 140
表 H.44 潮间带生物统计表 …… 141
表 H.45 微型污损生物记录表 …… 142
表 H.46 船舶污损生物记录表 …… 143
表 H.47 浮标污损生物记录表 …… 144
表 H.48 码头、桩柱污损生物记录表 …… 145
表 H.49 污损生物分析记录表 …… 146
表 H.50 污损生物种类记录表 …… 147
表 H.51 游泳动物拖网卡片 …… 148
表 H.52 鱼类生物学测定记录表 …… 149

表 H.53　虾类生物学测定记录表 …… 150
表 H.54　蟹类生物学测定记录表 …… 151
表 H.55　头足类生物学测定记录表 …… 152
表 H.56　游泳动物数量统计表 …… 153
表 H.57　鱼类体长测定统计表 …… 154
表 H.58　鱼类体重测定统计表 …… 155
表 H.59　鱼类怀卵量记录表 …… 156
表 H.60　声学调查观测记录表 …… 157

前　言

GB/T 12763《海洋调查规范》分为11个部分：

——第1部分：总则；

——第2部分：海洋水文观测；

——第3部分：海洋气象观测；

——第4部分：海洋化学要素调查；

——第5部分：海洋声、光要素调查；

——第6部分：海洋生物调查；

——第7部分：海洋调查资料交换；

——第8部分：海洋地质地球物理调查；

——第9部分：海洋生态调查指南；

——第10部分：海底地形地貌调查；

——第11部分：海洋工程地质调查。

其中第9部分、第10部分和第11部分对应于GB/T 12763—1991是新增部分。

本部分为GB/T 12763的第6部分，并代替GB/T 12763.6—1991《海洋调查规范　海洋生物调查》。

本部分与GB/T 12763.6—1991相比主要变化如下：

——调整了本部分的总体框架，增加了“12　潮间带生物调查”、规范性附录“潮间带生物调查中潮位测量法和几种设备图”(见附录E)和资料性附录“渔业资源声学调查与评估”(见附录G)；将1991年版的规范性附录“海水中叶绿素a、b、c的(分光光度法)测定”移至修改后的“5　叶绿素、初级生产力和新生产力的测定”中(1991年版的附录A；本版的5.2.2)；将原版的“第四篇浮游生物调查”修改后细分为“7　微微型、微型和小型浮游生物调查”、“8　大、中型浮游生物调查”和“9　鱼类浮游生物调查”三章，其中“7　微微型、微型和小型浮游生物调查”为新增的调查项目；

——在“2　规范性引用文件”中，增加了GB 17378.7海洋监测规范第7部分：近海污染生态调查和生物监测；

——在“3　术语和定义”中，增加和修改了有关术语(1991年版的3.4和3.7；本版的3.2、3.3、3.4、3.5、3.8、3.9、3.10和3.11)；同时每个术语都增补了对应的英语名称；

——在“4　一般规定”中，调查项目和主要仪器设备两条分别增补了新的测项和新的仪器设备(1991年版的5.1和6；本版的4.2.1和4.3)；在本版的“表1采水层次”中增加了200 m以深的采水层次(见4.2.4.1)；进一步规定了调查次数，细化了调查季度的时间(1991年版的5.5.2；本版的4.2.5)；修改和扩展了实验室的使用范围(1991年版的6.2.1，本版的4.3.2)；根据本次修订后的《海洋调查规范　第1部分：总则》GB/T 12763.1—2007的规定，航次报告和调查成果报告由原来的技术负责人编写修订为分别由首席科学家和项目负责人主持编写(1991年版的9.2.1和9.2.2，本版的4.6.1.4和4.6.1.5)等；

——在“5　叶绿素、初级生产力和新生产力测定”中，增加了“高效液相色谱(HPLC)法”、“叶绿素a和初级生产力的粒度分级测定”和“海洋新生产力(^{15}N示踪法)测定”(见5.2.3、5.4和5.5)；

——在“6　微生物调查”中，增加了“SYBR Green I直接计数法”、“DAPI直接计数法”、“细菌体积测定”、“^{3}H-亮氨酸示踪法”测定细菌生产量、“生态呼吸率测定”、“细菌比生长率、倍增时间和

世代时间的计算”和“微生物分类鉴定”(见 6.3.4.1.1.1、6.3.4.1.1.3、6.3.4.1.1.4、6.3.4.2.1.2、6.3.4.2.2.2、6.3.4.3 和 6.3.5);

——“7 微微型、微型和小型浮游生物调查”为新增的章;

——在“8 大、中型浮游生物调查”中,增加了网具类型,修改了连续观测频率、样品处理、样品编号和制图取值标准(1991 年版的 20.1.2、19.6、20.3.4、21.1 和 22.6;本版的 8.2.1.1、8.1.1.3、8.2.3.2、8.3.1 和 8.4.4);

——在“9 鱼类浮游生物调查”中,修改了采集网具和丰度计算公式(1991 年版的 20.1.2 和 22.4;本版的 9.2.1.1 和 9.4.1);

——在“10 大型底栖生物调查”中,增加了“深拖光学系统和深海底栖生物拖网”和“大型底栖生物海底照相、录像与潜水采样”(见 10.2.1.2.5 和附录 C.4);

——在“11 小型底栖生物调查”中,增加了“潜水取样”、“潮间带采芯样”、“硅溶胶 Ludox—TM 离心漂浮法”和群落结构与生物多样性多元统计分析(见 11.2.1.6 和 11.2.2.2、11.2.2.1、11.3.4和附录 D.4);修改了采样设备(1991 年版的 28.1;本版的 11.2.1);

——“12 潮间带生物调查” 为新增的章;

——在“13 污损生物调查”中,修改了技术要求内容(1991 年版的 31;本版的 13.1.1),增加了调查要素和水泥试板用于海岸港工建设的污损生物调查(13.1.2 和 13.2.1.1.1c));

——在“14 游泳动物调查”中,将“性腺成熟系数”和“摄食饱满系数”纳入“资料整理”(1991 年版的 37.3.1.5 和 37.3.1.6;本版的 14.4.2.4 和 14.4.2.5)。修改和增加了一些重要渔获物的性腺成熟度和鱼类含脂量的划分标准(见本版的附录 F.1.2、附录 F.1.3、附录 F.1.4、附录 F.1.6、附录 F.1.7、附录 F.1.8 和附录 F.3);修改了游泳生物拖网网型(1991 年版的附录 F7;本版的附录 F.4);

——修改和增加了资料性附录“海洋生物调查、分析记录表格式”(1991 年版的附录 G;本版的附录 H)。

——本部分应与 GB/T 12763.1 和 GB/T 12763.7 配套使用。

本部分的附录 A、附录 B、附录 C、附录 D、附录 E 和附录 F 为规范性附录,附录 G 和附录 H 为资料性附录。

本部分由国家海洋局提出。

本部分由国家海洋标准计量中心归口。

本部分由国家海洋局第三海洋研究所负责修订,国家海洋局第一海洋研究所、国家海洋局第二海洋研究所、中国海洋大学、中国水产科学研究院东海水产研究所、中国水产科学研究院黄海水产研究所等参加修订。

本部分的主要起草和修订人:张玉生、杨清良、陈瑞祥、朱明远、叶德赞、宁修仁、林景宏、戴燕玉、江锦祥、张志南、李荣冠、黄宗国、郑成兴、郑元甲、赵宪勇、吕瑞华、陆斗定、史君贤、蔡昱明、林茂和周红。

本部分所代替标准的发布情况为:

——GB/T 12763.6—1991。

海洋调查规范
第6部分:海洋生物调查

1 范围

GB/T 12763 的本部分规定了海洋生物调查的一般规定、技术要求和调查(测定)要素、采样、样品分析及资料整理的基本要求和方法。

本部分适用于海洋环境基本要素调查中的海洋生物调查。

2 规范性引用文件

下列文件中的条款通过 GB/T 12763 的本部分的引用而成为本部分的条款。凡是注日期的引用文件,其随后所有的修改单(不包括勘误的内容)或修订版均不适用于本部分,然而,鼓励根据本部分达成协议的各方研究是否可使用这些文件的最新版本。凡是不注日期的引用文件,其最新版本适用于本部分。

GB/T 14014 蚕丝、合成纤维筛网

GB/T 12763.1 海洋调查规范 第1部分:总则

GB/T 12763.7 海洋调查规范 第7部分:海洋调查资料处理

GB 17378.7 海洋监测规范 第7部分:近海污染生态调查和生物监测

3 术语和定义

GB/T 15919 确立的以及下列术语和定义适用于 GB/T 12763 的本部分。

3.1

叶绿素 chlorophyll

自养植物细胞中一类很重要的色素,是植物进行光合作用时吸收和传递光能的主要物质。叶绿素 a(Chl a)是其中的主要色素。

3.2

初级生产力 primary productivity

自养生物通过光合作用生产有机物的能力。通常以单位时间(年或天)内单位面积(或体积)中所产生的有机物(一般以有机碳表示)的质量计算,相当于该时间内相同面积(或体积)中的初级生产量。

[GB/T 15919—1995, 定义 2.210]

3.3

碳同化数 carbon assimilation number

指植物光合色素的光合作用效率。在 CO_2 与光照度充足的条件下,单位质量叶绿素与每小时所同化的碳量之比[常用"碳(毫克)/叶绿素(毫克)/小时"表示]。

[GB/T 15919—1995, 定义 2.217]

3.4

新生产力 new productivity

在真光层中再循环的氮为再生氮,由真光层之外提供的氮为新生氮。由再生氮源支持的那部分初级生产力称为再生生产力,由新生氮源支持的那部分初级生产力称为新生产力。

3.5

微生物　microbe

一群个体微小、结构简单、生理类型多样的单细胞或多细胞的低等生物，包括属于原核生物类的细菌、放线菌、支原体、立克次体、衣原体和蓝细菌，属于真核生物类的真菌（酵母菌和霉菌）、原生动物和显微藻类，以及属于非细胞生物类的病毒、类病毒和朊病毒。

3.6

细菌异养生长速率　bacterial heterotrophic growth rate

异养细菌利用有机物进行生长繁殖的速率。

3.7

细菌异养活性　bacterial heterotrophic activity

异养细菌进行生理代谢活动的能力。

3.8

细菌生产力　bacterial productivity

单位时间内、单位水体所产生的细菌生物量。

3.9

浮游生物　plankton

缺乏发达的运动器官，没有或仅有微弱的运动能力，悬浮在水层中，常随水流移动的生物。包括浮游植物和浮游动物两大类。

［GB/T 15919—1995，定义 2.126］

依个体的大小浮游生物可分为以下几种类型：粒径小于 2 μm 的称微微型浮游生物（picoplankton）；粒径为 2 μm～20 μm 的称微型浮游生物（nanoplankton）；粒径为 20 μm～200 μm 的称小型浮游生物（microplankton 或 netplankton）；粒径为 200 μm～2 000 μm 的称中型浮游生物（mesoplankton）；粒径为 2 000 μm～20 mm 的称大型浮游生物（macroplankton）；粒径大于 20 mm 的称巨型浮游生物（megaplankton）。

此外，鱼类浮游生物（ichthyoplankton）即为鱼卵和仔稚鱼。

3.10

底栖生物　benthos

栖息在水域基底表面或底内的生物。在海洋中，这类生物自潮间带至水深大于万米以上的超深渊带（深海沟底部）都有分布，是海洋生物中种类最多的一个生态类型，包括了大多数海洋动物门类，大型和微型定生海藻类和海洋种子植物。

［GB/T 15919—1995，定义 2.150］

依个体的大小，凡被孔径为 0.5 mm 套筛网目所截留的生物，称为大型底栖生物（macrobenthos）。凡能通过孔径为 0.5 mm 套筛网目，而被孔径为 0.042 mm 所截留的生物，称为小型底栖生物（meiobenthos）。

3.11

潮间带生物　intertidal benthos

生活在潮间带底表的植物和底表与底内的动物。

3.12

污损生物　fouling organism

生长在船底、浮标、平台和海中一切其他设施表面或内部的生物。这类生物一般是有害的。

［GB/T 15919—1995，定义 2.281］

3.13

游泳动物 nekton

具有发达的运动器官，在水层中能克服水流阻力自由游动的动物。如鱼类、大型虾、蟹类、头足类及海洋哺乳动物等。

[GB/T 15919—1995，定义 2.146]

4 一般规定

4.1 技术设计

根据调查任务进行技术设计，其内容包括调查断面、站位、项目、内容(含要素)、方法、时间、次数、专业配置、人员素质、船只、器材设备、预期成果等和调查计划编制。应特别注重海上采样和室内分析的技术要求和措施保障。调查计划编制的要求见 GB/T 12763.1 中的相关章节。

4.2 调查要求

4.2.1 调查项目

海洋生物调查项目包括：叶绿素、初级生产力和新生产力，微生物，微微型、微型和小型浮游生物，大、中型浮游生物，鱼类浮游生物，大型底栖生物，小型底栖生物，潮间带生物，污损生物和游泳动物。必要时，应包括渔业资源声学调查与评估。

4.2.2 辅助参数

海洋生物调查时，应视需要确定与其有关的辅助参数，并同步进行观测。

4.2.3 调查方式

海洋生物调查方式包括：大面观测、断面观测和连续观测。

4.2.4 采样方法、采样种类

4.2.4.1 采水样

适用于叶绿素浓度、初级生产力和新生产力，微生物，微微型、微型和小型浮游生物等调查项目的水样采集。应按规定水层采样(见表 1)。

表 1 采水层次

单位为米

测站水深范围	标准层次	底层与相邻标准层的最小距离
<15	表层、5、10、底层	2
15～50	表层、5、10、30、底层	2
50～100	表层、5、10、30、50、75、底层	5
100～200	表层、5、10、30、50、75、100、150、底层	10
>200	表层、5、10、30、50、75、100、150、200	

注 1：表层指海面下 0.5 m 深度以内的水层。

注 2：水深小于 50 m 时，底层为离底 2 m 的水层。

注 3：水深在 50～200 m 时，底层为离底 5 m 的水层。

注 4：可根据调查的特殊需要，酌情增加 200 m 以深的采水层次。

注 5：条件许可时，应充分考虑跃层和采集叶绿素次表层最大值所处的水层。

4.2.4.2 拖网采样

适用于大、中型浮游生物、鱼类浮游生物、大型底栖生物、游泳动物调查和渔业资源声学调查与评估等项目的采样。

4.2.4.3 底质采样

适用于微生物、潮间带生物和大、小型底栖生物调查项目的采样。

4.2.4.4 挂板和水面或水中设施上采样

适用于污损生物调查的采样。

4.2.5 调查时间、调查次数和季节划分

调查时间和调查次数应根据调查水域环境条件和调查目的确定。

a) 河口、港湾、沿岸海区和边缘海(marginal seas)调查

——受气象、流系的季节性影响显著的边缘海应每季度调查一次;受气候、水文的季节性影响明显且物质来源复杂的河口、港湾和沿岸海区,通常应每月(至少每季度)调查一次(潮间带生物每年调查2次~4次);如有特殊需要可酌情调整调查次数。若进行逐季或逐月调查,各季或各月调查的时间间隔应基本相等。进行河口、港湾调查时,应充分考虑潮汐的影响;

——一般以3月~5月为春季,6月~8月为夏季,9月~11月为秋季,12月~翌年2月为冬季,并分别以5月、8月、11月和2月代表春季、夏季、秋季和冬季。但热带海域应根据具体的海洋环境条件和调查目的酌情调整调查时间和调查次数。

b) 大洋和极地海域调查

应根据调查目的,选择调查时间,确定调查次数。

4.2.6 定位

按GB/T 12763.1的有关规定进行。

4.2.7 出海准备

海上所需物品,均应计算实用量和备用量;安装仪器设备,存放工具器皿,以安全方便为原则。

4.3 调查和分析仪器设备

4.3.1 主要仪器设备

调查和分析的主要仪器设备有采水器、网口流量计、各种网具及附件、底质采样器、漩涡分选装置、(水下)照相与摄影设备、探鱼仪(回声探测-积分系统);生物分类鉴定、计数、测定和称量的器械(如光学显微镜、倒置显微镜、落射荧光显微镜、分析天平等);离心、干燥、冷藏和烘干的设备;分光光度计、荧光计、液闪计数仪、质谱仪和高效液相色谱仪(HPLC)等仪器。

仪器设备、标准物质和试剂配备按GB/T 12763.1和本部分的有关规定进行。

4.3.2 调查船实验室

4.3.2.1 一般实验室

一般实验室应适用于叶绿素、浮游生物、底栖生物、潮间带生物、污损生物和游泳动物等样品处理和分析。

4.3.2.2 放射性实验室

放射性实验室应适用于初级生产力、新生产力和微生物样品应用^{14}C、^{15}N和^{3}H进行的分析测定,要求具有通风和放射性防护设施。放射性实验室与接触^{14}C、^{15}N和^{3}H的仪器设备都应具有明显标志,并定期检测放射性强度。

4.3.2.3 微生物实验室

微生物实验室应适用于微生物样品的处理,并应具备无菌操作设施。

4.3.3 调查船其他设施

a) 绞缆机,钢丝绳,起网吊杆,双拖网或单拖网(含网板)及探鱼仪等,均需适应大型底栖生物和游泳动物拖网的要求;

b) 动力绞车、钢丝绳、吊杆、海(淡)水源、工作电源、甲板工作空间和场所等条件,均需满足海洋生物各调查项目的采样要求。

4.4 采样

4.4.1 采样要求

4.4.1.1 采样位置

海洋生物调查现场采样时,应避开调查船的排污口。

4.4.1.2 采水样

使用调查项目规定的采水器采水，入水前应检查采水器的球盖是否打开，出水嘴是否关闭，准确放至预定水层，严守停滞时间。按要求取样、处理。

4.4.1.3 拖网采样

使用专业规定的网具采样，严格起、落网速度，准确判断网具到达预定水层。拖网时注意工作状态是否正常，遇异常情况应立即采取有效措施。起网后认真冲洗网具，收集样品，特别是粘附在网衣和网底管套筛绢上的生物样品严禁标本挟带。

4.4.1.4 采泥样

使用调查项目规定的采样器采样，严守操作程序，注意采样器的工作状态。按要求取样和处理。发现异常应重新采样。

4.4.1.5 挂板和水中设施上采样

按项目要求制板，正确选定挂板地点、挂板方式和采样设施。严格执行采样时间、取板程序和样品处理方法。

4.4.2 记录

各调查项目应按 GB/T 12763.1 和本部分的有关规定记录。样品采集、分析、鉴定和测定记录表的格式规定见本部分附录 H。

遇异常现象或新发现，除记录外还应现场拍照或录像。

4.4.3 采样工具、设备的要求

应满足 GB/T 12763.1 和本部分的有关规定。

4.5 样品分析

4.5.1 样品处理

各调查项目采得的样品应按 GB/T 12763.1 和本部分有关规定的具体要求处理。

4.5.2 样品测定

需要测定的样品，应按本部分相应调查项目的要求进行。

4.5.3 鉴定、计数

主要生物种类，一般应鉴定到种，并按本部分相应专业的要求计数。

4.5.4 样品保存

分析、测量、鉴定后的样品，可依资料分析、应用程度和学术价值高低，确定全部或部分保留。保留样品应按各专业的要求保管。

4.6 资料整理及报告编写

4.6.1 资料整理

4.6.1.1 计算、统计

鉴定、计数及测定结果按本部分各调查要素所规定的公式和格式进行计算、统计。

4.6.1.2 填写报表

按 GB/T 12763.1 和本部分的有关规定，并参考 GB/T 12763.7 的有关要求，填写各类报表。

4.6.1.3 绘制图表

有关数据资料形成后，根据本部分各调查要素的要求绘制各式图表。

4.6.1.4 编写航次报告

每航次海上调查完成后，首席科学家应按 GB/T 12763.1 有关规定主持编写航次报告。

4.6.1.5 编写调查报告

调查任务完成后，项目负责人应按 GB/T 12763.1 的有关规定和本部分各项目的调查结果主持编写调查成果报告。

4.6.2 资料归档、验收及成果鉴定

按 GB/T 12763.1 有关规定进行资料归档、验收和成果鉴定。

5 叶绿素、初级生产力和新生产力的测定

5.1 技术要求和测定要素

5.1.1 技术要求

5.1.1.1 叶绿素 a 测定

5.1.1.1.1 采样层次

见 4.2.4.1 表 1;条件许可时,应加采跃层上、跃层中、跃层下三层。

5.1.1.1.2 精密度

叶绿素 a 浓度在 0.5 mg/m^3 水平时,重复样品的相对误差为±10%。

5.1.1.2 初级生产力测定

5.1.1.2.1 采样层次

按光学深度,在光强为表层的 100%、50%、30%、10%、5%和 1%的深度上采水样,特殊情况可视要求而定。

5.1.1.2.2 测定范围

测定范围为 0.05 mg/(m^3 · h)~100 mg/(m^3 · h)。

5.1.1.2.3 精密度

初级生产力测定的精密度,当初级生产力在 30 mg/(m^3 · h)水平时,重复样品的相对误差为±10%。(培养 3h,加强度为 185 kBq 的放射性^{14}C)。

5.1.1.3 新生产力测定

条件允许或有特殊需要时,应进行新生产力测定,其技术要求为:

a) 培养瓶及采样器皿须酸洗以除去或尽量减少金属沾污;从采样到培养之间的时间间隔要尽量短,这期间要尽量避免阳光直射以减少光休克效应;在样品处理、过滤及同位素分析中,应避免外源氮的污染;
b) 当采用气体质谱时,无法测得$^{15}NH_4$-N 的^{15}N 的丰度和浓度,应注意由$^{15}NH_4$-N 再生同位素稀释效应导致的负误差。在无其他选择时可缩短培养时间至 2 h;
c) 实验过程中颗粒有机氮的增加会导致氮的吸收速率的低估;如果水体的生物量和生产率很高,可缩短培养时间以减少上述误差。

5.1.2 测定要素

测定要素为叶绿素、初级生产力和新生产力。

5.2 海水浮游植物色素测定

5.2.1 萃取荧光法(叶绿素 a)

5.2.1.1 方法原理

叶绿素 a 的丙酮萃取液受蓝光激发产生红色荧光,过滤一定体积海水所得的浮游植物用 90%丙酮提取其色素,使用荧光计测定提取液酸化前后的荧光值,计算出海水中叶绿素 a 的浓度。

5.2.1.2 主要仪器设备

a) 荧光计:激发光波长 450 nm,发射光波长 685 nm;
b) 抽滤装置:包括滤器、支架、抽滤瓶和真空泵;
c) 玻璃纤维滤膜:其截留效率相当于 0.65 μm 孔径的聚碳酸酯微孔滤膜;
d) 冰箱。

5.2.1.3 试剂

体积分数为 90%的丙酮、体积分数为 10%的盐酸、碳酸镁溶液 $\rho(MgCO_3)=10$ g/dm^3。

5.2.1.4 测定步骤

5.2.1.4.1 荧光计校准

5.2.1.4.1.1 校准频率

至少每半年一次。

5.2.1.4.1.2 标准叶绿素 a 溶液(ρ=1 mg/dm^3)制备

过滤一定量的生长良好、处于指数生长前期的培养硅藻，用 90%丙酮提取其叶绿素 a，或者用 90%丙酮溶解一定量的市售叶绿素 a 结晶，浓度大约为 ρ=1 mg/dm^3。

5.2.1.4.1.3 标准叶绿素 a 溶液浓度标定

使用分光光度计正确测定标准叶绿素 a 溶液的浓度。

5.2.1.4.1.4 叶绿素 a 标准工作溶液配制

用上述标准叶绿素 a 溶液配制浓度不同的标准工作溶液，供各量程档校准用。

5.2.1.4.1.5 换算系数 F_d 的测定

上述不同浓度的标准工作溶液，在不同量程档上进行酸化前后荧光值的测定。各量程档的换算系数 F_d 的计算公式：

$$F_d = \frac{\rho(\text{Chl a})}{R_1 - R_2} \quad \cdots\cdots(1)$$

式中：

F_d——量程档“d”的换算系数，单位为毫克每立方米(mg/m^3)；

ρ(Chl a)——叶绿素 a 的标准工作溶液的浓度，单位为毫克每立方米(mg/m^3)；

R_1——酸化前的荧光值；

R_2——酸化后的荧光值。

5.2.1.4.2 水样测定

5.2.1.4.2.1 采样

按 4.2.4.1 表 1 规定的深度采水样，并记录于表 H.1。

5.2.1.4.2.2 过滤

采样后，应尽快过滤。过滤海水的体积视调查海区而定，富营养海区一般可过滤 50 cm^3～100 cm^3；中营养海区过滤 200 cm^3～500 cm^3；寡营养海区可过滤 500 cm^3～1 000 cm^3。过滤时抽气负压应小于 50 kPa，记录于表 H.1。

5.2.1.4.2.3 滤膜保存

过滤后的滤膜应在 1 h 内提取，若无条件提取测量，可将滤膜对折，用铝箔包好，存放于低温冰箱(−20℃)，保存期可为 60 d 或放入液氮中保存期可为一年。

5.2.1.4.2.4 提取

将载有浮游植物的滤膜放入加有 10 cm^3 体积分数为 90%丙酮的提取瓶内，盖紧，摇荡，立即放于低温(0℃)冰箱内，提取 12 h～24 h。

5.2.1.4.2.5 荧光测定

测定步骤如下：

a) 取出样品放在室温、黑暗处约 0.5 h，使样品温度与室温一致；
b) 每批样品测定前后，以体积分数为 90%丙酮作对比液，测出各量程档的空白荧光值 F_{01} 和 F_{02}；
c) 将提取瓶内上清液倒入测定池中，选择适当量程档，测定样品的荧光值 R_b；
d) 加 1 滴体积分数为 10%盐酸于测定池中，30 s 后测定其荧光值 R_a；
e) 将结果记录于表 H.2。

5.2.2 分光光度法(包括叶绿素 a、b 和 c)

5.2.2.1 方法原理

叶绿素 a、b、c 的丙酮萃取液在红光波段各有一吸收峰。一定体积海水中的浮游植物经滤膜滤出，

用90%丙酮提取其叶绿素，应用分光光度计测定，根据三色分光光度法方程，计算海水中叶绿素a、b、c的浓度。

5.2.2.2 试剂

碳酸镁溶液：$\rho(MgCO_3)=10\ mg/dm^3$；体积分数为90%的丙酮。

5.2.2.3 主要仪器设备

主要仪器设备有以下几种：

a) 分光光度计：波长必须准确，波带宽度≤2 nm，消光值可读到0.001；
b) 抽滤装置：见5.2.1.2 b)；
c) 滤膜：截留效率相当于0.65 μm孔径的聚碳酸酯核孔滤膜或玻璃纤维滤膜、纤维素酯微孔滤膜或其他滤膜；
d) 贮样干燥器、研磨器、离心机、具塞离心管和冰箱。

5.2.2.4 测定步骤

5.2.2.4.1 采样

见5.2.1.4.2.1。

5.2.2.4.2 过滤

采样后应尽快过滤。将滤膜置于滤器上，加5 cm^3 碳酸镁溶液，接着过滤海水样，过滤时负压应小于50 kPa。过滤海水体积视调查水域而定，近岸水取0.5 dm^3～2 dm^3，外海水取5 dm^3～10 dm^3。

5.2.2.4.3 保存

过滤后的样品应立即研磨提取。若条件不允许，可将滤膜对折两次，置于贮样干燥器内低温(<−20℃)黑暗保存，期限最长两个月。

5.2.2.4.4 研磨

将载有浮游植物的滤膜放入研磨器，加2 cm^3 或3cm^3体积分数为90%的丙酮，研磨，后将样品移入具塞离心管中，研磨器用体积分数为90%丙酮洗涤2次或3次，洗涤液一并倒入离心管中，但总体积不能超过10 cm^3。

5.2.2.4.5 提取

将具塞离心管置于低温黑暗处提取30 min。

5.2.2.4.6 离心

提取液于4 000 r/min条件下离心10 min，上清液倒入刻度试管中，并定容为10 cm^3 或15 cm^3。

5.2.2.4.7 测定

将提取液注入光程为1 cm～10 cm的比色槽中，以体积分数为90%丙酮作空白对照，用分光光度计测定波长为750 nm、664 nm、647 nm、630 nm处的溶液消光值。测定结果记录于表H.3。

5.2.2.5 消光值选择

作浊度校正的750 nm处消光值不超过每厘米光程0.005，664 nm处消光值最好在0.1～0.8之间。

5.2.3 高效液相色谱(HPLC)法

5.2.3.1 方法原理

浮游植物所含各种光合色素经提取后，在一定溶剂系统中，可经高效液相色谱柱进行分离，再由检测器检测并获得色谱图。根据与标准色素比较保留时间及色谱图可鉴别色素种类，根据色谱峰的面积可计算含量。

5.2.3.2 主要仪器设备

a) 高效液相色谱仪：包括溶剂流动相系统、高压泵、进样器、色谱柱、检测器、计算机等；
b) 抽滤装置、玻璃纤维滤膜、液氮罐、超声波粉碎器和离心机等。

5.2.3.3 试剂

丙酮、水、甲醇、乙腈和乙酸乙酯(均为色谱纯);醋酸铵;BHT(2,6-二叔丁基对甲酚)、角黄素。

5.2.3.4 测定步骤

5.2.3.4.1 采样

同 5.2.1.4.2.1。

5.2.3.4.2 过滤

采样后,应尽快过滤,视海水中浮游植物数量,过滤 0.5 dm^3～4 dm^3 海水(贫营养海区 3 dm^3～4 dm^3,中等营养海区 1 dm^3～2 dm^3,富营养海区 0.5 dm^3～1 dm^3),过滤时使用孔径为 0.65 μm、直径为 25 mm 的玻璃纤维滤膜,抽滤负压不超过 50 kPa,并注意避光。

5.2.3.4.3 保存

过滤后,如不能立即提取,滤膜应保存在液氮罐中。在放入液氮或超低温冷冻之前,冷冻保存(－20℃)不能超过 20 h。滤膜可用预先标记好的铝箔包裹保存。

5.2.3.4.4 萃取

将滤膜从液氮中取出,解冻约 1 min,放入玻璃离心管中,加入 3$cm^3$90%丙酮,再加入 50 mm^3 角黄素作内标准物(角黄素亦可预先加在提取溶剂丙酮中)。用体积分数为 90%丙酮提取一张玻璃纤维滤膜作空白样。混合物用超声波粉碎(50 W, 30 s),然后在 0℃条件下提取 24 h。分析前将提取物混匀后离心去除细胞残屑,并用载有聚四氯乙烯滤膜(直径 13mm,孔径 0.2 μm)的注射式滤器过滤。

5.2.3.4.5 高效液相色谱测定

5.2.3.4.5.1 HPLC 系统准备

a) 高压进样阀(带有 200 mm^3 样品环);
b) C-18 保护柱(50 mm×4.6 mm);
c) 反相 C-18 色谱分析柱(250 mm×4.6 mm);
d) 紫外-可见吸收检测器(测定波长为 436 nm 和 450 nm);
e) 配有数据处理系统软件的计算机;
f) HPLC 溶剂:
 ——溶剂 A:甲醇∶0.5 mol 醋酸铵(80∶20), 0.01%BHT;
 ——溶剂 B:乙腈∶水(87.5∶12.5), 0.01%BHT;
 ——溶剂 C:乙酸乙酯。

以上溶剂均用色谱纯,使用前以 0.45 μm 滤膜过滤。

5.2.3.4.5.2 操作步骤

a) 用溶剂 A 建立并平衡 HPLC 系统 1 h,流量为 1 cm^3/min;
b) 根据所测样品的浓度范围,每种色素准备至少 5 个浓度的工作标准液,并以该标准液校正 HPLC 系统;
c) 取每种工作标准液 1 000 mm^3,以 300 mm^3 蒸馏水稀释,混匀并平衡 5 min,润洗样品注射器两次,进样 500 mm^3(样品环体积的 2.5 倍);
d) 色素样品和空白样的准备及进样方法同标准液。注意进样时应避免有气泡,样品间的进样间隔应保持均匀。预先混有蒸馏水或其他溶剂的样品不能滞留在自动进样器中,以免疏水性的色素从溶液中析出;
e) 进样后通过梯度洗脱程序(见表 2)对叶绿素和类胡萝卜素进行最佳分离,分析过程中通过氦气或在线脱气机对流动相溶液进行脱气;
f) 根据比较色素样品与标准的色谱峰的保留时间来鉴定样品的色素种类,收集各洗脱峰可进一步进行分光确定;
g) 填写表 H.4。

5.2.4 以上三种方法以萃取荧光法优先

5.3 海洋初级生产力(^{14}C 示踪法)测定

5.3.1 方法原理

一定数量的放射性碳酸氢盐 $H^{14}CO_3^-$ 或碳酸盐 $^{14}CO_3^{2-}$ 加入到已知二氧化碳总浓度的海水样品中，经一段时间培养，测定浮游植物细胞内有机 ^{14}C 的量，即可计算浮游植物通过光合作用合成有机碳的量。

5.3.2 主要仪器设备

a) 水下光量子仪、水下照度计或透明度盘；

b) 样品培养箱或培养瓶罩：用中性衰光材料，将光衰减为 100%，50%，30%，10%，5%和 1%；

c) 抽滤装置：见 5.2.1.2 b)；

d) 滤膜：孔径为 0.65 μm 的纤维素酯微孔滤膜；

e) 液体闪烁计数仪、通风橱和振荡器。

5.3.3 试剂

a) 过滤海水

用调查海区的海水，经玻璃纤维或纤维素酯微孔滤膜过滤，盛于干净的试剂瓶中备用。

b) ^{14}C 工作溶液

用过滤海水稀释 ^{14}C 贮备液，使放射性强度约为 1 850 kBq/cm^3，或视要求而定。

c) 闪烁液

d) 测总计数闪烁液

每 10 cm^3 闪烁液中加 0.2 cm^3 的苯乙胺。

e) 0.1 mol/dm^3 盐酸。

表 2 HPLC 梯度洗脱程序

时间/min	流量/($cm^3 \cdot min^{-1}$)	A%	B%	C%	状态
A. 分析步骤					
0.0	1.0	100	0	0	进样
2.0	1.0	0	100	0	梯度洗脱
2.6	1.0	0	90	10	梯度洗脱
13.6	1.0	0	65	35	梯度洗脱
18.0	1.0	0	31	69	梯度洗脱
23.0	1.0	0	31	69	保持
25.0	1.0	0	100	0	梯度洗脱
26.0	1.0	100	0	0	梯度洗脱
34.0	1.0	100	0	0	保持
B. 结束步骤					
0	1.0	100	0	0	分析完成
3.0	1.0	0	100	0	梯度洗脱
6.0	1.0	0	0	100	梯度洗脱
16.0	1.0	0	0	100	冲洗
17.0	1.0	0	0	100	结束

5.3.4 测定步骤

5.3.4.1 萃灭校正方程的确定

用一系列(一般不少于6个)^{14}C萃灭标准瓶,在液闪计数仪上测定。用直线回归法得出比求效率的方程。

5.3.4.2 采样深度的确定

使用水下光量子仪或照度计,测定水柱中不同深度的光辐射强度,或者用透明度盘测定海水的透明度,确定采样的光学深度。

5.3.4.3 水样测定

5.3.4.3.1 采样

按预定深度采样,并记录于表H.5。采样时应使用不透光和没有铜制部件的采水器,水样避免阳光直接照射。

5.3.4.3.2 水样分装

采样后,尽快在弱光下,将水样经孔径为200 μm左右的筛绢过滤,分装至培养瓶。培养瓶必须清洗干净,并经体积分数为2%的稀盐酸浸泡24 h以上。每层样品应包括两个白瓶和一个黑瓶,第一层和第四层样品还应各分装一个零时间培养瓶(或根据现场调查情况选定),零时间培养瓶中水样体积必须准确。剩余水样按5.2.1的规定,测定各层次水样的叶绿素a浓度。

5.3.4.3.3 加^{14}C工作液

取相同体积的^{14}C工作溶液加至每个培养瓶。所加数量视样品中浮游植物多少和培养时间而定。一般在水深小于200 m海区,培养24 h,加37 kBq～370 kBq,水深大于200 m海区加370 kBq～740 kBq。

5.3.4.3.4 取总计数样

用微量吸液器从每一零时间培养瓶中吸取一定体积水样两份,分别移入2个总计数闪烁瓶中,加20 cm^3闪烁液,盖紧,混匀,供作放射性活度测定。

5.3.4.3.5 培养

将已加有^{14}C的培养瓶(零时间培养瓶除外),放入各相应的培养箱内,或罩上各相应的培养罩,再放入透明的培养箱内。记下开始培养时间,培养箱应置于阳光不受遮蔽处,并用流动的表层海水保持培养期间的温度恒定,培养时间一般在2 h～24 h之间,并尽量接近当地中午时间。

5.3.4.3.6 零时间样品的过滤

培养开始后,立即过滤两个零时间样品,所得载有浮游植物的滤膜,放入闪烁瓶,在通风橱中,加入1 cm^3 0.1 mol/dm^3盐酸,15 min后加盖。或在通风橱中以浓盐酸蒸气熏滤膜15 min,放入闪烁瓶。

5.3.4.3.7 过滤

样品培养后,过滤水样步骤见5.3.4.3.6。

5.3.4.3.8 放射性活度测定

向装有带浮游植物的滤膜的闪烁瓶加入10 cm^3闪烁液,在振荡器上缓慢振荡至少20 min后,把闪烁瓶置于液体闪烁计数仪内使样品暗适应12 h后测定,并记录于表H.5。

5.3.4.4 水样二氧化碳总浓度测定

测定海水样品的盐度,计算海水中二氧化碳总浓度:

$$\rho(C) = (0.067S - 0.05) \times 12\,000 \qquad \cdots\cdots(2)$$

式中:

$\rho(C)$——海水中二氧化碳的总浓度(以C计),单位为毫克每立方米(mg/m^3);

S——海水实用盐度(无量纲)。

5.4 叶绿素a和初级生产力的粒度分级测定

5.4.1 叶绿素a

5.4.1.1 方法原理

通过不同孔径的滤膜可以把不同粒径的浮游植物分别截留下来,从而达到对不同粒度的浮游植物

进行分级测量的目的。粒级划分按国际通用标准，一般分为 20 μm～200 μm(小型)、2 μm～20 μm(微型)和<2 μm(微微型)三个粒级。分级后测量方法原理同 5.2.1.1。

5.4.1.2　主要仪器设备

a)　荧光计：同 5.2.1.2 a)；

b)　抽滤装置：同 5.2.1.2 b)；

c)　滤膜：200 μm 筛绢(用以除去较大型浮游生物)；20 μm 筛绢、2 μm 核孔滤膜、0.65 μm 玻璃纤维滤膜；

d)　冰箱。

5.4.1.3　试剂

同 5.2.1.3。

5.4.1.4　测定步骤

5.4.1.4.1　荧光计校准

同 5.2.1.4.1。

5.4.1.4.2　水样测定

5.4.1.4.2.1　过滤

采样后，先经 200 μm 筛绢过滤一遍，然后将充分混合均匀的水样一式两份，一份直接用玻璃纤维滤膜过滤；另一份则依次通过 20 μm 筛绢(在无抽滤负压条件下)、2 μm 核孔滤膜和 0.65 μm 玻璃纤维滤膜三种规格滤膜过滤，分别记为 $Micro^-$(或 Net^-)、$Nano^-$ 和 $Pico^-$。

5.4.1.4.2.2　样品保存和提取

分别同 5.2.1.4.2.3 和 5.2.1.4.2.4。

5.4.1.4.2.3　荧光测定

同 5.2.1.4.2.5。

5.4.2　初级生产力

5.4.2.1　方法原理

同 5.4.1.1 和 5.3.1。

5.4.2.2　主要仪器设备

a)　水下光量子仪、水下照度计或透明度盘；

b)　样品培养箱或培养瓶罩：同 5.3.2b)；

c)　抽滤装置：同 5.3.2c)；

d)　滤膜：20 μm 筛绢、2 μm 核孔滤膜、0.65 μm 的微孔玻璃纤维滤膜；

e)　液体闪烁计数仪、通风橱和振荡器。

5.4.2.3　试剂

同 5.3.3。

5.4.2.4　测定步骤

除过滤时将每层培养的两个白瓶(平行样)中的一个直接用 0.65 μm 的微孔玻璃纤维滤膜，而另一个则依次用 20 μm 筛绢、2 μm 的核孔滤膜、0.65 μm 的微孔滤膜分级过滤外，其余步骤同 5.3.4。

5.5　海洋新生产力(^{15}N 示踪法)测定

5.5.1　方法原理

海洋真光层(即光合作用层)生态系统中的氮营养盐可根据其来源划分为内源(如 NH_4-N、Urea-N)和外源(如 NO_3-N、N_2-N)两部分，由外源氮构成的初级生产力为新生产力，内源氮构成的初级生产力为再生生产力。因此通过 ^{15}N 同位素标记外源氮和内源氮，并测定它们的吸收率即可测算新生产力和再生生产力。由于 NO_3-N 和 NH_4-N 分别是最主要的外源氮和内源氮，通常只测 NO_3-N 和 NH_4-N 的吸收率。(注意新生产力概念在近海的局限性：当有 NH_4-N 等还原态氮来自系统外部时，由该法测

得的新生产力有负误差)。

5.5.2 主要仪器设备

质谱仪、消解设备——常规消煮炉。

5.5.3 试剂

a) 示踪剂:丰度为95%～99%原子的 $K^{15}NO_3$、$^{15}NH_4Cl$ 或 $(^{15}NH_4)_2SO_4$;

b) 氧化剂:AR级浓 H_2SO_4;

c) 增温剂:AR级 K_2SO_4;

d) 催化剂:AR级 $CuSO_4$,Se;

e) 加速剂:按 K_2SO_4 : $CuSO_4$: Se=200 : 20 : 1 比例配成;

f) 扩散剂:AR级 NaOH(10 mol/dm^3);

g) 吸收剂:GR级 HCl(0.6mol/dm^3)。

5.5.4 现场实验

5.5.4.1 采样与培养

a) 采样方法、水层深度与"^{14}C法测定初级生产力"相一致。若考虑大型浮游动物的干扰,可将现场水样用200 μm筛绢过滤以除之;

b) 实验采用酸洗的聚碳酸酯(polycarbonate)瓶。酸洗可采用无金属硝酸清洗法,使用该方法应非常小心,需多次的蒸馏水冲洗,确保无滞留的 NO_3-N 污染。为避免氮污染,也可用稀盐酸来代替硝酸。

c) 取500 cm^3～1 000 cm^3 海水用于现场培养实验(在条件允许的情况下培养体积尽量大一些以减少误差)。如果采取甲板流水控温模拟现场培养,应用不同透光率的遮光物来模拟各采样深度的现场光强度。对于深层样品,特别是温跃层内或温跃层下方水样的培养,另外采用控温处理是必要的。

5.5.4.2 示踪添加剂

$^{15}NO_3$-N 和 $^{15}NH_4$-N 的添加量应不多于所测现场氮浓度的10%,对于现场氮浓度低于检测限的情况,视营养盐分析方法的检测下限添加。

5.5.4.3 培养时间

培养时间一般为4 h。实验最少一天进行两次,白天一次,夜里一次。因为浮游植物氮吸收并非完全依赖于光,且浮游细菌也能利用一部分 NH_4-N 和 NO_3-N。

5.5.4.4 样品过滤与保存

培育完毕后,水样在负压<0.03 MPa条件下过滤到经450℃～500℃下灼烧5 h～6 h的玻璃纤维滤膜上,并用过滤海水清洗滤膜,除去滤膜孔隙中滞留的溶解态^{15}N。待海水刚刚滤完时立即停止负压,小心取下滤膜立即进行干燥或于-20℃下密闭贮存。

5.5.5 同位素分析

5.5.5.1 离子质谱法

样品经以下步骤处理:

a) 消解:采用Kjeldahl法消解膜样品,将有机氮转化为 NH_4^+ 态氮;

b) NH_4^+ 的扩散吸收:将消解液定容,取适量于小型Conway皿之外槽,将盛有300 mm^3～400 mm^3的吸收剂的小表面皿置于Conway皿之中心。在消解液中加入8 cm^3～10 cm^3 扩散剂,混匀后在40℃下扩散吸收12 h;

c) ^{15}N丰度测定:经扩散吸收提纯的样品,再经低温风干浓缩至15 mm^3～25 mm^3,取1 mm^3～2 mm^3于样品靶上,低温风干后进样测定^{15}N丰度;

d) 实验水样中的$^{15}NH_4$-N丰度,可直接经扩散吸收后测定,NH_4-N浓度、颗粒有机氮(PON)浓度可由同位素稀释法同时测得。

5.5.5.2 气体质谱法

将膜样品直接经 Dumas 法燃烧，将 PON 全部转变为 N_2，测定 N_2 中 ^{15}N 同位素丰度。

5.5.5.3 填写表 H.6。

5.6 资料整理

5.6.1 海水浮游植物色素的测定

5.6.1.1 萃取荧光法（叶绿素 a）的资料整理

5.6.1.1.1 数据计算

5.6.1.1.1.1 计算海水中叶绿素 a 浓度

$$\rho_v(\text{Chl a}) = \frac{F_d \cdot (R_b - R_a) \cdot V_1}{V_2} \tag{3}$$

式中：

$\rho_v(\text{Chl a})$——海水中叶绿素 a 质量浓度，单位为毫克每立方米（mg/m^3）；

F_d——量程档“d”的换算系数，单位为毫克每立方米（mg/m^3）；

R_b——酸化前荧光值；

R_a——酸化后荧光值；

V_1——提取液的体积，单位为毫升（cm^3）；

V_2——过滤海水的体积，单位为毫升（cm^3）。

5.6.1.1.1.2 计算水柱叶绿素 a 含量

$$\rho_s(\text{Chl a}) = \sum_{i=1}^{n-1} \frac{\rho_{vi}(\text{Chl a}) + \rho_{vi+1}(\text{Chl a})}{2} \cdot (D_{i+1} - D_i) \tag{4}$$

式中：

$\rho_s(\text{Chl a})$——水柱叶绿素 a 含量，单位为毫克每平方米（mg/m^2）；

$\rho_{vi}(\text{Chl a})$——第 i 层叶绿素 a 质量浓度，单位为毫克每立方米（mg/m^3）；

D_i——第 i 层的深度，单位为米（m）；

n——取样层次数；

$1 \leqslant i \leqslant n-1$。

5.6.1.1.1.3 计算水柱叶绿素 a 平均浓度值

$$\rho_v(\text{Chl a}) = \frac{\rho_s(\text{Chl a})}{D} \tag{5}$$

式中：

$\rho_v(\text{Chl a})$——水柱叶绿素 a 平均质量浓度值，单位为毫克每立方米（mg/m^3）；

$\rho_s(\text{Chl a})$——水柱叶绿素 a 含量，单位为毫克每平方米（mg/m^2）；

D——最大取样深度，单位为米（m）。

5.6.1.1.2 填写表 H.2

5.6.1.1.3 绘制分布图

5.6.1.1.3.1 平面分布图

a) 各层次分布图

等值线取值标准（单位为 mg/m^3）：0.10，0.20，0.30，0.50，0.75，1.00，1.50，2.00，3.00，5.00，10.00；

b) 含量分布图

等值线取值标准（单位为 mg/m^2）：1.00，1.50，2.00，3.00，5.00，10.00，20.00，30.00，50.00，100.00，200.00，300.00，500.00。

5.6.1.1.3.2 断面分布图

等值线取值标准（单位为 mg/m^3）：0.10，0.20，0.30，0.50，0.75，1.00，1.50，2.00，3.00，

5.00，10.00。

上述平面和断面分布图取值标准，可视具体情况增减。

5.6.1.2 分光光度法的资料整理

5.6.1.2.1 数据计算

5.6.1.2.1.1 计算提取液中叶绿素 a、b、c 的质量浓度

$$\rho_n(\text{Chl a}) = 11.85E_{664} - 1.54E_{647} - 0.08E_{630} \quad\cdots\cdots(6)$$

$$\rho_n(\text{Chl b}) = 21.03E_{647} - 5.43E_{664} - 2.66E_{630} \quad\cdots\cdots(7)$$

$$\rho_n(\text{Chl c}) = 24.52E_{630} - 1.67E_{664} - 7.60E_{647} \quad\cdots\cdots(8)$$

式中：

$\rho_n(\text{Chl a})$——提取液中叶绿素 a 的质量浓度，单位为微克每立方厘米（$\mu g/cm^3$）；

$\rho_n(\text{Chl b})$——提取液中叶绿素 b 的质量浓度，单位为微克每立方厘米（$\mu g/cm^3$）；

$\rho_n(\text{Chl c})$——提取液中叶绿素 c 的质量浓度，单位为微克每立方厘米（$\mu g/cm^3$）；

E_{664}——波长为 664 nm 处 1 cm 光程经浊度校正的消光值；

E_{647}——波长为 647 nm 处 1 cm 光程经浊度校正的消光值；

E_{630}——波长为 630 nm 处 1 cm 光程经浊度校正的消光值。

5.6.1.2.1.2 计算海水中叶绿素 a、b、c 的浓度

$$\rho(\text{Chl a}) = \frac{\rho_n(\text{Chl a}) \cdot V_1}{V_2} \quad\cdots\cdots(9)$$

$$\rho(\text{Chl b}) = \frac{\rho_n(\text{Chl b}) \cdot V_1}{V_2} \quad\cdots\cdots(10)$$

$$\rho(\text{Chl c}) = \frac{\rho_n(\text{Chl c}) \cdot V_1}{V_2} \quad\cdots\cdots(11)$$

式中：

$\rho(\text{Chl a})$——海水中叶绿素 a 的质量浓度，单位为毫克每立方米（mg/m^3）；

$\rho(\text{Chl b})$——海水中叶绿素 b 的质量浓度，单位为毫克每立方米（mg/m^3）；

$\rho(\text{Chl c})$——海水中叶绿素 c 的质量浓度，单位为毫克每立方米（mg/m^3）；

$\rho_n(\text{Chl a})$——提取液中叶绿素 a 的质量浓度，单位为微克每立方厘米（$\mu g/cm^3$）；

$\rho_n(\text{Chl b})$——提取液中叶绿素 b 的质量浓度，单位为微克每立方厘米（$\mu g/cm^3$）；

$\rho_n(\text{Chl c})$——提取液中叶绿素 c 的质量浓度，单位为微克每立方厘米（$\mu g/cm^3$）；

V_1——提取液的体积，单位为毫升（cm^3）；

V_2——过滤海水的体积，单位为升（dm^3）。

5.6.1.2.1.3 计算海水中叶绿素总质量浓度

$$\rho(\text{Chl}) = \rho(\text{Chl a}) + \rho(\text{Chl b}) + \rho(\text{Chl c}) \quad\cdots\cdots(12)$$

式中：

$\rho(\text{Chl})$——海水中叶绿素总质量浓度，单位为毫克每立方米（mg/m^3）；

$\rho(\text{Chl a})$——海水中叶绿素 a 的质量浓度，单位为毫克每立方米（mg/m^3）；

$\rho(\text{Chl b})$——海水中叶绿素 b 的质量浓度，单位为毫克每立方米（mg/m^3）；

$\rho(\text{Chl c})$——海水中叶绿素 c 的质量浓度，单位为毫克每立方米（mg/m^3）。

5.6.1.2.2 计算水柱叶绿素 a 含量

见 5.6.1.1.1.2。

5.6.1.2.3 计算水柱叶绿素 a 平均质量浓度

见 5.6.1.1.1.3。

5.6.1.2.4 填写表 H.3

5.6.1.2.5 绘制分布图

见 5.6.1.1.3。

5.6.1.3 高效液相色谱法的资料整理

5.6.1.3.1 色谱图检验

在获取色谱图后应立即进行检验，如果发现问题，应重新进样测定。一般情况下计算机软件会自动对每一个峰进行积分。但应对基线，峰的开始点和结束点进行核对。

5.6.1.3.2 色谱峰鉴别

每一个峰所代表的色素种类通常根据保留时间或色谱图来确定，如果在测试样品前测定了色素组成已知的培养藻类的样品或根据标准色素鉴别工作就较简单。

5.6.1.3.3 色素含量计算

色素含量是根据进样量和峰面积积分来计算的。

a) 计算色素响应系数

对每种色素，做出其吸收峰面积对进样色素质量的关系曲线，该色素的 HPLC 响应系数 F(area/10^{-6} g)通过色素峰面积与标准液进样量(μg)的回归斜率计算。

$$F = \frac{A}{M} \qquad (13)$$

式中：

F——色素响应系数；

A——色素的峰面积；

M——注射色素标准的质量(色素质量浓度与进样体积的乘积)。

b) 计算色素含量

$$C = \frac{A \cdot V_{\mathrm{ext}} \cdot A_{\mathrm{Blk}}^{\mathrm{Ca}}}{F \cdot V_{\mathrm{inj}} \cdot V_{\mathrm{flt}} \cdot A_{\mathrm{Smp}}^{\mathrm{Ca}}} \qquad (14)$$

式中：

C——色素含量，单位为微克每升(μg/L)；

A——样品色素峰面积；

V_{ext}——提取体积，单位为毫升(mL)；

V_{inj}——注射体积，单位为毫升(mL)；

V_{flt}——样品过滤体积，单位为升(L)；

$A_{\mathrm{Blk}}^{\mathrm{Ca}}$——丙酮中内标准物的峰面积；

$A_{\mathrm{Smp}}^{\mathrm{Ca}}$——样品中内标准物的峰面积。

5.6.1.3.4 填写表 H.4

5.6.2 初级生产力的资料整理

5.6.2.1 数据计算

5.6.2.1.1 计算加入^{14}C 的量

$$R = \frac{R_{\mathrm{t}} \cdot V_1 \times 1\,000}{V_2} \qquad (15)$$

式中：

R——加入^{14}C 的量，单位为千贝可(kBq)；

R_{t}——各总计数闪烁瓶测得^{14}C 数量的平均值，单位为千贝可(kBq)；

V_1——培养水样的体积，单位为毫升(mL)；

V_2——取测总计数水样的体积，单位为毫升(mL)。

5.6.2.1.2 **计算海洋初级生产力**

$$P_v = \frac{(R_s - R_b) \cdot \rho(C)}{R \cdot T} \quad \cdots\cdots (16)$$

式中：

P_v——海洋初级生产力(以C计)，单位为毫克每立方米小时[mg/(m³·h)]；

R——加入^{14}C的量，单位为千贝可(kBq)；

R_s——白瓶样品中^{14}C的放射性活度的平均值，单位为千贝可(kBq)；

R_b——零时间样品中^{14}C的放射性活度，单位为千贝可(kBq)；

$\rho(C)$——海水中二氧化碳的总浓度，单位为毫克每立方米(mg/m³)；

T——培养时间，单位为小时(h)。

5.6.2.1.3 **计算碳同化数**

$$I = \frac{P_v}{\rho_v(\mathrm{Chl\ a})} \quad \cdots\cdots (17)$$

式中：

I——碳同化数，单位为每小时(h^{-1})；

P_v——水体初级生产力，单位为毫克每立方米小时[mg/(m³·h)]；

$\rho_v(\mathrm{Chl\ a})$——水体中叶绿素a的浓度，单位为毫克每立方米(mg/m³)。

5.6.2.1.4 **计算水柱初级生产力**

$$P_s = \sum_{i=1}^{n-1} \frac{P_{vi} + P_{vi+1}}{2} \cdot (D_{i+1} - D_i) \quad \cdots\cdots (18)$$

式中：

P_s——水柱初级生产力，单位为毫克每平方米小时[mg/(m²·h)]；

P_{vi}——第i层初级生产力，单位为毫克每立方米小时[mg/(m³·h)]；

n——采样层次数；

D_i——第i层深度，单位为米(m)；

$1 \leqslant i \leqslant n-1$。

5.6.2.2 **填写表H.5**

5.6.2.3 **绘制分布图**

5.6.2.3.1 **平面分布图**

等值线取值标准[单位为 mg/(m²·h)]：1.0，5.0，10.0，20.0，30.0，50.0，100.0，150.0，200.0。

5.6.2.3.2 **断面分布图**

等值线取值标准[单位为 mg/(m³·h)]：0.1，0.3，0.5，1.0，3.0，5.0，10.0，30.0，50.0，100.0。

上述平面和断面分布图等值线取值标准，可视具体情况增减。

5.6.3 **叶绿素a和初级生产力的粒度分级测定的资料整理**

5.6.3.1 **叶绿素a的粒度分级测定的资料整理**

a) 分别计算出不同粒级(Micro⁻、Nano⁻和Pico⁻)浮游植物叶绿素a的含量和直接用玻璃纤维滤膜过滤的浮游植物叶绿素a的总量计算公式同5.6.1.1.1.1。

b) 水体中叶绿素a含量应为三个粒级叶绿素a之和：即Chl a=Micro⁻+Nano⁻+Pico⁻，这一计算结果与直接用玻璃纤维滤膜抽滤的结果应十分接近，若二者相差较大，水体中叶绿素a的含量则取二者的平均值。

c) 各不同粒级叶绿素a所占百分比的计算可根据各粒级叶绿素a的含量与各粒级叶绿素a之和

的比,即为该粒级叶绿素 a 与叶绿素 a 总量的百分比。

5.6.3.2 初级生产力粒度分级测定的资料整理

5.6.3.2.1 不同光学深度上初级生产力总值的获取

a) 三种粒级浮游植物的初级生产力之和;

b) 直接用 0.65 μm 滤膜抽滤的测量结果;

a)和 b)所获得量值应该十分接近,若二者差异较大,初级生产力的总值取二者的平均值。

5.6.3.2.2 不同粒级浮游植物的初级生产力占总生产力的百分比

每一种滤膜上的初级生产力的测定值与三种滤膜的测定值之和的比值。即为该粒级浮游植物初级生产力与总生产力的比值。

5.6.4 新生产力的资料整理

5.6.4.1 氮的比吸收率(specific uptake rate)计算

$$V = \frac{(a_p - a_o)}{(a_d - a_o)/t} \qquad \cdots\cdots(19)$$

式中:

V——比吸收率,单位为每小时(h^{-1});

a_o——^{15}N 的天然丰度 0.366 %原子;

a_d——介质(培养液)^{15}N 丰度;

a_p——PON 的^{15}N 丰度;

t——培育时间,单位为小时(h)。

5.6.4.2 氮的吸收率或转运率(transport rate)计算

$$\rho = V \cdot PON \qquad \cdots\cdots(20)$$

式中:

ρ——吸收率或转运率,单位为微摩尔每升小时或纳摩尔每升小时[μmol/(L·h)或 nmol/(L·h)];

V——周日氮吸收速率,单位为微摩尔每升小时或纳摩尔每升小时[μmol/(L·h)或 nmol/(L·h)],约等于白天的氮吸收速率与时间的积加上夜里的速率与时间的积;

PON 以及其他 N(NH_4-N、NO_3-N 等)的浓度单位根据测定精度可为 μmol/(L·h)或 nmol/(L·h)。

5.6.4.3 新生产力的计算

分别测得新生氮源(NO_3^--N)和再生氮源(NH_4^+-N)的比吸收率 $V_{新生}$ 和 $V_{再生}$,$V_{新生}$ 与($V_{新生}+V_{再生}$)之比即为 f 比,f 比与初级生产力之积即为新生产力,新生产力与初级生产力的单位相同。

5.6.4.4 填写表 H.6

5.6.5 填写报表

按本部分的有关规定填写报表。

6 微生物调查

6.1 技术要求和调查要素

6.1.1 技术要求

6.1.1.1 采样站位和层次

a) 站位布设应尽量和其他调查项目一致;

b) 采样水层见 4.2.4.1 表 1,但可去除 5 m 水层;大洋调查可增加 500 m、1 000 m、2 000 m……等水层的采样;

c) 海洋沉积物中微生物取样层次,大面调查取表层;断面调查时,将岩芯管以 3cm 间隔分层;对

于特殊调查项目，可根据实际需要确定采样层次。

6.1.1.2 无菌操作

a) 采水器上的采样瓶、袋应预先灭菌；

b) 实验室内水、泥样分样和分离培养鉴定过程中，均按无菌操作要求进行；

c) 其他凡属海上调查所需物品，如水样贮存瓶（棕色）、移液管（1 cm^3、5 cm^3、10 cm^3）、培养皿等均需洗净，包封灭菌、足量备用。

6.1.1.3 样品保存

样品应在采样后 2 h 内处理、分析。若暂放冰箱保存，不得超过 24 h。

6.1.2 调查要素

海洋微生物调查要素为：海洋微生物现存量，即病毒、细菌总数与微生物其他类群（放线菌、酵母、霉菌等）的丰度和海洋微生物的活性，即细菌生产力、微生物异养活性、生态呼吸率的测定。

6.2 采样

6.2.1 主要仪器设备

6.2.1.1 采水器

根据采样深度，选用尼斯金采水器或击开式采水器。

6.2.1.2 采泥器

箱式采样器、多管采样器或弹簧采泥器，见小型底栖生物调查的采样器（见 11.2.1.1）。

6.2.2 采水样

a) 见 4.4.1.2，并按实际情况选用采水器；

b) 采水器开启后，应停留片刻，待水样装满；

c) 按测定项目计算采水量，若同一水层分几次采样，样品应混匀，再按各测定项目要求分装、处理。

6.2.3 采泥样

用无菌工具从预定层次中取 10 g～20 g 样品，置于无菌容器。

6.3 样品分析

6.3.1 主要仪器设备

6.3.1.1 荧光显微镜

具备蓝光道和紫外光道供观察吖啶橙和 DAPI 染色，配置照相机或高分辨率并适用于弱光场的数码相机。

6.3.1.2 液体闪烁计数仪

具有测定^{14}C和^{3}H的功能。

6.3.1.3 恒温培养箱

控温范围：5℃～50℃。

6.3.1.4 抽滤装置

进口成品滤器组或自行装配 25 mm 和 47 mm 滤器和负压抽滤设备。放射性同位素示踪测试的抽滤设备必须专用，不能与微生物计数混用。

6.3.1.5 移液枪

置备不同功用的移液枪。

6.3.1.6 微生物自动鉴定仪

现有的微生物鉴定仪器主要应用于医学微生物鉴定，有条件的单位选用较适用于环境微生物鉴定的仪器产品。

6.3.1.7 消耗性材料

6.3.1.7.1 滤膜

a) 微孔滤膜(材料:醋酸纤维或硝化纤维):直径 25 mm 和 47 mm,孔径 0.2 μm、0.45 μm 和 0.8 μm;

b) 黑色核孔滤膜(材料:聚碳酸酯):直径 25 mm,孔径 0.2 μm;

c) 氧化铝滤膜:直径 25 mm, 孔径 0.02 μm。

6.3.1.7.2 注射器和滤器

一次性无菌注射器和 0.2 μm 无菌滤器。

6.3.1.7.3 荧光显微镜计数用的装样瓶

a) 50 cm^3 螺盖聚丙烯瓶:用前用 5%HCl 溶液浸泡 1 d 以上,并经 0.02 μm 滤膜过滤的蒸馏水漱洗并高压灭菌;

b) 50 cm^3 螺盖塑料瓶:用前用 5%HCl 溶液浸泡 1 d 以上,并经 0.2 μm 滤膜过滤的蒸馏水漱洗并高压灭菌。

6.3.1.7.4 同位素示踪培养瓶和闪烁计数瓶

a) 50 cm^3 螺盖培养管;

b) 125 cm^3 螺盖培养瓶;

c) 20 cm^3 或 7 cm^3 与闪烁仪匹配的玻璃或塑料闪烁瓶。

6.3.1.7.5 玻璃器皿

培养皿、BOD 培养瓶和常用玻璃器皿。

6.3.2 培养基、试剂和工作溶液

6.3.2.1 培养计数用的培养基、试剂

6.3.2.1.1 陈海水

取天然海水避光保存 3 个月以上,使用时取其清液或经 0.45 μm 滤膜过滤的滤液。

6.3.2.1.2 表面活性剂

将吐温-80(Tween-80)按体积分数为 1:2 000 比例配成水溶液(工作液)高压灭菌备用。

6.3.2.1.3 细菌培养基

进口 Marine 2216 琼脂成品培养基或自制 2216 E 培养基。优先选择进口成品培养基。

a) 进口 Marine 2216 琼脂成品培养基

——成分:见培养基瓶上标示;

——制备:照培养基瓶上标示进行。

b) 自制 2216 E 培养基

——成分:

蛋白胨	5.0 g
酵母膏	1.0 g
$FePO_4$	0.01 g
琼脂	20.0 g
陈海水	1 000 cm^3
pH	7.4~7.8

——制备:将各成分加热溶化,趁热分装,经 121℃(98.1 kPa)高压灭菌 20 min 后,制成平板。

6.3.2.1.4 放线菌培养基:高氏 1 号合成培养基

a) 成分:

可溶性淀粉	20.0 g
K_2HPO_4	0.5 g

KNO_3	1.0 g
$MgSO_4 \cdot 7H_2O$	0.5 g
$FeSO_4$	0.01 g
5%$K_2Cr_2O_7$	1.0 cm^3
琼脂	20.0 g
陈海水	1 000 cm^3
pH	7.2～7.4

b) 制备：称 5.0g $K_2Cr_2O_7$ 溶于 100 cm^3 蒸馏水中，装瓶；将其余各成分加热溶化，趁热定量分装。两者经 121℃(98.1 kPa)高压灭菌 20 min 按比例加入重铬酸钾溶液，摇匀，制成平板。

6.3.2.1.5 真菌培养基

庆大霉素马铃薯葡萄糖琼脂(PDA)(选国产成品或自制)。优先选择国产成品培养基。

a) 国产成品 PDA 庆大霉素培养基

——成分：

按培养基瓶上标示称重

庆大霉素	50.0 mg
陈海水	1 000 cm^3
pH	自然

——制备：将成品培养基加热溶化，趁热定量分装。经 121℃(98.1 kPa)高压灭菌 20 min，按比例加入庆大霉素，制成平板。

b) 自制 PDA 庆大霉素培养基

——成分：

马铃薯(去皮切块)	200 g
葡萄糖	20.0 g
庆大霉素	50.0 mg
琼脂	20.0 g
陈海水	1 000 cm^3
pH	自然

——制备：将去皮切块的马铃薯放入 1 000 cm^3 陈海水煮沸 30 min，用纱布过滤，补加海水至 1 000 cm^3，加入葡萄糖和琼脂溶化定量分装，121℃(98.1 kPa)高压灭菌 20 min，按比例加入庆大霉素混匀，制成平板。

6.3.2.2 荧光显微镜直接计数用的试剂和工作溶液

6.3.2.2.1 甲醛溶液

a) 经 0.02 μm 滤膜过滤的体积分数为 37%～40%甲醛溶液。

b) 经 0.2 μm 滤膜过滤的体积分数为 37%～40%甲醛溶液。

6.3.2.2.2 病毒直接计数溶液

a) SYBR Green I(DNA 特异性荧光染色剂)染色剂储备液市售，−20℃暗保存。

b) 荧光保护剂

——PBS(Phosphate Buffered Saline)甘油储备液

将 50%PBS(0.05 mol/L Na_2HPO_4，质量分数为 0.85%NaCl，pH7.5)和体积分数为 50%甘油混合并置冰箱保存。

——10% p-苯二胺(p-Phenylenediamine)储备液

市售，冷冻(结冰)暗保存。

6.3.2.2.3 质量分数为0.1%的吖啶橙(acridine orange)工作溶液

称0.2 g吖啶橙,溶于200 cm^3 经0.2 μm过滤的蒸馏水中,再用0.2 μm无菌滤器把吖啶橙溶液滤于棕色瓶中,并加入12 cm^3 经0.2 μm滤膜过滤的甲醛溶液,该溶液置冰箱保存期可达数月。

6.3.2.2.4 DAPI(4',6-diamidino-2-phenylindole,即4',6-二脒基-2-苯基吲哚)储备液和工作溶液

取10 mg DAPI溶于50 cm^3 蒸馏水中(200 μg/cm^3),用0.2 μm一次性滤器过滤,按1 cm^3～2 cm^3 量分装于1.5 cm^3～2 cm^3 小离心管中,在－20℃冷冻保存、备用,保存期可达一年。临用前取一小管储备液解冻后稀释成10 μg/cm^3,并用0.2 μm一次性滤器过滤,配制成工作溶液。

6.3.2.3 同位素示踪用的试剂和工作溶液。

6.3.2.3.1 [甲基-^{3}H]胸腺嘧啶核苷作溶液([methyl-^{3}H]-thymidine)储备液。

取市售放射性比活度高于1.85×10^{12} Bq/mmol的[甲基-^{3}H]胸腺嘧啶核苷储备液,使用前用经高压灭菌的蒸馏水稀释成5 nmol/cm^3 的工作液。

6.3.2.3.2 ^{3}H-亮氨酸(^{3}H-Leucine)工作溶液

取市售放射性比活≥2.22×10^{12} Bq/mmol的[4,5-^{3}H]亮氨酸,使用前用经高压灭菌的蒸馏水稀释成20 nmol/cm^3 亮氨酸工作液。

6.3.2.3.3 D-[UL-^{14}C]葡萄糖(D-[UL-^{14}C]glucose)工作液

取市售的放射性比活高于7.4×10^{10} Bq/mol的D-[UL-^{14}C]葡萄糖储备液,用氯化钠溶液p(NaCl)＝35 g/dm^3 稀释成1.85×10^5 Bq/cm^3 的工作液,并按10 μg/cm^3 量加入非放射性葡萄糖,然后定量分装于安瓿瓶中,封熔后高压(68.95 kPa)灭菌15 min备用。

6.3.2.3.4 三氯乙酸(TCA)淋洗液

配制质量分数为5%的三氯乙酸溶液。

6.3.2.3.5 80%乙醇溶液

用蒸馏水将无水乙醇稀释成体积分数为80%溶液。

6.3.2.3.6 闪烁液

市售成品闪烁液。

6.3.3 样品处理

6.3.3.1 水样处理

6.3.3.1.1 外荧光直接计数的水样

a) 病毒计数:取水样50 cm^3 于预先处理的采样瓶(见6.3.1.7.3a))中,加甲醛溶液(见6.3.2.2.1a))2.5 cm^3(甲醛在样品中的质量浓度为2%)固定样品,样品应立即分析,否则应在4℃避光保存,但只能保存一周。
b) 细菌计数:取水样50 cm^3 于预先处理的采样瓶(见6.3.1.7.3b))中,加甲醛溶液(见6.3.2.2.1b))2.5 cm^3(甲醛在样品中的质量浓度为2%)固定样品,样品于2℃～8℃低温保存。

6.3.3.1.2 滤膜萌发、平板计数的水样

按10 cm^3/dm^3 量加灭菌的吐温－80工作溶液。

6.3.3.1.3 细菌生产力水样

a) [甲基-^{3}H]胸腺嘧啶核苷示踪水样

取3支50 cm^3 带螺盖培养管,各加入20 cm^3 水样,其中一管加1 cm^3 甲醛混匀为对照,再向各管加入[甲基-^{3}H]胸腺嘧啶核苷工作溶液,使其终浓度为250 nmol/dm^3(即加入1 cm^3 该示踪剂的工作溶液),混匀,置现场温度培养1 h,再加入1 cm^3 甲醛于测样瓶中摇均,以终止培养并保存于冰箱。对活性较低的水体,应适当延长培养时间。

b) [^{3}H]亮氨酸示踪水样

用[4,5-^{3}H]亮氨酸代替[甲基-^{3}H]胸腺嘧啶核苷,使其在样品中的最终浓度为20 nmol/dm^3(即加20 mm^3 示踪剂于20 cm^3 样品中),其余步骤同上。

6.3.3.1.4 微生物异养活性测定的水样

取3个125 cm^3 带螺盖三角瓶，各加入100 cm^3 水样，其中1瓶加入5 cm^3 甲醛溶液作对照，再向各瓶加入D-[UL-^{14}C]葡萄糖工作溶液1 cm^3，盖紧混匀，置现场水温暗培养1 h后，加入5 cm^3 甲醛于测样中，摇匀以终止培养。

6.3.3.1.5 生态呼吸率测定水样

将水样按溶解氧测定要求注入4个250 cm^3BOD培养瓶中，其中2瓶立即固定，另2瓶置暗处于现场水温条件下培养1 d后，取出固定。对寡营养水体的样品应适当延长培养时间。

6.3.3.2 泥样处理

6.3.3.2.1 微生物计数泥样

取约2 g泥样，精确称重后，置于装有含吐温－80(10 cm^3/dm^3)的18 cm^3 海水并加玻璃珠的无菌三角瓶中，充分摇荡，制成悬浮液。

6.3.3.2.2 测定干重泥样

保存余下的泥样供测干重。

6.3.3.3 记录

将以上结果分别记录于表H.7、表H.10、表H.11和表H.12。

6.3.4 样品分析

6.3.4.1 水样微生物计数

微生物计数包括针对总菌数的"直接计数"和针对可培养微生物的"培养计数"。"直接计数"采用落射荧光显微镜，有条件的应采用流式细胞仪。

6.3.4.1.1 荧光显微镜直接计数

6.3.4.1.1.1 **SYBR Green I 直接计数法**

适用于测定病毒总数。也可同时测定细菌总数，但在病毒颗粒最佳分布的视野中，细菌数偏少，且制片操作步骤繁琐、氧化铝滤膜价格目前是核孔滤膜的4倍。其工作程序如下：

a) 滤器装配：长期未用的滤器应先装好滤器和抽滤装置，用经0.02 μm过滤的高压灭菌蒸馏水加满滤筒并抽滤清洗2次或3次，(当天使用的滤器，在各个样品测定前用同样方法清洗一次，清洗时不必取下衬垫滤膜)，再卸下滤筒在滤板上先放置孔径为0.45 μm或0.8 μm的25 mm微孔滤膜作衬底(衬底可多次使用)；然后将孔径为0.02 μm氧化铝滤膜放于衬垫上，再装配好滤筒；

b) 配制染色剂：临用前，取出SYBR Green I储备液(6.3.2.2.2 a))，用经0.02 μm过滤的无菌去离子水按1∶10稀释(如加5 mm^3 储备液到45 mm^3 稀释水中)，操作必须在弱光环境中进行，未用完的储备液应立即放回－20℃冰箱避光保存；

c) 配制荧光保护剂工作液：临用前取10% p-苯二胺储备液(6.3.2.2.2 b))、化霜、摇匀后，吸取10 mm^3 与990 mm^3PBS甘油储备液(6.3.2.2.2 b))混合，制成工作液，该工作液应避光、置冰浴，未用完的10% p-苯二胺储备液应立即再行冷冻，但该储备液管累计冻、融3次后，便须丢弃；如果p-苯二胺储备液或荧光保护剂工作液呈棕色，弃之不用并重新配制；

d) 加样：加入适量样品(1 cm^3～10 cm^3，即视野病毒数控制在50个左右)，若样品量少于1 cm^3 则应先加入2 cm^3 经0.02 μm过滤的无菌海水，再加样，以便病毒、细菌均匀分布于滤膜上；

e) 抽滤：在负压(15 kPa～20 kPa)条件下，把样品抽滤至干，卸下滤筒；

f) 染色：吸取97.5 mm^3 经0.02 μm过滤的无菌去离子水，滴入干净的无菌塑料培养皿，再向该水滴加入2.5 μL 10% SYBR Green I染色剂工作液(此时，染色剂浓度为0.25%)，培养皿及染色剂需置于冰浴、避光处；用扁平头镊子在负压状态下，取下载有样品的氧化铝滤膜，将膜的背面(非载样面)放在培养皿的染色液滴上冰浴、避光染色15 min；

g) 制片：染色后，取出滤膜，吸干贴上滤膜背面及边缘的液滴，把滤膜紧贴于载玻片上(载样面朝

上),并在盖玻片上加 30 mm^3 荧光保护剂工作液,具液面朝下盖紧滤膜(滤膜上下两面均不能有气泡),用指甲油密封盖玻片四周,制片在-20℃条件下可保存 2 周~3 周,但以立即计数为宜;

h) 计数:在荧光显微镜蓝色激发光道、油镜条件下,病毒颗粒呈针扎(pinprick)状、亮绿色,细菌细胞亦呈亮绿色,但其亮度强于病毒颗粒,且菌体比病毒颗粒大得多。随机取 10 个~20 个视野,计算病毒颗粒数(如果要同时计算细菌数的话,还需计算具有细菌形态的细胞数),每个样品至少计数 200 个病毒(或细菌);

i) 每次(不同测定日期)测定时,应加测不加样品的空白对照,对于空白制片,每个视野不得出现 1 个以上病毒(或细菌)。

j) 计算样品含病毒颗粒数

$$VN = \frac{N_a \cdot S}{S_f \cdot (1-0.05) \cdot V} \qquad \cdots\cdots(21)$$

式中:

VN——样品含病毒数,单位为个每升(particles/L);

N_a——各视野平均病毒数,单位为个(particles);

S——滤膜实际过滤面积,单位为平方毫米(mm^2);

S_f——显微镜视野面积,单位为平方毫米(mm^2);

V——过滤样品量(式中 0.05 为加入 37%~40% 甲醛占固定样品总体积的比例),单位为升(L)。

k) 将分析结果记录于表 H.8。

6.3.4.1.1.2 吖啶橙直接计数法

适用于测定细菌总数,工作程序如下:

a) 滤器装配:长期未用的滤器应先装好滤器和抽滤装置,用经 0.2 μm 过滤的高压灭菌蒸馏水加满滤筒并抽滤清洗 2 次或 3 次,(当天使用的滤器,在各个样品测定前用同样方法清洗一次,清洗时不必取下衬垫滤膜),再卸下滤筒在滤板上先放置孔径为 0.8 μm 或 0.45 μm 的 25 mm 微孔滤膜作衬底(衬底可多次使用);然后将黑色核孔滤膜(光滑面朝上)放于衬垫上,再装配好滤筒;

b) 加样:加入适量样品(控制在视野出现菌体 50 个左右),若样品量少于 1 cm^3 则应先加入 2 cm^3 经 0.2 μm 过滤的无菌海水,再加样,以便细菌均匀分布于滤膜上;

c) 抽滤:在负压(50 kPa)条件下,把样品抽滤至滤膜刚好呈湿润状态,并释放真空;

d) 染色:用无菌注射器吸取吖啶橙工作液,套上 0.2 μm 无菌滤器,沿滤筒壁加入约 1 cm^3 染色液,使其盖满滤膜并染色 5 min~10 min。而后在同样负压下抽滤至干;

e) 制片:在载玻片上加一小滴无荧光镜油,贴上滤膜(载菌一面朝上),并在盖玻片上加一小滴同样镜油,具油面朝下盖紧滤膜(滤膜上下两面均不能有气泡),用指甲油密封盖玻片四周,制片在-20℃条件下可保存数月,但以立即计数为宜;

f) 计数:在荧光显微镜蓝光道,油镜条件下,随机取 10 个视野,计算具有细菌形态呈亮绿色的细胞数;每个样品至少计数 300 个菌体;每次(不同测定日期)测定时,应加测不加样品的空白对照,空白制片,每个视野不得出现 1 个以上细菌;

g) 计算样品含菌数

$$BN = \frac{N_a \cdot S}{S_f \cdot (1-0.05) \cdot V} \qquad \cdots\cdots(22)$$

式中:

BN——样品含菌数,单位为个每升(cells/L);

N_a——各视野平均菌数，单位为个(cells)；

S——滤膜实际过滤面积，单位为平方毫米(mm^2)；

S_f——显微镜视野面积，单位为平方毫米(mm^2)；

V——过滤样品量(式中 0.05 为加入 37～40% 甲醛占固定样品总体积的比例)，单位为升(L)。

h) 将分析结果记录于表 H.8。

6.3.4.1.1.3 **DAPI 直接计数法**

适用于测定细菌总数，和吖啶橙染色法相比，背景更清晰，荧光色素干扰较少，菌体着色不易衰减但需要用紫外光激发滤光系统(即：G 365 激发滤光片，FT 395 分射滤光片，LP 420 吸收滤光片)的显微镜观察。其工作程序除了用 DAPI 工作溶液(即：10 $\mu g/cm^3$)代替吖啶橙染色液外，其余步骤均相同。当本计数法与吖啶直接计数法的计数结果有异议时，以本计数法为仲裁计数法。

6.3.4.1.1.4 **细菌体积测定——显微摄影、幻灯测量法**

细菌生物量(以 C 计)与细菌个体大小相关。通过测定细菌体积并借助于经验转换系数，把细菌转化为细菌生物量。已有许多方法应用于测量细菌体积，数码摄像并带影像自动分析软件系统属较先进的技术，需要大资金的设备投入，而显微摄影——幻灯测量法系普及型技术，具体工作程序如下：

a) 胶卷安装和系统测试：选用 ASA 400 彩色反转片胶卷，按显微摄影要求操作，并通过测试比较，选择适当的曝光组合；

b) 样品拍照：在荧光计数时，选择 5 个～10 个菌数较多的视野，进行拍照，以便在照片上获得足够多的菌体。同时记录照片序号和相对应的样品号；

c) 标尺拍照：在与细菌直接计数相同放大倍数条件下，利用显微镜普通光系统，清晰地拍照台围尺标度；

d) 制备幻灯投影：冲洗拍照的胶卷，制成幻灯片，装入幻灯机，投影于贴白纸的墙壁上；

e) 菌体测量：用游标卡尺测量幻灯投影台微尺的长度，注意测量不同部位的长度，以便获得统计平均值。选择轮廓清晰的菌体，测量其长度和宽度，并作记录。每测完一个菌体，应立即作标记，以防重复测量；

f) 细菌体积计算：先根据台微尺刻度实际长度和幻灯投影上测量长度的放大比例，计算出被测细菌实际长度和宽度，然后按下述公式，计算细菌体积；

$$V = \frac{\pi}{4} \cdot W^2 \cdot (L - \frac{W}{3}) \qquad \cdots\cdots (23)$$

式中：

V——细菌体积，单位为立方微米(μm^3)；

L——细菌长度，单位为微米(μm)；

W——细菌宽度(直径)，单位为微米(μm)。

g) 如果荧光显微镜配置高分辨率、适合于弱光场摄影的数码相机，则可不用胶卷，完成本项测定；

h) 将测定结果记录于表 H.9，然后把数据输入计算机，计算细菌体积。

6.3.4.1.1.5 **细菌生物量(以 C 计)计算**

根据下述经验公式，把样品的细菌细胞数，通过细胞平均体积，换算为样品的细菌生物量。

$$BB = 8.99 \cdot 10^{-8} \cdot V_m^{0.59} \cdot BN \qquad \cdots\cdots (24)$$

式中：

BB——样品的细菌生物量，单位为微克每升($\mu g/L$)；

V_m——菌细胞平均体积，单位为立方微米每个($\mu m^3/cell$)；

BN——直接计数的细菌数量，单位为个每升(cells/L)。

6.3.4.1.2 **培养计数法**

6.3.4.1.2.1 **滤膜萌发计数法**

适用于测定微生物活菌数，多用于水深大于 200 m 的海区。工作程序如下：

a) 滤膜处理:使用前,微孔滤膜(孔径 0.2 μm,直径 47 mm)用铝箔纸包好,高压灭菌;
b) 滤器装配:见 6.3.4.1.1.1 a),但不用加衬垫;
c) 加样:加适量样品(以每片滤膜出现 30 个～50 个左右的菌落为宜),若加样量少,应添加适量高压灭菌海水稀释;用于放线菌、酵母、霉菌计数的样品,加样量应略大;每个样品一般取两种过滤量,每种过滤量重复三片滤膜;
d) 抽滤:见 6.3.4.1.1.1 e);
e) 培养:将滤膜粗糙面(即:没有载滤物的一面)紧贴于备好的平板培养基上(滤膜和培养基间不得有气泡),将平板倒置于接近样品现场温度的恒温箱培养 4 d～15 d;
f) 计数:在放大镜下或用菌落计数器,按菌落形态,分别计算各种培养基中四大菌类的菌落数(必要时,用显微镜观察确证);
g) 计算样品含菌数

$$N=\frac{N_a}{(1-0.01)\cdot V} \qquad \cdots\cdots(25)$$

式中:

N——样品含菌落数,单位为个每升(CFU/L);

N_a——三片滤膜上的平均菌落数,单位为个(CFU);

V——过滤样品量(式中 0.01 为加入吐温-80 占样品体积的比例),单位为升(L)。

h) 将分析结果记录于表 H.10。

6.3.4.1.2.2 平板计数法

适用于测定水深小于 200 m 海区的微生物活菌数。工作程序如下:

a) 稀释:用高压灭菌海水制成梯度稀释液;
b) 接种:根据不同计数对象,取适当稀释度样品 0.1 cm^3(以平板上出现 30 个～300 个菌落为宜),接种于相应的平板培养基上,并涂布均匀;一般每个样品取两个稀释度,每个稀释度重复三个平板;
c) 培养:将平板倒置于接近现场温度恒温箱,培养 4 d～15 d;
d) 计数:见 6.3.4.1.2.1 f);
e) 计算样品含菌数

$$N=\frac{N_a\cdot D}{(1-0.01)\cdot V} \qquad \cdots\cdots(26)$$

式中:

N——样品含菌落数,单位为个每升(CFU/L);

N_a——三个平板平均菌落数,单位为个(CFU);

D——样品稀释倍数;

V——接种量,单位为升(L)。

f) 将分析结果记录于表 H.10。

6.3.4.1.2.3 最可能数(MPN)计数法

见附录 A。

6.3.4.2 水样微生物活性测定

6.3.4.2.1 细菌生产力测定

6.3.4.2.1.1 [甲基-^3H]胸腺嘧啶核苷酸示踪法

a) 采用孔径 0.2 μm 直径 25 mm 微孔滤膜,并装配好滤器和抽滤装置;
b) 抽滤:从冰箱拿出样品,倒入滤器,负压(30 kPa)条件下抽滤,并用 2 cm^3 冰浴的质量浓度为 5% 三氯乙酸漱洗培养瓶加入滤器,抽滤至干;

c) 淋洗：用 1 cm^3 冰浴的质量浓度为 5% 三氯乙酸淋洗滤筒内壁和滤膜，重复 8 次；

d) 制样：取出滤膜用两把无齿镊子，使其载滤物面朝内，对折两次，置闪烁瓶底部；加乙酸乙酯至装有滤膜的闪烁瓶，使其淹没滤膜（一般用 0.5 cm^3～1.0 cm^3），待滤膜完全溶解后，再加入 5 cm^3 闪烁液，并混匀，静置 2 d，使放射性物质均匀分布于闪烁液中；

e) 测定：将测样放入液体闪烁仪中测定放射性活度值；

f) 计算胸腺嘧啶核苷吸收率

$$R_i = \frac{U_s - U_b}{S_a \cdot T \cdot V} \qquad \cdots\cdots(27)$$

式中：

R_i——胸腺嘧啶核苷吸收率，单位为毫摩尔每升小时[mmol/(L·h)]；

U_s——测样放射性活度值，单位为贝可(Bq)；

U_b——空白样放射性活度值，单位为贝可(Bq)；

S_a——^{3}H 在甲基-胸腺嘧啶核苷中的放射性比活度，单位为贝可每毫摩尔(Bq/mmol)；

T——样品培养时间，单位为小时(h)；

V——样品加入量，单位为升(L)。

g) 计算细菌细胞生产

$$BP = 1.4 \times 10^{15} \cdot R_i \qquad \cdots\cdots(28)$$

式中：

BP——细菌生产力，单位为个每升小时[cells/(L·h)]；

1.4×10^{15}——吸收 1 mmol 胸腺嘧啶核苷所生产的细菌个数，单位为个每毫摩尔(cells/mmol)；

R_i——胸腺嘧啶核苷吸收率，单位为毫摩尔每升小时[mmol/(L·h)]。

h) 将分析结果记录于表 H.11。

6.3.4.2.1.2 ^{3}H-亮氨酸示踪法

工作程序除“淋洗”和“计算细菌细胞生产”以外，其余步骤同 6.3.4.2.1.1。

——淋洗：用冰浴的质量浓度为 5% 三氯乙酸淋洗滤筒内壁、滤器和滤膜 2 次，每次 3 cm^3；最后在抽滤状态下，取上滤筒，用少量冰浴的体积分数为 80%乙醇漱洗滤筒底部压盖部分的滤膜，抽滤至干；停止抽滤，取下滤膜，置于吸水纸上，待乙醇完全挥发。

——计算细菌生物量生产

$$BP = 1.55 \times 10^6 \cdot R_i \qquad \cdots\cdots(29)$$

式中：

BP——细菌生产力(以 C 计)，单位为微克每升小时[μg/(L·h)]；

R_i——亮氨酸吸收率，单位为毫摩尔每升小时[mmol/(L·h)]；

1.55×10^6——细菌吸收 1 mmol 亮氨酸转换为 C 生产量(以 μg 为单位)的换算系数。

注 1：菌体亮氨酸＝0.073 菌体蛋白质，菌体碳＝0.86 菌体蛋白质，亮氨酸分子量为 131.2。

注 2：当本示踪法与[甲基-^{3}H] 胸腺嘧啶核苷酸示踪法的测定结果有异议时，以本示踪法为仲裁示踪法。

6.3.4.2.2 微生物异养活性测定

6.3.4.2.2.1 ^{14}C 葡萄糖示踪法

工作程序如下：

a) 抽滤：使用微孔滤膜(孔径 0.2 μm，直径 25 mm)负压(30 kPa)抽滤样品至干，用 5 cm^3 无菌蒸馏水淋洗滤筒和滤膜，重复 3 次；

b) 制样：将滤膜迭成 4 折放入闪烁瓶底部，加乙酸乙酯使其淹没滤膜，待滤膜完全溶解后，再加入 5 cm^3 闪烁液，盖紧瓶口，并于暗处静置 24 h；

c) 测定：将制样放入闪烁仪，测定放射性活度值；

d) 标定：测定每批葡萄糖工作母液的^{14}C放射性活度值；

e) 计算葡萄糖吸收率

$$R_a = \frac{(R_s - R_b) \cdot A}{C \cdot T} \qquad \cdots\cdots(30)$$

式中：

R_a——葡萄糖吸收率，单位为微克每升小时[μg/(L·h)]；

R_s——样品放射性活度值，单位为贝可(Bq)；

R_b——空白样放射性活度值，单位为贝可(Bq)；

A——样品含添加的非放射性葡萄糖浓度，单位为微克每升(μg/L)；

C——添加^{14}C放射性活度，单位为贝可(Bq)；

T——样品培养时间，单位为小时(h)。

f) 将分析结果记录于表H.12。

6.3.4.2.2.2 生态呼吸率测定——溶解氧滴定法

a) 测定：按GB/T 12763.4中的“溶解氧测定”方法进行。本测定推荐使用进口的高精度溶解氧自动测定仪。

b) 计算

$$R_e = \frac{DO_0 - DO_t}{t} \qquad \cdots\cdots(31)$$

式中：

R_e——生态呼吸率，单位为微摩尔每升天[μmol/(L·d)]；

DO_0——样品培养前溶解氧浓度，单位为微摩尔每升(μmol/L)；

DO_t——样品培养后溶解氧浓度，单位为微摩尔每升(μmol/L)；

t——样品培养时间，单位为天(d)。

6.3.4.3 细菌比生长率(specific growth rate)、倍增时间(doubling time)、世代时间(generation time)的计算

a) 细菌比生长率

$$\mu = \frac{24BP}{BB} \qquad \cdots\cdots(32)$$

式中：

μ——细菌比生长率，单位为每天(d^{-1})；

BP——细菌生产力，单位为个每升小时[cells/(L·h)]或 微克每升小时[μg/(L·h)]；

BB——细菌现存量，单位为个每升(cells/L)或微克每升(μg/L)；

24——细菌生产力的单位由“h”转化为“d”的计算系数。

b) 世代时间

$$G = \frac{\ln 2}{\mu} = \frac{0.693}{\mu} \qquad \cdots\cdots(33)$$

式中：

G——世代时间，单位为天(d)；

μ——细菌比生长速率，单位为每天(d^{-1})。

c) 倍增时间

$$DT = \frac{1}{g} = \frac{\mu}{0.693} \qquad \cdots\cdots(34)$$

式中：

DT——倍增时间，单位为每天(d^{-1})；

μ——细菌比生长速率，单位为每天(d^{-1})；

g——世代时间，单位为天(d)。

6.3.4.4 泥样微生物计数

6.3.4.4.1 微生物计数

见 6.3.4.1.2.2。

6.3.4.4.2 计算泥样含菌数

$$N = \frac{N_a \cdot V_s \cdot D}{V \cdot W} \qquad \cdots\cdots(35)$$

式中：

N——菌数，单位为个每克(CFU/g)；

N_a——三个平板的平均菌落数，单位为个(CFU)；

V_s——悬浮液体积，单位为毫升(mL)；

D——悬浮液的再稀释倍数；

V——接种量，单位为毫升(mL)；

W——制悬浮液泥样的干重，单位为克(g)。

将分析结果记录于表 H.10。

6.3.5 微生物分类鉴定

条件许可时应进行微生物分类鉴定。

6.3.5.1 菌种分离、纯化和保存

用接种针从微生物培养计数的平板或滤膜上挑取全部菌落或部分区域的所有菌落，挑取时注意选择形态特征不同的菌落，挑取的菌落在新平板上反复划线分离，培养数天，选取新形成的单菌落，直至纯种，再接于斜面培养，长成后置冰箱保存待鉴定；如果不是纯种，应继续纯化。

6.3.5.2 种类鉴定

种类鉴定方法包括传统鉴定法、仪器自动鉴定法和分子生物学鉴定法。

6.3.5.3 菌种保藏

对一些有代表性或有保存价值的菌种，按菌种保藏法的要求，登记入库，长期保存。

6.4 资料整理

利用计算机保存分析、计算数据、制作报表、绘制分布图。

6.4.1 填写报表

按本部分的有关规定填写。

分别将病毒、细菌、放线菌、酵母菌和霉菌的数量(包括细菌生物量)以及细菌生产力和细菌异养活性的测定结果。

6.4.2 绘制分布图

6.4.2.1 断面分布图

一般以等值线表示，取值标准视具体情况而定。

a) 微生物数量和细菌生物量断面分布图；

b) 细菌生产力断面分布图；

c) 微生物异养活性断面分布图；

d) 生态呼吸率断面分布图。

6.4.2.2 大面分布图

一般以等值线表示，取值标准视具体情况而定。

a) 微生物数量和细菌生物量大面分布图；

b) 细菌生产力大面分布图；

c) 微生物异养活性大面分布图；

d) 生态呼吸率大面分布图。

7 微微型、微型和小型浮游生物调查

7.1 技术要求和调查要素

7.1.1 技术要求

7.1.1.1 采水层次

见 4.2.4.1 表 1，具体层次视不同调查项目的要求确定。

7.1.1.2 采水量

7.1.1.2.1 浮游植物

水深大于 200 m 的海区，每次采水不少于 1 000 cm^3；水深小于 200 m 的海区不少于 500 cm^3；发生富营养化或赤潮海区视具体情况而定，一般每次采水 100 cm^3。

7.1.1.2.2 浮游动物

采水量依动物的密度而定，一般调查控制在 1 dm^3～50 dm^3 之间。在浮游动物丰富的内湾和发生动物性赤潮的水域，采水量为 100 cm^3。

7.1.1.3 垂直拖网深度

水深大于 200 m 的海区拖网深度为 200 m，水深小于 200 m 的海区拖网深度从底至表。

7.1.1.4 垂直分段拖网水层

根据测站深度规定采样水层如表 3（有些专项调查可视项目要求根据现场温度、盐度、叶绿素等跃层分段采样）。

表 3 微微型、微型和小型浮游生物垂直分段采样水层

单位为米

测站水深范围	采样水层
<20	10～0，底～10
20～30	10～0，20～10，底～20
30～50	10～0，20～10，30～20，底～30
50～100	10～0，20～10，30～20，50～30，底～50
100～200	10～0，20～10，30～20，50～30，100～50，底～100
>200	10～0，20～10，30～20，50～30，100～50，200～100

7.1.1.5 连续观测时间与次数

水深小于 50 m 的海区每 3 h 采样一次，共采 9 次；水深大于 50 m 的海区每 4 h 采样一次，共采 7 次。

7.1.1.6 种类鉴定与计数

除需作特殊处理的微微型、微型浮游生物种类以及培养观察的特殊类群之外，原则上鉴定到种的标本比例应在 80％以上；鉴定到属的比例应在 90％以上。水采样品每次实际标本镜检数不少于 100 个～200 个；网采样品每次实际标本镜检数不少于 500 个。

7.1.2 调查要素

调查要素包括微微型、微型和小型浮游生物的种类组成和丰度分布（时间、空间分布）。

7.2 采样

7.2.1 采样设备

7.2.1.1 采水器

采水器容积可为 2.5 dm^3、5 dm^3 或 10 dm^3。

7.2.1.2 采泥器

根据条件采用箱式采样器、多管采样器或弹簧采泥器采集甲藻等孢囊样品。

7.2.1.3 网具

根据调查海区情况和采样对象选用，见表4。

表4 微型和小型浮游生物网具的规格及适用对象

序号	网具名称	网长/cm	网口内径/cm	网口面积/m^2	筛绢规格(孔径近似值 mm)	适用范围及采集对象
1	小型浮游生物网	280	37	0.1	JF 62(0.077)<br>JP 80(0.077)	适用于30 m以深垂直或分段采集小型浮游生物
2	浅水 Ⅲ 型浮游生物网	140	37	0.1	JF 62(0.077)<br>JP 80(0.077)	适用于30 m以浅垂直或分段采集小型浮游生物
3	手拖定性浮游植物网	60	22	0.038	NY20HC(0.020)<br>NY10HC(0.010)	用于小型和微型浮游植物的种类组成分析以及藻种的分离
注：筛绢规格见 GB/T 14014。						

7.2.1.4 网底管

网底管套的筛绢必须与网衣筛绢的规格相同。

7.2.1.5 网口流量计

使用前必须经过标定，每航次标定一次。

7.2.1.6 量角器

角弧形量角器。

7.2.1.7 沉锤

根据水流速度和风浪大小，使用质量为10 kg～40 kg的铅制沉锤。

7.2.1.8 绞车及钢丝绳

绞车变速范围为0.3 m/s～1 m/s，并附有排缆装置和钢丝绳计数器，钢丝绳直径为3.6 mm～5.0 mm。

7.2.1.9 吊杆

高度为5 m～6 m(深水拖网须大于6 m)，负荷为500 kg～1 000 kg；吊杆的舷间距1 m左右，并能调节位置。

7.2.1.10 冲水设备

水泵、水管、水桶和吸水球等，用于冲喷收集粘贴在网衣或网底管套筛绢上的标本。

7.2.2 采样前的准备

a) 根据调查要素、站数、层次计算采样数量，配以足量的样品瓶、相应的固定剂及其他器材。

b) 固定液：

——鲁哥氏液(Lugol's solution)：100 g碘化钾溶于1 dm^3 蒸馏水，加入50 g碘使其溶解，再加入100 cm^3 冰醋酸；

——缓冲甲醛溶液：商用甲醛(体积分数约为40%)加入同量蒸馏水，1 dm^3 约20%的甲醛溶液加100 g六次甲基四胺；

——多聚甲醛溶液(体积分数为25%)。

7.2.3 海上采样与样品保存

7.2.3.1 微微型(光合)浮游生物

a) 主要采集特定水层个体小于2.0 μm的生物；

b) 微微型浮游生物包括异养细菌和自养型生物。对于微微型光合浮游生物(photosynthetic picoplankton),在此只表述使用落射荧光显微镜,根据其所含色素的荧光特性可区分为单细胞的蓝细菌(cyanobacteria)和微微型光合真核生物(photosynthetic pico-eukaryote),前者又通常以聚球藻(Synechococcus sp)占优势;

c) 采水样使用进口多瓶采水器或国产有机玻璃制成的 2.5 dm^3 采水器;

d) 采集 50 cm^3～200 cm^3 水样,加多聚甲醛溶液固定 (1%终浓度),用液氮保存。采样情况记录于表 H.14。

7.2.3.2 **微型浮游生物**

a) 主要采集特定水层个体小于 20 μm 的微型金藻、微型甲藻、微型硅藻、无壳纤毛虫和领鞭虫等样品;

b) 按预定水层和规定量采集水样。如需去除大于 20.0 μm 的生物,可先用孔径 20 μm 的筛绢预过滤。样品用鲁哥氏液固定,每 1 L 水样加入 10 cm^3～15 cm^3,根据样品的实际浓度可作适当增减。如需要对样品做电镜观察分析,则选用戊二醛固定,根据样品浓度可加入样品体积的 2%～5%。采样情况记录于表 H.14。

7.2.3.3 **小型浮游生物**

a) 主要采集水柱中个体小于 200 μm 的绝大部分浮游植物、无壳纤毛虫、砂壳纤毛虫、轮虫、桡足类幼体、放射虫和有孔虫等样品;

b) 按不同水深选用小型浮游生物网或浅水Ⅲ型网进行垂直拖网,拖网速度:落网为 0.5 m/s;起网为 0.5 m/s～0.8 m/s,样品用缓冲甲醛溶液固定,加入量为样品体积的 5%,根据样品的实际浓度可作适当增减,并记录于表 H.14;

c) 用手拖定性浮游植物网进行水平(可在网口三根网绳的接合点加一个使锤)或垂直拖网,样品用鲁哥氏液或缓冲甲醛溶液固定;

d) 如需要对样品做电镜观察分析,则选用戊二醛固定,根据样品浓度可加入样品体积的2%～5%。

7.2.3.4 **孢囊**

a) 选择在软质沉积环境如海底凹处和内湾等处采集孢囊沉积物样品;

b) 根据条件采用箱式采样器、多管采样器或弹簧采泥器采样,取沉积物上部 10 cm 软泥,分别用孔径 0.12 mm、0.038 mm 和 0.022 mm 筛网过筛,再对已过筛的样品进行超声波处理以去除杂质;

c) 一般对样品进行低温避光保存,固定剂会使孢囊失去部分诊断特征,如需要长期保存,可选用缓冲甲醛溶液固定。

7.3 **样品分析**

7.3.1 **主要仪器和设备**

落射荧光显微镜、光学显微镜、倒置显微镜、沉降器、滤器、支架、抽滤瓶、手持泵或真空泵。

7.3.2 **样品编号**

各类样品须有总编号。总编号由代表采样海区、采样方式、使用网型、采样年份和样品序号等内容的代号依次组成,每份贮存样品的瓶外须贴有总编号的外标签,瓶内须放有总编号、站号和采样日期等内容的内标签(见附录 B.1),填写表 H.16。

7.3.3 **样品分类鉴定与丰度测定**

标本鉴定在有条件情况下采用活体与固定样品相结合、网采与水采样品相结合的方法,尽可能获取水体中浮游生物群落的真实信息;定量计数一般以采水样品为准,水柱的定量计数以网采样品为准,网采样品可作为种类组成分析的补充和某些个体大于拖网筛绢孔径的种类的定量计数。

7.3.3.1 **微微型光合浮游生物**

a) 按照预定层次量取 10 cm^3～50 cm^3 水样；

b) 通过直径为 25 mm、孔径为 0.2 μm 的黑色核孔滤膜，抽滤负压不超过 50 kPa；

c) 取下滤筒，将滤膜放在载玻片上，在滤膜上加一滴水样，盖上盖玻片（滤膜两面均不能有气泡）；

d) 在落射荧光显微镜下使用绿光或蓝光激发，一般使用 40 倍物镜观察，随机取至少 20 个视野，分别计数具有光亮的桔黄色荧光的含藻红蛋白的聚球藻细胞和呈砖红色荧光的含叶绿素的微微型光合真核生物细胞。计数结果记录于表 H.17。

7.3.3.2 **微型和小型浮游生物鉴定与计数**

7.3.3.2.1 **沉降计数法**

用于采水样品浮游生物计数，见附录 B.2.1，计数结果记录于表 H.19。

7.3.3.2.2 **浓缩计数法**

用于网采或采水样品浮游生物计数，见附录 B.2.2，计数结果记录于表 H.18。

7.3.3.3 **甲藻孢囊鉴定与计数**

浓缩或直接显微计数，计数结果记录于表 H.20。

7.4 **资料整理**

7.4.1 **丰度计算**

7.4.1.1 **落射荧光显微镜计数**

$$N = \frac{N_a \cdot S}{S_1 \cdot V} \qquad \cdots\cdots (36)$$

式中：

N——样品中细胞数，单位为个每毫升(cells/mL)；

N_a——各视野平均细胞数，单位为个(cells)；

S——滤膜滤水面积，单位为平方厘米(cm^2)；

S_1——视野面积，单位为平方厘米(cm^2)；

V——过滤样品量，单位为毫升(mL)。

7.4.1.2 **光学显微镜计数**

7.4.1.2.1 **沉降计数**

$$C = \frac{N_i}{V_i} \qquad \cdots\cdots (37)$$

式中：

C——单位体积海水中标本总量，单位为个每毫升(cells/mL)；

N_i——三个分样计数的标本总个数，单位为个(cells)；

V_i——三个分样的总体积，单位为毫升(mL)。

7.4.1.2.2 **浓缩计数**

7.4.1.2.2.1 **网采样品**

$$C = \frac{n \cdot V_1}{V_2 \cdot V_n} \qquad \cdots\cdots (38)$$

式中：

C——单位体积海水中标本总量，单位为个每立方米(cells/m^3)；

n——取样计数个数，单位为个(cells)；

V_1——水样浓缩后的体积，单位为毫升(mL)；

V_2——滤水量，单位为立方米(m^3)；

V_n——取样计数的体积，单位为毫升(mL)。

7.4.1.2.2.2 采水样品

$$C = \frac{n' \cdot V'_1}{V'_2 \cdot V'_n} \quad \cdots\cdots (39)$$

式中：

C——单位体积海水中标本总量，单位为个每升(cells/L)；

n'——取样计数个数，单位为个(cells)；

V'_1——水样浓缩后的体积，单位为毫升(mL)；

V'_2——原采水量，单位为升(L)；

V'_n——取样计数的体积，单位为毫升(mL)。

7.4.1.3 甲藻孢囊细胞浓度

孢囊细胞浓度以 cells/cm^3 沉积物或 cells/g 沉积物干重计算。

7.4.2 填写报表

按本部分的要求和规定填写各种报表。

7.4.3 绘制分布图

平面分布图一般用等值线表示，取值标准如下：

a) 浮游植物细胞总数(网采样品单位为 10^4cells/m^3，采水样品单位为 10^2cells/dm^3)：5,10,50,100,500,1 000,5 000,10 000；

b) 浮游植物优势种细胞丰度(网采样品单位为 10^4 cells/m^3，采水样品单位为 10^2cells/dm^3)：1,5,10,50,100,500,1 000,5 000；

c) 孢囊细胞丰度(单位为 cells/cm^3)：1,10,100；

d) 浮游动物个体丰度(网采样品单位为 10^2ind/m^3，采水样品单位为 10^2ind/dm^3)：1,5,10,50,100。

8 大、中型浮游生物调查

8.1 技术要求和调查要素

8.1.1 技术要求

8.1.1.1 大面观测垂直拖网深度

水深大于 200 m 的海区拖网深度为 200 m。水深不足 200 m 的海区从底至表拖曳。

8.1.1.2 断面观测垂直分段拖网水层

根据测站或采集深度规定采样水层如表 5（有些专项调查可视研究对象或已知的现场温、盐等跃层分布状况酌情调整)。

表 5 大中型浮游生物垂直分段采样水层

单位为米

测站水深范围	采样水层
<20	10～0,底～10
20～30	10～0,20～10,底～20
30～50	10～0,20～10,30～20,底～30
50～100	10～0,20～10,50～20,底～50
100～200	20～0, 50～20,100～50,底～100
200～300	20～0,50～20,100～50,200～100,底～200
300～500	20～0, 50～20, 100～50, 200～100, 300～200,底～300
500～1 000	50～0, 100～50, 200～100, 300～200, 500～300,底～500
注：1 000 m 以深采样水层视调查对象而定。	

8.1.1.3 连续观测时间与次数

水深小于 50 m 的每 3 h 采样一次，共采 9 次；水深大于 50 m 而采样深度在 500 m 以浅的每 4 h 采样一次，共采 7 次，亦可视研究对象酌情缩短采集的间隔时间，并相应增加采样次数。采集深度大于 500 m 的采集间隔时间与相应的采集次数视具体情况而定。

8.1.1.4 垂直拖网

现场调查时，垂直拖网（尤其是起网过程中）不得停顿，钢丝绳倾角不得大于 45°，如果遇到大于 45° 时，只能作为定性样品，同时，应重新采样一次。冲网时应保持较大的水压，确保网中样品全部收入标本瓶。

8.1.1.5 生物量测定精密度

a) 体积分数测定±0.1 cm^3；

b) 湿重生物量测定±1 mg；

c) 干重生物量测定±0.1 mg。

8.1.1.6 样品分析

样品分析要求 90%以上的物种鉴定到种（幼体除外），并按种计数。

8.1.2 调查要素

调查要素包括大、中型浮游生物种类组成和数量分布（时间、空间分布）。

8.2 采样

8.2.1 采样设备

8.2.1.1 网具

30 m 以浅海域应采用浅水Ⅰ型或Ⅱ型浮游生物网、30 m 以深海域应采用大型或中型浮游生物网作垂直或分段取样，如有特殊要求，可选择其他网具，见表 6。一些特殊研究对象可参照渔业资源声学调查方法（见附录 G）。

表 6 大中型浮游生物网具的规格及适用对象

序号	网具名称	网长/cm	网口内径/cm	网口面积/m^2	筛绢规格（孔径近似值 mm）	适用范围及采集对象
1	大型浮游生物网	280	80	0.5	CQ 14(0.505)<br>JP 12(0.507)	适用于 30 m 以深垂直或分段采集大、中型浮游动物、鱼卵和仔、稚鱼。
2	中型浮游生物网	280	50	0.2	CB 36(0.160)<br>JP 36(0.169)	适用于 30 m 以深垂直或分段采集中、小型浮游动物和夜光藻。
3	浅水Ⅰ型浮游生物网	145	50	0.2	CQ 14(0.505)<br>JP 12(0.507)	适用于 30 m 以浅垂直或分段采集大、中型浮游动物、鱼卵和仔、稚鱼。
4	浅水Ⅱ型浮游生物网	140	31.6	0.08	CB 36(0.160)<br>JP 36(0.169)	适用于 30 m 以浅垂直或分段采集中、小型浮游动物和夜光藻。
5	深水浮游生物网	510	113	1.0	CQ 20(0.336)<br>JQ 20(0.322)	适用于生物高密度区长距离拖曳采集大、中型浮游动物。
6	WP2 型浮游生物网	271	57	0.25	CB 30(0.198)<br>JP 32(0.202)	适用于采集中型浮游动物（国外较常用）。
7	北太平洋浮游生物标准网	180	45	0.16	CQ 20(0.336)<br>JQ 20(0.322)	适用于采集大、中型浮游动物（国外较常用）。
8	WP3 型浮游生物网	279	113	1.0	JP7(1.025)	适用于采集活动能力较大的浮游动物和大型水母等。

8.2.1.2 **网底管**

网底管套的筛绢必须与网衣筛绢的规格相同。

8.2.1.3 **网口流量计**

使用前必须经过标定，每航次标定一次。

8.2.1.4 **量角器**

角弧形量角器(垂直采样过程中钢丝绳倾角＞45°的不可作为定量样品)。

8.2.1.5 **沉锤**

根据水流速度和风浪大小，使用质量为 10 kg～40 kg 的铅制沉锤。

8.2.1.6 **绞车及钢丝绳**

绞车变速范围为 0.3 m/s～1 m/s，并附有排缆装置和钢丝绳计数器，钢丝绳直径为 3.6 mm～5.0 mm。

8.2.1.7 **吊杆**

高度为 5 m～6 m(深水拖网需大于 6 m)，负荷为 500 kg～1 000 kg；吊杆的舷间距 1 m 左右，并能调节位置。

8.2.1.8 **冲水设备**

水泵、水管、水桶和吸水球等。供收集非活体样品时使用，前二者用于起网出水面至一定高度时从凌空的网外侧冲洗，使粘网的标本集中于网底管；后二者用于喷冲网底管筛绢套上的样品。

8.2.2 **采样前的准备**

根据调查要素、站数，层次计算采样数量，配以足量的样品瓶、固定剂及其他器材。

8.2.3 **海上采样**

一般只采单样，供湿重生物量测定后进行种类鉴定与个体计数，若同时要求体积分数或干重生物量测定，则应同时采双样或三样，并记录于表 H.14 或表 H.15。

8.2.3.1 **拖网速度**

落网为 0.5 m/s；起网为 0.5 m/s～0.8 m/s。

8.2.3.2 **样品处理**

样品用中性甲醛溶液固定，加入量为样品体积的 5%。需进行电镜观察的样品，用戊二醛固定，加入量为样品体积的 2%～5%。

8.3 **样品分析**

8.3.1 **样品编号**

各类样品需有总编号。总编号由代表采样海区、采样方式、使用网型、采样年份和样品序号等内容的代号依次组成(见附录 B.1)，填写表 H.16。

8.3.2 **填写标签**

每份贮存样品的瓶外需贴有总编号的外标签，瓶内需放有总编号、海区、站号和采集日期等内容的内标签。

8.3.3 **浮游动物生物量测定**

以大型或浅水Ⅰ型浮游生物网的样品为准。特殊研究对象或特殊海区可视具体情况而定。

8.3.3.1 **体积分数测定**

见附录 B.3.1，测定结果记录于表 H.21。

8.3.3.2 **湿重生物量测定**

见附录 B.3.2，测定结果记录于表 H.22。

8.3.3.3 **干重生物量测定**

见附录 B.3.3，测定结果记录于表 H.23。

8.3.4 浮游动物种类鉴定与个体计数

以大型或浅水Ⅰ型浮游生物网的样品为准，特殊研究对象或特殊海区可视具体情况而定，见附录B.3.4，鉴定与计数结果记录于表H.24。

8.3.5 夜光藻的计数

夜光藻以中型或浅水Ⅱ型网为准，但在其个体较小(<200 μm)的季节(如南方的秋、冬季)应参考小型网或浅水Ⅲ型网的采集结果。见附录B.3.4，结果记录于表H.25。

8.4 资料整理

8.4.1 计算浮游动物生物量

8.4.1.1 体积分数

$$\gamma_B = \frac{V_B}{V} \qquad \cdots\cdots(40)$$

式中：

γ_B——单位体积海水中浮游动物的体积分数，数值以10^{-6}表示；

V_B——样品体积，单位为毫升(mL)；

V——滤水量，单位为立方米(m^3)。

8.4.1.2 湿重生物量

$$P_B = \frac{m_B}{V} \qquad \cdots\cdots(41)$$

式中：

P_B——单位体积海水中浮游动物的湿重含量，单位为毫克每立方米(mg/m^3)；

m_B——样品湿重含量，单位为毫克(mg)；

V——滤水量，单位为立方米(m^3)。

8.4.1.3 干重生物量

$$P'_B = \frac{m'_B}{V} \qquad \cdots\cdots(42)$$

式中：

P'_B——单位体积海水中浮游动物的干重含量，单位为毫克每立方米(mg/m^3)；

m'_B——样品干重，单位为毫克(mg)；

V——滤水量，单位为立方米(m^3)。

8.4.2 计算浮游动物或夜光藻的个体密度

$$C_B = \frac{N_B}{V} \qquad \cdots\cdots(43)$$

式中：

C_B——单位体积海水中浮游动物或夜光藻的个体密度，单位为个每立方米(ind/m^3或$cells/m^3$)；

N_B——全网个数，单位为个(ind或cells)；

V——滤水量，单位为立方米(m^3)。

8.4.3 填写报表

按本部分的有关规定填写报表。

8.4.4 绘制分布图

平面分布图一般用等值线表示。在特殊情况下(如测站稀少或测站不连续时)也可用不同等级的圆圈或符号表示。

8.4.4.1 等值线取值标准如下：

a) 夜光藻个体密度(单位为$10^2 cells/m^3$)：5,10,50,100,500,1 000,5 000；

b) 浮游动物湿重生物量(单位为mg/m^3)：5,10,25,50,100,250,500,1 000,5 000；

c) 浮游动物干重生物量(单位为 mg/m^3)：1，2.5，5，10，25，50，100；

d) 浮游动物体积分数(单位为 10^{-6})：0.2，0.5，1，2.5，5，10；

e) 浮游动物总个体密度(单位为 ind/m^3)：5，10，25，50，100，250，500，1 000，5 000；

f) 浮游动物主要种或主要类别的个体密度(单位为 ind/m^3)：1，5，10，25，50，100，250，500，1 000，5 000。

g) 以上取值标准，可视具体情况酌情增减。

8.4.4.2 **圆圈或符号取值标准如下：**

a) 夜光藻个体密度(单位为 10^2cells/m^3)：＞0～5，＞5～10，＞10～50，＞50～100，＞100～500，＞500～1 000，＞1 000～5 000，＞5 000；

b) 浮游动物湿重生物量(单位为 mg/m^3)：＞0～5，＞5～10，＞10～25，＞25～50，＞50～100，＞100～250，＞250～500，＞500～1 000，＞1 000～5 000，＞5 000；

c) 浮游动物干重生物量(单位为 mg/m^3)：＞0～1，＞1～2.5，＞2.5～5，＞5～10，＞10～25，＞25～50，＞50～100，＞100；

d) 浮游动物体积分数(单位为 10^{-6})：＞0～0.2，＞0.2～0.5，＞0.5～1，＞1～2.5，＞2.5～5，＞5～10，＞10；

e) 浮游动物总个体密度(单位为 ind/m^3)：＞0～5，＞5～10，＞10～25，＞25～50，＞50～100，＞100～250，＞250～500，＞500～1 000，＞1 000～5 000，＞5 000；

f) 浮游动物主要种或主要类别的个体密度(单位为 ind/m^3)：＞0～1，＞1～5，＞5～10，＞10～25，＞25～50，＞50～100，＞100～250，＞250～500，＞500～1 000，＞1 000～5 000，＞5 000。

g) 以上取值标准，可视具体情况酌情增减。

9 鱼类浮游生物调查

9.1 技术要求和调查要素

9.1.1 技术要求

9.1.1.1 垂直或倾斜拖网深度

水深大于 200 m 的海区拖网深度为 200 m 至表垂直拖网或斜拖，水深小于 200 m 的则由底至表垂直拖网或斜拖。

9.1.1.2 水平拖网深度

水平拖网深度为 0 m～3m 层。

9.1.1.3 垂直或倾斜分段拖网水层

根据测站深度、调查性质和目的来确定。

9.1.1.4 种类鉴定

主要鱼类浮游生物应鉴定到属或科。

9.1.2 调查要素

调查要素包括鱼卵和仔、稚鱼的种类组成和数量分布(时间和空间的分布)。

9.2 采样

9.2.1 采样设备

9.2.1.1 网具

30 m 以浅海域应采用浅水Ⅰ型浮游生物网垂直取样，30 m 以深海区应采用大型浮游生物网垂直取样或用双鼓网(即：Bongo 网)倾斜取样。此外可根据海区位置或深度、调查目的和采样对象选用不同的网具，见表 7。

9.2.1.2 网底管

网底管套的筛绢与网衣筛绢的规格必须相同。

9.2.1.3 网口流量计

使用前必须经过标定，每航次标定一次。

9.2.1.4 量角器

角弧形量角器。

9.2.1.5 沉锤

根据水流速度和风浪大小，使用质量为 10 kg～40 kg。

9.2.1.6 绞车及钢丝绳

绞车变速范围为 0.31 m/s～1 m/s，并附有排缆装置和钢丝绳计数器，钢丝绳直径为 3.6 mm～5.0 mm。

9.2.1.7 吊杆

高度为 5 m～6 m(深水拖网大于 6 m)。负荷为 500 kg～1 000 kg；吊杆的舷间距 1 m 左右，并能调节位置。

9.2.1.8 冲水设备

水泵、水管、水桶和吸水球等。

9.2.2 样品采集

9.2.2.1 定性采样

一般在海水表层(0 m～3 m)或其他水层进行水平拖网 10 min～15 min，船速为 1 kn～2 kn。所用网具，水层及拖网时间应分别根据调查的目的和调查区鱼卵和仔、稚鱼密度来决定。并记录于表 H.26。

表 7 鱼类浮游生物网具的规格及适用对象

序号	网具名称	网长/cm	网口内径/cm	网口面积/m^2	筛绢规格(孔径近似值 mm)	适用范围、采集方法和对象
1	大型浮游生物网	280	80	0.5	CQ14(0.505)<br>JP12(0.507)	我国最常用的网具，适用于表层水平拖曳及 30 m 以深、200 m以浅垂直采集鱼卵和仔、稚鱼。
2	浅水Ⅰ型浮游生物网	145	50	0.2	CQ14(0.505)<br>JP12(0.507)	适用于 30 m 以浅垂直采集鱼卵和仔、稚鱼。
3	双鼓网(Bongo 网)	360<br>360	60<br>60	0.28<br>0.28	CQ14(0.505)<br>JP12(0.507)<br>CQ20(0.336)<br>JQ20(0.322)	这是联合国粮农组织推荐的国际上通用的双联网。适用于定量垂直或倾斜采集鱼卵和仔、稚鱼。网口需系流量计。
4	北太平洋浮游生物标准网	180	45	0.16	CQ20(0.336)<br>JQ20(0.322)	国际上常用的网具；适用于定量垂直或平拖采集鱼卵和仔、稚鱼。
5	WP3 网	279	113	1.0	JP7(1.025)	适用于采集个体较大、活动力强的仔、稚鱼。
注：采用序号 1～4 的网型垂直或斜拖取样时，应结合大型浮游生物网表层平拖取样作为定性样品。						

9.2.2.2 定量采样

由海底至海面垂直或倾斜拖网。落网速度为 0.5 m/s，起网速度为 0.5 m/s～0.8 m/s。也可采用定性采样方法进行，但网口需系流量计。所用网具见 9.2.1.1，并记录于表 H.26。

9.2.2.3 样品处理

样品用中性甲醛溶液固定，加入量为样品体积的 5%。

9.3 样品分析

9.3.1 样品编号

各类样品的总编号应根据采样海区，采样方式，采用网型，采样年份和样品序号等内容的代号依次编写(见附录 B.1)，填写在表 H.27。

9.3.2 填写标签

每份保存样品除在瓶外贴有总编号的外标签外，瓶内还需放有总编号、站号和采样日期等内容的内标签。

9.3.3 鱼卵和仔、稚鱼个体数

以定量样品为准，定性样品为参考，结果记录于表 H.28 和表 H.29。

9.4 资料整理

9.4.1 丰度的计算

9.4.1.1 垂直采集的样品

$$G = \frac{N}{V} \qquad \cdots\cdots (44)$$

式中：

G——单位体积海水中鱼卵或仔、稚鱼个体数，单位为粒每立方米或尾每立方米(ind/m^3)；

N——全网鱼卵或仔、稚鱼个体数，单位为粒或尾(ind)；

V——滤水量，单位为立方米(m^3)。

9.4.1.2 平拖、斜拖或垂直取样

$$G_a = \frac{N_a}{S \cdot L \cdot C} \qquad \cdots\cdots (45)$$

式中：

G_a——单位体积海水中鱼卵或仔、稚鱼个体数，单位为粒每立方米或尾每立方米(ind/m^3)；

N_a——全网鱼卵或仔、稚鱼个体数，单位为粒(ind)或尾(ind)；

S——网口面积，单位为平方米(m^2)；

L——流量计转数；

C——流量计校正值。

9.4.1.3 水平拖曳样品(定性)

以粒/网(ind/net)或尾/网(ind/net)计算。

9.4.2 填写报表

按本部分的有关规定填写报表。

9.4.3 绘制分布图

平面分布图一般用等值线或不同量级的圆圈符号表示。

9.4.3.1 等值线的取值标准如下：

a) 鱼卵和仔、稚鱼总量(单位为 ind/m^3 或 ind/100 m^3)：1，5，10，25，50，100，250，500，1 000，5 000；
b) 鱼卵和仔、稚鱼主要科、属或种(单位为 ind/m^3 或 ind/100 m^3)：1，5，10，25，50，100，200，300，400，500，1 000；

c) 数量小于上述等级时,可用“+”标在测站上,以示出现;

d) 上述取值标准,可视具体情况酌情增减。

9.4.3.2 圆圈的取值标准如下:

a) 鱼卵和仔、稚鱼总量(ind/m^3 或 ind/100 m^3)为>0~1,>1~5,>5~10,>10~25,>25~50,>50~100,>100~250,>250~500,>500~1 000,>1 000~5 000,>5 000;

b) 鱼卵和仔、稚鱼主要科、属或种(ind/m^3 或 ind/100 m^3)为>0~1,>1~5,>5~10,>10~25,>25~50,>50~100,>100~200,>200~300,>400~500,>500~1 000,>1 000;

c) 数量小于上述等级时,可用“+”标在测站上,以示出现;

d) 上述取值标准,可根据具体情况酌情增减。

10 大型底栖生物调查

10.1 技术要求和调查要素

10.1.1 技术要求

10.1.1.1 采泥样面积

每站不小于 0.2 m^2。

10.1.1.2 套筛孔径

上层 2.0 mm~5.0 mm,中层 1.0 mm,底层 0.5 mm。

10.1.1.3 生物量测定精密度

湿重生物量±0.01 g,干重±0.1 mg;烘干温度 70℃~100℃。

10.1.1.4 拖网采样船速

必须在 2 kn 左右。

10.1.1.5 种类鉴定计数

常见种必须给出种名,按种计数。

10.1.2 调查要素

包括测定生物量、栖息密度、种类组成、数量分布及其群落结构。

10.2 采样

10.2.1 采样设备

10.2.1.1 采泥器

10.2.1.1.1 抓斗式采泥器

水深小于 200 m 的海区一般使用采样面积为 0.1 m^2 的采泥器,水深大于 200 m 的海区使用采样面积为 0.25 m^2 的采泥器;港湾调查可酌用 0.05 m^2 的采泥器。

10.2.1.1.2 弹簧采泥器

采泥样面积为 0.1 m^2。

10.2.1.1.3 箱式采样器

采样体积为 500 mm×500 mm×500 mm(面积为 0.25 m^2)。另一种小型箱式采样器采样体积为 250 mm×250 mm×250 mm(面积为 0.063 m^2)。深海取样或取分层泥样时,应使用箱式采样器。

10.2.1.2 拖网

10.2.1.2.1 阿氏拖网

水深小于 200 m 的海区一般使用网口宽度为 1.5 m~2.0 m;港湾调查可用网口宽度为 0.7 m~1.0 m;大洋深海调查一般采用网口宽度为 2.5 m~3.0 m。

10.2.1.2.2 三角形拖网

网口大小及网衣结构同阿氏拖网。适合于沿岸水域和底质较复杂的海区采样。

10.2.1.2.3 桁拖网

一般适用于水深 100 m 以内的海区，特别是底质松软的海区。

10.2.1.2.4 双刃拖网

适于底质为岩礁、碎石或砂砾的海区。

10.2.1.2.5 深拖光学系统和深海底栖生物拖网

用深拖光学系统获得深海底栖生物录像和照相底片；用深海底栖生物拖网采集底栖生物标本。

10.2.1.3 绞车和吊杆

水深小于 200 m 的海区，一般用负荷 2 000 kg 的绞车和吊杆。绞车速度以 0.2 m/s～1 m/s 为宜。吊杆应高出船舷 5 m，舷间距 1 m。专用于采泥样的绞车及吊杆负荷为 1 000 kg。大洋深海采样；使用大型网具应按实际需要，配备液压绞车及龙门吊杆。

10.2.1.4 钢丝绳

一般拖网使用直径为 8 mm～10 mm 的软钢丝绳。采泥专用绞车，一般使用直径为 6 mm～8 mm 的软钢丝绳。大洋深海调查使用万米钢丝绳。

10.2.1.5 底栖动物漩涡分选装置

由筒体、漩涡发生器、分流器、支架和余渣收集盘组成，专供淘洗泥样及分选标本。

10.2.1.6 套筛

由三层不同孔径的筛子和支架组成，上层筛的孔径为 2.0 mm～5.0 mm，中层为 1.0 mm，下层为 0.5 mm。必须与漩涡分选装置配合使用。

10.2.2 海上采样

10.2.2.1 采泥

10.2.2.1.1 采泥器选择

采用面积为 0.05 m^2 的采泥器，每站采 5 个平行样品；采用 0.1 m^2 的采泥器，每站采 2 个～4 个平行样品；采用 0.25 m^2 的采泥器，每站采 1 个或 2 个(平行)样品。

10.2.2.1.2 泥样淘洗

采用漩涡分选装置淘洗时，泥样分批倒入筒体，应注意调节分流龙头开关至较大颗粒沉积物不致搅起溢出筒体。

10.2.2.2 拖网

10.2.2.2.1 投网

调查船航速在 2 kn 左右，航向稳定后投网。拖网绳长一般为水深的 3 倍，近岸浅水区应为水深3 倍以上，拖网时间为 15 min；水深 1 000 m 以上的深海，拖网绳长为水深的 1.5 倍～2.0 倍，拖网时间 30 min～1 h。

10.2.2.3 样品处理

10.2.2.3.1 采泥和拖网样品

应按类别、个体大小、柔软脆弱和坚硬带刺者分别装瓶。

10.2.2.3.2 定量采泥样品

应全部取回(包括余渣)。

10.2.2.3.3 定性拖网所获得的样品

数量过大时，可取总质量的一小部分称重，计算每个种的个体数，经换算得到总个体数。对于数量大且定名准确的种类，可保留一定数量供生物学等测定，其余计数和称重后可倾弃。称重和计数结果记录于表 H.30。

10.2.2.3.4 典型生态意义的标本

应拍照、观察并记录。

10.2.2.3.5 保存

a) 固定液

中性甲醛溶液、丙三醇乙醇溶液、甲醛乙醇混合液、布因(Bouinn)固定液、四氯四碘荧光素染色剂固定液(见附录 C);

b) 固定和保存

——采泥和拖网样品,应按类别使用不同的固定液。暂时性保存使用体积分数为5%～7%中性甲醛溶液,永久性保存应用体积分数为75%丙三醇乙醇溶液或体积分数为75%乙醇;

——大型藻类一般用体积分数为6%甲醛溶液保存;

——海绵动物先用体积分数为85%乙醇固定,后换以体积分数为75%乙醇加体积分数为5%丙三醇保存;

——腔肠动物、纽形动物、环节动物以及部分甲壳动物先以薄荷脑或硫酸镁麻醉,后换体积分数为5%中性甲醛溶液固定。纽形动物应用布因固定溶液固定12 h～24 h后,按顺序分别用体积分数为30%、50%、70%的乙醇浸洗至无色时止,最后用体积分数为70%的乙醇保存,供切片用;

——星虫类、虫益 虫类、腕足动物、软体动物、部分甲壳动物、棘皮动物和鱼类直接用体积分数为5%中性甲醛溶液固定。个体数较大的鱼类和头足类样品(0.25kg以上),应将体积分数为10%甲醛溶液注射入腹腔。棘皮动物的海胆,固定前应先刺破围口膜;

——余渣固定时,用四氯四碘荧光素染色剂固定液,便于室内标本挑拣;

c) 按上述固定的样品,超过两个月未能进行分离鉴定,应更换一次固定液。

10.2.2.3.6 记录

每站采样结束,应即填写表H.30,表中采泥和拖网样品总数,系指每站采得各类别生物分离后的瓶数和包数。记事栏记录该站工作情况。

10.2.2.3.7 填写标签

已装瓶的每号样品需投入标签。放入样品桶的样品,应先用纱布包装,并另加一个竹签。

10.3 样品分析

10.3.1 样品核对

每航次结束,应认真核对样品和采样记录是否相符。

10.3.2 样品编号

一般按调查采样站位先后,采泥和拖网序号等先后以代号编排(见附录C.2)。称重结果记录于表H.31。

10.3.3 样品登记

调查船返航后,必须及时处理采泥和拖网样品。按分类系统排列编号,并分别记录于表H.31和表H.32。每瓶样品(包括样品桶内的样品)应换以新编号的标签,并同时核对。

10.3.4 鉴定、计数

a) 鉴定时发现某号样品中出现两种以上,应即分开,另编新号,并及时填写相应记录表,同时投放鉴定标签;

b) 易断的纽虫、环节动物按头部计数;软体动物的死壳不计数;数量大时,可取其中一部分称重计数、换算。

10.3.5 测定生物量

a) 湿重生物量;

b) 管栖动物应剥去管子(小管可保留);寄居蟹应去螺壳称重;软体动物一般不去贝壳,但需吸尽壳表水分;

c) 个体大、数量多的软体动物的壳和肉分别称干、湿重;

d) 有孔虫、石珊瑚和部分钙质苔藓虫可不计重。

10.4 资料整理

10.4.1 定量泥样资料

10.4.1.1 计算

种类个体数和生物量分别换算为 ind/m^2 和 g/m^2。

10.4.1.2 数据汇总

各站所得种类或类群的个体密度和生物量列表统计。

10.4.1.3 栖息密度和生物量

按各类群生物所占密度和生物量百分数，用圆形图或柱状图或矩形图表示。

10.4.1.4 栖息密度和生物量分布图

a) 绘制总密度和总生物量分布图。绘制无脊椎动物重要门类（环节动物、软体动物、甲壳动物和棘皮动物四大类）的密度和生物量分布图；

b) 密度分布图一般以等值线或不同大小的圆圈表示，密度（ind/m^2）的取值标准：<5，10，25，50，100，250，500，1 000，>1 000；

c) 生物量分布图，一般以等值线或不同大小的圆圈表示，生物量（g/m^2）取值标准：1，5，10，25，50，100，250，500，1 000，>1 000。

10.4.1.5 种类分布表

鉴定后的定量样品，按分类系统记录于表 H.33。

10.4.1.6 主要种类分布图

选择对总密度和总生物量，或对各类群密度和生物量起决定作用和分布普遍的种类，绘制分布图。

10.4.2 拖网资料

10.4.2.1 种类分布表

鉴定后的定性样品，记录于表 H.34。

10.4.2.2 主要种类分布图

定性拖网主要种类分布图，绘制方法见 10.4.1.6。

10.4.3 种类名录

采泥和拖网的样品鉴定之后，按分类系统顺序，列出调查海区大型底栖生物种类名录。

10.4.4 填写报表

按本部分的有关规定填写报表。

11 小型底栖生物调查

11.1 技术要求和调查要素

11.1.1 技术要求

a) 从取样器取芯样，必须是未受扰动的采泥样品、未受扰动的标志是沉积物表面有一定深度的上覆水及取样器闭合严紧无任何撤漏；

b) 每站随机取芯样（或多管取样器样品 2 个，用于多元统计分析的重点站位取芯样应不少于 4 个）；

c) 栖息密度以 $ind/10\ cm^2$ 或 $10^6 ind/m^2$ 表示；

d) 生物量以（$\mu g \cdot dwt$）/$10\ cm^2$ 或（$g \cdot dwt$）/m^2 表示。

e) 干重生物量精密度±0.01 mg，当使用超微量分析天平（感量 0.1 μg）称重时应使用配套的水分 测定仪；

f) 条件许可时，应在近岸硬底、水深 40 m 以内且透明度较好的浅水区内，携带 SCUBA（配套的水下呼吸器）等装置进行潜水取样。

11.1.2 调查要素

小型底栖生物调查要素包括测定主要类群组成、栖息密度、生物量和优势类群的种类组成、群落结构和生物多样性。

11.2 采样

11.2.1 采样设备

11.2.1.1 采样器

见大型底栖生物调查。应依次选择各类箱式采样器和弹簧采泥器。条件允许时可采用多管采样器或潜水采样。

11.2.1.2 有机玻璃管

内径 2.2 cm(=3.8 cm^2)、2.6 cm(=5.3cm^2)、3.6 cm(=10 cm^2)和 4.4 cm(=15 cm^2),前两种适用于泥质和砂泥质,后两者适用于泥砂质和砂质。后一种还适用于轻潜水手持取样。

11.2.1.3 套筛网目

上层孔径为 0.5 mm,中层为 0.2 mm,下层为 0.042 mm。

11.2.1.4 撬式小型生物拖网

网衣孔径为 0.35 mm~0.45 mm,主要用于定性分析。

11.2.1.5 船上设备

同大型底栖动物调查。

11.2.1.6 潜水员和设备

有证潜水员至少 2 名。设备包括:面罩、呼吸管、调节器、浮力调节装置、潜水衣、潜水仪表、气瓶、压铅皮带、靴子和蛙鞋等(见附录 D 图 D.5)。配套船只:带舷外发动机,可承载 3 人~5 人。

11.2.2 海上采样

11.2.2.1 采芯样

潮间带取样应尽可能与大型底栖生物调查同步,即在选定的潮滩区,代表性断面,代表性站位(高潮、中潮和低潮)取样,每站按工作需要取芯样 2 个~4 个。泥质滩取芯样长度 8 cm~10 cm,划分为 0 cm~2 cm,2 cm~5 cm,>5 cm;泥砂质滩取芯样 12 cm~16 cm,划分为 0 cm~4 cm,4 cm~8 cm,8 cm~12 cm,12 cm~16 cm;砂质滩取芯样 24 cm~28 cm,每 4 cm 一层,砂质滩需借助橡皮锤敲击取样管顶部,以达到所需取样深度。取样管拔出之前,均需在管顶部加堵橡皮塞,一旦取样管离底,即用手封住下端。拔出时若发现芯样扰动应重新取样。

用有机玻璃管从箱式取样器中采芯样(再采样),芯样长度为 10 cm。采样位置必须离开取样器边缘至少 2 cm,随机采芯样两个,重点站位可分别在两个取样器中分别取 2 个或 3 个芯样。

11.2.2.2 潜水取样

条件许可时可进行潜水采样。由潜水员手持有机玻璃管直接取样,顶端和底端应分别加堵橡皮塞,方法同潮间带取样,样品用密封塑料袋盛装后提上水面。

11.2.2.3 小型生物拖网采样

操作程序与大型底栖生物拖网基本相同,拖网速度(可利用停机后的余速)应保持在 1 kn,拖网时间 5 min。

11.2.2.4 环境因子的测定

应至少包括沉积物粒度,含水量(%),总有机碳(%),Chl a 和 Phl a,用于粒度分析和有机碳分析的沉积物量不应少于 50 g,Chl a 和 Phl a 用 2.6 cm 内径的有机玻璃管取芯样 2 个,装入塑料袋后立即放入−20℃冰柜内保存。回到实验室应尽快测定。

11.2.3 样品处理

11.2.3.1 试剂

包括麻醉剂、固定剂和染色剂。

11.2.3.2 芯样

观察芯样颜色，记录“RPD”层(氧化还原电位不连续层)深度，将样品装于125 cm^3 或200 cm^3 广口塑料瓶中。

11.2.3.3 样品分层

现场取样时，取芯管一旦取样，立即按5 cm～10 cm，2 cm～5 cm和0 cm～2 cm，将样品分别推置于样品瓶内。

11.2.3.4 样品分装

拖网样品吊上甲板后，搅匀，取2个100 cm^3 的样品(沉积物、碎屑等)分别装入500 cm^3 的广口样品瓶中。

11.2.3.5 麻醉

定量采泥和拖网样品，均加入与样品等体积的麻醉剂，摇动静置10 min。

11.2.3.6 固定

麻醉后的采泥和拖网样品，均加入与样品等体积的固定剂固定。

11.2.3.7 填写标签

已装瓶的每号样品，需投入已填写好的标签(见附录D)。

11.2.3.8 记录

每站结束，应填写表H.35；记录的表格、标签和样品瓶号应严格核对。

11.2.3.9 活体样品

供活体观察的样品，不加麻醉剂和固定剂，装瓶后即放入冰箱冷藏保存。

11.3 样品分析

11.3.1 仪器设备

11.3.1.1 分离装置

分两层套筛，套筛直径为10 cm，搁放在相应直径稍大的800 cm^3 或1 000 cm^3 量杯上，上层网筛孔径为0.5 mm，下层为0.042 mm，若细砂颗粒过多，两层套筛的中间加一中层网筛，孔径为0.2 mm。

11.3.1.2 分离淘洗装置

用于砂质样品的分离(见附录D中的图D.2)。淘洗时应使用过滤海水。

11.3.1.3 台式离心机

转速5 000 r/min。

11.3.1.4 分样器

见附录D中的图D.1。

11.3.1.5 微量分析天平

感量为0.01 mg和0.000 1 mg两种。

11.3.2 砂质沉积物的分离(倾上浮液淘洗法)

a) 样品分离前加入四氯四碘荧光素染色剂，染色24 h以上。每100 cm^3 样品加入5 cm^3 染色剂溶液；
b) 移样品至1 dm^3 容量的广口杯内，加过滤海水至800 cm^3，加盖，颠倒摇动数次，静置1 min～3 min(视颗粒组成而定)；
c) 上浮液通过由两层套筛组成的分离网筛，以上重复淘洗三次；
d) 用洗瓶分别冲洗两层网筛上残留物至备好的计数培养皿中(见附录D)，供计数；
e) 砂质样品的连续淘洗法，见附录D。

11.3.3 泥质沉积物的分离(分层分离法)

a) 染色见11.3.2a)；
b) 倾样品至由三层套筛组成的分离装置上，用洗瓶冲洗至绝大部分较细粒级的颗粒被冲尽；

c) 用洗瓶分别冲洗三层网筛上的残留物至计数培养皿中供计数；

d) 动物数量过大时，应采用分样器取分样分选；

e) 分选用套筛，特别是最底层的网筛应定期在体视显微镜下检查，发现锈蚀或网孔堵塞或变形应立即更换网筛。

11.3.4 泥质沉积物分离的硅溶胶(Ludox-TM)离心漂浮法

a) 硅溶胶溶液的制备，取 2 份 Ludox-TM 加 3 份蒸馏水用比重计测试，将相对密度调至 1.15，备用；

b) 取 15 cm^3 的沉积物样品，置于 100 cm^3 的离心管中加 45 cm^3～60 cm^3 预先制备的硅溶胶溶液，加盖摇动充分混合；

c) 将离心管静置 5 min 以便较重的颗粒沉降；

d) 将成对离心管对称放置离心机中，关闭离心机盖，3 min 内加速至 1 800 r/min 维持 3 min；

e) 将含有生物的上悬液通过 0.042 mm 网筛，用蒸馏水彻底清洗将样品冲至计数皿中；

f) 余渣加同样份量的硅胶溶液，重复以上程序 2 次或 3 次。

11.3.5 取分样

a) 与沉积物分离后的样品，若动物数量太多（超过 500 个体）时，可随机取分样鉴定计数；

b) 将样品移入分样器，注入蒸馏水至 2 dm^3，加顶盖，颠倒摇动，静置 1 h，取分样 2 个或 3 个。

11.3.6 计数

a) 在高倍体视显微镜下(≥40×)观察，鉴定和计数，将不同类群的个体数分别记录于表 H.36、定性样品记录于表 H.38；

b) 对“软型”小型动物 如腹毛虫、涡虫、颚咽动物等，应尽量活体观察、鉴定和记数，对“硬型”小型动物如线虫、桡足类、介形类、动吻类等可制成临时性或永久性封片观察、鉴定和计数。

11.3.7 生物量测定

11.3.7.1 体积换算法

该法适用于小型动物各主要类群。取显微镜描图仪测量结果，换算体积：

$$V = L \cdot W^2 \cdot C \qquad (46)$$

式中：

V——体积，单位为 10 的负三次方立方毫米(10^{-3} mm^3)；

L——体长(长尾种类至锥状部，具丝状尾种类至肛门)，单位为毫米(mm)；

W——身体最大体宽，单位为毫米(mm)；

C——换算系数(不同类群的换算系数，见附录 D 和图 D.4)。

干重换算法：

$$d_w = V \cdot K \cdot D \qquad (47)$$

式中：

d_w——个体平均干重生物量，单位为微克(μg)；

V——个体体积，单位为 10 的负三次方立方毫米(10^{-3} mm^3)；

K——假定平均相对密度为 1.13；

D——假定干湿比为 0.25。

11.3.7.2 直接称重法

a) 随机取称样 2 份或 3 份，用重蒸水小心地冲洗，然后用吸管将样品置于微型铝箔（或微型秤皿)内。每份样品所需动物数量依类群而异，线虫 100 条～200 条，底栖桡足类 30 个～50 个，介形类 10 个～20 个，多毛类 10 条～20 条；

b) 将样品置于标准水分测定仪(红外加热或卤素加热)；

c) 线虫和桡足类样品分别置于感量 0.1 μg 超微量分析天平中称重 3 次，介形类和多毛类分别置

于感量 0.01 mg 微量分析天平中称重 3 次，记录于表 H.37。

每次称重应相应地称皿 3 次，将结果记录于表 H.37。

11.4 资料整理

11.4.1 精密度

a) 小型动物样品计数的精密度，以标准误差或置信度(95% C.L)表示；

b) 群落结构差异的统计检验，若取样前已存在某种零假设，则可根据几种试验设计类型做出检验。3 个重复样时，成对比较的显著水平最小不过 10%，4 个为 3%，5 个为 1%，若要在 5%的水平获得显著差异，一般至少需要 4 个重复样。

11.4.2 密度

11.4.2.1 密度计算

$$D = \frac{T}{\pi d^2} \times 10^4 \qquad \cdots\cdots(48)$$

式中：

D——个体密度，单位为个每平方米(ind/m^2)或 10 的六次方个每平方米(10^6 ind/m^2)；

T——重复芯样的个体平均数，单位为个(ind)；

d——取样管内径，单位为厘米(cm)。

11.4.2.2 密度空间分布

按表 H.36 要求，计算各站总密度，调查海区平均密度和年平均密度，并绘制密度等值线图。

11.4.2.3 密度垂直分布

按表 H.36 要求(0 cm～2 cm，2 cm～5 cm，5 cm～10 cm，>10 cm)计算各分层所占百分比，并相应计算各类群的百分比组成。

11.4.3 生物量

a) 生物量计算

$$B = \sum_{i=1}^{N} \overline{d}_w \cdot \overline{D}_i \qquad \cdots\cdots(49)$$

式中：

B——小型动物的总生物量单位为克每平方米或 10 的六次方微克每平方米(g/m^2 或 10^6 μg/m^2)；

$\overline{d}_w$—— 第 i 个种群的个体平均体重，单位为微克(μg)；

$\overline{D}_i$——第 i 个种群的个体平均密度，单位为个每平方米或 10 的六次方个每平方米(ind/m^2 或 10^6 ind/m^2)；

N——动物的类群数。

b) 根据以上分别计算各站总生物量，填写表 H.37。

11.4.4 填写报表

按本部分的有关规定填写报表。

11.4.5 绘图

a) 各类群密度百分组成图，密度平面分布和垂直分布图；

b) 主要类群生物量百分组成图，平面分布图和垂直分布图；

c) 有条件时可作多变量分析有关图，如聚类图，标序图等。

12 潮间带生物调查

12.1 技术要求和调查要素

12.1.1 技术要求

12.1.1.1 调查地点和断面的选择

a) 调查地点和断面选择必须根据调查目的而定。通常应选择具有代表性的、滩面底质类型相对

均匀、潮带较完整、无人为破坏或人为扰动较小且相对较稳定的地点或断面；

b) 在调查海区，选择不同生境（如泥滩、泥沙滩、沙滩和岩石岸）的潮间带断面（不少于3条断面），每条断面不少于5个站，岩石岸每个站不少于2个定量样方，泥滩、泥沙滩不少于4个定量样方，沙滩不少于8个样方。断面位置应有GPS定位或陆上标志，走向应与海岸垂直。

12.1.1.2 潮间带的划分

根据当地的潮汐水位参数或岸滩生物的垂直分布，将潮间带划分为高潮区、中潮区和低潮区，或高潮区：上层，下层；中潮区：上层，中层，下层；低潮区：上层，下层。（详见附录E）。

12.1.1.3 取样站布设

通常在高潮区布设2个站、中潮区布设3个站、低潮区1个站或2个站。在滩面较短的潮间带，在高潮区布设1个站、中潮区布设3个站、低潮区1个站。

12.1.1.4 调查时间

a) 潮间带生物采样必须在大潮期间进行；或在大潮期间进行低潮区取样，小潮期间再进行高、中潮区的取样；

b) 对于基础（背景）调查，通常按春季、夏季、秋季和冬季进行一年四个季度月调查。对于一些专项调查，根据要求可选择春、秋季两个季度月进行调查。

12.1.1.5 采样面积

硬相（岩石岸）生物取样，用25 cm×25 cm的定量框取2个样方；在生物密集区取样，采用10 cm×10 cm定量框取样。软相（泥滩、泥沙滩、沙滩）生物取样，用25 cm×25 cm×30 cm的定量框取4个样方～8个样方。同时进行定性取样与观察。定性取样在高潮区、中潮区和低潮区至少分别取1个样品。

12.1.2 调查要素

潮间带生物调查要素包括不同生境的种类组成、数量（栖息密度、生物量或现存量）及其水平分布和垂直分布。

12.2 采样

12.2.1 采样设备

12.2.1.1 采样器和定量框

泥、沙等底质类型的生物取样，采用滩涂定量采样框（见图E.4）。其结构包括框架和挡板两部分，均用1.5 mm～2.0 mm厚度的不锈钢板弯制而成，规格为25 cm×25 cm×30 cm。配套工具是平头铁锹。岩岸生物取样采用25 cm×25 cm的定量框。若在高密度生物量的潮区取样，可采用10 cm×10 cm定量框取样。计算覆盖面积，则用相应的计数框（见图E.3）。其框架可用镀锌铁皮或3 mm厚的塑料板制成。配套工具有小铁铲（或木工凿子）、刮刀和捞网。

12.2.1.2 漩涡分选装置和过筛器

a) 漩涡分选装置：该装置参见图E.5。用于潮间带滩涂调查的生物样品淘洗时，应配备有3.88 kW～7.35 kW的抽水机；

b) 过筛器：当漩涡分选装置无法使用时，或遇某些不宜采用该装置淘洗的样品，可直接采用过筛器（见图E.6）。筛网孔目1.0 mm。

12.2.2 样品采集

12.2.2.1 生物样品采集

a) 滩涂定量取样用定量框，样方数每站通常取4个～8个（合计0.25 m^2～0.5 m^2）。样方位置的确定可用标志绳索（每隔5 m或10 m有一标志）于站位两侧水平拉直，各样方位置要求严格取在标志绳索所标位置，无论该位置上生物多寡，均不能移位。取样时，先将取样器挡板插入框架凹槽，用臂力或脚力将其插入滩涂内；继而观察记录框内表面可见的生物及数量；然后，用铁锹清除挡板外侧的泥沙再拔去挡板，以便铲取框内样品。铲取样品时，若发现底层仍有生物存在，应将取样器再往下压，直至采不到生物为止，一般深度达30 cm。若需分层取样，可视底质

分层情况确定。

b) 岩石岸取样用 25 cm×25 cm 的定量框，每站取 2 个样方。若生物栖息密度很高，且分布较均匀，可采用 10 cm×10 cm 的定量框。确定样方位置应在宏观观察基础上选取能代表该潮区生物分布的特点。取样时，先将框内的易碎生物(如：牡蛎、藤壶等)计数，并观察记录优势种的覆盖面积。然后用小铁铲、凿子或刮刀将框内所有生物刮取净。

对某些栖息密度很低的潮间带生物，可采用 25 m^2 的大面积计数(个数或洞穴数)，并采集其中的部分个体，求平均个体重，再换算成单位面积的数量。

为全面反映各断面的种类组成和分布，在每站定量取样的同时，应尽可能将该站附近出现的动植物种类收集齐全，以作分析时参考，定性样品务必与定量样品分装，切勿混淆。

取样时，测量各潮区优势种的垂直分布高度和滩面宽度，描述生物分布带的特征。

12.2.2.2 水质和沉积物样品采集

12.2.2.2.1 水样采集

应在各断面调查的同时，于高平潮和低平潮时各采一次水样。河口区在两次采水期间内增加一次。岩沼和滩涂水洼内积水应另行采样。必要时，酌情对生物定量取样站穴内积水或底质间隙水采样分析。

12.2.2.2.2 沉积物取样

应与生物定量取样同步进行，取样站数依滩涂底质变化酌情而定。遇表、底层沉积类型有明显差异时；应分层取样，并记录其层、色、嗅味。其样品编号必须与该站生物定量样品编号一致。

12.2.3 样品的处理与保存

12.2.3.1 生物样品的淘洗

12.2.3.1.1 漩涡分选装置淘洗法

本法在小船上随着潮水上涨或退落进行操作，以减少样品搬运困难。若无船只可直接在滩涂上进行淘洗，分选装置和抽水机应附设防沉底板，并需考虑水源的充分供给。分选操作步骤如下：

a) 将该装置牢靠固定在小船(或滩涂)上，用消防水管连结装置和抽水机；

b) 启动抽水机，待装置的筒体内约注有 1/2 海水时，调节分流器水压使涡流适中，并及时倒入待淘洗样品；

c) 约经 10 min 涡动，大多数体轻、柔软的生物从出水口分选流出，截留于套筛(收集器)上。

d) 当进出筒体的水色相近时，即可打开装置的分流阀、关闭进水阀，并取一网筛(孔径 2 mm)置于筒体下，打开排渣阀排出余渣；

e) 将各套筛截留的余渣中生物挑拣干净。

12.2.3.1.2 过筛器淘洗法

当不具备使用漩涡分选装置时，可采用过筛器直接淘洗法。

12.2.3.2 生物样品的处理与保存

a) 采得的所有定量和定性标本，经洗净，按类别分开装瓶(或用封口塑料袋装)，或按大小及个体软硬分装，以防标本损坏；

b) 滩涂定量调查，未能及时处理的余渣，可只拣出肉眼可见的标本后把余渣另行装瓶(袋)，回实验室在双筒解剖镜下挑拣；

c) 谨防不同站或同一站的定量和定性标本混杂，务必按站或样方装瓶(袋)后，将写好的相应标签(见附录 E.4)分别投入各瓶(袋)中；

d) 按序加入体积分数为 5%左右的中性甲醛固定液。余渣固定时，用四氯四碘荧光素染色剂固定液，便于室内标本挑拣。固定液配制方法见附录 C.1；

e) 为便于标本鉴定，对一些受刺激易引起收缩或自切的种类(如：腔肠动物、纽形动物)，先用水合氯醛或乌来糖少许进行麻醉后再行固定；某些多毛类(如沙蚕科、吻沙蚕科)，先用淡水麻醉，再加固定液固定。藻类标本除用 5%中性甲醛固定的外，最好带回一些完整的新鲜藻体，制作腊

叶标本，以保持原色和长久保存。

12.3 样品分析

12.3.1 室内分析

12.3.1.1 标本整理

12.3.1.1.1 .核对

a) 按调查地点、断面、站号，将定量和定性标本分开；

b) 依野外记录，核对各站取得的标本瓶(袋)数。

12.3.1.1.2 分离、登记

a) 标本分离按断面或站进行，以免不同站(或不同样方)的标本混入。若有余渣带回，切勿遗忘将其中标本拣出归入；

b) 分离的标本经初步鉴定，以种为单位分装，并及时加入固定液。除海绵、苔藓虫等含钙质动物改用体积分数为75%酒精固定外，其余用体积分数为5%左右的中性甲醛保存；

c) 按分类系统依次排列、编号，用绘图墨水写好标签，标签上填写的除标本号和种名因分离可能改变外，其余各项均应与野外投放的标签一致。待墨汁干后，分投各标本瓶中；

d) 按新编序号分别将定量和定性标本登记于表 H.40 和表 H.41 中。

12.3.1.1.3 称重、计算

a) 定量标本须固定 3 d 以上方可称重，若标本分离时已有 3d 以上的固定时间，称重可与标本分离、登记同时进行；

b) 称重时，标本应先置吸水纸上吸干体表水分。称重软体动物和甲壳动物保留其外壳(必要时，对某些经济种或优势种可分别称其壳和肉重)。大型管栖多毛类的栖息管子、寄居蟹的栖息外壳以及其他生物体上的伪装物、附着物，称重时应予剔除；

c) 称重采用感量为 0.01 g 的药物天平、扭力天平或电子天平等。在称重前后计算各种生物的个体数(岩岸采集的易碎生物个体数由野外记录查得。群体仅用质量表示)；

d) 将称重、计数结果填入表 H.40 各相应栏目，并注明湿重(甲醛湿重或酒精湿重)、干重(烘或晒)。必要时可称取灰分重；

e) 依据取样面积，将记录表中各种数据换算为单位面积的栖息密度(ind/m^2)和生物量(g/m^2)。

12.3.1.2 标本鉴定

a) 优势种和主要类群的种类应力求鉴定到种，疑难者可请有关专家鉴定或先进行必要的特征描述，暂以 SP_1、SP_2、SP_3……表示，然后再行分析、鉴定；

b) 鉴定时若发现一瓶中有两种以上生物，应将其分出另编新号，注明标本原出处，并及时更改标签和表格中有关数据；

c) 种类鉴定结果若与原标签初定种名不符，亦应立即更改标签。

12.3.1.3 标本保存

经鉴定、登记后的标本，应按调查项目编号归类，妥善保存，以备检查和进一步研究。且须建立制度，定期检查、添加或更换固定液，以防标本干涸和霉变。

12.4 资料整理

12.4.1 野外采集记录表

a) 野外记录应有专人负责，填写表 H.39；绘制站位分布图；记录环境基本特征、生物分布、生物异常等现象；负责填写标签；

b) 各断面的生物带以及出现的生物异常、死亡、群落演替等现象，应用录像机或照相机拍录下来；

c) 野外记录是第一手资料，应用铅笔(或碳素墨水)填记，字迹须清晰，记录后妥善收存，严防受潮或丢失。

12.4.2 种类名录

根据表 H.40 和表 H.41 将每次采得的所有种类按分类系统依次列出，各物种标明中文名和拉丁名、采集时间、地点、断面、站号及分布潮区。

12.4.3 种类分析记录表

为了便于统计每个测站的种类及其数量，以站点为单位将每个种类的栖息密度和生物量汇总登记于表 H.40 和表 H.41 中。

12.4.4 种类分布表

为便于分析各种类时空分布特点，可依据表 H.40 记录，以种为单位，将其在各断面、各站位、各不同季节的栖息密度和生物量汇总登记于表 H.42 中。

12.4.5 主要种和优势种垂直分布表

为便于绘制主要种和优势种垂直分布图，将有代表性的、数量较大的种类的栖息密度和生物量按潮区、站位汇总于表 H.43。

12.4.6 主要类群统计表

根据本部分的有关规定，将种类名录，以断面或取样站为统计单位，计算各生物类群的种数和比率，填入表 H.44 中，表内类群名称可依不同底质类型增减。

12.4.7 填写报表

按本部分的有关规定填写报表。

13 污损生物调查

13.1 技术要求和调查要素

13.1.1 技术要求

a) 现场调查时，大型和微型污损生物试板回收力争完整齐全，且应保持试板生物标本完好；
b) 对船舶和其他海上设施进行污损生物调查时，要求代表性强且取样准确；
c) 大型污损生物样品分析时，要求优势种鉴定到种，湿重生物量的精确度为±0.01g。微型污损生物的优势种应鉴定到种。

13.1.2 调查要素

污损生物的调查主要为大型污损生物调查，调查要素包括种类、数量、附着期和季节变化、水平分布和垂直分布等。如有特殊需要时，应进行微型污损生物调查。

13.1.2.1 大型污损生物调查

应以试板调查为主，辅以船舶及其他海上设施调查。调查要素包括大型污损生物的种类、数量、附着期和季节变化。

13.1.2.2 微型污损生物调查

应采用载玻片进行，调查主要的微型污损生物的种类及数量。

13.2 采样

13.2.1 大型污损生物调查

13.2.1.1 港湾挂板调查

13.2.1.1.1 试板

a) 分月板、季板、半年板和年板。一律用 3 mm 厚的环氧酚醛玻璃布层压板，每片试板正中钻两个相距 50 mm，孔径 7 mm 的串板孔；
b) 每个挂板点放 1、2 两组板，每组两个水层，周年共需挂放和回收 76 片试板，具体数量和规格见表 8；

表 8　试板种类、规格及数量

板　别	月　板	季　板	半 年 板	年　板
规格/mm	3×80×140	3×80×145	3×80×150	3×80×150
数量/片	2×2×12=48	2×2×4=16	2×2×2=8	2×1×2=4

c）对于海岸港工建设的污损生物挂板调查则采用水泥试版，试板规格一律为 150 mm×150 mm×20 mm，每片水泥试板正中钻两个相距 50 mm，孔径 7 mm 的串板孔。

13.2.1.1.2　挂板点选择

a）首先了解挂板海区的水深、透明度、水温、盐度和海流等情况。挂板期间还必须有周年的月平均水温、盐度资料；

b）确定站位的原则是：有浮码头、浮筏、浮标或水产业的吊养绳缆等可供挂板；便于管理；水流畅通，水域开阔；

c）在一个港湾或一段近岸水域，通常只需设一个挂板点。河口区等环境变化大的水域，可增设一个或几个点，且必须兼顾不同盐度梯度或水流畅通程序不同的点。

13.2.1.1.3　挂板周期、时间及层次

a）周期和时间：每点挂板一周年。分月板、季板、半年板和年板，从 3 月 1 日开始，同时挂放，并按时回收和更换新板。3 月～5 月，6 月～8 月，9 月～11 月和 12 月～翌年 2 月分别代表春、夏、秋、冬四个季度。3 月～8 月和 9 月～翌年 2 月分别代表上半年和下半年；

b）层次：每组板都分表层和中层两个水层。表层板的上缘正好露出水面，中层板离水面 2.0 m。大潮期间低潮时的水深仍大于 5 m 的水域，可在近海底 0.5 m 处增挂底层板。

13.2.1.1.4　放板和取板

在每月的头三天取、放试板。挂放在水中的试板表面应与水面垂直。从水中取出的试板应在现场包于纱布中，并系以标签，然后固定在体积分数为 5%～8%的中性甲醛溶液中。

13.2.1.2　港湾以外海区挂板调查

13.2.1.2.1　站位

根据离岸远近布站。

13.2.1.2.2　水层

分表层（离海面 2 m），10 m，25 m，50 m，100 m，150 m，200 m……和底层（离海底 5 m）。

13.2.1.2.3　挂板时间和周期

6 月上旬挂板，历时一周年取板。视需要可酌情增加季板。

13.2.1.2.4　试板

试板材料为环氧酚醛玻璃布层压板，规格 3 mm×200 mm×300 mm。

13.2.1.2.5　挂板和取板

挂于特制浮标或潜标上的试板，每个水层挂两片。取板时，必须在现场将试板装入纱布袋并浸于固定液中。

13.2.1.3　船舶及其他海中设施调查

13.2.1.3.1　取样要求

取样前必须现场拍照或录像，现场测量厚度和覆盖面积率。根据不同对象，填写调查记录表。取样面积根据生物的多少酌定，一般是 20 cm×20 cm 和 30 cm×30 cm。

13.2.1.3.2　取样位置

按下列规定实施：

a）船舶：取样位置包括水线、船首侧面、船体底部、船尾底部、舵和螺旋桨等六个位置，并记录于表 H.46；

b） 浮标：取样位置包括浮筒的水线、侧面、底部、尾部、尾内和沉块，并记录于表 H.47；
c） 浮筏及浮码头：取样位置包括水线、侧面和底部；
d） 码头桩柱：取样位置包括潮间带的高、中、低三个潮区和大潮低潮线下 1 m 的潮下区，必须测量污损生物群落的垂直分布和分带；观察和测量优势种的垂直分布上界和下界，并记录于附录表 H.48；
e） 海中平台：根据石油平台的类型和所在海区，酌情取样；在条件许可时，选择有代表性的桩腿表面，每隔 5 cm 潜水取一样品，直至海底，取样前先进行水下录像；
f） 遥测浮标、潜标及水下仪器仪表：取样位置必须注意不同水层和不同部位的代表性；
g） 海底的声纳外壳、沉船及海底电缆：必须同时在顶部和近海底部位、暴露部位和隐蔽部位取样；
h） 冷却水管道系统：取样位置包括管道口外的过滤栅、过滤鼓、及离管道口不同距离的管道内壁；
i） 渔业设施：包括定置网具、养殖网箱和网笼、浮筏和浮球、人工鱼礁等。

13.2.2 微型污损生物调查

13.2.2.1 试板

用体积分数为 70％乙醇浸泡的载玻片（25 mm×75 mm）。

13.2.2.2 挂板

13.2.2.2.1 方式和层次

垂直悬挂在浮体上。分三层，表层距水面 0.5 m～1.0 m；底层离海底 1 m；中层在表、底层的中间。

13.2.2.2.2 时间和周期

每季度一次。分别在 2 月、5 月、8 月和 11 月上旬挂放。挂放周期，分别为 1 h、4 h、8 h、16 h 和1 d、3 d、7 d、14 d、21 d、28 d。

13.2.2.3 取板

每次取三片。在海水中操作。经现场海水轻漂洗后装入盛有无菌海水的容器内。用于宏观检查、硅藻鉴定和计数的试板，以体积分数为 3％的戊二醛固定；用于细菌分离和鉴定的试板，则及时送实验室。

13.3 样品分析

13.3.1 大型污损生物

13.3.1.1 分析要素

大型污损生物的样品分析包括以下五种要素，其中前三者为必测要素，后二者可酌定。

13.3.1.1.1 种类

含种类数和种名，优势种鉴定到种。

13.3.1.1.2 数量

数量包含五个指标，按下列规定实施：

a） 厚度：测量试板的四个角落和中央五个点的平均厚度；某些藻类、水螅和草苔虫应拉直测量；群落中厚度相差悬殊者，加测最大厚度；
b） 覆盖面积率：整个群落覆盖附着基的百分比率；
c） 附着面积率：每种污损生物附着基底总面积与最底层附着基面积的百分比率；草苔虫、水螅和藻类以在水中实际覆盖面积计算；
d） 密度：逐种记录单位面积的个体数；海藻、海绵、水螅、苔藓虫和复海鞘等以片、块或丛计；
e） 湿重：吸去外表水分后的质量，精密度为±0.01 g。

13.3.1.1.3 附着期和季节变化

根据试板上的种类和数量，确定主要种类的附着期、附着强度及其季度变化。

13.3.1.1.4 水平分布

应注意离岸距离、盐度梯度、流系及水流畅通程度等生态因子与污损生物分布的关系。

13.3.1.1.5 垂直分布

必须有表层和接近海底的数据。

13.3.1.2 试板分析

分析前先拍照。港湾试板和港湾外试板的分析面积，分别为200 cm^2 和500 cm^2。依次测量群落的平均厚度、最大厚度和覆盖面积率。逐种计量密度、附着面积率和湿重。然后把标本刮下，按种分装，填写标签，将结果记录于表 H.49。

13.3.1.3 船舶及其他海中设施样品分析

逐种分别计量个数和湿重。将结果记录于表 H.49。

13.3.1.4 种类鉴定

种类定名后，按标本号记录于表 H.49。

13.3.2 微型污损生物

13.3.2.1 观察

肉眼观察、显微镜观察和进一步作扫描电镜观察。

13.3.2.2 测定干重

在温度为 105℃的烘箱中烘干至恒重，称重。

13.3.2.3 硅藻计数和鉴定

将试板置于显微镜下直接计数或将硅藻刮下后计数。经制片后进行种类鉴定，优势种鉴定到种。

13.3.2.4 四大菌类计数

13.3.2.4.1 预处理

取板后必须立即进行预处理，并于 24 h 内完成。制成菌悬浮液供计数。

13.3.2.4.2 计数

按下列步骤实施：

a) 平板计数

将菌悬浮液分别涂布于细菌、真菌、酵母和放线菌的培养基上(培养基见 6 微生物调查)，经 25℃恒温培养 4～7 d，计算菌落数并记录于表 H.45，同时挑取不同菌落特征的纯菌落至相应的培养基上，经 25℃培养见 4～7 d后，供菌株鉴定。方法详见 6.3.4.1.2.2；

b) 附着细菌的荧光显微计数

见 6.3.4.1.1，必须经分散均匀后，方可计数。

13.3.2.5 其他微型生物计数和鉴定

包括微型海藻、原生动物和线虫等。

13.4 资料整理

13.4.1 大型污损生物

13.4.1.1 种类

按表 H.50 逐种填写，并编制种类名录及其出现频率，确定优势种。

13.4.1.2 数量

整理逐月、逐季、半年和年度的附着厚度、覆盖面积率、附着面积率和湿重及各大类湿重的百分比。

13.4.1.3 附着期

绘制主要种类的附着期、附着强度和季节变化图。

13.4.1.4 水平分布

在有港湾和外海资料的情况下，可根据离岸距离、盐度梯度、流速及水流通畅程度，总结出污损生物的水平分布及其与环境因子的相关性。

13.4.2 微型污损生物

a) 分析对比各附着期内不同类型群落的宏观特征；

b) 编制各附着期内种属名录及其优势种类名录；

c) 绘制细菌、硅藻的消长曲线；

d) 绘制干重变化曲线。

13.4.3 填写报表

按本部分的有关规定填写报表。

14 游泳动物调查

14.1 技术要求和调查要素

14.1.1 技术要求

14.1.1.1 调查类型

根据调查目的，游泳生物调查可划分为以下调查类型：

a) 专题性大面定点调查

为某一特定目的而进行的调查。

b) 资源监测性调查

为对某一种或多种渔业资源进行定期性或非定期性的定点调查和非定点性的调查和探捕。

c) 渔业资源声学调查与评估(选做。见附录G)

在前两类调查中，如果调查船具备声学调查与评估的功能，也可采用声学调查和试捕取样相结合的方法进行。渔业资源声学调查与评估技术见附录G。

14.1.1.2 调查时间和调查范围

应根据调查对象群体的不同生活阶段(产卵、索饵、越冬)确定调查时间和调查范围，并以此作为调查计划设计的依据。根据调查目的、鱼类洄游规律和海底地形地貌等环境条件确定调查断面和站位。

14.1.1.2.1 调查计划的设计

通常包括调查的目的意义，调查时间(含航次安排)，调查海区和范围，调查船只(含网具的类型和规格)，调查要素和方法以及注意事项等。站位设计、航线设计和调查计划设计应按以下规定进行。

a) 定点调查站位的设计

——调查水深小于200 m的大陆架海区

通常应采用网格状均匀定点法，可根据不同的调查目的按经度、纬度各15′～60′的距离布站。也可选择通过不同的主要渔场、不同的资源密度分布区、或不同的等深线分布区设置断面定点站位。但遇到有障碍物或海底严重凹凸不平的地方，应适当移动站位位置。

——调查水深大于200 m的大陆架斜坡、深海或大洋洋区

设站的方法同水深小于200 m的大陆架海区所用方法，但站位的距离可适当放大。

b) 航线设计

在保证达到调查目的的前提下，航线应遵循安全和经济两个原则，在保证安全的条件下要选取顺风、顺流航距最短的经济航线。

c) 定期性或非定期性资源监测调查计划的设计

根据监测对象的洄游规律，划定监测调查的海区范围(一个至数个区域)，确定每块区域探捕调查的天数，由监测船船长决定放网和起网的地点和时间，每网按发给的表格做好渔获物的登记，测定海水表温，同时按要求留取生物学测定样品，并在航次的最后一网随机留取样品鱼，连同其他生物学测定样品带给研究单位分析测定，研究单位定期(1个月至3个月)把监测调查的结果撰写成简报发送有关单位参考。

14.1.1.2.2 拖网时间和拖网速度

定点站位每站拖网时间为 1 h，拖速应根椐调查对象游泳能力的强弱和调查船的性能综合考虑，调查中小型底层鱼类以 2 kn～3 kn 为宜，调查游泳能力强的大型底层鱼类(鳕鱼等)和中上层鱼类以 3 kn～4 kn 左右为宜。

14.1.1.2.3 调查时间

对于白天贴底的鱼类如带鱼、鲳鱼等应安排在白天调查，对于夜里贴底的鱼类如马面鲀和虾类等应安排在夜间调查。如果日、夜均调查，应做昼、夜间渔获率的对照试验，求算出昼、夜网的修正系数。

14.1.1.3 调查网具

应选取选择性能小的网具作为调查网具。

14.1.1.4 渔获物分析

分析渔获物的组成和生物学测定样品一定要按随机取样的原则进行。

14.1.2 调查要素

调查要素包括游泳动物的种类组成、数量分布、群体组成，生物学和生态学特征及其时空变化等。

14.2 采样

14.2.1 主要工具与设备

14.2.1.1 调查船

14.2.1.1.1 调查船性能

调查船应由专业调查船承担，或选择适于在调查海区(沿海、近海、外海和远洋)作业且设备条件良好的渔船承担。

在同一个项目的专项调查或同种对象的资源监测调查中，如果由一对(艘)以上的调查船承担时，调查船的功率和性能要相同或基本相同，如果有明显差异，要作对照试验，求出不同单位调查船之间的修正系数。

14.2.1.1.2 调查船的主要仪器和设备

应具备能在调查海区中定位的卫星定位仪、能在调查海区与陆地基地联络的通讯设备，性能良好的探鱼仪和雷达，能随时观察曳网情况的网位仪，与调查水深和调查网具相匹配的起网机和起吊设备，具备渔获物样品冷藏库、冷冻库或超低温冷冻库(金枪鱼)等。专业调查船应具备生物学和生态学实验室，如进行声学调查，应具备声学仪器室。

14.2.1.2 调查取样网具

调查网具包括调查专用底层拖网（见附录 G，图 G.1)、调查专用变水层拖网(见附录 G，图 G.2)、双船底层有翼单囊 A 型拖网和 B 型拖网(见附录 F，图 F.1 和图 F.2)、以及单船有翼单囊拖网(见附录 F，图 F.3)。如同时需要了解渔获物幼鱼数量时，双船底层有翼单囊 A 型拖网、B 型拖网和单船有翼单囊拖网的囊网需加目大 20 mm 左右的套网。在特殊海域或对特定调查对象调查时，应因地制宜地选择最适网具，并保持相对稳定。

调查船必须备有备用网具和充足的渔具属具。

14.2.1.3 调查主要工具和设备的可比性

在同一项目调查和资源监测性调查中，应注意保持调查船性能和调查网具的性能和规格的一致性，如果有明显的变动，应做对照实验，求出差异系数，以确保调查资料有良好的可比性。

14.2.2 操作程序

14.2.2.1 拖网采样

14.2.2.1.1 放网

放网的位置要综合拖速、拖向、流向、流速、风向和风速等多种因素，在距标准站位位置 2 n mile～4 n mile时放网，经 1 h 拖网后正好到达标准站位位置或附近。

临放网前要准确测定船位，放网时间以停止曳纲投放，曳纲着底开始受力时为准。

14.2.2.1.2 拖网

拖网中要尽可能保持拖网方向朝着标准站位，记录鱼群映象出现的水层、经、纬度和拖网速度的改变情况，要注意周围船只动态和调查船的拖网是否正常等，若出现不正常拖网时，应视其情况改变拖向或立即起网。

14.2.2.1.3 起网

临起网前必须准确测定船位，起网过程中两船的卷网速度要一致，起网时间以起网机开始卷收曳纲的时间为准。如遇严重破网等重大渔捞事故导致渔获物大量减少时，应重新拖网。

14.2.2.1.4 记录各项渔捞要素

必须把每站渔捞要素记录在表 H.51。

14.2.3 样品处理

14.2.3.1 渔获物样品处理

14.2.3.1.1 估计站位渔获物总质量

把囊网里的全部渔获物倒在甲板上，记录估计的网次总质量(kg)，如果囊网外有加套网的，套网里的渔获物要另行保存和分析测定。

14.2.3.1.2 留取渔获物分析样品

渔获物总质量在 30 kg～40 kg 以下时，全部取样分析，大于 40 kg 时，从中挑出大型的和稀有的标本后，从渔获物中随机取出渔获物分析样品 20 kg 左右，然后把余下的渔获物按品种和不同规格装箱，记录该站次准确渔获总质量(kg)，并从其中再留取特殊需要的样品，如不同体长组的年龄、胃含物和怀卵量的样品等。

样品如不在现场分析，应装箱(袋)扎好标签，做好记录，核对无误后及时冰鲜或速冻或浸制。如是小型标本要装好瓶子放好标签，用体积分数 5%的甲醛或工业酒精固定。

14.3 样品分析

14.3.1 主要仪器设备

解剖镜(体视显微镜)、显微镜、电子秤、提秤、台秤、天平(感量 0.1 g,0.01 g 和 0.001 g)各一台(杆)、量鱼板(长度 500 mm，每格 1 mm)、解剖刀和卷尺，并具备用品件。

14.3.2 核对样品

每航次调查结束时要认真核对保存的样品和记录是否相符。

14.3.3 渔获物样品分析

渔获物样品分析必须鉴定到种，记录各种类的名称、样品质量、尾数，样品中最小、最大体长(肛长、胴长或全长等，mm)和最小、最大体重(g)。把样品分析的结果记录在表 H.51。对调查目标鱼种、主要经济鱼种和渔获物优势种随机留出生物学测定样品 35 ind～110 ind，少于 30 ind 的全测。

14.3.4 生物学测定

生物学测定按种类进行，测定前将样品洗净、沥干，逐尾排列、编号，依次进行各项测定，少于30 ind 的全部测定，长度以 mm 为单位、质量以 g 为单位，测定数据记录于表 H.52～表 H.55。

14.3.4.1 鱼类

14.3.4.1.1 长度

按鱼种选测：

a) 全长

自吻端至尾鳍末端的长度。鳗类和犀鳕类等以全长代表鱼体长度，其他鱼类以全长为辅助观测项目，测定数据记录于表 H.52；

b) 体长

自吻端至尾椎骨末端的长度。尾椎骨末端易于观察的石首鱼科、鲷科、鲆科、鲽科等以体长代表鱼体长度；

c) 叉长

自吻端至尾叉的长度。马鲛鱼、鲳鱼、鲐鱼(Scomber japonicus)、鲹鱼和鳓鱼、黄鲫等鲱科鱼类及其他尾叉明显的鱼类以叉长代表鱼体长度；

d) 肛长

自吻端至肛门前缘的长度。尾鳍、尾椎骨不易测量的海鳗、带鱼和鲨鱼等以肛长代表鱼体长度；

e) 体盘长

自吻端至胸鳍后基的长度。胸鳍扩大与头相连构成体盘的鳐属、魟属等以体盘长代表鱼体长度。

以上长度资料也可用腊纸刺孔保存，把样品按雌雄和性腺成熟度分堆放好，在腊纸上依次(分不同行)刺孔。腊纸上要记录种名，捕捞时间、地点，腊纸起点长度和刺孔样品总质量或各性腺成熟期的样品质量等。

14.3.4.1.2 **体重**

a) 体重：鱼体的总质量；

b) 纯体重：除去性腺、胃、肠、心、肝、鳔等内脏及体腔内脂肪层的鱼体质量。

14.3.4.1.3 **年龄样品**

a) 对于尚未掌握年轮形成时间的鱼种，必需周年(每月一次)采集样品，每月样品应有从小至大不同体长组的样品，每个体长组要有 10ind～30ind 样品。对于只为了解渔获物的年龄组成的应从网次渔获物中随机取样；

b) 测定不同鱼类的年龄，往往使用不同的年龄介质，经常采用的年龄介质有鳞片、耳石、鳍条硬棘、脊椎骨和匙骨等数种。

——鳞片：以鳞片为主测定年龄的鱼种有鳓鱼、黄鲫、蓝圆鲹等。采鳞片前应除去浮鳞，取鱼体第 1 背鳍前部下方至侧线上方或鱼体中部一定部位的鳞片 10 枚～20 枚，若该处鳞片脱落，可取胸鳍覆盖处的鳞片，洗净后放入该鱼种编号袋中保存；

——耳石：以耳石为主测定年龄的鱼类有小黄鱼、大黄鱼、白姑鱼、带鱼、鲐鱼、马鲛鱼等。切开颅顶骨或翻开鳃盖，切开听囊，取出一对耳石，洗净后放入该鱼种编号袋中保存；

——脊椎骨：以脊椎骨为主测定年龄的鱼种有绿鳍马面鲀、黄鳍马面鲀等。取基枕骨后的脊椎骨 10 节左右，除去附骨和肌肉，写上标签按测定的编号顺序以细绳栓好，阴干保存。

14.3.4.1.4 **性腺成熟度与怀卵量样品**

工作程序如下：

a) 区分性别，剖开鱼体胸、腹腔，按性腺鉴别雌(♀)与雄(♂)，不能分辨雌雄者，记为雌雄不分(⚥)；

b) 性腺成熟度，一般采用目测法，根据性腺不同发育阶段的外部形态特征，将性腺成熟度划分为六期(见附录 F.1.1)，将目测结果记录于表 H.52；称重法，即性腺成熟系数，它是性腺质量占纯体重的千分数。称量卵巢和精巢质量的最大误差不得大于±0.2 g；

c) 怀卵量，它是雌性成熟个体卵巢中持有的卵粒数量。每次按不同鱼体长度组收集 4 期的卵巢标本 10 个，放入具有种名、编号、采样时间和站号标签的瓶中，用体积分数为 5% 甲醛溶液固定。

14.3.4.1.5 **摄食强度和消化道样品**

工作程序如下：

a) 摄食强度，目测法：根据胃内食物充满情况，摄食强度划分为五级(见附录 F.2.1)；称重法：称消化道内食物质量，供计算其占鱼体纯体重的千分数——饱满系数；

b) 消化道样品，每次取胃肠样品 50 个，放入具有种名、编号、采样时间和站号标签的瓶中，用体积

分数为5%甲醛溶液固定。

14.3.4.1.6 含脂量

a) 含脂量的测定主要用于中上层鱼类；

b) 含脂量以目测法观测，分为4级(见附录F.3)。测定结果记录于表H.52。

14.3.4.2 虾类

14.3.4.2.1 性别和性比

对虾类根据交接器、真虾类根据生殖孔的位置分辨雌与雄，记录于表H.53，并统计其比例。

14.3.4.2.2 长度、质量

a) 头胸甲长：眼窝后缘至头胸甲后缘的长度；

b) 体长：眼窝后缘至尾节末端的长度；

c) 体重：虾体总质量。

14.3.4.2.3 交配率

在虾类交配季节，计算已交配雌虾所占的百分比：

a) 对虾类：已交配的雌虾，交接器内充满乳白色精液；

b) 真虾类：抱卵的雌虾即为已交配。

14.3.4.2.4 性腺成熟度

剪开雌虾头胸甲，对虾类和毛虾的性腺成熟度均分为5期(见附录F.1.2和附录F.1.3)。

14.3.4.2.5 摄食强度和胃含物样品

a) 摄食强度：按胃含物的多少，分为4级(见附录F.2.2)；

b) 胃含物样品：每次取虾类头胸部或胃50个，放入注有种名、编号、采样时间和站号的标签，用体积分数为5%的甲醛溶液固定。

14.3.4.3 蟹类

14.3.4.3.1 性别和性比

按腹部形状区分雌、雄，记录于表H.54，并计算其百分比。

14.3.4.3.2 头胸甲长度和宽度

a) 头胸甲长：从头胸甲的中央刺前端至头胸甲后缘的垂直距离；

b) 头胸甲宽：头胸甲两侧刺之间的距离(必测要素)；

c) 腹部长：腹部弯折处至尾节末端的垂直距离；

d) 腹部宽：第五、第六腹节间缝的长度。

14.3.4.3.3 体重

蟹体总质量。测定数据记录于表H.54。

14.3.4.3.4 性腺成熟度

以梭子蟹为例，性腺成熟度分为6期(见附录F.1.4)。

14.3.4.3.5 交配率

雌性幼蟹首次交配后，腹部由三角形变为椭圆形，体内的两个储精囊内各有一个精荚。

14.3.4.3.6 摄食强度和胃含物样品

同虾类(见14.3.4.2.5)。

14.3.4.4 头足类

14.3.4.4.1 性别和性比

a) 头足类的雄性个体具有茎化腕，其功能是在交配期把精荚传递给雌体。不同种类茎化腕的形态不一样：乌贼类的茎化部分为吸盘骤然变小或消失；柔鱼和枪乌贼类的茎化部分为吸盘变为肉突状；蛸类的茎化部分为在茎化腕的顶部形成端器，端器由交接基、精沟和舌叶组成；

b) 乌贼类的茎化腕为左侧第 4 腕;柔鱼类的茎化腕多数种类为右侧第 4 腕,少数种为左侧第 4 腕或第 4 对腕,其中北太平洋产的柔鱼(巴特柔鱼),幼体时茎化腕的茎化部分很短,随着个体的增大才逐步增长;枪乌贼类为左侧第 4 腕;蛸类为右侧第 3 腕。观察结果记录于表 H.55,并计算其百分比。

14.3.4.4.2 胴体长度

a) 以胴体背部中线的长度为胴体长度;

b) 无针乌贼,自胴体前端至后缘凹陷处;

c) 有针乌贼,自胴体的前端至螵蛸的后端;

d) 柔鱼和枪乌贼,自胴体的前端至胴体末端;

e) 蛸类,不测胴体长度。

14.3.4.4.3 体重和纯体重

a) 体重:头足类个体总质量;

b) 纯体重:除去性腺、胃、肠、心、肝、鳃、墨囊、盲囊等内脏的个体质量。

14.3.4.4.4 性腺成熟度

乌贼、枪乌贼和柔鱼类的性腺成熟度均分为 6 期(见附录 F.1.5、附录 F.1.6),茎柔鱼的性腺成熟度分为 5 期(见附录 F.1.7),真蛸的性腺成熟度分为 3 期(见附录 F.1.8)。

14.3.4.4.5 摄食强度和胃含物样品

同鱼类(见 14.3.4.1.5)。

14.4 资料整理

14.4.1 拖网卡片

14.4.1.1 计算各站次和各航次渔获物种类组成

首先把留取部份样品的种类(含非游泳动物种类)质量和尾数换算成该站次总渔获量的质量和尾数,然后计算各站次渔获物种类每小时的质量(kg/h)和尾数(ind/h)及其百分比,把计算结果记录于表 H.51中。把鱼、虾、蟹类和头足类按其分类系统的顺序列出种名(学名),分别记录于表 H.56,统计该航次(月份或季度或全年)的种类组成,如表 H.56 所示。

14.4.1.2 绘制各站总渔获量和主要种类数量分布图

一般以不同大小的实心圆、或含有不同图案的圆圈表示。取值标准可由电脑自动分级,也可根据数值的分布状况人为分级。图示的单位一般有 kg/h 和 ind/h 两类。

14.4.1.3 绘制各航次(月份或季度或年份)调查的游泳动物种类组成和数量的百分比图

一般以圆圈图案或柱形图表示,图示单位为百分比(%)。

14.4.2 生物学测定资料

按雌、雄分别整理,测定尾数不多时,可合并整理。

14.4.2.1 长度组成

14.4.2.1.1 各站次长度组成

将每次测定的个体长度资料按长度组整理,统计其分布频数、频率,最小和最大体长,优势体长组的范围和比例,求算平均长度。鱼类的体长组一般均以 10 mm 为一组距。幼鱼、虾类等个体小的,可以 5 mm或 2 mm 为一组距。若遇正好落在体长组端点上时归为上一组。

14.4.2.1.2 不同渔场、海区、时间的体长组成

按不同的渔场、海区、月份、季度等统计其长度组成,统计要素同 14.4.2.1.1。

14.4.2.2 质量组成

要素和方法与长度组成相同。体重组的组距视体重分布的范围具体确定。

14.4.2.3 年龄鉴定和统计

a) 根据所采的各鱼种鳞片、或耳石、或脊椎骨、或硬棘鳍条鉴定年龄,记录于表 H.52;

b) 年龄归组，在年轮形成后到同年 12 月底，以年轮数代表年龄，记为 0、1、2、3、4……n。从下年 1 月开始到新轮出现前则以年轮数加“+”号表示年龄，记为 0^+、1^+、2^+、3^+、4^+……n^+。归组时将 0^+ 和 1、1^+ 和 2、……n^+ 和 $n+1$ 归入同年龄组；

c) 统计每次样品中各龄鱼在各体长组和体重组中的分布及其占总尾数的比例，其结果记录于表 H.52和表 H.57、表 H.58。计算各年龄组的平均长度和平均质量。

14.4.2.4 性腺资料分析

a) 分别统计雌、雄鱼尾数，计算其百分比；

b) 统计雌、雄鱼性腺成熟度各期尾数，计算其所占的百分比；

计算性腺成熟系数，计算公式：

$$K_m = \frac{W_s}{W_p} \times 1\,000 \qquad \cdots\cdots(50)$$

式中：

K_m——性腺成熟系数，数值以 10^{-3} 表示；

W_s——性腺质量，单位为克(g)；

W_p——鱼体纯体重，单位为克(g)。

性腺性成熟系数的计算结果记录于表 H.52。

虾、蟹及头足类等用以上方法统计计算，其结果记录于表 H.53 至表 H.55。

c) 计数怀卵量，将保存的卵巢样品吸干外表的水分，用感量 0.01 g 的天平称总质量，然后将卵巢中的卵粒充分混合后，用感量 0.001 g 的天平称出 0.2 g～1 g 的卵子(视卵粒大小而定)，取双样计数，误差为±5%。计算卵子总数量(怀卵量)，并记录于表 H.59。

14.4.2.5 摄食强度和胃含物分析

a) 按雌、雄分别统计各摄食等级的尾数，计算其百分比；

按雌、雄鱼分别计算每尾鱼的饱满系数，计算公式：

$$K_f = \frac{W_e}{W_p} \times 1\,000 \qquad \cdots\cdots(51)$$

式中：

K_f——饱满系数，单位为 10 的负三次方(10^{-3})；

W_e——消化道内食物质量，单位为克(g)；

W_p——鱼体纯体重，单位为克(g)。

饱满系数的计算结果记录于表 H.52 至表 H.55。

b) 分析胃含物，将胃含物样品吸去水分，用感量 0.01 的天平称总质量。计数胃含物中各种饵料生物的个数，并分别称重。对鉴别出的各种饵料生物，按个数和各种成分的质量计算其百分比；

c) 绘制饵料生物个数和质量的组成图。

14.4.3 撰写调查航次小结或监测调查报告

a) 调查航次小结的主要内容为：调查目的意义；调查时间；调查海区范围；调查船只；调查网具类型和规格；参加调查的主要科研人员和船长等；调查执行情况；调查取得的主要结果；存在的主要问题和建议等。附实际调查站位和航线图、总渔获量和主要渔获种类渔获量分布图等；

b) 撰写调查总结报告。

14.4.4 填写报表

按本部分的有关规定填写报表。

附 录 A
（规范性附录）
微生物最可能数（MPN）计数及其检索表

A.1 微生物最可能数（MPN）计数法

适用于测定特殊生理种群的微生物数量，如大肠菌群、粪链球菌、弧菌、硝化细菌等。

A.1.1 培养液

根据对象菌的生理特征，采用相应选择性培养基，定量分装于试管。

A.1.2 样品

将样品制成10倍浓度关系的梯度系列。

A.1.3 接种

选择三个适当的连续梯度浓度，如样品中菌数少时，可选取1 cm^3，10 cm^3，100 cm^3 原液（后两种原液必须经孔径0.2 μm滤膜浓缩）。然后移入装有培养基的各试管，每个浓度必须加5管。加样量不得过度稀释培养液。

A.1.4 培养

根据对象菌的特征，确定培养温度和时间。

A.1.5 计数

与对照管比较，准确判别和记录各浓度梯度出现阳性的管数，从表A.1最可能数（MPN）计数法（15管）菌数检索表中查出菌数近似值。若15管全部出现阳性或阴性反应，即选择的样品浓度梯度过高或过低。

A.1.6 计算样品含菌数

$$N=\frac{N_a}{10V} \qquad \cdots\cdots(A.1)$$

式中：

N——样品含菌数，单位为个每升（cells/L）；

N_a——表A.1中的最可能数，单位为个（cells）；

V——接种最高浓度管所含样品原液的体积，单位为升（L）。

A.1.7 记录

将分析结果记录于表H.13。

表 A.1 最可能数（MPN）计数法（15管）菌数检索表

阳性管数			每100 cm^3 水样的菌数最近似值	95%可信限值		阳性管数			每100 cm^3 水样的菌数最近似值	95%可信限值	
5个10 cm^3 管	5个1 cm^3 管	5个0.1 cm^3 管		下限	上限	5个10 cm^3 管	5个1 cm^3 管	5个0.1 cm^3 管		下限	上限
0	0	0	<2			0	2	0	4	<0.5	11
0	0	1	2	<0.5	7	0	2	1	6	<0.5	15
0	0	2	4	<0.5	11	0	3	0	6	<0.5	15
0	1	0	2	<0.5	7	1	0	0	2	<0.5	7
0	1	1	4	<0.5	11	1	0	1	4	<0.5	11
0	1	2	6	<0.5	15	1	0	2	6	<0.5	15

表 A.1(续)

阳性管数			每 100 cm³ 水样的菌数最近似值	95%可信限值		阳性管数			每 100 cm³ 水样的菌数最近似值	95%可信限值	
5 个 10 cm³ 管	5 个 1 cm³ 管	5 个 0.1 cm³ 管		下限	上限	5 个 10 cm³ 管	5 个 1 cm³ 管	5 个 0.1 cm³ 管		下限	上限
1	0	3	8	1	19	3	2	2	20	6	60
1	1	0	4	<0.5	11	3	3	0	17	5	46
1	1	1	6	<0.5	15	3	3	1	21	7	63
1	1	2	8	1	19	3	4	0	21	7	63
1	2	0	6	<0.5	15	3	4	1	24	8	72
1	2	1	8	1	19	3	5	0	25	8	75
1	2	2	10	2	23	4	0	0	13	3	31
1	3	0	8	1	19	4	0	1	17	5	46
1	3	1	10	2	23	4	0	2	21	7	63
1	4	0	11	2	25	4	0	3	25	8	75
2	0	0	5	<0.5	13	4	1	0	17	5	46
2	0	1	7	1	17	4	1	1	21	7	63
2	0	2	9	2	21	4	1	2	26	9	78
2	0	3	12	3	28	4	2	0	22	7	67
2	1	0	7	1	17	4	2	1	26	9	78
2	1	1	9	2	21	4	2	2	32	11	91
2	1	2	12	3	28	4	3	0	27	9	80
2	2	0	9	2	21	4	3	1	33	11	93
2	2	1	12	3	28	4	3	2	39	13	106
2	2	2	14	4	34	4	4	0	34	12	96
2	3	0	12	3	28	4	4	1	40	14	108
2	3	1	14	4	34	4	5	0	41	14	110
2	4	0	15	4	37	4	5	1	48	16	124
3	0	0	8	1	19	5	0	0	23	7	70
3	0	1	11	2	25	5	0	1	31	11	89
3	0	2	13	3	31	5	0	2	43	15	114
3	1	0	11	2	25	5	0	3	58	19	144
3	1	1	14	4	34	5	0	4	76	24	180
3	1	2	17	5	46	5	1	0	33	11	93
3	1	3	20	6	60	5	1	1	46	16	120
3	2	0	14	4	34	5	1	2	63	21	154
3	2	1	17	5	46	5	1	3	84	26	197

表 A.1(续)

阳性管数			每 100 cm³ 水样的菌数最近似值	95%可信限值		阳性管数			每 100 cm³ 水样的菌数最近似值	95%可信限值	
5 个 10 cm³ 管	5 个 1 cm³ 管	5 个 0.1 cm³ 管		下限	上限	5 个 10 cm³ 管	5 个 1 cm³ 管	5 个 0.1 cm³ 管		下限	上限
5	2	0	49	17	126	5	4	0	130	35	302
5	2	1	70	23	168	5	4	1	172	43	486
5	2	2	94	28	219	5	4	2	221	57	698
5	2	3	120	33	281	5	4	3	278	90	849
5	2	4	148	38	366	5	4	4	345	117	117
5	2	5	177	44	515	5	4	5	426	145	1 161
5	3	0	79	25	187	5	5	0	240	68	754
5	3	1	109	31	253	5	5	1	348	118	1 005
5	3	2	141	37	344	5	5	2	542	180	1 405
5	3	3	175	44	503	5	5	3	920	300	3 200
5	3	4	212	53	669	5	5	4	1 600	640	5 800
5	3	5	253	77	788	5	5	5	≥2 400		

附 录 B
(规范性附录)
浮游生物样品编号、生物量测定、计数和鱼类浮游生物网具

B.1 浮游生物样品编号

根据海上采样记录，各类样品依次排列编号。编号由代表采样海区、采样方式、使用网型、采样年份和样品序号等内容的代号依次组成。

B.1.1 采样海区表示

采样海区可用汉语拼音的第一个字母表示。

B.1.2 调查方式和网具

D——大型浮游生物网自海底至海面垂直采样；
Z——中型浮游生物网自海底至海面垂直采样；
X——小型浮游生物网自海底至海面垂直采样；
Ⅰ——浅水Ⅰ型浮游生物网自海底至海面垂直采样；
Ⅱ——浅水Ⅱ浮游生物网自海底至海面垂直采样；
Ⅲ——浅水Ⅲ型浮游生物网自海底至海面垂直采样；
SH——深水浮游生物网自海底至海面垂直采样；
W_2——Wp_2 型浮游生物网自海底至海面垂直采样；
W_3——WP_3 型浮游生物网自海底至海面垂直采样；
N——北太平洋浮游生物标准网自海底至海面垂直采样；
B——双鼓网（即：Bongo 网）自海底至海面垂直采样；
F——垂直分段采样；
L——连续观测采样；
S——采水；
Sdy——大型浮游生物网表层水平拖曳采样。

B.1.3 采样年份表示

采样年份以阿拉伯数字表示。

B.2 微微型、微型和小型浮游生物丰度和细胞特性测定

B.2.1 沉降计数法

B.2.1.1 主要工具

倒置显微镜、沉降器等。

B.2.1.2 鉴定与计数

将水样或混合样（根据调查性质及不同要求，由 50 cm^3 或 100 cm^3 等量的数层水样混合而成）每份取 3 个分样，分别装满 3 个等体积的沉降器（10 cm^3～20 cm^3），加盖玻片静置 24 h 后，使用倒置显微镜鉴定，计数。取样体积应视样品浊度和浮游植物密度而定。

B.2.2 浓缩计数法

B.2.2.1 主要仪器设备

显微镜、取样管、浮游植物计数框等。

B.2.2.2 鉴定与计数

视样品中浮游植物数量多少，浓缩或稀释至适当体积，用取样管搅拌均匀，迅速将取样管直立于样

品中，准确地一次吸取所需体积并移入浮游植物计数框，加盖玻片后进行鉴定与计数；浮游植物的计数视其数量多少确定计数全部、1/2 或 1/4，每个样品重复计数 3 次。浮游动物每次的计数值应在 100 个以上。

B.2.3 不易计数类别的处理

在鉴定和计数过程中，凡遇失去色素的浮游植物细胞和细胞不到一半的残体均不计数。未完成细胞分裂者作为一个细胞计数。成团的大群体和束状群体等不易计数的种类时，可用等级符号表示其出现量的多少。对成大群体的近底层种类或底栖种类应单独计数，并单项列入浮游植物总量中。

B.2.4 流式细胞测定技术(FCM)

B.2.4.1 使用范围

用于海洋微微型光合浮游生物（Photosynthetic picoplankton，<2 μm，主要包括聚球藻-Synechococcus，原绿球藻-Prochlorococcus 和微微型真核藻-Picoeukaryotes)丰度和细胞特性的测定。流式细胞测定技术可对那些因表面无光泽而无法被落射荧光显微术辨别的浮游植物细胞进行分析，并对个体进行快速和精确测量，能够分辨自养和异养类群，也可区分碎屑或沉积物。

B.2.4.2 主要仪器设备

流式细胞测定仪、液氮罐等。

B.2.4.3 试剂和溶液

10 cm^3 经 0.2 μm 滤膜预过滤的 10％多聚甲醛、200 mm^3 经 0.2 μm 滤膜预过滤的 25％戊二醛、10 mmol磷酸缓冲液、0.95 μm 荧光微珠悬浊液（每毫升经 0.2 μm 滤膜过滤海水中有 10^6 个左右)、鞘液——经 0.2 μm 滤膜过滤的海水。

B.2.4.4 测定步骤

B.2.4.4.1 技术要求

微微型光合浮游生物的流式细胞测定最好在船上进行，使用现场采集的新鲜样品；当无法现场测定时，用聚甲醛和戊二醛溶液固定，再经液氮速冻保存，可最大限度地保持浮游植物的细胞特性。

测定注意事项：应小心保存样品；很好地区分生物细胞和噪声；正确鉴别不同类群；精细测量样品在仪器中的流速等。

B.2.4.4.2 样品采集和保存

将 1 毫升样品放入预先贴好标签的冷冻小管中，加入 100 mm^3 体积分数为 10％多聚甲醛和 0.5％戊二醛的混合液，在室温下放置 15 min 后，将样品置于液氮中速冻，然后转移到－80℃条件下保存直至分析。

B.2.4.4.3 流式细胞分析

将样品置于 37℃水浴中迅速解冻，将 10 mm^3 的荧光微珠悬浊液和 1 cm^3 的样品加入贴好标签的流式细胞测定管中，在仪器上进行分析。

B.2.4.4.4 数据获取

使用列表模式(Listmode)提取数据。

B.2.4.4.5 数据处理

B.2.4.4.5.1 不同类群的区分

通过对各细胞光散射参数（前角光散射—FSC 和侧角光散射—SSC，表征细胞大小）和荧光参数（叶绿素—FL3、藻胆蛋白—FL2，表征细胞所含光合色素类别与含量）的测定所获反映细胞特性的信号进行多项组合的双参数分布图的综合分析，来实现不同类群微微型光合浮游生物的区分。例如，聚球藻含藻红蛋白，原绿球藻和微微型真核藻不含或极少含（FL2 高度差异)；细胞大小：微微型真核藻≫聚球藻＞原绿球藻（SSC 高度差异)；叶绿素含量：微微型真核藻≫聚球藻＞原绿球藻（FL3 高度差异)。

B.2.4.4.5.2 各类群细胞丰度的计算

在给定样品中每个类群的绝对细胞丰度可用下式计算：

$$C_{pop} = N_{pop}/(R \cdot T) \cdot (V_{total}/V_{sample}) \qquad \cdots\cdots (B.1)$$

式中：

C_{pop}——类群丰度，单位为个每立方毫米（cells/mm³）；

N_{pop}——获取细胞数，单位为个（cells）；

R——样品流速，单位为立方毫米每分钟（mm³/min）；

T——样品测定时间，单位为分钟（min）；

V_{total}——样品体积加添加物（固定剂，荧光微珠等）体积，单位为立方毫米（mm³）；

V_{sample}——样品体积，单位为立方毫米（mm³）。

B.3 大、中型浮游生物生物量测定、种类鉴定与个体计数

B.3.1 浮游动物体积分数测定

B.3.1.1 主要仪器设备

浮游动物体积测量器、滴定管（50 cm³）和真空泵（30 dm³/min）等。

B.3.1.2 测定

去除样品中的杂物，标定体积测量器的体积为 50 cm³，将样品倾入体积测量器内进行抽滤，样品中的水分滤出后，拧上底盖，再用装满 50 cm³ 海水的滴定管从测量器的加水孔注入海水至液面与指针尖端相接触为止。此时留在滴定管中的水量即代表浮游动物的体积，换算浮游动物体积分数（10^{-6}）。

B.3.2 浮游动物湿重生物量测定

B.3.2.1 主要仪器设备

电子天平（感量 0.001 g）、真空泵（30 dm³/min）、布氏漏斗和抽滤瓶等。

B.3.2.2 测定

去除样品中的杂物。取网孔略小于采样网孔的筛绢，剪成与漏斗内径相同的圆块，用水浸湿后沥干称重，并作标定质量的标记，可多次使用。测定时，将标定质量的筛绢平铺于漏斗中，倾入样品抽滤片刻，移出载有样品的筛绢至吸水纸上吸去筛绢底表多余水分，最后使用电子天平称重。从总质量减去筛绢质量即得样品湿重，换算浮游动物湿重生物量（mg/m³）。称重完毕，将样品倒回原样品瓶、供种类鉴定和个体计数用。

B.3.3 浮游动物干重生物量测定

B.3.3.1 主要仪器设备

分析天平（感量 0.01 mg）、烘箱（±1℃）、真空泵（30 dm³/min）。

B.3.3.2 测定

用已知质量的筛绢过滤样品，烘干（60℃）24 h 后称重。总质量减去筛绢质量为浮游动物干重含量。换算浮游动物的干重生物量（mg/m³）。

B.3.4 浮游生物种类鉴定与个体计数

B.3.4.1 主要仪器设备

体视显微镜、普通显微镜、浮游生物计数框和浮游生物取样管等。

B.3.4.2 鉴定与计数

将样品倒入浮游生物计数框中，于体视显微镜下鉴定计数。若样品数量大，浮游动物样品可先挑出个体较大的大型甲壳类、箭虫等全部计数，其余样品用浮游生物取样管取样（1/10 或 1/20）鉴定、计数，换算浮游动物个体数（ind/m³）；夜光藻样品可直接用浮游生物取样管取样（1/10 或 1/20）计数，换算夜光藻个体数（ind/m³）。

B.3.4.3 残体的计数

浮游动物残缺个体按头部或尾部(管水母按上泳钟或下泳钟、保护叶或生殖泳钟)计数,同一种类(或同一态)的残体二者只能取一,并以数量较多者为准。

B.4 鱼类浮游生物个体计数

B.4.1 主要仪器设备

体视显微镜,普通显微镜,鱼卵和仔、稚鱼计数框和取样框。

B.4.2 计数

将采集样品倒入计数框中先挑出鱼卵和仔、稚鱼于体视显微镜下按种类及其不同的发育阶段分别鉴定计数。卵不分发育期;仔稚鱼分为仔鱼期和稚鱼期。

B.4.3 不易记数种类的处理

在鉴定计数过程中,遇到发育不好的坏卵均要计入总量。

B.5 鱼类浮游生物网图

B.5.1 双鼓网

双鼓网网图见图 B.1,其构造与规格见表 B.1。

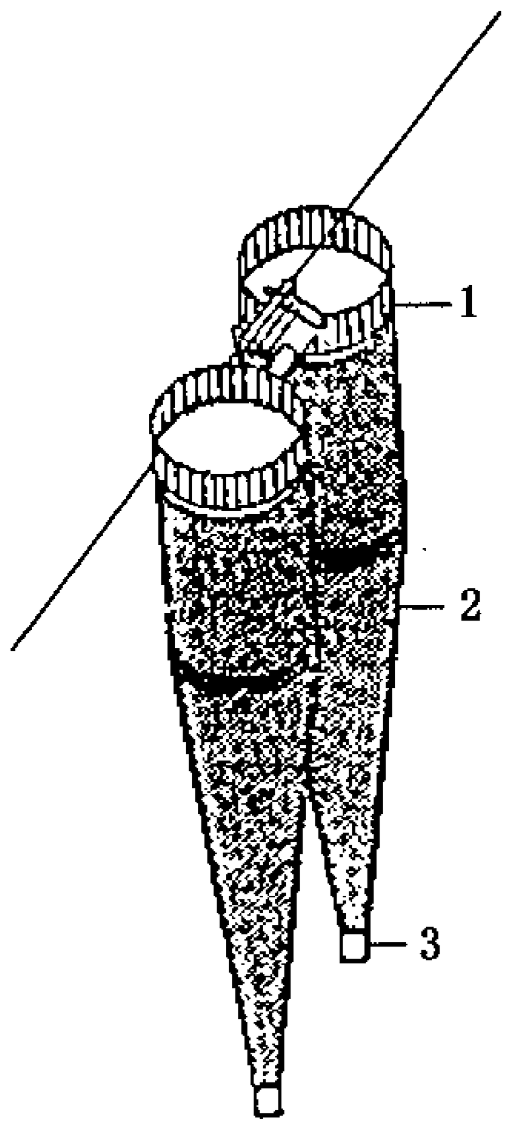

图 B.1 双鼓网图

表 B.1 双鼓网构造与规格

部 位	尺 寸 与 材 料	
网口	内径 60 cm,面积 0.28 m^2,网圈由直径 1 cm 的不锈钢管或圆铁制成	
过滤部	1	长 10 cm,细帆布制成
	2	长 350 cm,型号和规格分别为 JP12(孔径 0.507 mm)或 CQ14 (孔径 0.505 mm)及 CQ20 (孔径 0.336 mm)或 JQ20 (孔径 0.322 mm)筛绢制成
网底部	3	内径 9 cm,长 5 cm,细帆布制成
全长		360 cm(网底部未计在内)

B.5.2 北太平洋网

北太平洋网网图见图 B.2,北太平洋网构造与规格见表 B.2。

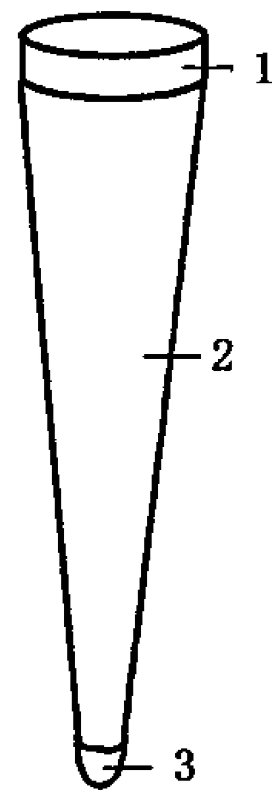

图 B.2 北太平洋网图

表 B.2 北太平洋网构造与规格

部　位	尺　寸　与　材　料	
网口	内径 45 cm，面积 0.16 m^2，网圈用直径 1 cm 的不锈钢管或圆铁制成	
过滤部	1	长 10 cm，细帆布制成
	2	长 170 cm，CQ20（孔径 0.336 mm）或 JQ20（孔径 0.322 mm）筛绢制成
网底部	3	内径 9 cm，长 5 cm，细帆布制成
全长	180 cm（网底部未计在内）	

附　录　C
（规范性附录）
大型底栖生物固定液配制、样品编号和海底照相与录像

C.1　固定液配置

C.1.1　中性甲醛溶液

体积分数为 5%甲醛溶液加十水合四硼酸钠或六亚甲基四胺。

C.1.2　丙三醇乙醇溶液

体积分数为 75%乙醇加体积分数为 5%丙三醇。

C.1.3　甲醛乙醇混合液

体积分数为 2%甲醛溶液与体积分数为体积分数为 50%乙醇等量混合。

C.1.4　布因(Bouinn)固定液

三硝基苯酚(苦味酸)饱和溶液 75 cm^3、甲醛溶液 25 cm^3、冰乙酸 5 cm^3。

C.1.5　四氯四碘荧光素染色剂固定液

1 g 四氯四碘荧光素溶于 1 dm^3 体积分数为 10%的甲醛溶液中。

C.2　大型底栖生物样品编号

C.2.1　采泥样品编号 MXAY

M——调查船代号(代号可随船名而改变)

X——采样站先后序号(*X*=1、2、3……)

A——采泥样品代号(代号固定不变)

Y——采泥样品序号(*Y*=1、2、3……)

举例:第 1 站第 1 个样品号为 M1A1,第 1 站第 2 个样品号为 M1A2,依次类推。

C.2.2　拖网样品编号 MXBZ

M、*X* 见 C.2.1;

B——拖网样品代号(代号固定不变);

Z——拖网样品序号(*Z*=1、2、3……)。

C.3　标签格式

站号____________ 海区____________	
样品号__________ 深度____________ m	
底质____________ 采泥器____________ m^2	
日期__________年______月______日	
种名______________________________	

采泥样品标签(5 cm×3.5 cm)

站号____________ 海区____________	
样品号__________ 深度____________ m	
底质____________ 网型____________	
日期__________年______月______日	
种名______________________________	

拖网样品标签(5 cm×3.5 cm)

C.4　海底照相、录像与潜水取样

如有特殊需要,应进行海底照相、录像和潜水取样。

为了准确掌握调查海区底栖生物资源分布、数量等情况,应置备潜水设备和配有潜水员,采用国际

上通用的水下图像观察记录和潜水取样方法，采用定点照相与区域范围摄像相结合的方式，对调查区域海底生物资源情况利用水下摄像机进行观察记录，并刻制成光盘长期保存。在采样区首先利用水下照相机对采样区底质表面状况进行水下照相，然后潜水采样(尤其适合于无法采泥或拖网的硬底区)。

C.4.1 仪器设备

C.4.1.1 定位设备

定位使用GPS定位仪，定位精度±5 m。

C.4.1.2 水下照相机

采用水下照相机，工作水深30 m。图像传感器为500万以上像素的影像感应器，影像达2048×1536像素。光学取景器，6倍数码变焦，3倍光学变焦，设有USB界面，方便地将数码影像传至电脑。内置记忆棒(memory stick)插槽。采用记忆棒存储高分辨率数字图像。同时设有闪光灯。

C.4.1.3 水下摄像机

水下彩色摄像机，工作水深为50 m，水平解像度为500线，光学变焦25倍，数字变焦50倍，具有超级红外夜摄功能。安装0.7X鱼眼镜头后，视场角超过110°。

C.4.2 观测记录方法

首先在需要进行水中观测和记录的海区利用GPS定位仪进行定位，然后潜水员手持照相机或摄像机在水下进行图像观察记录，其中水下照相采用定点方式；水下摄像采用区域范围方式，一般一个区域摄像时间为1 h左右。

附 录 D
（规范性附录）
小型底栖生物样品分离、计算和几种仪器设备图

D.1 样品分离

D.1.1 标签格式

站号__________ 海区__________
样品号________ 采泥器________
底质__________ 芯样序号______
分层号________ 瓶号__________
日期________年_____月_____日

采泥取芯样样品标签（5 cm×3 cm）

站号__________ 海区__________
样品号________ 网型__________
底质__________ 样品号________
瓶号__________ 放绳长度______
日期________年_____月_____日_____时

拖网取泥样样品标签（5 cm×3 cm）

样品编号见附录 C.2。

D.1.2 分样器

见图 D.1，有机玻璃筒底由隔片等分为 8 个分室，各室底部均配有可插入橡皮塞的小孔，筒旁有一侧孔，恰好位于分室之上，为沉降后分室上部水的排水口，筒顶配有密封盖。

D.1.3 计数皿和计数板

D.1.3.1 计数皿

直径 8 cm～10 cm 的有机玻璃培养皿，皿底部刻划一定间隔的横线（或横竖线组成的方格）用于小型生物不同类群的计数。

D.1.3.2 计数板

仿浮游动物计数板，长、宽、高分别为 8 cm×14 cm×1.5 cm。具体大小应视体视显微镜的底盘大小而定。

D.1.4 小型底栖生物的分离

D.1.4.1 封闭连续淘洗法（图 D.2）

a) 按图 D.2 接水泵或自来水龙头，调节水压至沉积物充分淘洗而又不致使砂粒冲出管 A。
b) 淘洗时间依分样品的量而异，一般定量分层样品应淘洗 10 min。
c) 淘洗完成后，淘洗水通过网筛（孔径 0.042 mm 至 0.050 mm）至水槽。
d) 冲洗网筛上的残留物（小型底栖生物）至已备好的计数皿或计数板内，供计数。若不能立即计数，应保存在盛有 5%甲醛的小玻璃管内（或盘尼西林小瓶中）。

D.1.4.2 海冰法（图 D.3）

a) 取适量海水（最好来自采样生境的海水），过滤、冰冻、破碎备用；
b) 将一定长度的有机玻璃管（4.4 cm～6.0 cm 内径），固定于滴定管架上，管底粘附一定孔径的尼龙网片（孔径大小以底质及要观察的动物类群而异）；
c) 将砂样移入有机玻璃管，依次放置脱脂棉，碎海冰，并调整管底网筛正好浸入结晶皿内的过滤海水为宜；
d) 随海冰的融化，小型动物被收集于结晶皿内，结晶皿内的海水以一定孔径（0.042 mm～0.050 mm）网筛过滤，残渣冲至计数皿内，供镜检；
e) 本法适应于小型动物和微型动物的定性分离，有助于初学者对小型底栖动物类群的鉴别。

D.1.4.3 快速离心分离

a） 硅溶胶（Ludox-TM）离心分选，适用于泥质样品；

b） 离心管中每次添加的硅溶胶溶液一定要与沉积物样品充分搅匀，否则会降低分选效率；

c） 硅溶胶液与样品的比例（3～4∶1）、离心速度（1 800 r/min）、离心时间（约 5 min）和离心重复次数（3 次左右），按以上程序可达到至少 95%以上的分离效率，但不同的海域不同的沉积类型可能会有所差异，建议在初试时可对以上参数作一些对比和检验；

d） 硅溶胶，具腐蚀性，应在通风橱内操作，操作人员应带塑料手套，硅溶胶应存放在有色容器并保存在低温暗处。

D.2 小型底栖生物不同类群的换算系数

表 D.1 小型底栖生物的换算系数表

类　群	换算系数 C
线 虫	530
介形类	450
动吻类	295
涡 虫	550
腹毛虫	550
缓步动物	614
多毛类	530
寡毛类	530
等足类	230
注：桡足类的换算系数 C 依形状而不同，见图 D.4。	

D.3 药剂的配制

a） 麻醉剂：氯化镁溶液 $\rho(MgCl_2)=750\ g/dm^3$。

b） 固定剂：体积分数为 10%甲醛、海水、过滤。

c） 染色剂：1 g 四氯四碘荧光素溶于 1 dm^3 体积分数为 10%的甲醛溶液中。

d） 硅溶胶溶液（Ludox-TM）：应加蒸馏水调至相对密度 1.15。

D.4 小型底栖生物群落结构与生物多样性（应用于群落生态学研究。选做）

该项要素视工作需要和条件状况确定是否进行或部分进行。

D.4.1 多变量分析

多变量分析包括一系列以等级相似性为基础的非参数技术方法：等级聚类（cluster）、非度量多维标度（MDS）、主分量分析（PCA）、ANOSIM 检验、SIMPER 分析、BIOENV/BVSTEP 分析和 RELATE 检验。

a） 原始生物资料矩阵和环境资料矩阵的建立；

b） 样品间（非）相似性测定和（非）相似性矩阵的建立第 j 与第 K 个样品间的 Bray-Curtis 相似性 S_{jk} 由以下公式计算：

$$S_{jk}=100\times\left\{1-\frac{\sum_{i=1}^{p}|y_{ij}-y_{ik}|}{\sum_{i=1}^{p}(y_{ij}+y_{ik})}\right\} \quad \cdots\cdots(D.1)$$

式中：

y_{ij}——原始矩阵第 i 行和第 j 列的输入值。

注：即第 j 个样品中第 i 种的丰度（或生物量）（$i=1,2,\cdots p$；$j=1,2,\cdots\cdots n$），y_{ik} 由此类推。

c) 计算原始环境矩阵中每对样品间环境组成非相似性，产生一个三角形非相似性矩阵。第 j 与第 k 个样品间的欧氏距离非相似性 d_{jk} 为：

$$d_{jk}=\sqrt{\sum_{i=1}^{p}(y_{ij}-y_{ik})^2} \quad \cdots\cdots(D.2)$$

d) 通过样品的聚类和标序展示群落结构格局，参见附录 D；

e) 群落结构差异的统计检验，ANOSIM 检验，BIOENV/BVSTEP 分析和 RELATE 检验；

f) 群落结构与环境变量的多元相关分析，采用主分量分析（PCA）及相关检验。

D.4.2 单变量分析和生物多样性的测定

a) 香农-威纳信息指数（Shannon-Wiener information index）

$$H'=-\sum(P_i\cdot\log P_i) \quad \cdots\cdots(D.3)$$

式中：

P_i——样品中第 i 种的个体数占该样品总个体数之比。

注：log 默认以 e 为底，2 和 10 为可选。

b) 种丰富度指数（Margalef's species richness）

$$d=\frac{S-1}{\ln N} \quad \cdots\cdots(D.4)$$

式中：

S——样品包含的种数；

N——总个体数。

c) 均匀度指数（Pielou's evenness）

$$J'=\frac{H'}{\ln S} \quad \cdots\cdots(D.5)$$

式中：

H'——为香农-威纳信息指数；

S——为样品包含的种数。

d) 辛普森优势度指数（Simpson's dominance）

$$1-\lambda'=1-\frac{\sum N_i(N_i-1)}{N(N-1)} \quad \cdots\cdots(D.6)$$

式中：

N_i——是样品中第 i 种的个体数，单位为个（ind）；

N——为该样品的总个体数，单位为个（ind）。

D.5 取样大小 95%置信度的计算

$$95\%\ 置信度界限=\log^{-1}\left[\bar{y}\pm t_{0.05}(n-1)\times\sqrt{\frac{s}{n}}\right] \quad \cdots\cdots(D.7)$$

式中：

$\bar{y}$——几何平均数；

s——方差。

D.6 潜水装置

潜水员的潜水装置见图 D.5。

单位为毫米

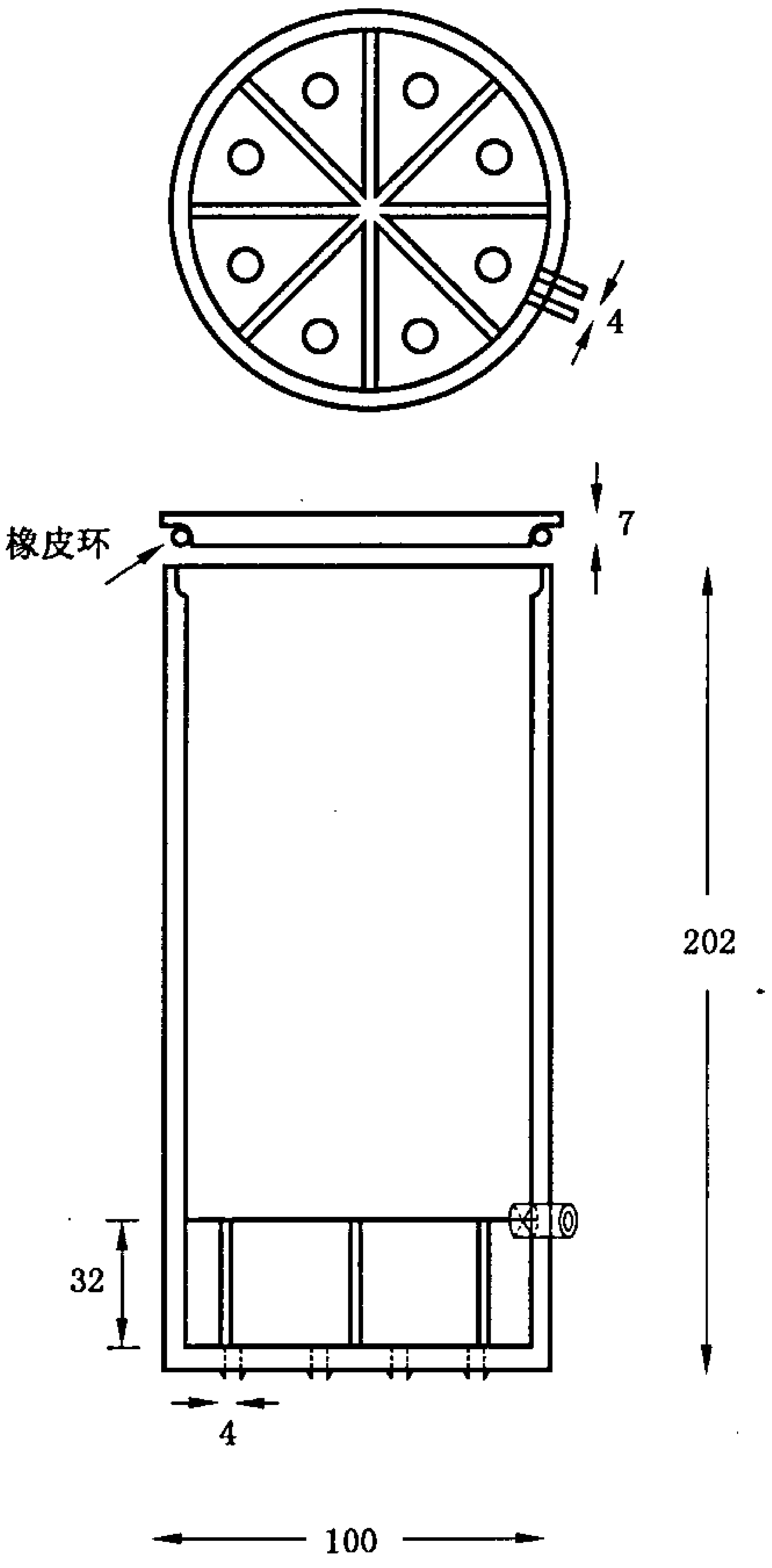

图 D.1 小型底栖生物分样器结构底面观和侧面观示意图

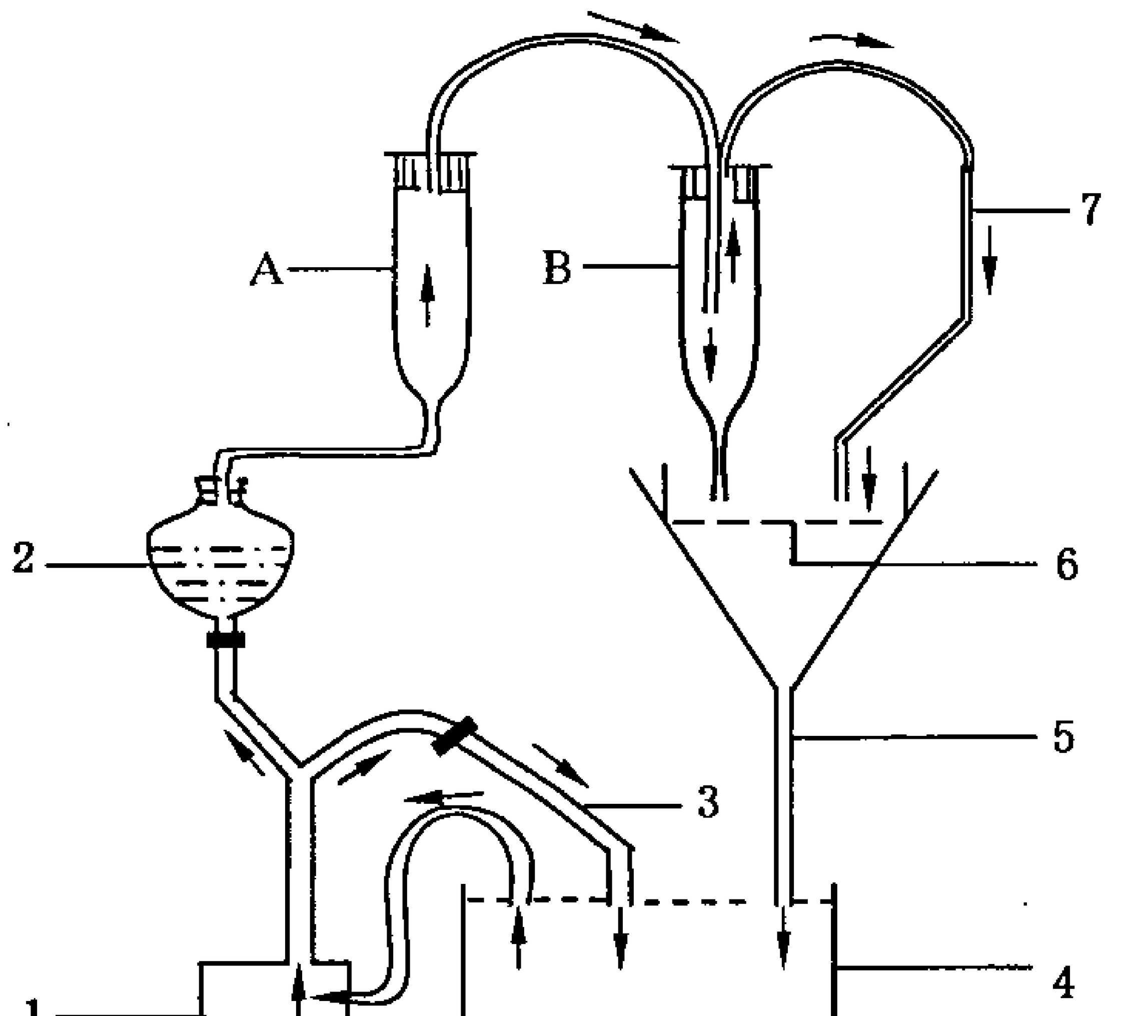

1——水泵；
2——沉积物样品；
3——旁路回水管；
4——水槽；
5——回水管；
6——蓄留小型生物的网筛；
7——溢水管。

图 D.2 砂质样品封闭淘洗装置示意图

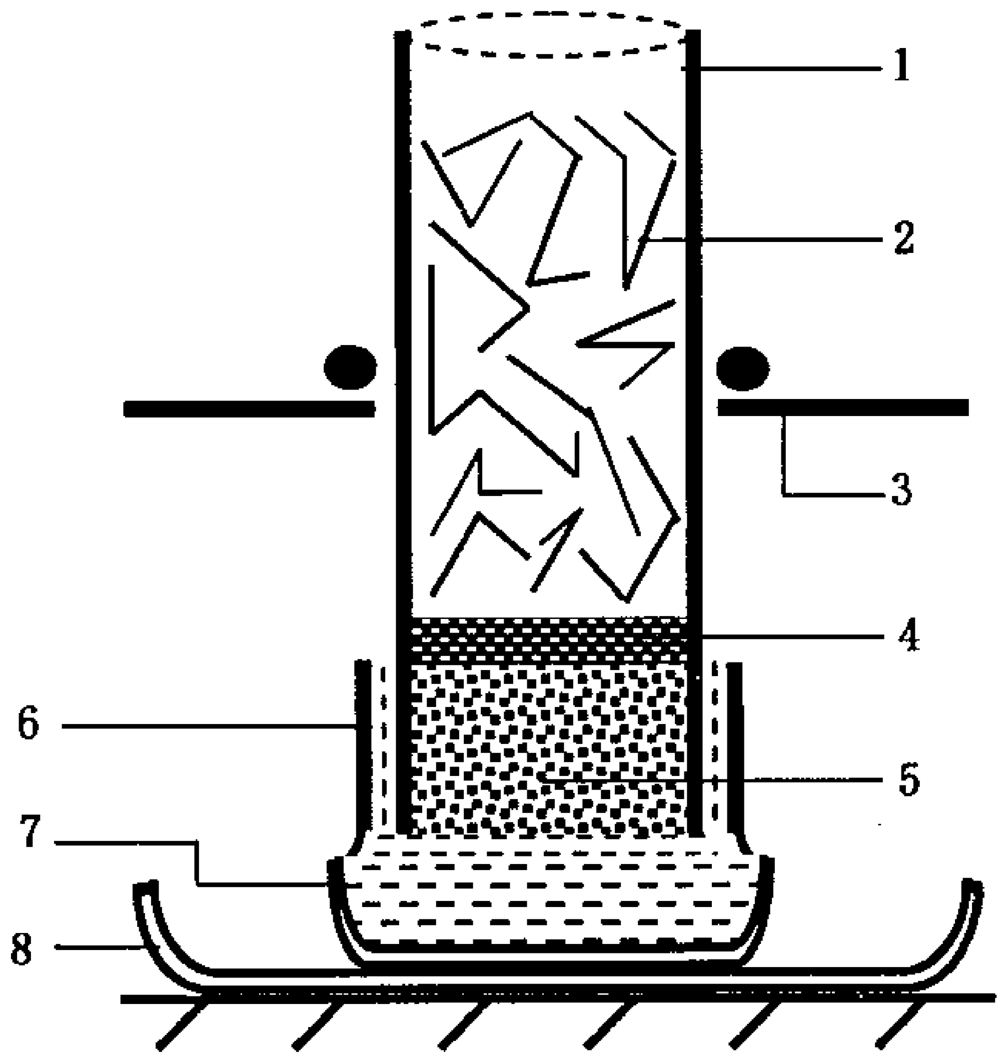

1——有机玻璃管；
2——海冰；
3——支撑架；
4——脱脂绵；
5——沉积物；
6——网筛；
7——内盛海水结晶皿；
8——大培养皿。

图 D.3 海冰法分选小型生物示意图

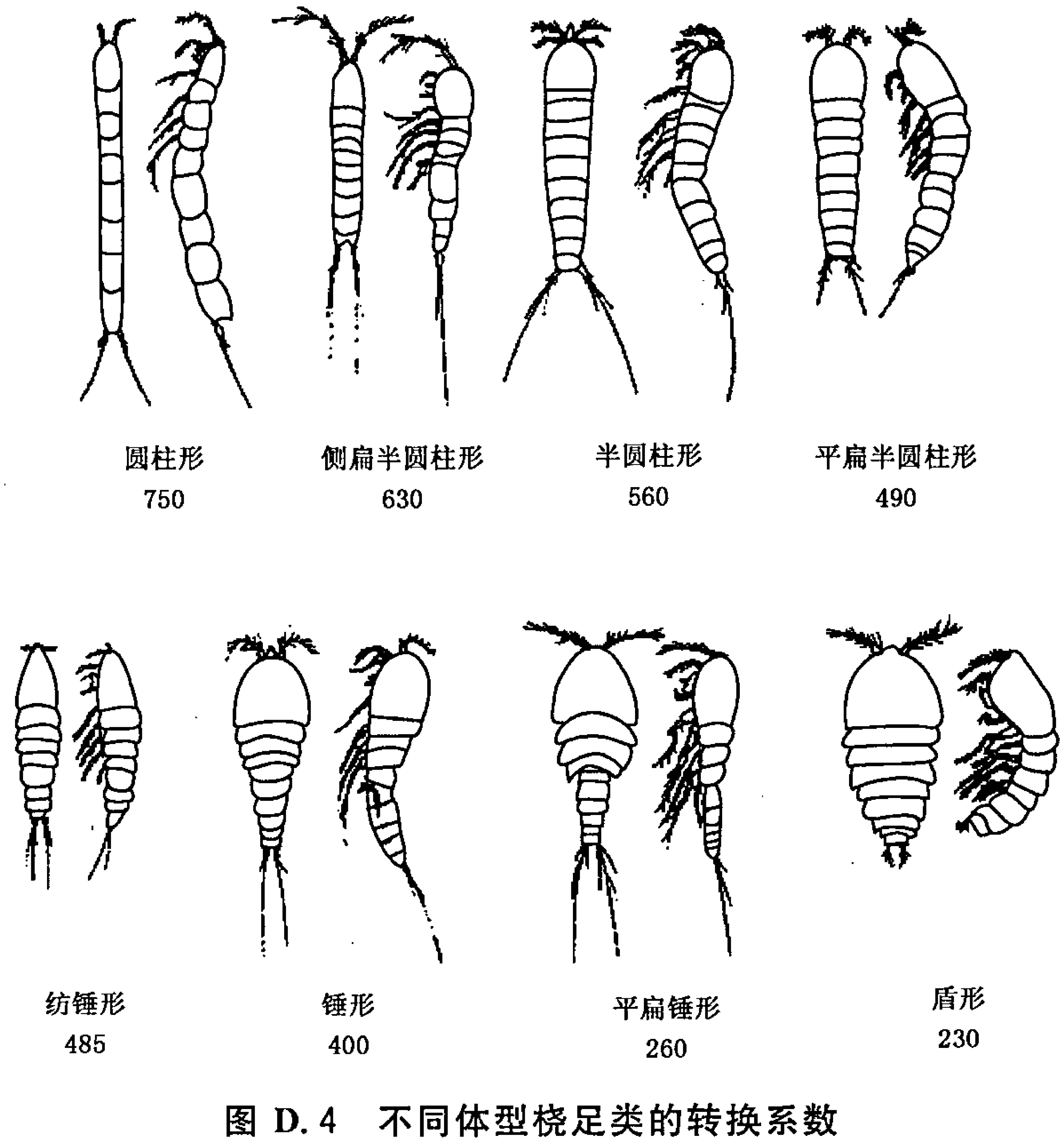

图 D.4 不同体型桡足类的转换系数

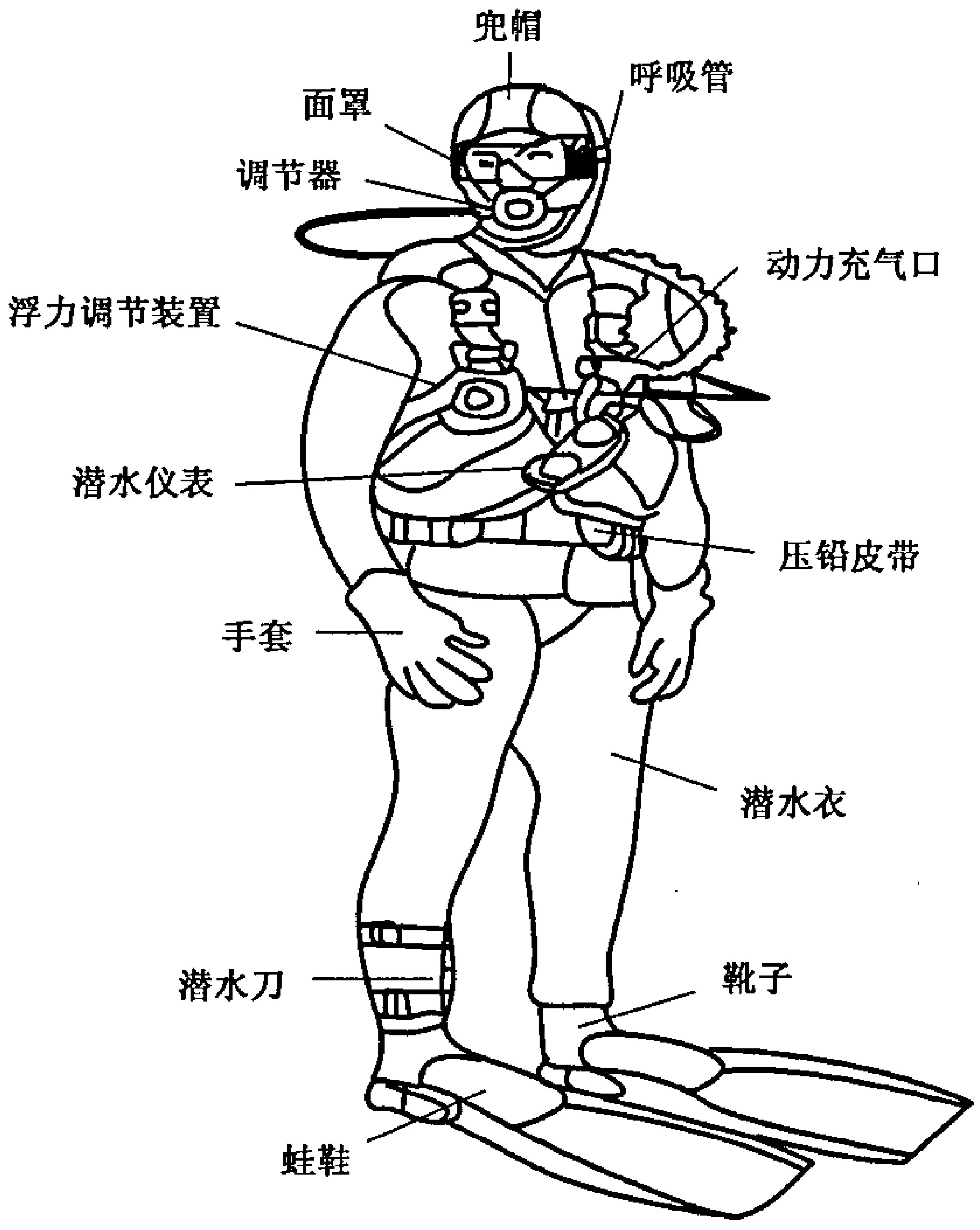

图 D.5 携带配套的水下呼吸器(SCUBA)等装置的潜水员示意图

附　录　E
（规范性附录）
潮间带生物调查中潮位测量法和几种设备图

E.1　潮间带的划分

调查地点选定后，应根据当地的潮汐水位参数或岸滩生物的垂直分布，将潮间带划分为若干区（带）、层（亚带），划分方法如下：

E.1.1　潮汐水位参数划分法

E.1.1.1　半日潮类型

a）高潮区（带）：最高高潮线至小潮高潮线之间的地带；

b）中潮区（带）：小潮高潮线至小潮低潮线之间的地带；

c）低潮区（带）：小潮低潮线至最低低潮线之间的地带。

E.1.1.2　日潮类型

a）高潮区（带）：回归潮高潮线至分点潮高潮线之间的地带；

b）中潮区（带）：分点潮高潮线至分点潮低潮线之间的地带；

c）低潮区（带）：分点潮低潮线至回归潮低潮线之间的地带。

E.1.1.3　混合潮类型

a）高潮区（带）：高高潮线至低高潮线之间的地带；

b）中潮区（带）：低高潮线至高低潮线之间的地带；

c）低潮区（带）：高低潮线至低低潮线之间的地带。

E.1.2　生物垂直分布带划分法

根据生物群落在潮间带的垂直分布来划分，由于生物群落可随纬度高低、底质类型、外海内湾、盐度梯度、向浪背浪、背阴向阳等复杂环境因素的不同而改变，因此，要提供一个统一模式是困难的。一般而言，岩石岸大体分为：滨螺带；藤壶-牡蛎带；藻类带。各地在调查时可根据各区、层的群落优势种给予更确切的命名。

在外侧沿岸和岛屿，因受浪击的影响，生物种类的分布超过高潮区时，应测量生物带的高度，也应在生物带相应的部位进行样品的采集。

E.2　站点潮位的计算方法

E.2.1　潮汐水位曲线图解法（见图 E.1）

该法是记录调查断面各站被潮水淹没或露出时间，把它标于从调查地点附近验潮站抄录的当日每时实测潮位绘制的图 E.1 上，则知其潮高基准面上高度，如：站 1 于 8 时 15 分淹没，其高度是 1.95 m；又如：站 2 于 14 时 07 分露出，其高度应是 3.5 m。

此外，根据各站淹没或露出时间，还可应用“等腰梯形图卡”法（见潮汐表后介绍），求得各站的潮高基准面上高度。

E.2.2　直接测量法（见图 E.2）

本法较适于坡度大、范围窄的岸滩，无需等待潮水淹没或退离，就可测得其潮高基准面上高度。方法如图 E.2 所示，由当日最低潮位逐渐向上测量。设：站 1 是当日最低潮位（可从验潮站查得其潮高基准面上高度，设为 0.6 m），若测站 2 高度，则只需在站 1、2 分别立标杆甲、乙，并于两标杆间拉一绳索，使之与海面平行或同时垂直两标杆，记下两标杆高度，经简单计算，便知其潮高基准面上高度。例如：标杆甲、乙高度分别为 2.1 m 和 0.8 m，则站 2 高度为 1.9（0.6 m+2.1 m−0.8 m）。依次同样可测量上面各站。

若调查地点远离验潮站，可依本法实际测得数据，与两侧验潮站观测的潮时和潮差作比较，用内插法求得各站的高度。须注意的是，当风浪较大时，此法误差较大，应慎用。

E.2.3 水准仪测量法

本法精度高，在已有大地高程测量的地方，只需在欲测的潮间带附近找得水准点（埋设的水准标石），依站序向下测量。若无大地测量作依据，则应从当日最低潮位（其潮高基准面上高度可由验潮站查得）逐站向上测量。

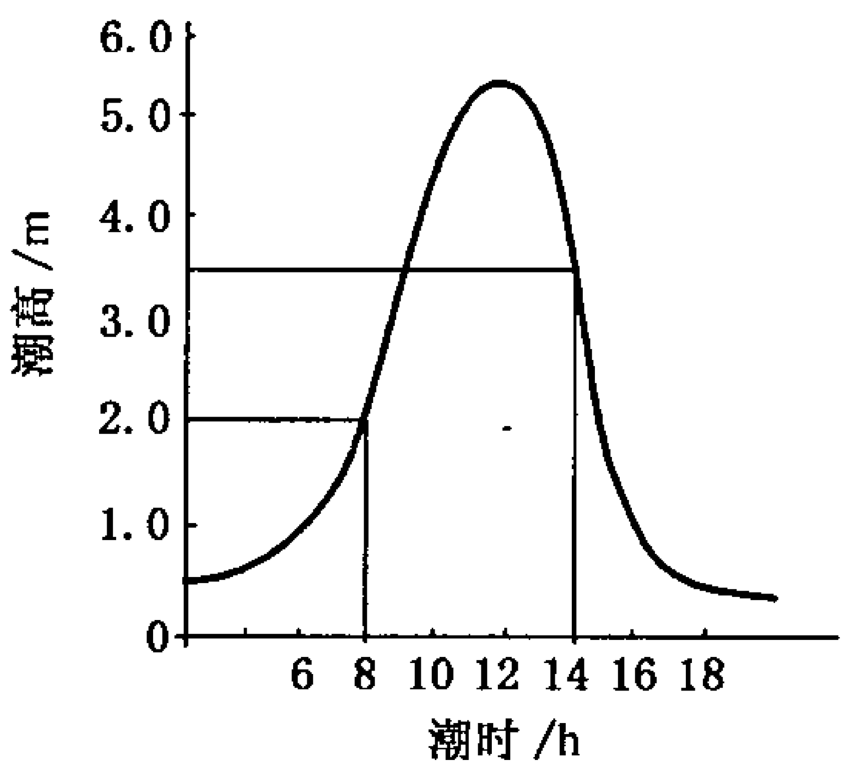

图 E.1 潮汐水位曲线图解法曲线

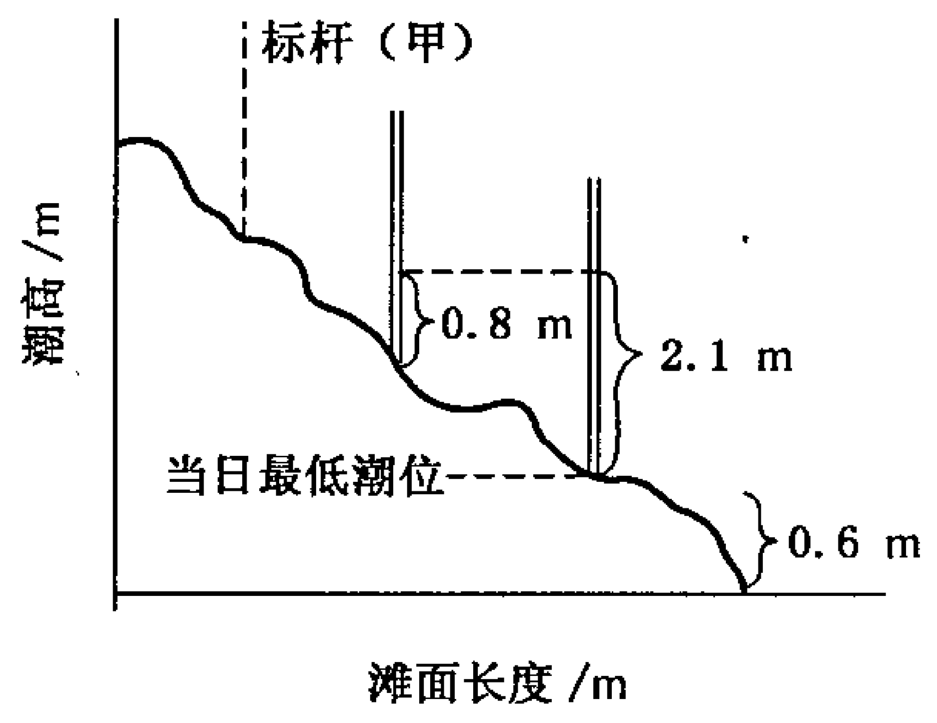

图 E.2 直接测量法曲线

E.3 几种仪器设备图

E.3.1 覆盖面积计数框图和岩石定量采样框图

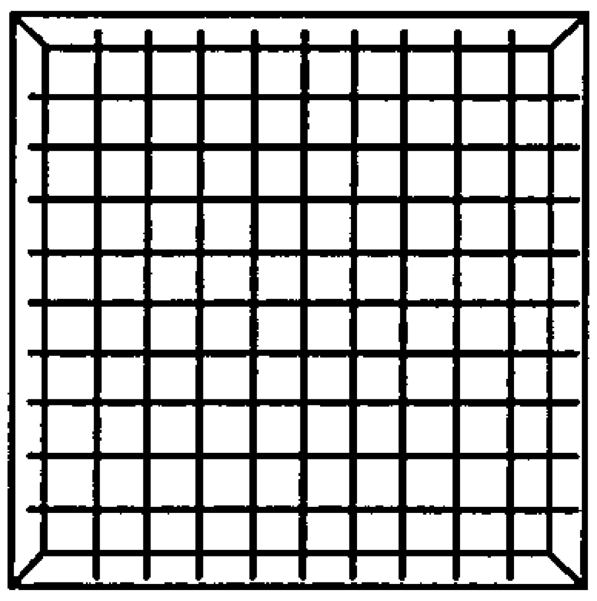

图 E.3 a） 覆盖面积计数框

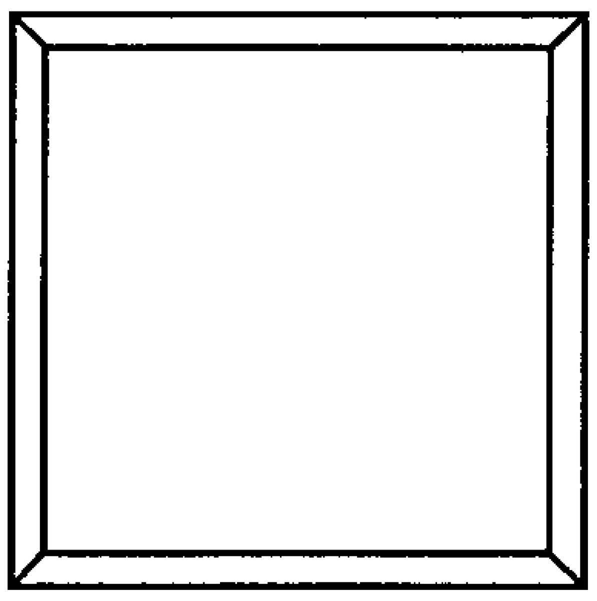

图 E.3 b） 岩石定量采样框

E.3.2 滩涂定量采样框图

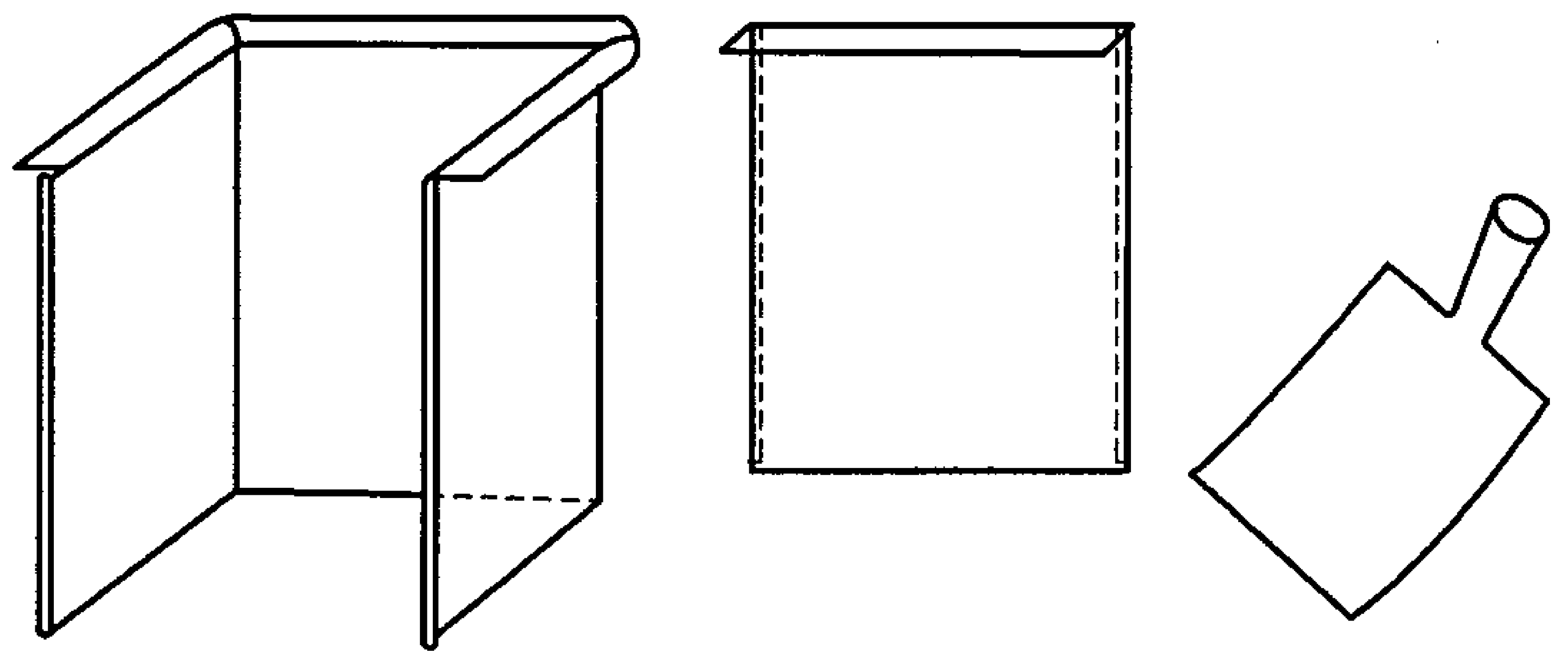

图 E.4 滩涂定量采样框

E.3.3 旋涡分选装置

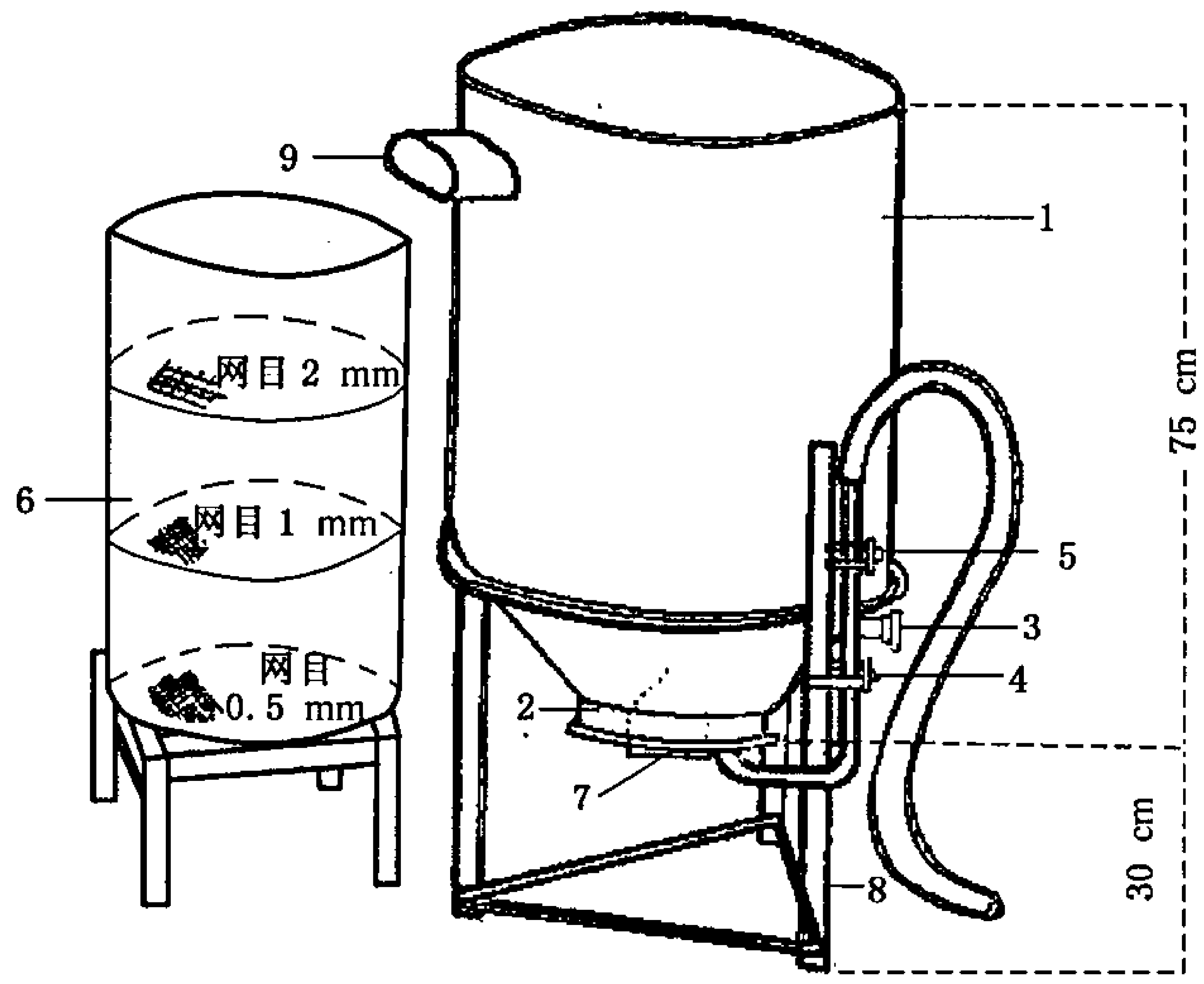

1——筒体；
2——旋涡发生器；
3——进水管；
4——进水阀；
5——分流阀；
6——生物收集器；
7——排渣阀；
8——支架；
9——出水口。

图 E.5 旋涡分选装置

E.3.4　过筛器

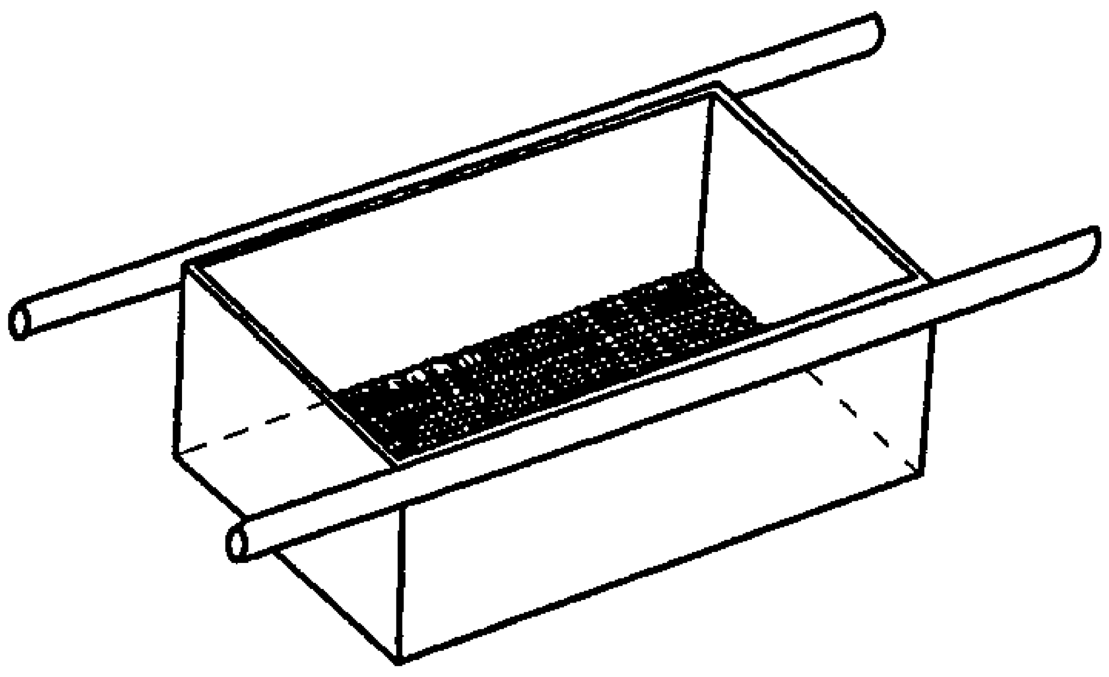

图 E.6　过筛器

E.4　标签

海区____________	断面____________	站位____________
样方____________	潮区____________	底质____________
取样时间____		
种名____		

样品标签(5 cm×3.5 cm)

附 录 F
（规范性附录）
游泳动物性腺成熟度、摄食强度、含脂量及拖网网型

F.1 游泳动物性腺成熟度

F.1.1 鱼类性腺成熟度分为6期

a) 1期——性腺未发育的个体。性腺不发达，紧附于体壁内侧，呈细线状或细带状，肉眼不能识别雌雄。

b) 2期——性腺开始发育或产卵后重新恢复的个体。卵巢呈细管状或扁带状，半透明呈浅红肉色，肉眼能辨明性别，但看不出卵粒。精巢扁平稍透明，呈灰白色或灰褐色。

c) 3期——性腺正在成熟的个体。性腺已较发达，卵巢体积增大，占腹腔1/3～1/2，呈白色或浅黄色，肉眼可看出卵粒。卵粒互相粘连成团块状，难分离。精巢表面呈灰白色或稍具浅红色，压挤精巢，无精液流出。

d) 4期——性腺即将成熟的个体。卵巢体积较大，占腹腔2/3左右，卵粒明显，圆形，呈桔红色或桔黄色，容易使其彼此分离，有时能看到半透明卵，轻压鱼腹无成熟卵流出。精巢显著增大，呈白色，轻压鱼腹能有少量精液流出。

e) 5期——性腺完全成熟，即将或正在产卵的个体。卵巢饱满，充满体腔，卵粒大而透明，且各自分离，对鱼腹稍加压力，透明卵粒即行流出。精巢充满精液，呈乳白色，稍加压力，精液即行流出。

f) 6期——产卵、排精后的个体。性腺萎缩，松弛，充血，呈暗红色。其体积显著缩小，卵巢套膜增厚。性腺内部常残留少量卵粒或精液。

以上6期为一般的划分标准，可根据不同鱼类的情况和需要，对某一期再划分A期、B期，如：5_A期、5_B期等。

若性腺成熟状况处于相邻两期之间，可写出两期的数字，中间加一破折号，如3期－4期、4期－3期等。比较接近那一期，将该期的数字写在前面，如：4期－3期，表明性腺成熟度比较接近于第4期。

如性细胞属于分次成熟，每一生殖季节进行多次产卵的鱼类，可根椐已产过和余下的性细胞发育情况记录，如6期～3期或6期～4期，表明产卵后卵巢内还有一部分卵粒处于3期或4期，但卵巢外观上具有部分6期的特征。

F.1.2 对虾类性腺成熟度分为5期

a) 1期——未发育期。卵巢小，无色，轮廓不清，肉眼不能辨别卵粒。

b) 2期——发育早期。已交配，卵巢开始发育，呈乳白色或淡黄色，卵粒肉眼能辨别，但不能分离。

c) 3期——发育后期。卵巢已大，前叶及中叶已成熟，通过甲壳可见，后叶增宽。肉眼易于辨别卵粒，卵巢表面龟裂，黄色或灰绿色。

d) 4期——成熟期。卵巢膨大，卵粒极为明显，前叶、中叶充分成熟，轮廓清楚，通过甲壳可见，表面龟裂突起，呈褐绿色或灰绿色。

e) 5期——产后恢复期。卵巢萎缩，卵已排空，外观近似1期，呈灰白色、淡红色或淡黄色。

F.1.3 毛虾的性腺成熟度要用显微镜辨别，分为5期

a) 1期——未成熟期。卵巢内以小型卵为主，大小卵粒(0.0230 mm～0.082 8 mm)混杂，卵巢边

缘部分卵粒较大。雌虾交接器内没有精荚。

b) 2期——成熟期。卵巢内卵粒大部分达D型及C型(D、C型的卵径约0.084 mm～0.161 mm),这时卵巢显著增大,边缘呈纹状突起,突起与突起重叠,在第一腹节背部的卵巢向两侧扩张,第二节腹节处则向上隆起,大部分雌虾交接器内已有精荚。

c) 3期——临产卵期。卵巢内卵粒均达A、B型(卵径0.082 8 mm～0.207 0 mm)。卵粒排列整齐,位于第一腹节背部的卵巢已穿过上部肌肉与甲壳接触,并扩展到身体两侧。第二腹节背部的卵巢已挤开上部肌肉向上突出。卵巢呈绿色或棕色。

d) 4期——产卵期。卵巢边缘突起部分已无卵粒,但在卵巢中部血管附近仍有大量小型卵粒,卵巢内部空虚,或仅存极少数卵粒,卵巢外形极不一致,且左右不对称,只剩极薄的一层贴附在其上部的肌肉上。

e) 5期——产后恢复期。卵母细胞已排空,卵原细胞在发育过程中。

F.1.4 梭子蟹性腺成熟度分为6期

a) 1期——尚未交配,腹部呈三角形,性腺未发育呈乳白色,肉眼很难分辨雌雄。

b) 2期——已交配,雌体腹部由三角形变为椭圆形,体内的两个储精囊内各有一个精荚,性腺开始发育,卵巢呈乳白色或粉红色,细带状,肉眼可辨雌雄。

c) 3期——卵巢呈淡黄色或黄红色,带状,肉眼可见细小的卵粒,但不能分离。

d) 4期——卵巢发达,桔红色或红色,扩展到头胸甲的两侧,卵粒明显可见。

e) 5期——卵巢发达且柔软,红色,腹部抱卵,卵粒大小均匀。

f) 6期——已排过卵,卵巢退化,腹部抱卵。

F.1.5 乌贼性腺成熟度分为6期

a) 1期——卵巢很小,卵粒大小相近,卵粒全不透明。

b) 2期——卵巢较大,卵粒大小不一,小型的不透明卵占优势,有少数透明卵或半透明卵,并有花纹卵粒。输卵管内没有卵粒,缠卵腺较小。

c) 3期——卵巢大,约占外套腔的1/4,卵粒大小不一,小型不透明卵很多,约占卵巢的1/2。输卵管中有卵粒,卵粒彼此相连,大约占全部卵数的1/3,有些卵粒还未成熟,缠卵腺较大。

d) 4期——卵巢很大,约占外套腔的1/3,卵粒大小显著不同,小型不透明卵仍占多数,约占卵巢的1/3。输卵管中卵粒很多,约占全部卵数的1/2,缠卵腺很大,约占外套腔的2/5。

e) 5期——卵巢十分膨大,约占外套腔的1/2,小型不透明卵很少,其卵径也小。输卵管中卵粒多而大,约占全部卵数的3/5,透明卵一般分离,呈草绿色。缠卵腺十分肥大,呈白色,其中充满粘液体,表面光滑发亮,约占外套腔的1/2。

f) 6期——已产过卵,卵巢萎缩,其中有少量卵粒稍呈灰褐色。输卵管松弛、呈黄色、尚有少数透明卵存在。缠卵腺干瘪略呈黄色,表面皱纹很多,约占外套腔的1/3。

F.1.6 枪乌贼和太平洋褶柔鱼类的性腺成熟度分为6期

a) 1期——性腺未发育,很小,肉眼可从茎化腕尾部区分雌雄。

b) 2期——发育期。雌体卵巢很小,成带状,卵粒大小不一、全不透明,输卵管很小、管内没有卵粒,输卵管腺小,缠卵腺和副缠卵腺已形成,但还很小,副缠卵腺呈淡黄色;雄性个精巢条状,前列腺肉眼略可见。

c) 3期——未成熟期。卵巢约占外套腔的1/4,卵粒大小不一,小型白色不透明卵约占卵巢的1/2,输卵管中已有少数彼此相连的卵粒,输卵管腺增大呈乳白色,副缠卵腺已出现朱红色的斑点,缠卵腺增大;雄性个体前列腺增大,贮精囊明显,输精管末端膨大成精荚囊,并有少量精荚。

d) 4期——成熟(交尾)期。卵巢大,约占外套腔的1/3,卵粒大小显著不同,白色小型的不透明卵

占卵巢的1/3,输卵管中卵粒很多,约占卵数的1/2,输卵管腺出现微黄色的小点。副缠卵腺有黄豆大,朱红色的斑点多,缠卵腺肥大,呈乳白色。已交尾,受精囊中有精子;雄性个体精巢很大,贮精囊和精荚囊均饱满,轻压阴茎,精荚能排出,轻触精荚,能弹出精子。

e) 5期——完全成熟(产卵)期。卵巢十分膨大,约占外套腔的1/2,小型不透明的卵很少,输卵管中的卵粒多而大、透明分离,约占全部卵数的3/5。输卵管腺呈黄褐色,生殖孔大、松弛。整个副缠卵腺均呈朱红色,缠卵腺十分肥大,色白,表面光滑,可占外套腔的1/3;雄性个体的精巢缩小,比精荚囊还小,精荚囊中精荚饱满,挤到阴茎附近。

f) 6期——产后期。雌性个体已产过卵,卵巢萎缩,尚存的少量卵粒稍呈灰色。输卵管松弛,呈黄色,仅存少量透明卵。副缠卵腺暗红色,缠卵腺干瘪呈淡黄色,表面有皱纹,约占外套腔的1/4。生殖孔松弛破裂,呈雨伞状;雄性个体已完成交尾行为,精巢和前列腺萎缩,呈淡灰色,精荚囊至阴茎存有少量精荚,有的精荚膜已破裂,精子溢出;产后的个体消瘦尖细,胴体肌肉很薄,雄体的眼睛浑浊。

F.1.7 茎柔鱼的性腺成熟度分为5期

a) 1期——未成熟期。雌性,缠卵腺细小而透明,输卵管尚未形成,卵粒未出现。雄性,精巢白色,纤维质,精荚器官细小,透明至半透明,精荚囊空瘪无物。

b) 2期——成熟中。雌性,卵巢具颗粒状表面,缠卵腺呈灰白色和奶油色,输卵管不很明显。雄性,精巢呈奶油色,精荚囊内有少数白色微片。

c) 3期——成熟期。雌性,整个输卵管约占外套腔的1/3,卵呈浓桔黄色,缠卵腺呈白色,与输卵管大小相近。雄性,精荚囊内充满精荚,精巢呈白色,体积增大。

d) 4期——产卵期。雌性,输卵管缩小,内有少数卵子,缠卵腺缩小以至于瘦瘪,呈白蔷薇色。雄性,精荚囊软,半透明,大小均等,内有残余精荚。性器官萎缩期开始。

e) 5期——产后期。产完卵,性器官处于明显的萎缩期。缠卵腺几乎空瘪并明显缩小。

F.1.8 真蛸性成熟度分为3期

a) 1期——未成熟期。雌性,卵巢乳白色。雄性,精荚囊中无精荚。

b) 2期——半成熟期。雌性,卵巢略呈黄色,不透明。雄性,精荚囊中有精荚。

c) 3期——成熟期。雌性,卵巢黄色,透明。雄性,精荚囊中充满精荚。

F.2 游泳动物摄食强度

F.2.1 鱼类摄食强度分为5级

a) 0级——空胃。

b) 1级——胃内有少量食物,其体积不超过胃腔的1/2。

c) 2级——胃内食物较多,其体积超过胃腔的1/2。

d) 3级——胃内充满食物,但胃壁不膨胀。

e) 4级——胃内食物饱满,胃壁膨胀变薄。

F.2.2 虾类摄食强度分为4级

a) 0级——空胃。

b) 1级——胃内仅有少量食物(少胃)。

c) 2级——胃内食物饱满,但胃壁不膨胀(半胃)。

d) 3级——胃内食物饱满,且胃壁膨胀(饱胃)。

F.3 鱼类含脂量分为4级

a) 0级——内脏表面及体腔壁均无脂肪层。

b) 1 级——胃表面有薄的脂肪层,其覆盖面积不超过胃表面的 1/2。

c) 2 级——胃肠表面 1/2 以上的面积被脂肪层覆盖。

d) 3 级——整个胃肠表面被脂肪层覆盖,脂肪充满体腔。

F.4 拖网网型

F.4.1 双船底层有翼单囊 A 型拖网网型

双船底层有翼单囊 A 型拖网网图(见图 F.1);

F.4.2 双船底层有翼单囊 B 型拖网网型

a) 双船底层有翼单囊 B 型拖网网图(见图 F.2);

b) 双船底层有翼单囊 B 型拖网网具第 7 段~第 24 段编结规格(见表 F.1)。

F.4.3 单船有翼单囊拖网网型

单船有翼单囊拖网网图(见图 F.3)。

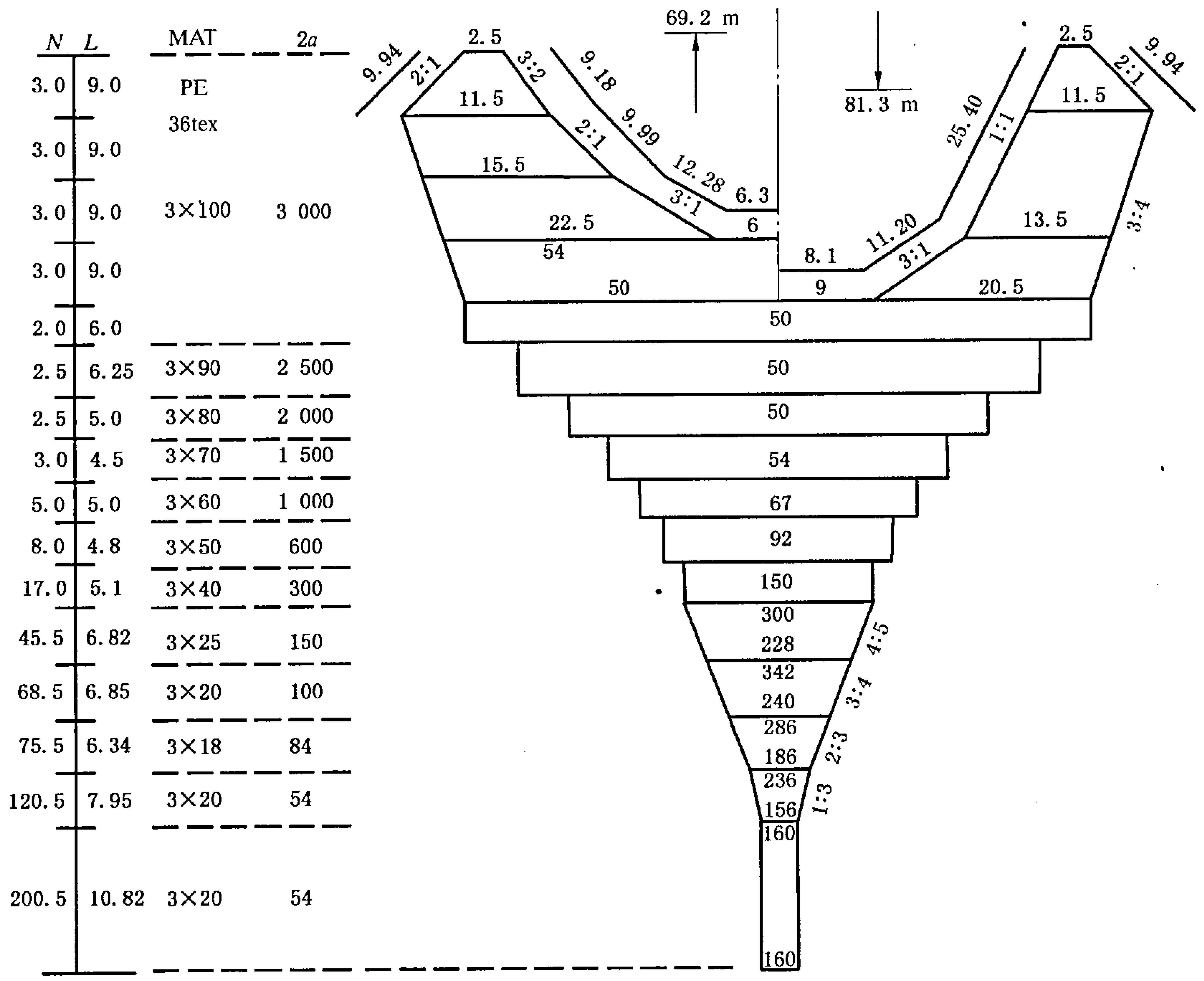

图 F.1 双船底层有翼单囊 A 型拖网网图

(规格 300 m×111.43 m/69.2 m)

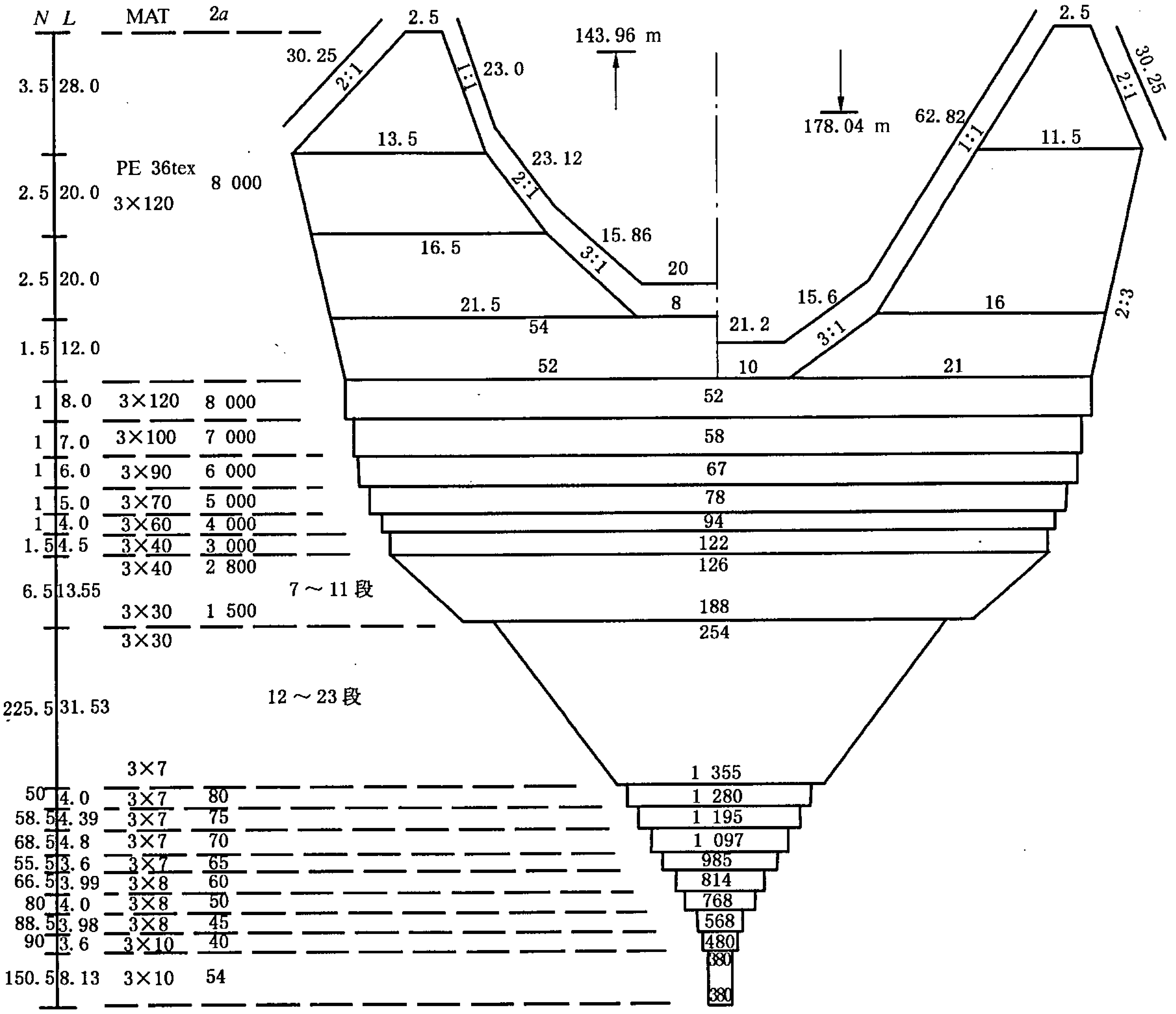

图 F.2 双船底层有翼单囊 B 型拖网网图

(规格 816 m×196.08 m/143.96 m)

表 F.1 双船底层有翼单囊 B 型拖网网具第 7 段～第 24 段编结规格

名称	段 号	网衣编结宽度		网衣编结高度		网目大小 (2a)/mm	网线规格
		起 目	终 目	目数(N)	L/m		
部分网身网衣	7	126	126	1	2.8	2800	3×40
	8	136	136	1	2.5	2500	3×40
	9	146	146	1	2.2	2200	3×40
	10	154	154	1	2.0	2000	3×40
	11	164	164	1	1.8	1800	3×30
	12	188	188	1.5	2.25	1500	3×30
	13	254	254	1.5	1.5	1000	3×30
	14	304	304	2.5	2.0	800	3×30
	15	384	384	3.5	2.1	600	3×20
	16	746	746	6.5	1.95	300	3×15

表 F.1(续)

名称	段号	网衣编结宽度		网衣编结高度		网目大小(2a)/mm	网线规格
		起目	终目	目数(N)	L/m		
部分网身网衣	17	1 064	1 064	12	2.4	200	3×8
	18	1 102	1 102	15.5	2.79	180	3×8
	19	1 237	1 237	21.5	3.23	150	3×8
	20	1 440	1 440	26.5	3.18	120	3×8
	21	1 600	1 600	36	3.6	100	3×7
	22	1 540	1 540	50	2.38	95	3×7
	23	1 493	1 493	31	2.79	90	3×7
	24	1 355	1 355	42.5	3.61	85	3×7

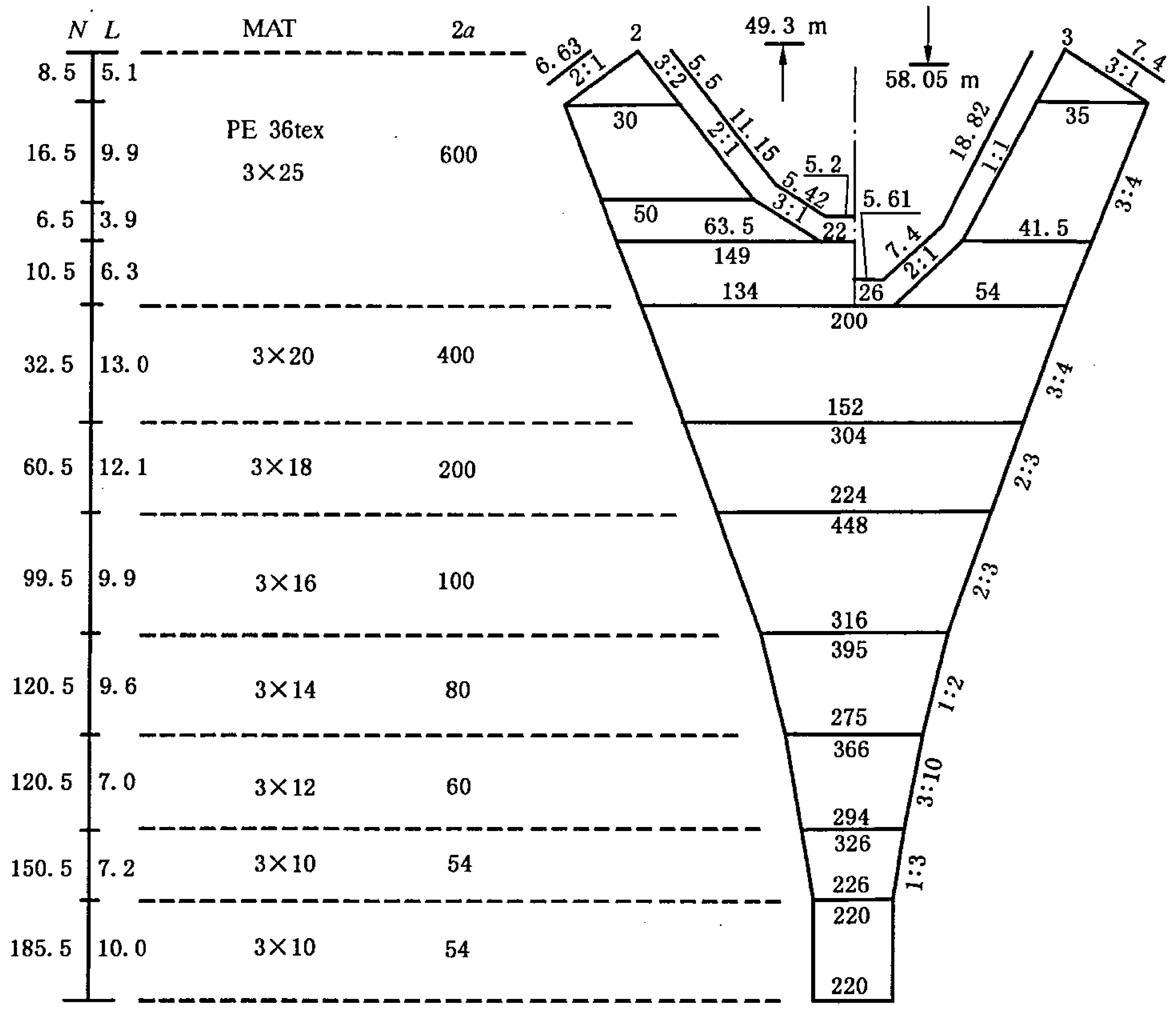

图 F.3 单船有翼单囊拖网网图

(规格 240 m×94 m/49.3 m)

附　录　G
（资料性附录）
渔业资源声学调查与评估（选做）

G.1　技术要求和调查要素

G.1.1　技术要求

a）在调查开始及航次结束时，应严格按照本部分的要求对回声探测-积分系统各进行一次声学校正，以确保原始声学数据的准确性；

b）深入了解调查对象的生态习性和声学映像特征；准确测定和统计现场拖网渔获物组成比例及各渔获种类（包括非调查对象种类）的体长和体重分布，以便进行映像分析和积分值分配；

c）准确查明或现场测定调查对象和各主要渔获种类的目标强度，以对调查对象进行定量声学评估；

d）调查的实施、样品分析和生物学测定以及数据的处理等均应严格按照本部分的有关规定执行；

此外，对调查设计和鱼类的目标强度作以下规定和定义。

G.1.1.1　调查设计

G.1.1.1.1　调查时间

调查时间应根据调查的主要目的而定。以资源量评估为主的调查航次，一般选择调查对象分布较为集中、分布格局较为稳定的时期进行。另外，调查时间的选择还应考虑调查对象的分布特点及环境、海况因素。

G.1.1.1.2　调查范围

尽可能涵盖调查对象的整个分布区域。

G.1.1.1.3　调查航线

G.1.1.1.3.1　航线类型

根据调查区域的地理形状，调查航线一般分为平行断面型和“之”字型两种。“之”字型航线主要用于沿岸线呈狭长带状分布或环岛屿分布的生物资源调查。平行断面则适用于除调查区域特别复杂外的绝大部分海域，是航线设计的首选。平行断面又可分为等距平行断面、分区等距平行断面和分区随机平行断面三种形式。

G.1.1.1.3.2　等距平行断面

调查范围内所有调查断面均等间距布设。声学调查与环境调查同步进行时，一般选择等距平行断面。

G.1.1.1.3.3　分区等距平行断面

根据调查对象的密度分布特征，将整个调查范围划为若干分区；按调查对象的分布密度分配各分区内的断面数。各分区断面间距可以不同，但同分区内断面应等间距布设。

G.1.1.1.3.4　分区随机平行断面

将整个调查范围划为若干分区，各分区断面数预先设定，但同一分区内断面的起始位置则随机布设，一般由随机数字产生器（随机函数程序）确定。

G.1.1.1.4　断面间距

a）断面间距一般为10′～1° 经度、10′～1°纬度（约10 n mile～60 n mile）；

b）间距的选择取决于调查期限和调查精度的要求。当调查期限是主要限制因素时，应根据船速计算总允许航程来确定可能完成的断面数及相应的断面间距，同时为往返于港口与调查区域及生物学拖网取样和其他作业预留足够时间；

c）当调查精度为首要考虑因素时，可利用下式初步估算所需调查断面的总长度(D，n mile)：

$$D=\frac{\sqrt{A}}{4\cdot(CV)^2} \qquad \cdots\cdots(G.1)$$

式中：

A——调查范围的面积，单位为平方海里(n $mile^2$)；

CV——调查要求可接受的资源评估结果的变异系数(标准差与平均值的比值，精度的一种表示形式)。

G.1.1.1.5 **断面走向**

a）如果鱼类的分布在某一方向上存在明显的密度梯度，则调查断面应沿鱼类的密度梯度方向布设；

b）如果调查区域呈狭长带分布，则调查断面应平行于调查区域的短边；

c）如果调查期间鱼类存在定向洄游现象，则调查断面应平行于鱼类的洄游方向；

d）调查航线的起、止位置最好落于离陆基港较近的一侧。

G.1.1.1.6 **站位布设**

G.1.1.1.6.1 **定点布站与机动加站相结合**

在预设站位的基础上，根据映像的分布水层适量增设底层或变水层拖网取样站位。这种布站策略适用于群落结构和主要调查种类并重的调查。

G.1.1.1.6.2 **完全机动取样**

不预设取样站位，在调查过程中完全根据实时观测的声学映像进行生物学拖网取样。用于针对调查种类设计的调查。

G.1.1.1.7 **调查航速**

以 10 kn±2 kn 为宜。

G.1.1.2 **鱼类的目标强度**

目标强度是描述探测目标对声波的反射能力的一个物理量。它的基本表达形式为：

$$TS=20\log\frac{\sigma}{4\pi} \qquad \cdots\cdots(G.2)$$

式中：

TS——探测目标的目标强度，单位为分贝(dB)；

σ——探测目标的声学截面，单位为平方米(m^2)。

鱼类(或其他渔业生物)的目标强度是将回声积分值转换为绝对资源量的关键参数。因此在调查前应了解调查海域生物种类的目标强度；对未知目标强度的种类应力求在调查过程中对其进行现场测定或参考相似种类的目标强度。

G.1.2 **调查要素**

调查要素包括渔业资源的数量分布、洄游路线、种类组成、群体结构以及生物学特征等，进行资源量评估。

G.2 采样

G.2.1 主要工具与仪器设备

G.2.1.1 **调查船**

装有回声探测—积分系统、能进行底拖网和变水层拖网取样、自噪声较低的生物资源调查船。

G.2.1.2 **取样网具**

应使用选择性较低的专用调查网具，包括底层拖网和变水层拖网。

a）底层拖网：单拖网，四片式结构，网口 836 目×120 mm，囊网网目 24 mm(见图 G.1)。

b) 变水层拖网：单拖网，四片式结构，网口 1 000 目×400 mm，囊网网目 22 mm(见图 G.2)。

G.2.1.3 主要仪器设备

科研用回声探测-积分系统，视调查对象的不同，工作频率可选 18 kHz、38 kHz、70 kHz、120 kHz、200 kHz；声学仪器校正的成套工具，包括小型绞车、0.6 mm 的单丝尼龙线及标准校正球等；声学数据下载、存储及后处理系统；计算机、数据光盘刻录机或其他大容量数据存储媒介；彩色映像打印机；网具监测系统；导航定位仪、航速仪(计程仪)等。

G.2.1.4 声学仪器校正

在调查开始前及航次结束后采用标准球方法对回声探测-积分系统各进行一次声学校正。标准球的悬挂如图 G.3 所示，悬垂深度 10 m～30 m。单波束系统的校正内容主要为系统的积分增益，分裂波束系统的校正还包括目标强度增益与波束参数测定。

G.2.1.4.1 积分增益的测定

对稳定于声轴上的标准球进行积分，并将其测定值 $s_{A,m}$ 与理论值进行比较。标准球理论积分值的计算公式为：

$$s_{A,t} = \frac{4\pi \cdot \sigma_{st} \cdot 1\,852^2}{\psi \cdot R_{st}^2} \qquad \text{(G.3)}$$

式中：

$s_{A,t}$——标准球的理论积分值，单位为平方米每平方海里($m^2/n\ mile^2$)；

σ_{st}——标准球的声学截面，单位为平方米(m^2)；

ψ——换能器波束的等效立体角，以球面度表示(sr)；

R_{st}——标准球与换能器表面的距离，单位为米(m)；

$1\,852^2$——面积单位 m^2 与 n $mile^2$ 之间的转换系数。

如果 $s_{A,m}$ 与 $s_{A,t}$ 不同，则需对系统的积分增益进行修正，公式为：

$$G_n = G_o + \frac{10\log(s_{A,m}/s_{A,t})}{2} \qquad \text{(G.4)}$$

式中：

G_n——校正后的新增益，单位为分贝(dB)；

G_o——校正前的旧增益，单位为分贝(dB)。

G.2.1.4.2 SIMRAD 分裂波束式换能器目标强度增益与波束参数测定

通过调整三条悬挂尼龙线的长度，使标准球历经波束的每一位置。使用专业的 Lobe 程序计算目标强度增益及相关波束参数。

G.2.2 目标强度的测定

G.2.2.1 目标强度与体长关系式

对多数鱼类而言，其目标强度与体长关系可以下式表述：

$$TS = a\log l + b \qquad \text{(G.5)}$$

式中：

TS——鱼的目标强度，单位为分贝(dB)；

l——鱼体的长度，单位为厘米(cm)；

a、b——回归系数。

公式 G.5 需通过对不同体长鱼的目标强度进行测定后经回归分析确定。对多数梭型鱼类而言，a 一般在 20 左右，G.5 式可简化为：

$$TS = 20\log l + b_{20} \qquad \text{(G.6)}$$

式中：

b_{20}——G.5 式中斜率 a 预设为 20 时的截距 b 值。

G.2.2.2 目标强度的现场测定

G.2.2.2.1 测定条件与要求

鱼类离散分布且组成单一。需同时拖网取样以确认鱼种并进行各项生物学测定。

G.2.2.2.2 直接测定法

SIMRAD 分裂波束式回声探测-积分系统具有单体鱼目标强度测定功能，可利用其串行口输出的单体目标回波数据对渔业生物的目标强度进行现场直接测定。

G.2.2.2.3 间接测定法

利用计数-积分法或其他统计方法对可分辨为单体目标的种类的平均目标强度进行现场测定。

G.2.3 声学数据的采集

G.2.3.1 积分起始水层

起始积分水层至少应为换能器近场距离的两倍。换能器近场与远场分界面的距离 R_b(m)大约为：

$$R_b = \frac{a^2}{\lambda} \qquad \cdots\cdots (G.7)$$

式中：

a——换能器的直径或长边，单位为米(m)；

λ——波长，单位为米(m)。

常用工作频率 38 kHz 和 120 kHz 回声探测-积分系统的积分起始水层一般为 3 m～5 m。

G.2.3.2 积分终止水层

当水深＜1 000 m 时，积分至海底之上 0.5 m～1 m；

当水深＞1 000 m 时，积分至 1 000 m。

G.2.3.3 积分水层厚度

基本等间距设置。根据水深可选 5 m、10 m、20 m、50 m、100 m 或 200 m。

G.2.3.4 基本积分航程单元

当调查范围的尺度较小时选 1 n mile；当调查尺度是 5 n mile 的多倍时选 5 n mile。

G.2.4 生物学数据的采集

在预设站位及映像密集区投放底层或变水层拖网采集产生回波映像的生物样品。进行变水层拖网时应使用网具监测系统进行瞄准捕捞。根据映像密度情况作 10 min～60 min 有效拖曳，获取适量样品进行生物种类组成与各渔获种类的体长、体重组成分析。

G.2.5 观测记录

调查过程中值守人员应填写观测记录，记录表格如表 H.60 所示。表中基本积分航程单元为 5 n mile，每页记录对应的航程为 100 n mile，在航程栏第一空格处填写本页的起始航程(×××00)。表中时间、水深和经纬度等记录内容均指每一基本积分航程单元结束时刻的数据；调查信息栏则据情填写包括船舶行程信息、拖网信息、站位、海况、气象、现场渔业生产船动态以及其他可供映像分析参考的相关信息等。

G.3 数据处理

G.3.1 声学数据的预处理

排除偶尔出现的非生物来源回波信号，如气泡、不规则海底及本底噪声等，对原始积分值进行必要的修正。

G.3.2 生物学数据的预处理

统计、计算各网次渔获物中除底栖虾蟹类和鲆鲽类等非常贴底鱼种之外的所有鱼种和头足类的尾数、体长分布、平均体长、均方根体长、平均体重等数据，供积分值分配和生物量密度计算之用。

G.3.3 映像分析和积分值分配

以基本积分航程单元为单位进行映像分析和积分值判读。根据生物学取样资料和映像特征来鉴别

产生回波映像的目标生物种类，并将预处理后的总积分值(s_A)分配给对回声积分作出贡献的每一生物种类，计算公式如下：

$$s_{Ai} = \frac{s_A \cdot n_i \cdot \bar{\sigma}_i}{\sum_{i=1}^{k} n_i \cdot \bar{\sigma}_i} \quad \cdots\cdots\cdots\cdots (G.8)$$

式中：

s_{Ai}——第 i 种生物分配的积分值，单位为平方米每平方海里($m^2/n\ mile^2$)；

n_i——渔获物中第 i 种生物的尾数，单位为个(ind)；

$\bar{\sigma}_i$——第 i 种生物的平均声学截面，单位为平方米(m^2)；

k——渔获中参与积分值分配的生物种类的种数。

每种鱼类和头足类的平均声学截面由公式 G.2 和公式 G.5 或公式 G.6 算得。鱼类和头足类的目标强度与体长关系见表 G.1。

G.4 资源量评估

G.4.1 断面法

以断面观测值代表断面两侧各半个断面间距海域内的平均值。各断面所代表海域资源量之和即为调查范围内的总资源量。

某一给定断面所代表海域内评估种类的资源尾数(N,ind)和生物量(B,g)分别为：

$$N = \frac{\bar{s}_A \cdot D \cdot S}{\bar{\sigma}} \quad \cdots\cdots\cdots\cdots (G.9)$$

$$B = N \cdot \bar{w} \quad \cdots\cdots\cdots\cdots (G.10)$$

式中：

$\bar{s}_A$——断面内评估种类的平均积分值，单位为平方米每平方海里($m^2/n\ mile^2$)；

D——断面长度，单位为海里(n mile)；由断面起止经纬度算得；当纬度为 θ 时，一个经度的里程为 $60 \cdot \cos\theta$ n mile；

S——断面间距，单位为海里(n mile)；

$\bar{\sigma}$——断面内评估种类的平均声学截面，单位为平方米(m^2)；

$\bar{w}$——断面所代表海域内评估种类的平均体重，单位为克(g)。

G.4.2 方区法

将整个调查范围划分为若干小方区，以方区为单元进行计算，各方区内资源量之和即为调查范围内的总资源量。

某一给定方区内评估种类的资源尾数(N,ind)和生物量(B,g)分别为：

$$N = \frac{\bar{s}_A \cdot A}{\bar{\sigma}} \quad \cdots\cdots\cdots\cdots (G.11)$$

$$B = N \cdot \bar{w} \quad \cdots\cdots\cdots\cdots (G.12)$$

式中：

$\bar{s}_A$——方区内评估种类的平均积分值，单位为平方米每平方海里($m^2/n\ mile^2$)；

A——方区面积，单位为平方海里($n\ mile^2$)；

$\bar{\sigma}$——方区内评估种类的平均声学截面，单位为平方米(m^2)；

$\bar{w}$——方区内评估种类的平均体重，单位为克(g)。

采用分区法进行资源评估，当航线恰巧落在分区边界时，应预先约定航线上的观测值所代表的方区；同一观测值不能在不同方区内重复使用。

表 G.1 鱼类目标强度与体长关系参考值

种　类	关 系 式	备　注
鳀	$TS=20\log l-72.5$	l 为叉长
带鱼	$TS=20\log l-68.3$	l 为肛长
闭鳔鱼类	$TS=20\log l-68.0$	l 为体长
喉鳔鱼类	$TS=20\log l-72.5$	l 为体长
无鳔鱼类	$TS=20\log l-80.0$	l 为体长
枪乌贼	$TS=20\log l-80.0$	l 为胴长
注：TS 单位为分贝(dB)；l 单位为厘米(cm)。		

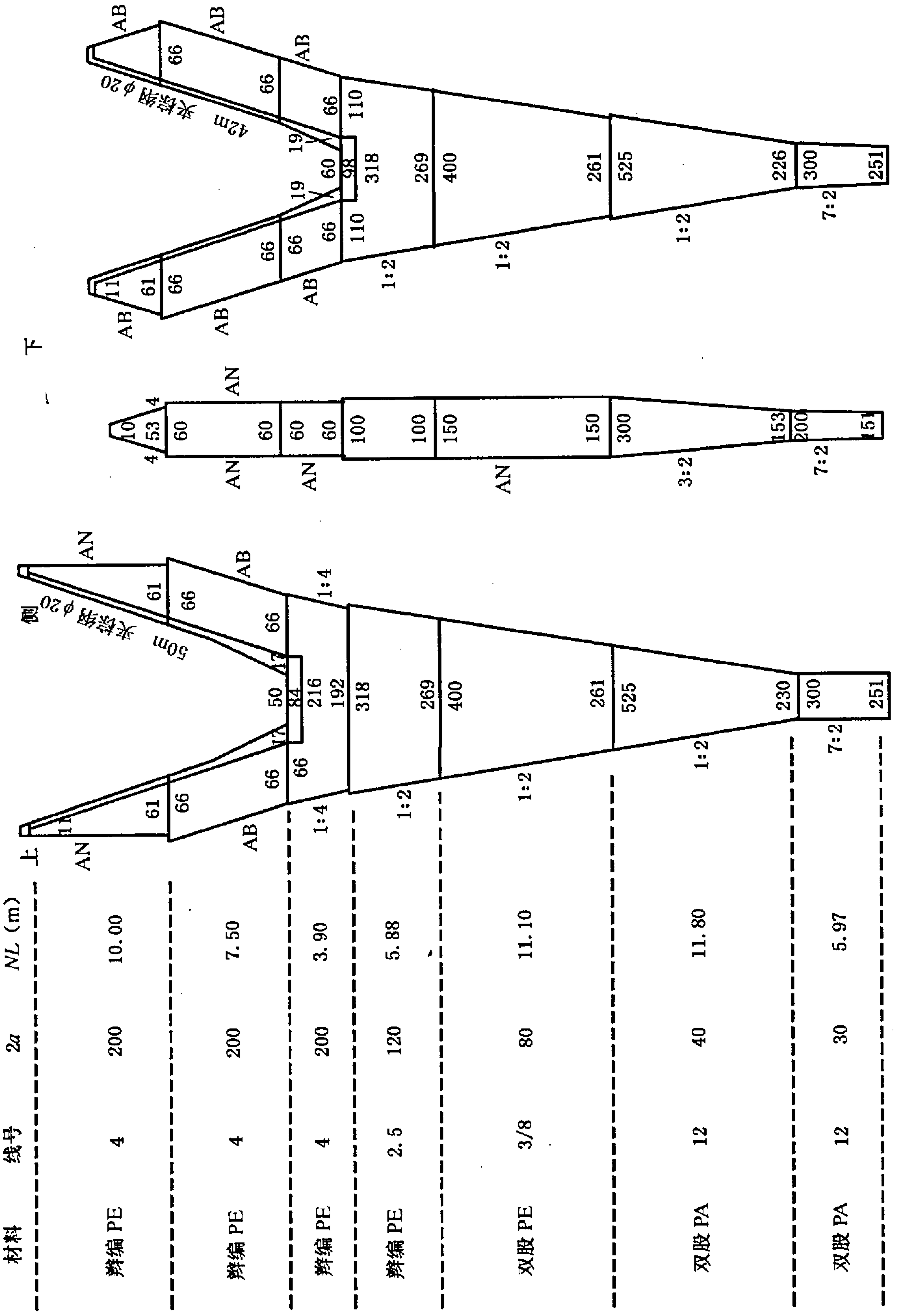

图 G.1 调查专用底层拖网网图

PA210	2a	◇	NL (m)
48×3	400	61	24.4
32×3	400	49.5	20.0
32×3	300	59.5	18.0
24×3	200	69.5	14.0
18×3	120	149.5	18.0
12×3	80	99.5	8.0
12×3	60	199.5	12.0
12×3	38	299.5	11.4

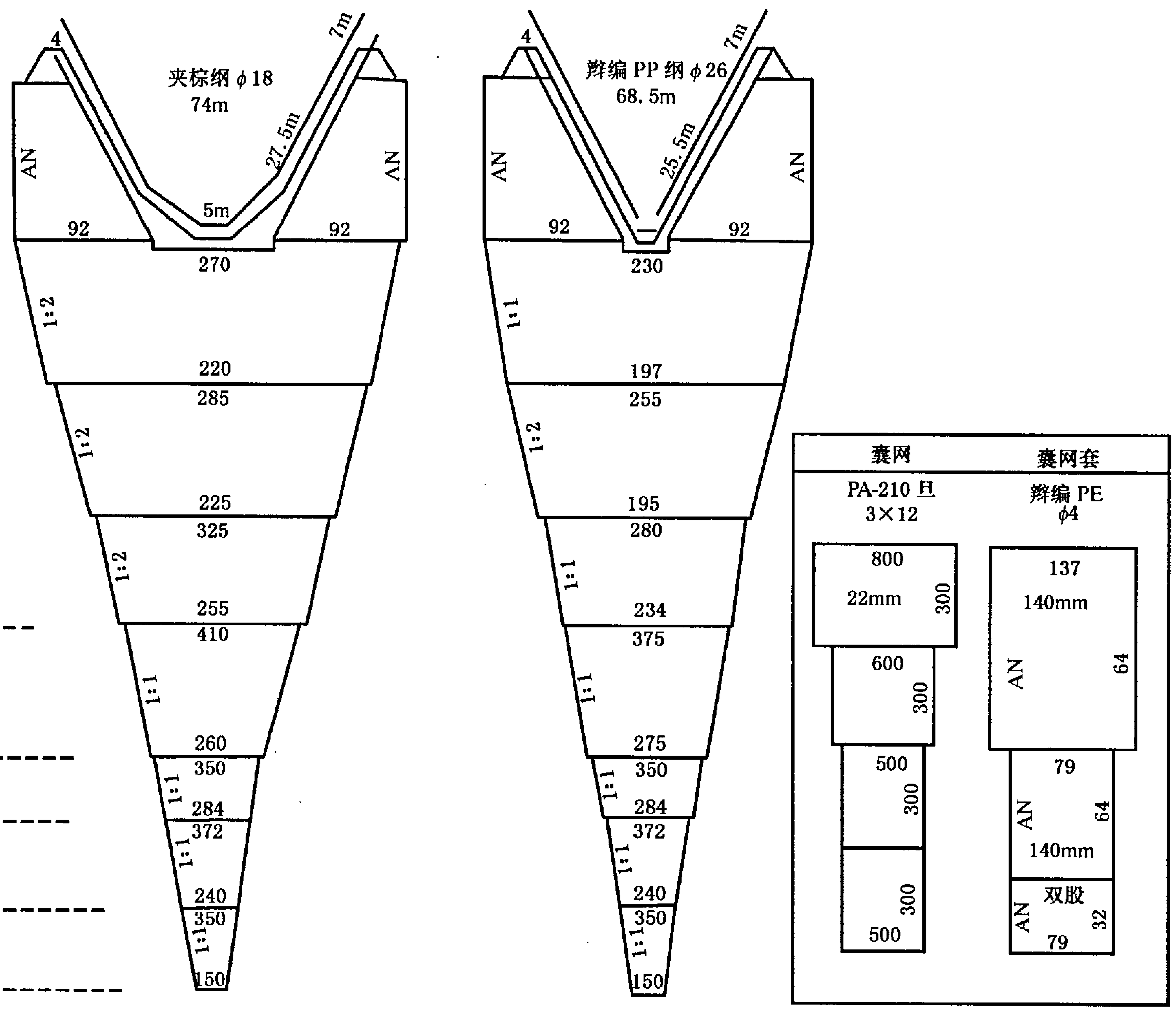

图 G.2 调查专用变水层拖网网图

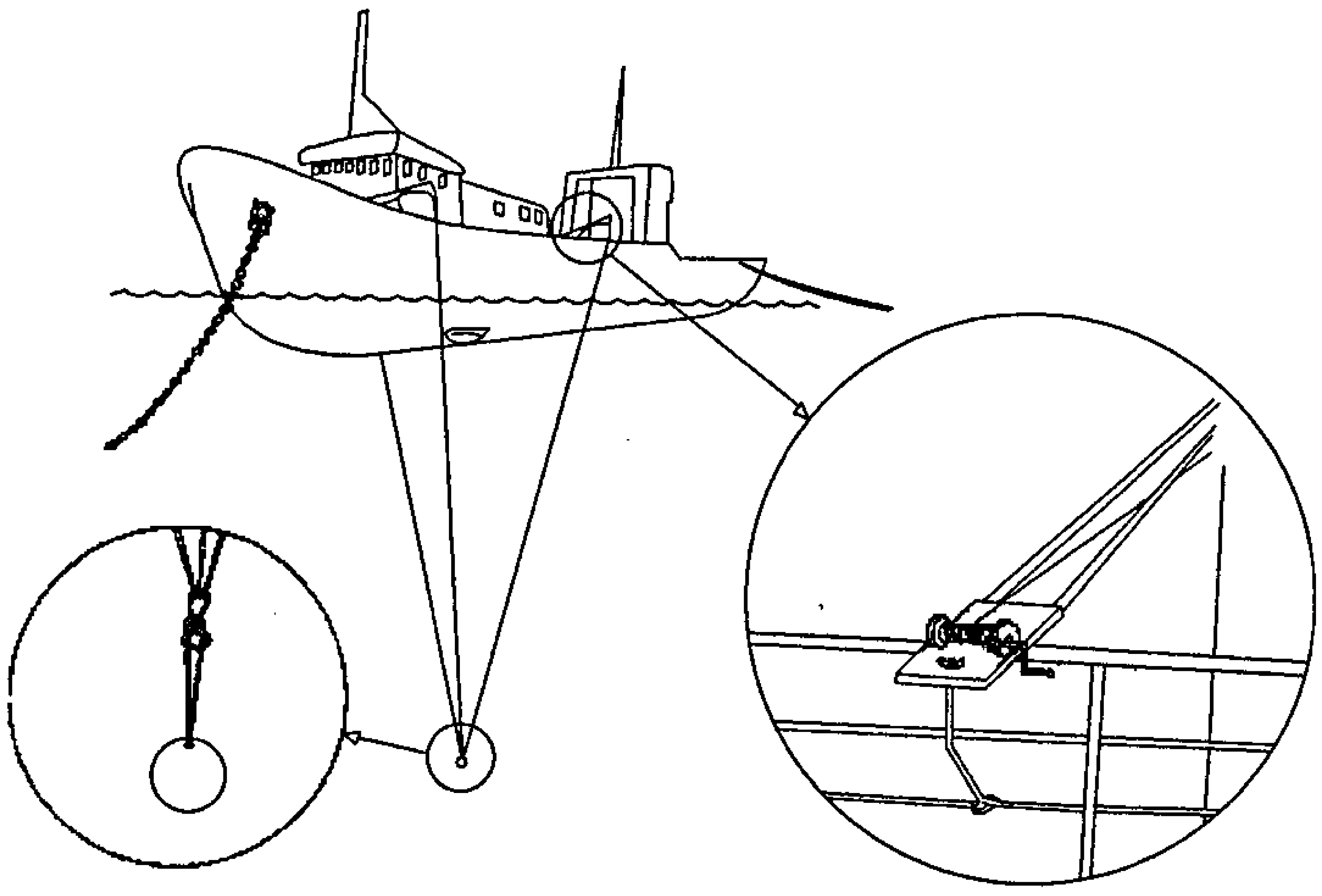

图 G.3　声学仪器校正标准目标的悬挂示意图

附 录 H
（资料性附录）
海洋生物调查采样和分析记录表格式

表 H.1～表 H.60 给出了海洋生物调查中样品采集、鉴定和分析记录表格式。

表 H.1 叶绿素采样记录表

共____页 第____页

海区________船名________航次________站号________实测站位经度________纬度________

水深________m 采样日期________年____月____日 透明度________m

水色________天气状况________海况________测量项目________测量方法________

滤膜类型________滤膜孔径________μm 抽气负压________kPa 储样条件________干燥器型号________

序号	预测深度<br>m	实测深度<br>m	采水时间<br>时：分	水样号	过滤时间<br>时：分	过滤水样量<br>dm^3	滤膜贮存号	备　注
1								
2								
3								
4								
5								
6								
7								
8								
9								
10								
11								
12								
13								
14								
15								
16								
17								
18								
19								
20								
21								
22								
23								
24								

采样________记录________过滤________校对________

表 H.2　叶绿素(萃取荧光法)测定记录表

共____页 第____页

海区__________ 船名______________________________ 航次__________ 站号______________________________

实测站位经度__________________________ 纬度______________________ 水深____________________________m

采样日期__________ 年________月________日 荧光计型号______________ 滤膜型号________________________

提取液体积____________________cm³ 测定日期______________年________月________日

空白测定	量程档										
	F01										
	F02										
	F0										
序号	实测深度 m	采样时间	过滤水样量 dm³	提取瓶号	量程档	换算系数 Fd	酸化前荧光读数 Rb	酸化后荧光读数 Ra	叶绿素 *a* 浓度 mg/m³	脱镁叶绿素 *a* 浓度 mg/m³	备注
1											
2											
3											
4											
5											
6											
7											
8											
9											
10											
11											
12											
13											
14											
15											
16											
17											
18											
19											
20											

测定__________________ 计算__________________ 校对__________________

表 H.3 叶绿素(分光光度法)测定记录表

共____页 第____页

海区____________ 船名____________ 航次____________ 站号____________

实测站位经度____________ 纬度____________ 水深____________m

采样日期______年______月______日 分光光度计型号____________ 比色皿光程____________cm

测定日期______年______月______日 提取液体积______cm^3 过滤水样体积______dm^3 提取时间______

序号	实测深度 m	滤膜贮存瓶号	离心管号	刻度试管号	光密度值				海水中叶绿素浓度 mg/m^3			备注
					OD 750	*OD* 664	*OD* 647	*OD* 630	ρ(Chl a)	ρ(Chl b)	ρ(Chl c)	
1												
2												
3												
4												
5												
6												
7												
8												
9												
10												
11												
12												
13												
14												
15												
16												
17												
18												
19												
20												

测定____________ 计算____________ 校对____________

表 H.4 叶绿素(高效液相色谱法)测定记录表

共____页 第____页

高效液相色谱仪型号__________ 色谱柱类型__________ 流动相速度($cm^3 \cdot min^{-1}$)__________

检测器类型__________ 型号__________ 测定波长__________ 测定日期______年______月______日

序号	样品号	滤膜号	提取液体积 V_{ext} cm^3	进样体积 V_{inj} mm^3	色谱峰面积 A	色素响应系数 F ($area/10^{-6}g$)	过滤水体积 V_{flt} cm^3	内标峰面积 A^{ca}	叶绿素 a 浓度 mg/m^3	备注
1										
2										
3										
4										
5										
6										
7										
8										
9										
10										
11										
12										
13										
14										
15										
16										

测定__________ 计算__________ 校对__________

表 H.5 初级生产力采样、过滤、测定记录表

共____页 第____页

海区________________ 船号________________ 航次______________ 站号____________

实测站位经度__________ 纬度__________ 水深__________m 水色__________ 透明度______________m

表面辐照度__________W/m^2 盐度__________ 温度__________ 采样日期________年________月________日

天气状况__________ 海况__________ 培养时间______日______时______分至________日________时________分

加入^{14}C量______________________

测定日期________年________月________日

序号	相对光强 %	预定深度 m	实测深度 m	采样时分	水样筒号	培养瓶号		过滤体积	贮存瓶号	初级生产力 mg/(m^3·h)	叶绿素a浓度 mg/m^3	生产力指数	备注
1						白瓶							
						黑瓶							
						零时							
2						白瓶							
						黑瓶							
						零时							
3						白瓶							
						黑瓶							
						零时							
4						白瓶							
						黑瓶							
						零时							
5						白瓶							
						黑瓶							
						零时							
6						白瓶							
						黑瓶							
						零时							

采样______________ 记录______________ 过滤______________ 校对______________

表 H.6 新生产力采样、过滤、测定记录表

共____页 第____页

海区________船号________航次________站号________实测站位经度________纬度________

水深________m 水色________透明度________m 温度________盐度________日期________年________月________日 天气状况________海况________

序号	采样水层 m	采样深度 m	采样深度温度 ℃	$[NO_3]$ μM	$[NH_4]$ μM	培养温度 ℃	培养开始时间	培养结束时间	培养瓶号		过滤体积	保存样品编号	a_d	a_p	$V_{[NO_3]}$ h^{-1}	$V_{[NH_4]}$ h^{-1}	f	初级生产力 $mg/(m^3 \cdot h)$	新生产力 $mg/(m^3 \cdot h)$	备注
1									白瓶											
									黑瓶											
									零时											
2									白瓶											
									黑瓶											
									零时											
3									白瓶											
									黑瓶											
									零时											
4									白瓶											
									黑瓶											
									零时											
5									白瓶											
									黑瓶											
									零时											
6									白瓶											
									黑瓶											
									零时											

采样________记录________过滤________校对________

表 H.7　微生物现场采样记录表

共____页 第____页

海区__________ 船名__________ 航次__________ 采样器__________
站号__________ 经度__________ 纬度__________ 水深__________m
采样时间______年______月______日______时______分 至______日______时______分

序号	样品类型	层次	样号	直接计数分样	培养计数分样	示踪测定分样	生态呼吸率分样	
				分样号	分样号	分样号	对照瓶号	测样瓶号
1								
2								
3								
4								
5								
6								
7								
8								
9								
10								
11								
12								
13								
14								
15								
16								
17								
18								
19								
20								
21								
22								
23								
24								
记 事：								

采样__________ 记录__________ 校对__________

表 H.8 海洋水体病毒、细菌数量直接计数记录表

共____页 第____页

海区______________ 船名______________ 航次______________ 采样器______________ 计数方法______________

站号	样号	最大水深 m	水层 m	采样时间	计数时间	加样量 cm^3	各视野病毒数(particles)或细菌数(cells)								平均 particles 或 cells	总病毒数 (particles/dm^3) 或总菌数(cells/dm^3)	备 注

记事：

分析______________ 记录______________ 校对______________

表 H.9 细菌菌体大小测定记录表

共____页 第____页

海区________________ 船名________________ 航次________________

站号		样号		站号		样号	
照片号	菌体序号	菌体长(*L*)<br>mm	菌体直径(*W*)<br>mm	照片号	菌体序号	菌体长(*L*)<br>mm	菌体直径(*W*)<br>mm

台微尺标定显微照片放大倍数							
	1	2	3	4	5	6	放大倍数
实值(μm)							
测值(mm)							

记事：

测量________________ 记录________________ 校对________________

表 H.10　水样、泥样微生物培养计数和菌株分离记录表

共____页 第____页

海区________ 船名________ 航次________ 采样时间________年____月____日

站号	样号	层次	样品类型	底质样		培养基	培养温度 ℃	稀释度	皿、膜号	菌落数 CFU					菌数 CFU/dm^3 或 CFU/g	分离菌株	备　注
				样量 g	稀释水 cm^3					*d*	*d*	*d*	*d*	平均			

记事：

分析________ 记录________ 校对________

表 H.11 水样细菌生产力记录表

共____页 第____页

海区______________ 船名______________ 航次______________ 示踪方法______________

站号	水样号	水层<br>m	水温<br>℃	水样量<br>dm^3	培养时间<br>自 时 分<br>至 时 分	培养<br>温度<br>℃	样品<br>或<br>空白	培养<br>时数<br>h	闪烁<br>瓶号	Bq 值	样品<br>实际<br>Bq	加入放射<br>性剂量<br>Bq	放射性<br>比活度<br>Bq/mmol	吸收率<br>$mmol/(dm^3 \cdot h)$	细菌生产力<br>cells 或<br>$\mu g/(dm^3 \cdot h)$
闪烁计数仪型号						备注									

分析______________ 记录______________ 校对______________

表 H.12 水样细菌异养活性测定记录表

共____页 第____页

海区________ 船名________ 航次________

站号	样品号	水层<br>m	水温<br>℃	水样量<br>cm^3	培养时间<br>自 时 分<br>至 时 分	培养温度<br>℃	样品或空白	培养时数<br>h	闪烁瓶号	Bq 值	样品实际 Bq 值	加入放射性量 Bq 值	加入非放射性葡萄糖量 μg	葡萄糖吸收量<br>$\mu g/(dm^3 \cdot h)$	备注
闪烁计数仪型号						备注									

分析________ 记录________ 校对________

表 H.13 样品细菌最可能数(MPN)记录表

共____页第____页

海区______________ 船名______________ 航次______________

站号	样号	层次	样品类型	底质样		对象菌（群）名称	培养基	培养温度 ℃	稀释度	各浓度阳性管数										菌数 CFU/dm^3 或 CFU/g	分离菌株
				样量 g	稀释水 cm^3					*d*					*d*						
记事：																					

分析______________ 记录______________ 校对______________

表 H.14　浮游生物海上采样记录表

共____页 第____页

海区__________ 船名__________ 航次__________ 站号__________ 实测站位 经度__________ 纬度__________

水深________ m 采样时间________年______月______日______时______分至______日________时________分

<table>
<tr><th colspan="2" rowspan="2">采样项目</th><th rowspan="2">瓶号</th><th rowspan="2">绳长
m</th><th colspan="2">倾角
(°)</th><th colspan="2">流量计</th><th rowspan="2">备　注</th></tr>
<tr><th>开始</th><th>终了</th><th>编号</th><th>转数
r</th></tr>
<tr><td rowspan="5">拖
网</td><td>网</td><td></td><td></td><td></td><td></td><td></td><td></td><td></td></tr>
<tr><td>网</td><td></td><td></td><td></td><td></td><td></td><td></td><td></td></tr>
<tr><td>网</td><td></td><td></td><td></td><td></td><td></td><td></td><td></td></tr>
<tr><td>网</td><td></td><td></td><td></td><td></td><td></td><td></td><td></td></tr>
<tr><td>网</td><td></td><td></td><td></td><td></td><td></td><td></td><td></td></tr>
<tr><td rowspan="7">采
水</td><td></td><td></td><td></td><td></td><td></td><td colspan="2">采水量
cm^3</td><td></td></tr>
<tr><td>层</td><td></td><td></td><td></td><td></td><td colspan="2"></td><td></td></tr>
<tr><td>层</td><td></td><td></td><td></td><td></td><td colspan="2"></td><td></td></tr>
<tr><td>层</td><td></td><td></td><td></td><td></td><td colspan="2"></td><td></td></tr>
<tr><td>层</td><td></td><td></td><td></td><td></td><td colspan="2"></td><td></td></tr>
<tr><td>层</td><td></td><td></td><td></td><td></td><td colspan="2"></td><td></td></tr>
<tr><td>层</td><td></td><td></td><td></td><td></td><td colspan="2"></td><td></td></tr>
<tr><td colspan="9">海况：</td></tr>
</table>

采样______________________ 记录______________________校对______________________

表 H.15 浮游生物垂直分层拖网采样记录表

共____页 第____页

海区__________ 船名__________ 航次__________ 站号__________

实测站位经度__________ 纬度__________ 水深__________ m 网型__________

采样时间自______年______月______日______时______分至______日______时______分

采样层次	瓶号	绳长 m		倾角 (°)		备注
		放出	闭锁	上升时	闭锁时	
海况：						

采样__________ 记录__________ 校对__________

表 H.16　浮游生物样品登记表

共____页 第____页

海区________________________ 船名________________________ 航次________________________

样品编号	海上瓶号	站号	日期	时间	水深 m	层次	倾角 (°)		绳长 m	流量计		滤水量 m^3	备注
							开始	终了		转数 r	标定值 m^3/r		

编号________________________ 校对________________________

表 H.17 微微型光合浮游生物细胞数量记录表

共____页 第____页

海区__________ 船名__________ 航次__________ 站号__________

实测站位经度____________________ 纬度____________________

采样时间______ 年______ 月______ 日______ 时 至______ 日______ 时

水样号______ 水深______ m 水层______ m 水温______ ℃ 采样器______

记录要素	聚球藻 (Synechococcus)						微微型光合真核生物 (Euk)		
	藻红蛋白 (PE)			藻蓝蛋白(PC)					
过滤面积 (cm^2)									
视野面积 (cm^2)									
过滤水样量 (cm^3)									
各视野细胞数									
平均值									
每毫升水样细胞数									

采样__________ 分析__________ 记录__________ 校对__________

表 H.18 微型和小型浮游生物标本个数计数记录表

共____页 第____页

海区______________ 船名______________ 航次______________

样品编号______________ 站号______________ 水层(或绳长)__________ m 采样日期______________

浓缩体积________ cm^3 计数体积________ cm^3 计数日期________滤水(或采水)量________ m^3 ________(dm^3)

种 名	数 量	小 计	密度 cells/dm^3 或 cells/m^3	备 注
总 计				
注:本表适用于浓缩计数法的记录,网采样品单位为个/m^3,采水样品单位为个/dm^3。				

计数______________ 校对______________

表 H.19 采水浮游生物标本个数计数记录表

共____页 第____页

海区______________ 船名______________ 航次______________ 站号______________ 水深__________ m

样品编号________ 采样日期________ 计数日期________计数体积________ cm^3 层次__________ m

种　　名	数　　量			小计	密度 cells/cm^3 或 cells/dm^3	备　　注
	第一分样	第二分样	第三分样			
总　　计						

注：本表适用于沉降计数法的记录。

计数______________________________ 校对______________________________

表 H.20　浮游植物(孢囊)细胞记录表

共____页 第____页

海区________________ 船名________________ 航次________________

站号__________ 水层__________ m 采样时间________________ 滤水量__________ m^3

浓缩体积__________ cm^3 计数体积__________ cm^3 计数日期________________

种　名	数　量	小　计	密度<br>$cells/cm^3$,$cells/dm^3$ 或 $cells/m^3$
甲藻总计			
硅藻总计			
其他			
总计			

计数________________ 校对________________

表 H.21　浮游动物体积分数测定记录表

共____页 第____页

海区________________ 船名________________ 航次________________

采样日期________________ 测定日期________________

样品编号	站号	海水+动物体积 cm^3	海水体积 cm^3	动物体积 cm^3	滤水量 m^3	体积分数 10^{-6}	备　注

测定________________计算________________校对________________

表 H.22 浮游动物湿重生物量测定记录表

共____页 第____页

海区________________ 船名________________ 航次________________

采样日期________________ 测定日期________________

样品编号	站号	总重量<br>mg	筛绢湿重<br>mg	动物湿重<br>mg	滤水量<br>m^3	湿重生物量<br>mg/m^3	备　　注

测定________________ 计算________________ 校对________________

表 H.23　浮游动物干重生物量测定记录表

共____页 第____页

海区______________ 船名______________ 航次______________

采样日期______________ 测定日期______________

样品编号	站号	总重量 mg	筛绢干重 mg	动物干重 mg	滤水量 m^3	干重生物量 mg/m^3	备　注

测定______________ 计算______________ 校对______________

表 H.24 浮游动物个体计数记录表

共____页第____页

样品编号____________站号____________水深____________m 绳长____________m 水层____________m

滤水量____________m^3 取样____________采样日期____________计数日期____________

种 名	数 量	全网个数	密度 ind/m^3	备 注
种数　　　　总计				

计数____________统计____________校对____________

表 H.25 夜光藻个体计数记录表

共____页 第____页

海区______________ 船名______________ 航次______________

采样日期______________ 测定日期______________

样品编号	站号	滤水量 m^3	取样 %	数量	全网个数	密度 cells/m^3	备注

计数______________ 统计______________ 校对______________

表 H.26　鱼类浮游生物海上采集记录表

共____页 第____页

海区____________ 站号____________ 水深__________ m　船名____________

实测站位经度______________________________ 纬度______________________

采集时间自______年______月______日______时______分至______月______日______时______分

采集项目		瓶号	绳长<br>m	倾角<br>(°)		流量计		备注
				开始	终了	编号	转数<br>r	
垂直或斜拖	网							
	网							
	网							
	网							
水平拖曳				拖网时间 min				
定性样品<br>（网型、层次）								
海况								

采集____________________ 记录____________________ 校对____________________

表 H.27 鱼类浮游生物标本登记表

共____页 第____页

调查海区____________________ 船名____________________ 日期____________________

标本编号	海上瓶号	站号	日期	时间	水深 m	层次	倾角(°)		拖网时间	绳长 m	流量计		滤水量 m^3	流量计校对值	备注
							开始	终了			转数 r	标定值 m^3/r			

编号____________________ 计算____________________ 校对____________________

表 H.28 鱼类浮游生物计数记录表

共____页第____页

标本编号____________ 站号____________ 水深____________ m 绳长____________ m 水层____________

取样____________ 滤水量____________ m³ 采样日期________________ 计数日期________________

种名	发育阶段	数量 ind	全网个数 ind	密度 ind/m³	备注
总计	鱼卵				
	仔、稚鱼				

计数________________ 校对________________

表 H.29 鱼类浮游生物数量统计表

共____页 第____页

站号													
采集时间	日期												
	时间												
拖网层次													
拖网时间 min													
种类		密度 ind/m³											
总计	鱼卵												
	仔、稚鱼												

记录____________ 校对____________

表 H.30 大型底栖生物海上采样记录表

共____页 第____页

海区________ 船名________ 航次________ 站号________ 编号________

经度________ 纬度________ 水深________ m 放绳长度________ m 底质________

底温________℃ 底盐________ 采泥器________ m^2 采泥次数________ 样品厚度________ cm

网型________ 网宽________ m 拖网距离________

采泥时间________ 年________月________日________时________分

拖网时间________ 年________月________日________时________分 至________时________分 计________分

采泥样品总数		拖网样品总数		
优势种类记录				
序号	种　名	总个数<br>ind	取回个数<br>ind	备　注
1				
2				
3				
4				
5				
6				
7				
8				
9				
10				
11				
12				
13				
14				
15				
16				
17				
18				
记事：				

采样________ 记录________ 校对________

表 H.31 大型底栖生物定量分析记录表

共____页 第____页

海区______________ 船名________________ 航次______________ 站号_________________编号_________

水深______________ m 底质__________________ 采泥器________________ m^2 采样次数______________

采样厚度______________ 采样时间_________ 年_________ 月_________ 日_________ 时_________ 分

序号	种名	个体数 ind	密度 ind/m^2	重量 g	生物量 g/m^2	
					湿重	干重
1						
2						
3						
4						
5						
6						
7						
8						
9						
10						
11						
12						
13						
14						
15						
16						
17						
18						
19						
20						
21						
22						
23						
24						

采样________________ 称重________________ 计算________________校对________________

表 H.32 大型底栖生物定性分析记录表

共____页 第____页

海区__________ 船名__________ 航次__________ 站号__________ 编号__________

水深__________m 底质__________ 网型__________ 网宽__________m 拖网距离__________m

拖网时间__________年______月______日______时______分 至______时______分 计______分

序号	种　　名	个体数<br>ind	备　　注
1			
2			
3			
4			
5			
6			
7			
8			
9			
10			
11			
12			
13			
14			
15			
16			
17			
18			
19			
20			
21			
22			
23			
24			

采样__________ 记录__________ 校对__________

表 H.33 大型底栖生物定量分析种类分布记录表

共____页 第____页

海区____________________ 船名____________________ 航次________

科名____________________ 种名________________________________

站号	标本号	采样日期	分布密度		生物量 g/m^2		深度 m	底温 ℃	底盐	底质	备注
			个体数 ind	密度 ind/m^2	湿重	干重					

鉴定____________________ 记录____________________ 校对____________________

表 H.34 大型底栖生物定性分析种类分布记录表

共____页 第____页

海区________________ 船名________________ 航次________

科名________________ 种名________________

站号	标本号	采样日期	个体数<br>ind	深度<br>m	底温<br>℃	底盐	底质	备 注

鉴定________________ 记录________________ 校对________________

表 H.35 小型底栖生物海上采样记录表

共____页 第____页

海区________ 船名________ 航次________ 编号________

站号________ 实测站位经度________ 纬度________

水深________m 底质________ 表温________℃ 底温________℃

表盐________底盐________ 表氧________% 底氧________%

采泥器________m^2 类型________ 采样厚度________cm 取样管类型________

内径________cm 采样时间________年________月________日________时________分

类别	芯样号	分层				
		0～2 cm	2～5 cm	5～10 cm	＞10 cm	
		瓶样号				
小型生物	01					
	02					
	03					
	04					
	05					
沉积物叶绿素和有机碳	06					
	07					
	08					
记事：						

采样________记录________校对________

表 H.36 小型底栖生物定量分析记录表 1

共____页 第____页

海区________ 船名________ 航次________ 站号________ 编号________

实测站位 经度________ 纬度________ 水深________m

采泥器________m^2 类型________ 样品厚度________cm 底质________

采样时间________ 年________ 月________ 日________ 时________分

类群	芯样号	分层									
		0～2 cm	2～5 cm	5～10 cm	>10 cm				个体数 ind	密度 ind/m^2	备注
		瓶样号									
线虫	01										
	02										
	03										
	平均										
桡足类	01										
	02										
	03										
	平均										
介形类	·01										
	02										
	03										
	平均										
	01										
	02										
	03										
	平均										
	01										
	02										
	03										
	平均										
总数量											

注：各类群所占百分比为线虫 %，桡足类 %，介形类 %，其他 %

记事：

分选________ 记录________ 校对________

表 H.37 小型底栖生物定量分析记录表 2

共____页 第____页

海区____________ 船名____________ 航次____________ 站号____________ 编号____________

经度________ 纬度__________ 水深________ m 底质________ 采泥器__________ m^2 类型__________

样品厚度____________ cm 取样称重时间________ 年________月________日________时________ 分

类群	样品序号	第一次称重(μg)				第二次称重(μg)				第三次称重(μg)				个体平均μg	生物量μg/m^2
		称重数量	总重量	称皿重	平均重	称重数量	总重量	称皿重	平均重	称重数量	总重量	称皿重	平均重		
线虫	1														
	2														
桡足类	1														
	2														
介形类	1														
	2														
	1														
	2														
	1														
	2														
其他	1														
	2														
小型动物															

分析____________________ 校对____________________

表 H.38 小型底栖生物定性分析记录表

共____页 第____页

海区________________船名______________航次______________站号______________编号______________

实测站位经度________________纬度________________水深______________m 底质______________

采泥器______________m^2 类型______________拖网网型______________拖网时间______________至____

采样时间______________年______________月______________日______________时______________分

类群	序号	种名	数量<br>ind	备注

分选________________________鉴定________________________

表 H.39 潮间带生物野外采集记录表

共____页 第____页

项目编号______________ 地点______________ 断面______________ 站号__________样方号______________

潮带______________ 底质______________ 取样面积______________ m^2 样品厚度______________cm

气温______________℃ 水温______________℃ 底温______________℃ 气象______________

采样日期__________ 年__________月__________日

定量标本瓶数：		定性标本瓶数：		
次序	种名或类群	个数<br>ind	覆盖面积<br>cm^2	备注
1				
2				
3				
4				
5				
6				
7				
8				
9				
10				
11				
12				
13				
14				
15				
16				
记事：				

采集______________________ 记录______________________ 校对______________________

表 H.40 潮间带生物定量分析记录表

共____页 第____页

项目编号________ 地点________ 断面________ 站号________ 样方号________

潮区________ 底质________ 取样面积________ m^2 样品厚度________ cm

采样日期________ 年________ 月________ 日

序号	种 名	个体数 ind	密 度 ind/m^2	重量 g	生物量 g/m^2	备注
1						
2						
3						
4						
5						
6						
7						
8						
9						
10						
11						
12						
13						
14						
15						
16						
17						
18						
19						
20						
21						
22						
23						
24						
合 计						

鉴定________ 称重________ 计算________ 校对________

表 H.41 潮间带生物定性分析记录表

共____页 第____页

项目编号____________ 地点____________ 断面____________ 站号________ 样方号____________

潮区________________ 底质______________ 取样面积____________ m² 样品厚度__________ cm

采样日期________ 年________ 月________ 日

序号	种　　名	个体数<br>ind	备　　注
1			
2			
3			
4			
5			
6			
7			
8			
9			
10			
11			
12			
13			
14			
15			
16			
17			
18			
19			
20			
21			
22			
23			
24			

鉴定________________________ 填表________________________ 校对________________________

表 H.42 潮间带生物种类分布表

共____页 第____页

科________________ 种名________________

地点	断面	潮区	站号	标本号	采样日期	生物量 g/m^2	密度 ind/m^2	定性标本状况	体长 cm	底质

鉴定________ 记录________ 校对________

表 H.43 潮间带生物主要种类垂直分布表

共____页 第____页

海区________________ 断面________________ ______年______月______日

序号	站号	1		2		3		4		5		6		7		备注
	种名	生物量 g/m²	密度 ind/m²	生物量 g/m²	密度 ind/m²	生物量 g/m²	密度 ind/m²	生物量 g/m²	密度 ind/m²	生物量 g/m²	密度 ind/m²	生物量 g/m²	密度 ind/m²	生物量 g/m²	密度 ind/m²	
1																
2																
3																
4																
5																
6																
7																
8																
9																
10																
11																
12																
13																
14																
15																
16																
17																
18																

记录________________ 校对________________

表 H.44 潮间带生物统计表

共____页第____页

海区________ 断面________

潮区	藻类		多毛类			软体动物			甲壳动物			棘皮动物			其他动物		
	生物量 g/m²	种数	生物量 g/m²	密度 ind/m²	种数	生物量 g/m²	密度 ind/m²	种数	生物量 g/m²	密度 ind/m²	种数	生物量 g/m²	密度 ind/m²	种数	生物量 g/m²	密度 ind/m²	种数

记录________ 校对________

表 H.45 微型污损生物记录表

共____页 第____页

海区__________ 挂板位置__________ 水深__________ m 水温__________℃

潮汐__________ 挂板日期__________ 取板日期__________ 板别__________

序号	试板表面粘膜特征	干重<br>mg/cm²	细菌附着量<br>ind/cm²	硅藻附着量<br>ind/cm²	主要种类及<br>其附着特征	备注

采样__________ 记录__________ 校对__________

表 H.46 船舶污损生物记录表

共____页 第____页

船名______________ 吨位______________ 进坞港口______________ 航线______________ 航速__________

两次坞修间隔________________________ 涂层种数和道数________________________ 取样时间__________

部位	厚度<br>mm	覆盖面积率<br>%	取样面积<br>cm^2	样品号	备注
水线					
前侧					
中侧					
后侧					
底					
螺旋桨					
舵					
记事：					

采集________________________ 记录________________________ 校对________________________

表 H.47 浮标污损生物记录表

共____页 第____页

海区______________ 浮标名称______________ 浮标位置 经度______________纬度____________________

下水时间________________________ 取样时间________________________ 涂层______________

部位	厚度<br>mm	覆盖面积率<br>%	取样面积<br>cm^2	样品号	优势种
水线					
体侧					
底					
尾外					
尾内					
锚链					
沉块					
记事：					

取样________________________ 记录________________________ 校对________________________

表 H.48 码头、桩柱污损生物记录表

共____页 第____页

海区________________ 地址________________ 码头名称________________

码头质地____________ 最大潮差____________ 建成时间____________ 取样时间____________

潮区<br>m	厚度<br>mm	覆盖面积率<br>%	取样面积<br>cm^2	样品号	优势种
高					
中					
低					

种类垂直分布	种　　名	分布范围<br>cm	密集范围<br>cm

记事：

取样________________ 记录________________ 校对________________

表 H.49　污损生物分析记录表

共____页 第____页

海区________________ 站名________________ 取样时间________________

板别________________ 下板时间________________ 计样面积________________cm^2

样品号	类别	种名	个体数 ind	密度 ind/m^2	附着面积%			标本湿重 g	附着湿重 g/m^2	备注
					左	右	平均			
平均厚度　mm;覆盖面积率　%;合计　ind/m^2					%			g/m^2		
群落特点:										

分析________________ 统计________________ 校对________________

表 H.50　污损生物种类记录表

共____页 第____页

海区____________ 中文名____________ 拉丁名____________

序号	标本号	个体数<br>ind	密度<br>ind/m²	附着面积率<br>%	标本湿重<br>g	附着湿重<br>g/m²	备注
记事：							

记录____________ 校对____________

表 H.51　游泳动物拖网卡片

共____页 第____页

海区__________ 船名__________ 航次__________ 站号__________ 拖网号次__________ 日期__________

风向__________ 风力__________ 天气__________ 气压__________ 气温__________℃ 表层水温______℃

网具类型______________________ 规格______________________ 囊网网目尺寸______________mm

放网:时间________位置________N(S)________E(W)渔区________水深________m 拖速________kn 拖向______

起网:时间________位置________N(S)________E(W)渔区________水深________m 拖速________kn 拖向______

曳纲长度______________m 拖网时间______________________h 渔捞事故______________________

总渔获量______________________kg(估计)______________kg(准确) 平均______________________kg/h

样品标本号______________________________ 样品重量______________________________kg

探鱼仪映像____

其他记事：____

种　类　组　成

种　类	全部或部分取样样品				全　网　渔　获　量				备注
	重量 g	尾数 ind	体长范围 mm	体重范围 g	重量 g	尾数 ind	重量 kg/h	尾数 ind/h	

记录______________________ 校对______________________

表 H.52 鱼类生物学测定记录表

共____页 第____页

海区________船名________航次________站号________渔区________实测站位________ N(S)__________ E(W)

种名________________ 水深________________ m 采样时间________________ 网具________________ 渔获量____kg

编号	长度 mm		重量 g				性别			性腺成熟度（期）	性腺成熟系数 10^{-3}	摄食强度（级）	摄食饱满系数 10^{-3}	含脂量（级）	年龄	备注
	全长	体长	鱼体重	纯体重	性腺重	胃肠重	♀	♂	⚥							

测定________________ 记录________________ 校对________________ 测定日期__________ 年______月______日

表 H.53 虾类生物学测定记录表

共____页 第____页

海区________ 船名________ 航次________ 站号________ 渔区________

实测站位________ N(S)________ E(W)种名________ 水深________m

采样时间________ 网具________ 渔获量________kg

编号	长度 mm		重量 g		性别		性腺成熟度（期）	性腺成熟系数 ‰	摄食强度（级）	摄食饱满系数 ‰	已交配（√）	备注
	体长	头胸甲长	体重	性腺重	♀	♂						

测定________ 记录________ 校对________ 测定日期________年________月________日

表 H.54 蟹类生物学测定记录表

共____页 第____页

海区____________ 船名______________ 航次____________ 站号____________ 渔区__________

实测站位____________________ N(S)____________________ E(W)种名____________________

水深____________ m 采样时间______________ 网具______________ 渔获量______________ kg

序号	头胸甲 mm		腹部 mm		重量 g		性别		性腺成熟度（期）	性腺成熟系数 ‰	摄食强度（级）	摄食饱满系数 ‰	备注
	长度	宽度	长度	宽度	体重	性腺	♀	♂					

测定__________ 记录__________ 校对__________ 测定日期________ 年________月________日

表 H.55 头足类生物学测定记录表

共____页 第____页

海区__________ 船名__________ 航次__________ 站号__________ 渔区__________

实测站位__________ N(S)__________ E(W)种名__________

水深__________m 采样时间__________ 网具__________ 渔获量__________kg

编号	长度 mm			重量 g			性别		性腺成熟度（期）	性腺成熟系数 ‰	摄食强度（级）	摄食饱满系数 ‰	备注
	胴长			总体重	纯体重		♀	♂					

测定__________ 记录__________ 校对__________ 测定日期__________年__________月__________日

表 H.56　游泳动物数量统计表

共____页 第____页

海区________ 航次________ 船名________ 作业方式________ 网型________

调查时间________年________月________日至________年________月________日

网序	月	日	站位		时间		拖网时间 h	底层水温 ℃	总渔获量		主要种类渔获数量					
			纬度	经度	放网	收网										
			放网	放网					重量	尾数	重量	尾数	重量	尾数	重量	尾数
			起网	起网					kg/h	ind/h	kg/h	ind/h	kg/h	ind/h	kg/h	ind/h
备注	实际操作时，应以 kg、ind、kg/h 和 ind/h 为单位各自独立列成一个表格进行统计。															

统计________ 校对________ ________年________月________日

表 H.57 鱼类体长测定统计表

共____页 第____页

海区______________ 船名________________________ 航次________________ 站号________________

渔区______________ 实测站位______________ N(S)______________ E(W)水深______________________m

种名________________________采样时间______________ 网具______________ 渔获量______________kg

体长组 mm	年龄												共计			各体长组	
	♀	⚥	♂	♀	⚥	♂	♀	⚥	♂	♀	⚥	♂	♀	⚥	♂	总尾数	%
小计																	
各年龄组总尾数																	
各年龄组占总尾数 %																	

计算________________ 校对________________ __________年__________月________日

表 H.58 鱼类体重测定统计表

共____页 第____页

海区______________ 船名________________________ 航次_________________ 站号_________________

渔区______________ 实测站位______________ N(S)______________ E(W) 水深____________________m

种名________________________ 采样时间______________ 网具______________ 渔获量______________kg

体重组<br>g	年龄												共计			各体重组	
	♀	♂	⚥	♂	♀	⚥	♀	♂	⚥	♂	♀	⚥	♀	♂	⚥	总尾数	%
小计																	
各年龄组总尾数																	
各年龄组占总尾数<br>%																	

计算________________ 校对________________ ____________年____________月__________日

表 H.59 鱼类怀卵量记录表

共____页 第____页

海区________________ 船名________________ 航次________________ 站号________

渔区________________ 实测站位________________ N(S)____________ E(W) 水深____________m

种名________________ 采样时间________________ 网具________________ 渔获量____________kg

编号	长度 mm	重量 g		年龄	成熟度（期）	性腺重量 g	取样重量 g	绝对怀卵量（个）		相对怀卵量（个）		备注
		总重	纯重					取样卵数	全部卵数			

测定____________ 记录____________ 校对____________ 测定日期________ 年______月______日

表 H.60 声学调查观测记录表

共____页 第____页

海区______________ 调查船______________ 调查代码______________ 日期______________

时间	航程	水深<br>m	总积分值<br>(m^2/n $mile^2$)	位置		调查信息
				纬度	经度	
	5					
	10					
	15					
	20					
	25					
	30					
	35					
	40					
	45					
	50					
	55					
	60					
	65					
	70					
	75					
	80					
	85					
	90					
	95					

记录______________ 校对______________

参 考 文 献

[1] GB/T 1.1—2000 标准化工作导则 第1部分:标准的结构和编写规则.北京:中国标准出版社,2001.

[2] GB/T 12763.6—1991 海洋调查规范 海洋生物调查.北京:中国标准出版社,1991.

[3] GB/T 15919—1995 海洋学术语海洋生物学.北京:中国标准出版社,1996.

[4] GB/T 12763.4—1991 海洋调查规范 海水化学要素观测.北京:中国标准出版社,2004.

[5] 国家海洋局.海洋调查规范 第五分册:海洋生物调查.北京:海洋出版社,1975.

[6] 沈国英,施并章.海洋生态学(第二版).北京:科学出版社,2002.

[7] 焦念志,王荣,黄庆文.^{15}N示踪—离子质谱法测定新生产力的研究.海洋与湖沼,1993,24(1):65-69.

[8] 陈天寿.微生物培养基的制造与应用.北京:中国农业出版社,1995.

[9] 东秀珠,蔡妙英.常见细菌系统鉴定手册.北京:科学出版社,2001.

[10] 邵力平.真菌分类学.北京:中国农业出版社,1984.

[11] 宁修仁,刘子琳,史君贤,等.南极普里兹湾及其邻近海域浮游植物粒度分级生物量和初级生产力.南极研究,1993,5(4):50-62.

[12] 阎逊初.放线菌分类和鉴定.北京:科学出版社,1992.

[13] 宁修仁,蔡昱明,李国为,等.南海北部微微型光合浮游生物的分布及环境调控.海洋学报,2003,25(3):83-97.

[14] 郑重,李少菁,许振组.海洋浮游生物学.北京:海洋出版社,1984.

[15] 张仁斋,陆惠芬,赵传细,等.中国近海鱼卵与仔鱼.上海:上海科学技术出版社,1985.

[16] 环境厅自然保护局(日本).海域自然环境保全基础调查,重要沿岸水域生物调查报告书.1998.

[17] 张志南,李永贵,于子山.黄河口水下三角洲及其邻近水域小型底栖动物的初步研究.海洋与湖沼,1989,20(3):197-207.

[18] 夏世福,刘效瞬.海洋水产资源调查手册(第二版).上海:上海科学技术出版社,1981.

[19] 董正之.世界大洋经济头足类生物学.济南:山东科学技术出版社,1991,2-26.

[20] 郑元甲,金保好.北太平洋海区柔鱼生物学特征初步研究.远洋渔业,1997,4 :59-63.

[21] 赵宪勇,陈毓桢,李显森,等.多种类海洋渔业资源声学评估技术和方法探讨.海洋学报,2003,25(增刊1):192-202.

[22] Dugdale R C, Georing J J. Uptake of new and regenerated forms of nitrogen in primary productivity. Limnology and Oceanography,1967, 23: 196-209.

[23] Jeffrey S W, Mantoura R F C, Wright S W. Phytoplankton pigments in oceanography. UNESCO Publishing, 1997, 327-428.

[24] Ye D Z, Joiris C. Bacterial numbers and biomass in Antarctic water, Chinese Co mmittee on Antarctic Research (ed.), Proceeding of the International Symposium on Antarctic Research, China Ocean Press, 1989, 329-335.

[25] Holt J G. Bergey's Manual of Determinative Bacteriology, 9th Edn, Williams & Wilkins, 1994.

[26] Kurtzman C P, Fell J N. The Yeast, A Taxonomic Study, 4th Edn.,Elsevier, Amsterdam,1998.

[27] Parsons T R. A Manual of Chemical and Biological Methods for Seawater Analysis. Pergamon Press, 1984.
[28] Paul J H. Marine Microbiology—Methods in Microbiology. Acadmic Press, 2001.
[29] SCOR, JGOSF Protocols, UNESCO Publishing, June 1994.
[30] Ning X R, Vaulot D. Standing stock and production of phytoplankton in the estuary of the Changjiangand the adjacent East China Sea Mar. Ecol. Prog. Ser. 1988, 49:141-150.
[31] Wood A M. Discri mination between types of pigments in marine Synechococcus spp by scanning spectroscopy, epifluorescence microscopy and flow cytometry, Limnol. Oceanogr, 1985, 30:1303-1315.
[32] Sournia A. Phytoplankton Manual, Monographs on Oceanographic Methodology, UNESCO, Paris, 1978, 1-337.
[33] 中谷敏邦.鱼卵仔稚鱼わよひ饵生物の采集法.海洋と生命,1987,9(2).
[34] Harris R. ICES Zooplankton Methodology Mannual. Acad Press, 2000, 1-684.
[35] Holme N A, Mclntyre A D. Methods for the Study of Marine Benthos. Blackwell Scientific Publications. London, 1984, 1-387.
[36] Higgins R P, Thiel H. Introduction to the Study of Meiofauna. The Smithsonian Institution Press Washington, D. C. , 1988.
[37] MacLennan D N, Simmonds E J. Fisheries Acoustics. London: Chapman & Hall. 1992.
[38] Foote K G, Knudsen H P, Vestnes G, et al. Calibration of acoustic instruments for fish density estimation: a practical guide. ICES Coop. Res. Rep. No. 144, 1987.

GB/T 12763.6—2007

中 华 人 民 共 和 国

国 家 标 准

海洋调查规范

第 6 部分:海洋生物调查

GB/T 12763.6—2007

*

中国标准出版社出版发行

北京复兴门外三里河北街 16 号

邮政编码:100045

网址 www.spc.net.cn

电话:68523946 68517548

中国标准出版社秦皇岛印刷厂印刷

各地新华书店经销

*

开本 880×1230 1/16 印张 10.75 字数 313 千字

2007 年 12 月第一版 2007 年 12 月第一次印刷

*

GB/T 12763.6-2007

如有印装差错 由本社发行中心调换

版权专有 侵权必究

举报电话:(010)68533533
